# Supplementary material for: Discovery of bone morphogenetic protein 7-derived peptide sequences that attenuate the human osteoarthritic chondrocyte phenotype
Source: Mol Ther Methods Clin Dev. 2021 Mar 17;21:247–61. doi: 10.1016/j.omtm.2021.03.009 (PMC8022858; doi:10.1016/j.omtm.2021.03.009)
Supplement: Document S1. Figures S1–S6 and Tables S1–S13 [file mmc1.pdf]

OMTM, Volume *21*

Supplemental information

Discovery of bone morphogenetic protein 7-derived peptide sequences that attenuate the human osteoarthritic chondrocyte phenotype

Marjolein M.J. Caron, Ellen G.J. Ripmeester, Guus van den Akker, Nina K.A. P. Wijnands, Jessica Steijns, Don A.M. Surtel, Andy Cremers, Pieter J. Emans, Lodewijk W. van Rhijn, and Tim J.M. Welting

SUPPLEMENTARY FIGURES

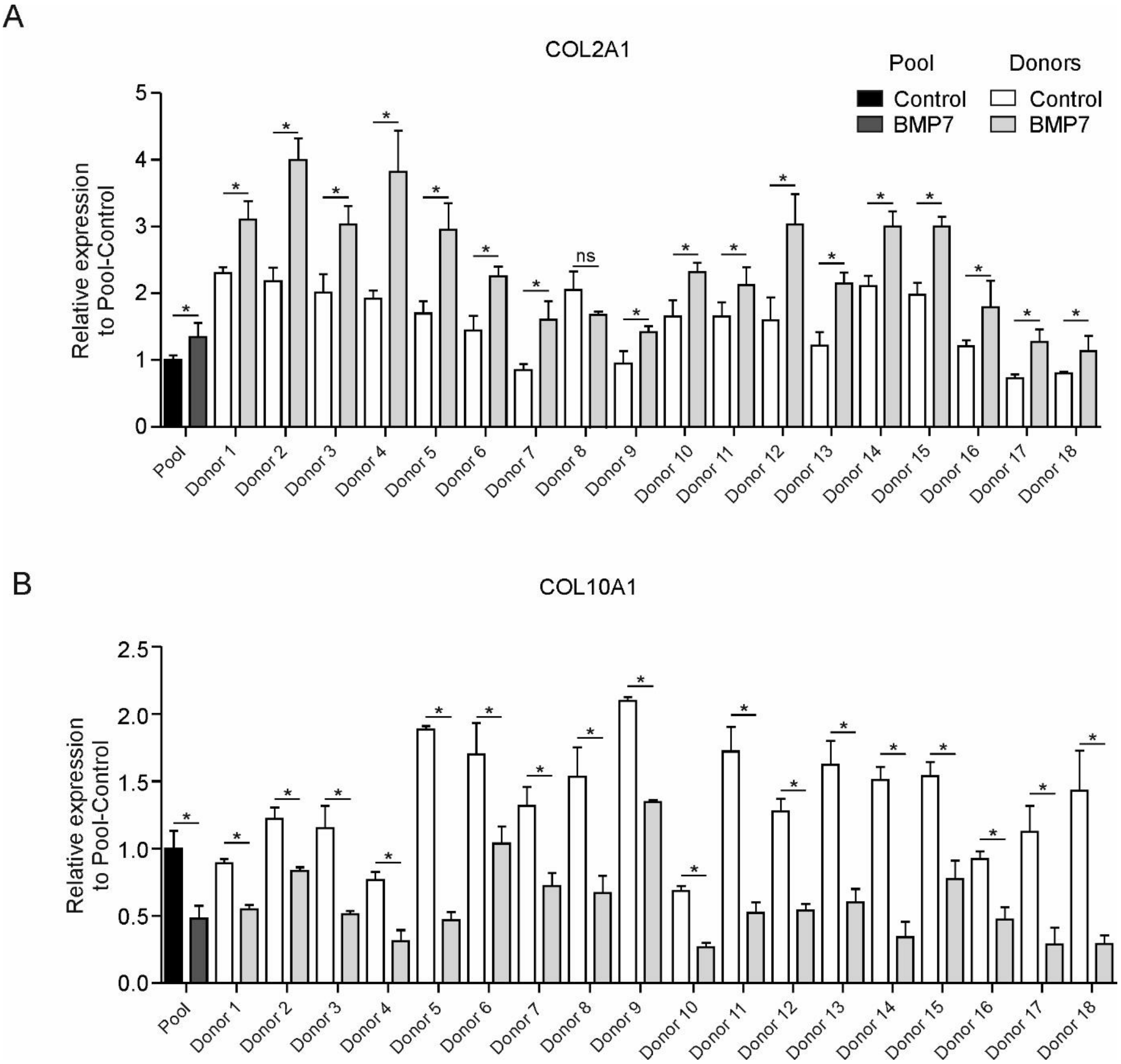

Supplementary Figure 1: Validation of BMP7 responsiveness of OA-HACs and pool. A: COL2A1 mRNA expression of OA-HAC pool and its individual donors in response to BMP7 (1 nM) for 24 hours. **B:** COL10A1 mRNA expression of OA-HAC pool and its individual donors in response to BMP7 (1 nM) for 24 hours. Gene expression was normalized for 28S rRNA expression and set relative to Pool Control condition. Error bars represent mean ± SEM and statistical significance for BMP7 versus control condition for each donor and the pool as determined by unpaired two-tailed student’s t-test is represented as: * is p<0.05, ** is p<0.01, *** is p<0.001 and ns is=not significant.

Supplementary Figure 2: Full RT-qPCR library screening data for SOX9, COL2A1, NKX3-2, RUNX2, COL10A1, ALPL, COX-2, MMP13, ADAMTS5, IL6 mRNAs and for all peptide concentrations tested. **A:** SOX9, **B:** COL2A1, **C:** NKX3-2 mRNAs expression in OA-HAC pool following 24 hours exposure to individual BMP7 library peptides at concentrations of 1000, 10, 10 or 1 nM. Controls represent vehicle and full-length recombinant BMP7 (1 nM). Peptide identities are indicated and are organized from the N-terminus toward C-terminus of BMP7. **D/E:** RUNX2, **F/G:** COL10A1, **H/I:** ALPL, **J/K:** COX-2, **L/M:** MMP13, **N/O:** ADAMTS5, **P/Q:** IL6; same as above, but shown with or without y-axis interruption. Gene expression was normalized for 28S rRNA expression and set relative to Pool Control condition. Error bars represent mean ± SEM.

A

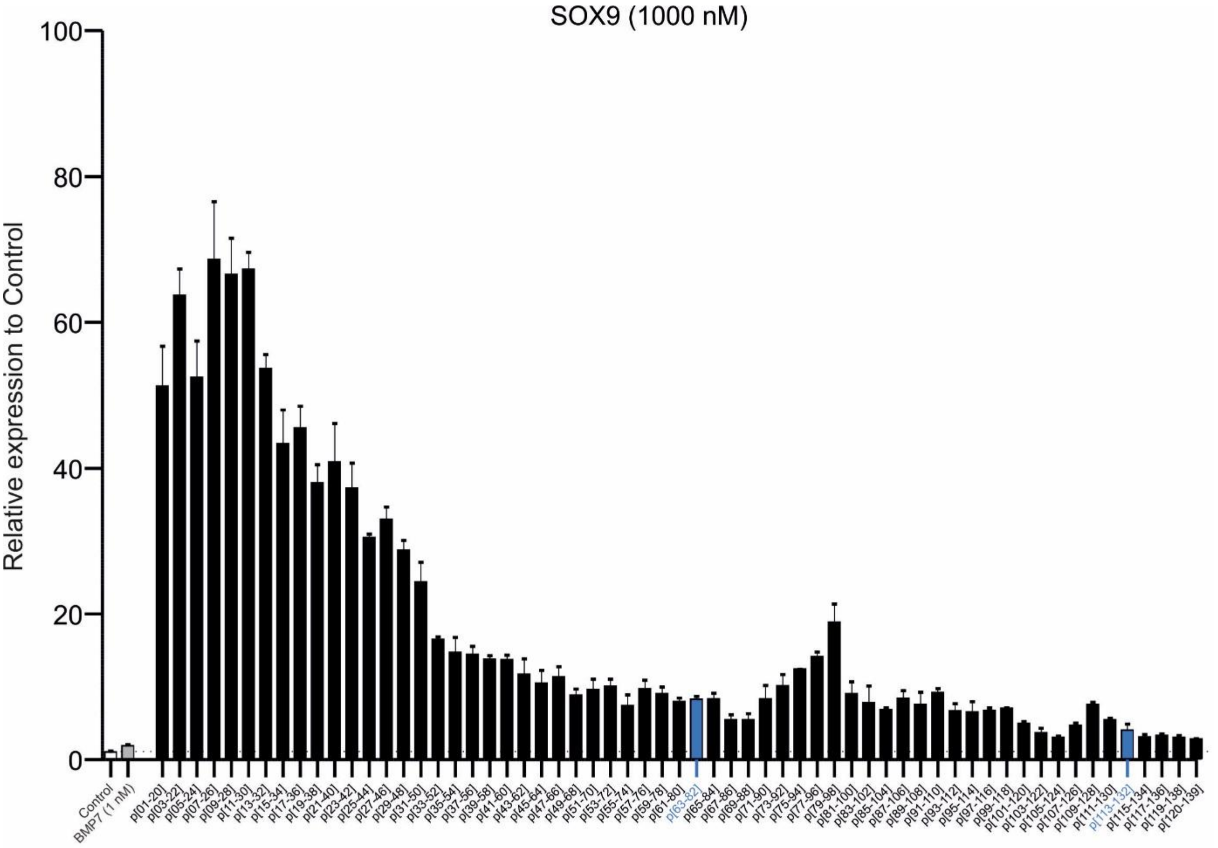
SOX9 (1000 nM)
Relative expression to Control
100
80
60
40
20
0
Control
BMP7 (1 nM)

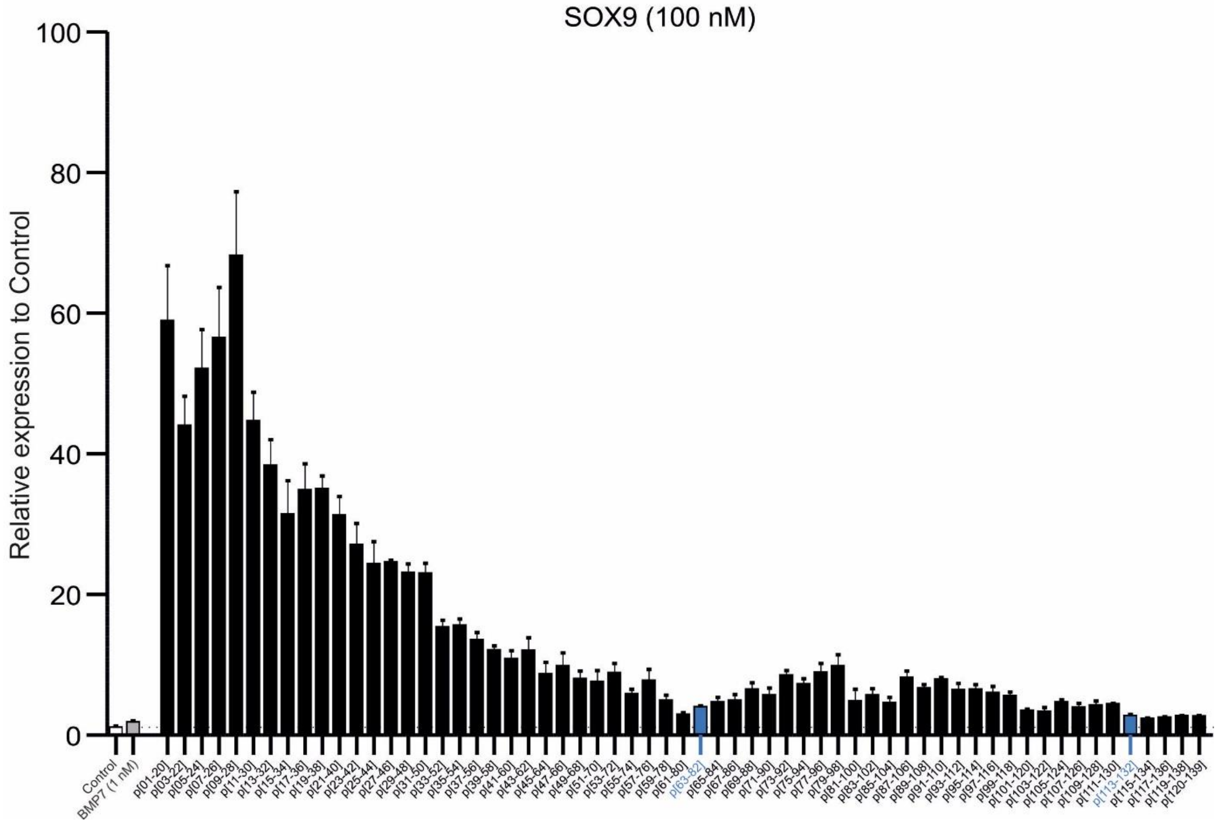
SOX9 (100 nM)
Relative expression to Control
100
80
60
40
20
0
Control
BMP7 (1 nM)

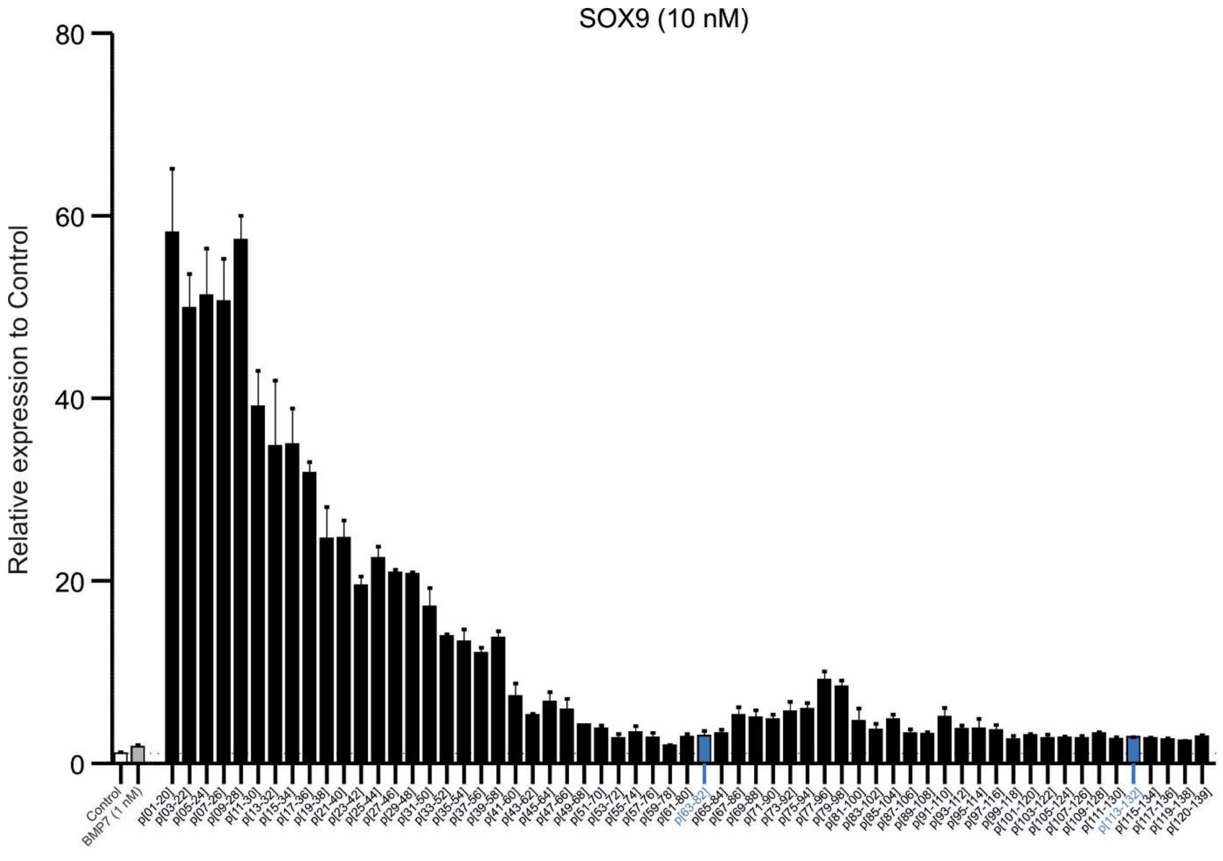
SOX9 (10 nM)
Relative expression to Control
80
60
40
20
0
Control
BMP7 (1 nM)
p[01-20]
p[03-22]
p[05-24]
p[07-26]
p[09-28]
p[11-30]
p[13-32]
p[15-34]
p[17-36]
p[19-38]
p[21-40]
p[23-42]
p[25-44]
p[27-46]
p[29-48]
p[31-50]
p[33-52]
p[35-54]
p[37-56]
p[39-58]
p[41-60]
p[43-62]
p[45-64]
p[47-66]
p[49-68]
p[51-70]
p[53-72]
p[55-74]
p[57-76]
p[59-78]
p[61-80]
p[63-82]
p[65-84]
p[67-86]
p[69-88]
p[71-90]
p[73-92]
p[75-94]
p[77-96]
p[79-98]
p[81-100]
p[83-102]
p[85-104]
p[87-106]
p[89-108]
p[91-110]
p[93-112]
p[95-114]
p[97-116]
p[99-118]
p[101-120]
p[103-122]
p[105-124]
p[107-126]
p[109-128]
p[111-130]
p[113-132]
p[115-134]
p[117-136]
p[119-138]
p[120-139]

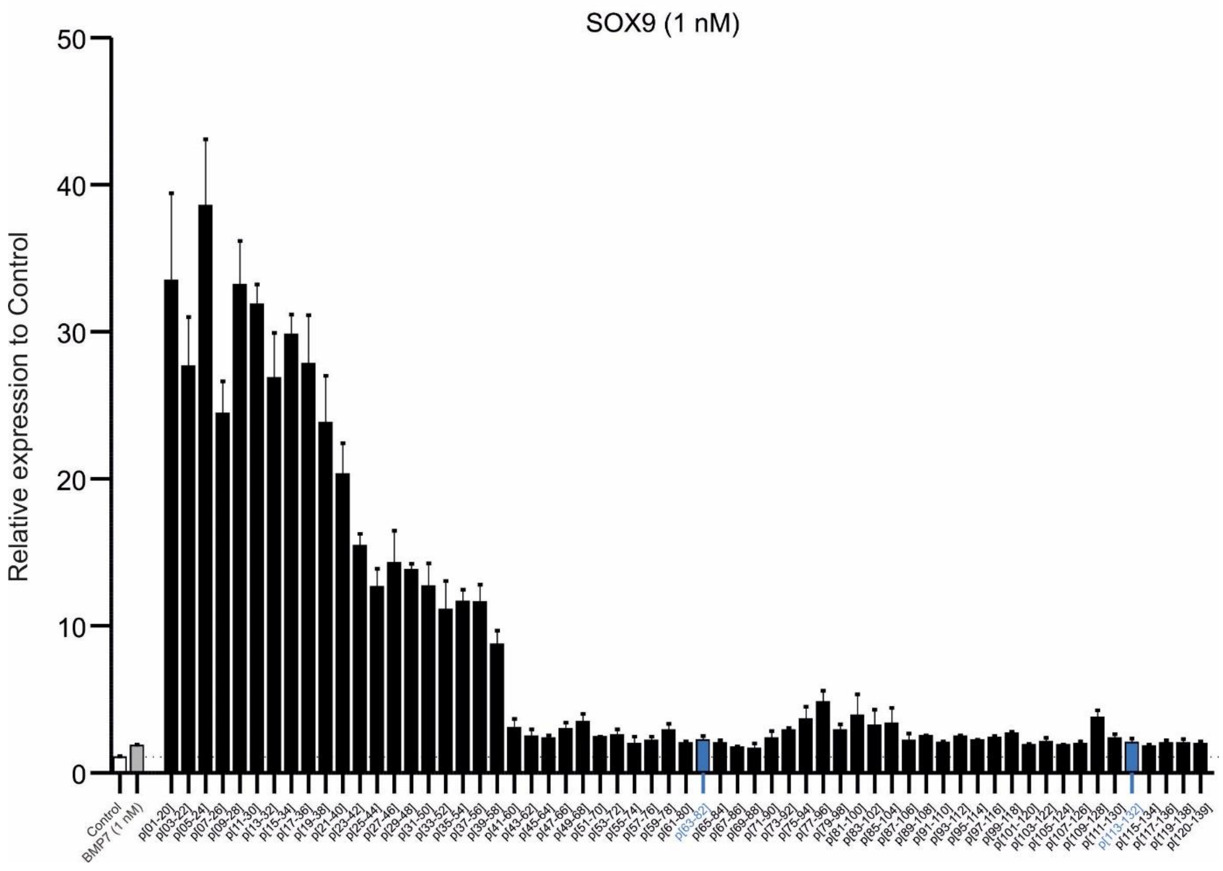
SOX9 (1 nM)
Relative expression to Control
50
40
30
20
10
0
Control
BMP7 (1 nM)
p[01-20]
p[03-22]
p[05-24]
p[07-26]
p[09-28]
p[11-30]
p[13-32]
p[15-34]
p[17-36]
p[19-38]
p[21-40]
p[23-42]
p[25-44]
p[27-46]
p[29-48]
p[31-50]
p[33-52]
p[35-54]
p[37-56]
p[39-58]
p[41-60]
p[43-62]
p[45-64]
p[47-66]
p[49-68]
p[51-70]
p[53-72]
p[55-74]
p[57-76]
p[59-78]
p[61-80]
p[63-82]
p[65-84]
p[67-86]
p[69-88]
p[71-90]
p[73-92]
p[75-94]
p[77-96]
p[79-98]
p[81-100]
p[83-102]
p[85-104]
p[87-106]
p[89-108]
p[91-110]
p[93-112]
p[95-114]
p[97-116]
p[99-118]
p[101-120]
p[103-122]
p[105-124]
p[107-126]
p[109-128]
p[111-130]
p[113-132]
p[115-134]
p[117-136]
p[119-138]
p[120-139]

B

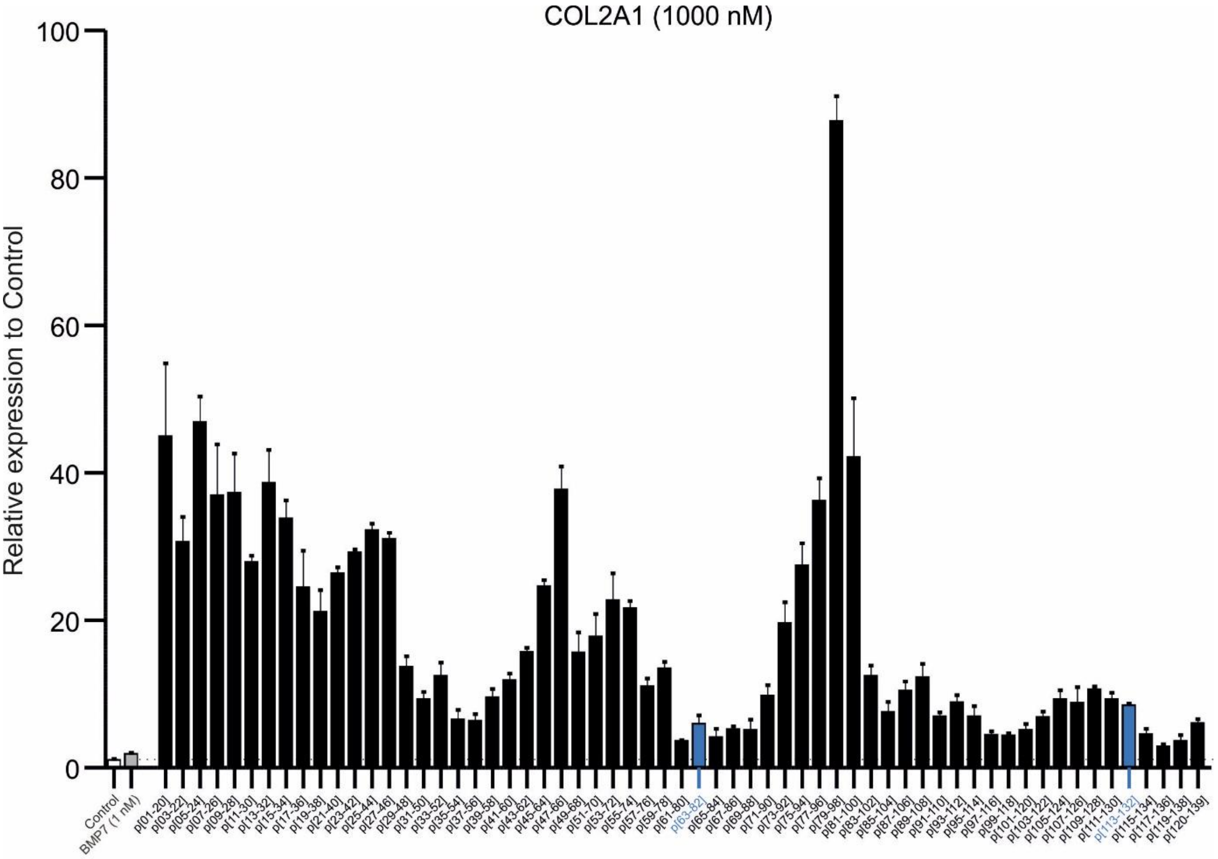
COL2A1 (1000 nM)
Relative expression to Control
100
80
60
40
20
0
Control
BMP7 (1 nM)
p[01-20]
p[03-22]
p[05-24]
p[07-26]
p[09-28]
p[11-30]
p[13-32]
p[15-34]
p[17-36]
p[19-38]
p[21-40]
p[23-42]
p[25-44]
p[27-46]
p[29-48]
p[31-50]
p[33-52]
p[35-54]
p[37-56]
p[39-58]
p[41-60]
p[43-62]
p[45-64]
p[47-66]
p[49-68]
p[51-70]
p[53-72]
p[55-74]
p[57-76]
p[59-78]
p[61-80]
p[63-82]
p[65-84]
p[67-86]
p[69-88]
p[71-90]
p[73-92]
p[75-94]
p[77-96]
p[79-98]
p[81-100]
p[83-102]
p[85-104]
p[87-106]
p[89-108]
p[91-110]
p[93-112]
p[95-114]
p[97-116]
p[99-118]
p[101-120]
p[103-122]
p[105-124]
p[107-126]
p[109-128]
p[111-130]
p[113-132]
p[115-134]
p[117-136]
p[119-138]
p[120-139]

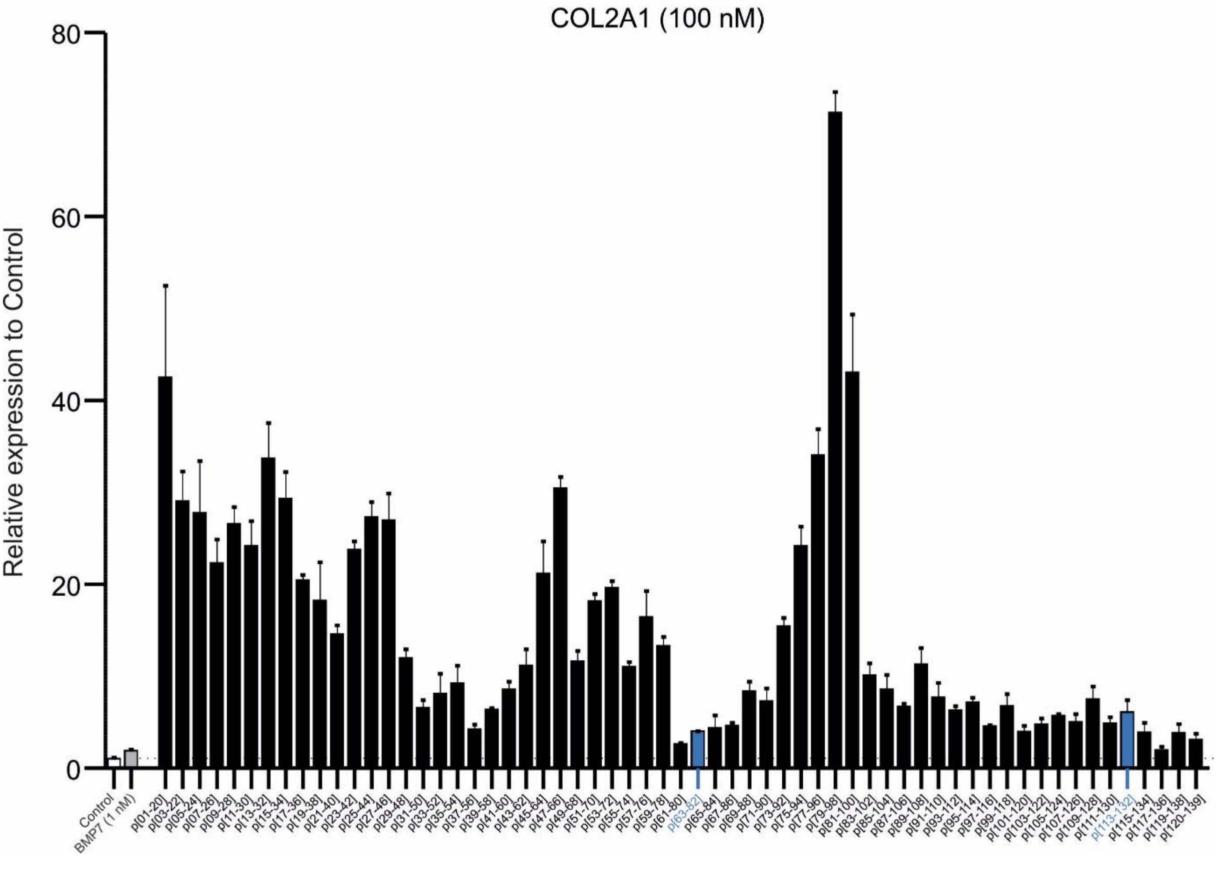
COL2A1 (100 nM)
Relative expression to Control
80
60
40
20
0
Control
BMP7 (1 nM)
p[01-20]
p[03-22]
p[05-24]
p[07-26]
p[09-28]
p[11-30]
p[13-32]
p[15-34]
p[17-36]
p[19-38]
p[21-40]
p[23-42]
p[25-44]
p[27-46]
p[29-48]
p[31-50]
p[33-52]
p[35-54]
p[37-56]
p[39-58]
p[41-60]
p[43-62]
p[45-64]
p[47-66]
p[49-68]
p[51-70]
p[53-72]
p[55-74]
p[57-76]
p[59-78]
p[61-80]
p[63-82]
p[65-84]
p[67-86]
p[69-88]
p[71-90]
p[73-92]
p[75-94]
p[77-96]
p[79-98]
p[81-100]
p[83-102]
p[85-104]
p[87-106]
p[89-108]
p[91-110]
p[93-112]
p[95-114]
p[97-116]
p[99-118]
p[101-120]
p[103-122]
p[105-124]
p[107-126]
p[109-128]
p[111-130]
p[113-132]
p[115-134]
p[117-136]
p[119-138]
p[120-139]

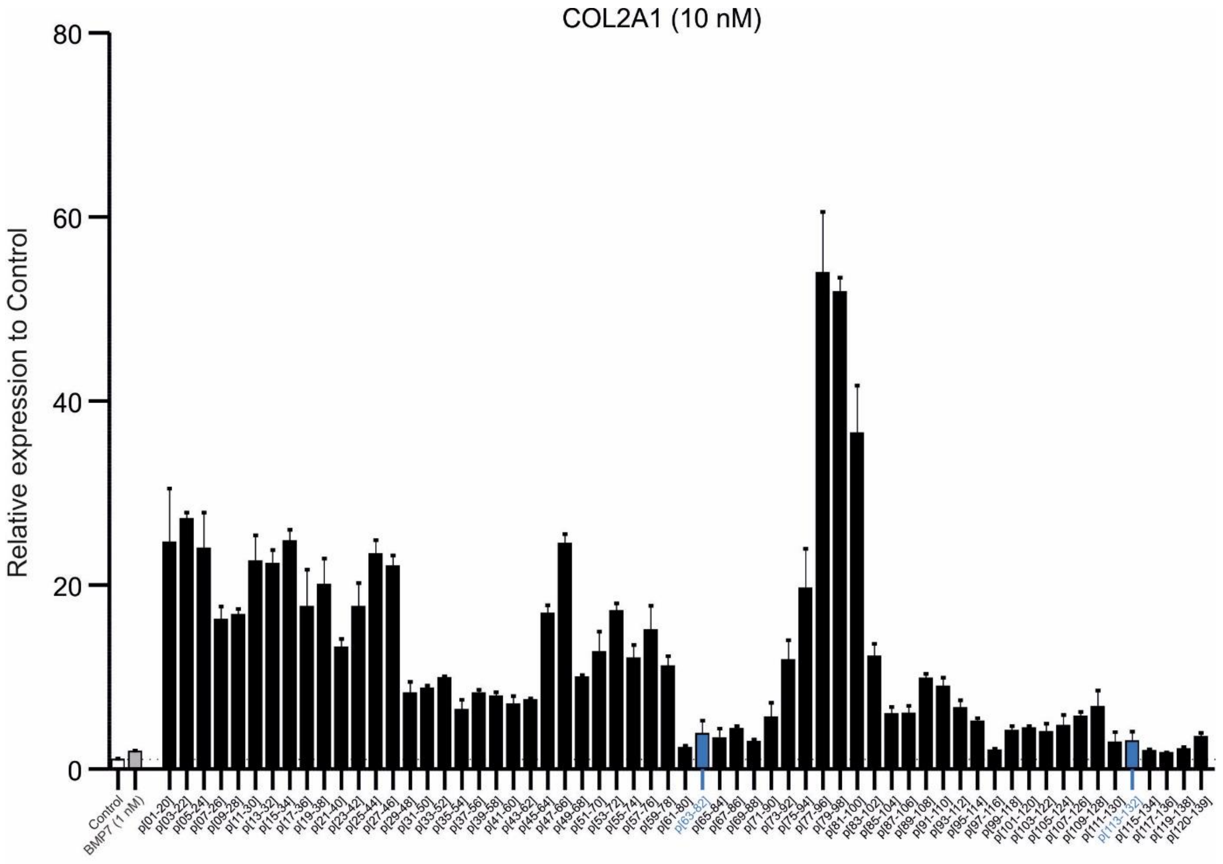
COL2A1 (10 nM)
Relative expression to Control
0
20
40
60
80
Control
BMP7 (1 nM)
p[01-20]
p[03-22]
p[05-24]
p[07-26]
p[09-28]
p[11-30]
p[13-32]
p[15-34]
p[17-36]
p[19-38]
p[21-40]
p[23-42]
p[25-44]
p[27-46]
p[29-48]
p[31-50]
p[33-52]
p[35-54]
p[37-56]
p[39-58]
p[41-60]
p[43-62]
p[45-64]
p[47-66]
p[49-68]
p[51-70]
p[53-72]
p[55-74]
p[57-76]
p[59-78]
p[61-80]
p[63-82]
p[65-84]
p[67-86]
p[69-88]
p[71-90]
p[73-92]
p[75-94]
p[77-96]
p[79-98]
p[81-100]
p[83-102]
p[85-104]
p[87-106]
p[89-108]
p[91-110]
p[93-112]
p[95-114]
p[97-116]
p[99-118]
p[101-120]
p[103-122]
p[105-124]
p[107-126]
p[109-128]
p[111-130]
p[113-132]
p[115-134]
p[117-136]
p[119-138]
p[120-139]

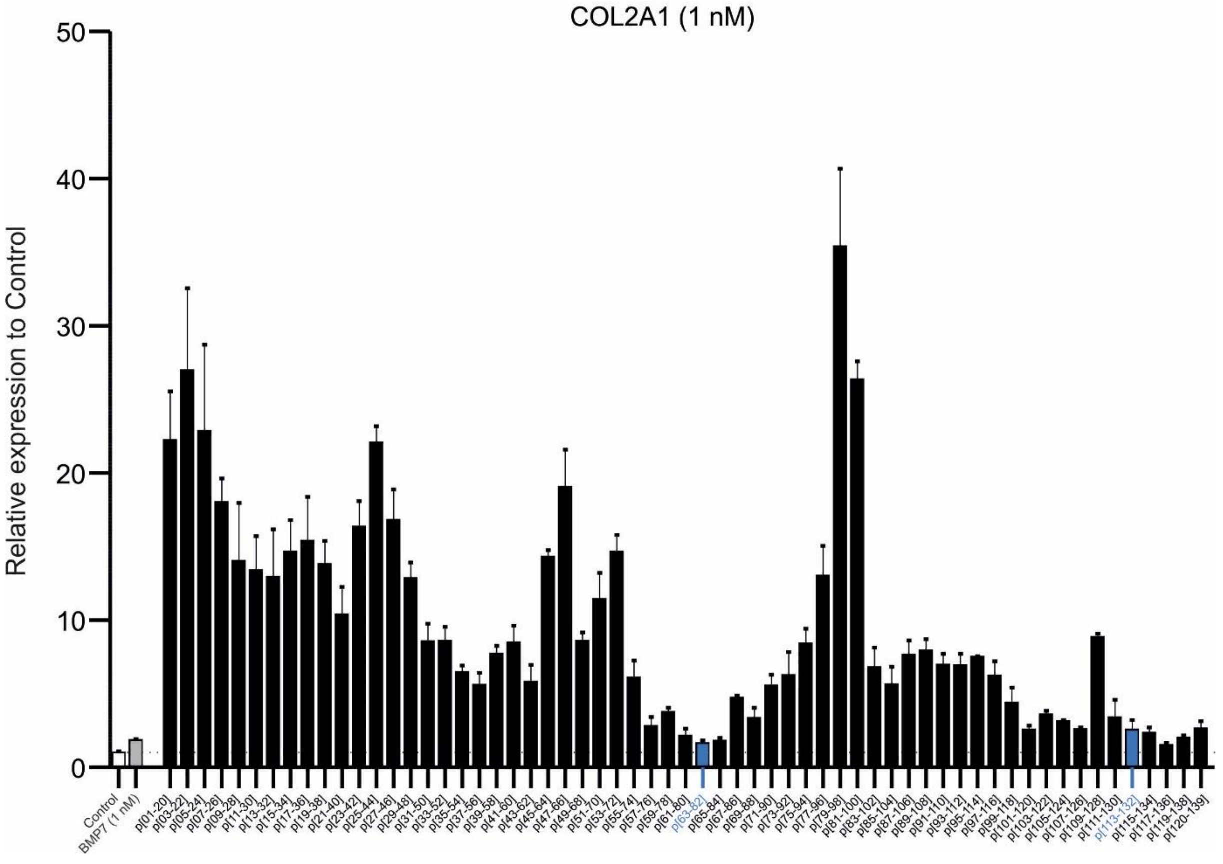
COL2A1 (1 nM)
Relative expression to Control
0
10
20
30
40
50
Control
BMP7 (1 nM)
p[01-20]
p[03-22]
p[05-24]
p[07-26]
p[09-28]
p[11-30]
p[13-32]
p[15-34]
p[17-36]
p[19-38]
p[21-40]
p[23-42]
p[25-44]
p[27-46]
p[29-48]
p[31-50]
p[33-52]
p[35-54]
p[37-56]
p[39-58]
p[41-60]
p[43-62]
p[45-64]
p[47-66]
p[49-68]
p[51-70]
p[53-72]
p[55-74]
p[57-76]
p[59-78]
p[61-80]
p[63-82]
p[65-84]
p[67-86]
p[69-88]
p[71-90]
p[73-92]
p[75-94]
p[77-96]
p[79-98]
p[81-100]
p[83-102]
p[85-104]
p[87-106]
p[89-108]
p[91-110]
p[93-112]
p[95-114]
p[97-116]
p[99-118]
p[101-120]
p[103-122]
p[105-124]
p[107-126]
p[109-128]
p[111-130]
p[113-132]
p[115-134]
p[117-136]
p[119-138]
p[120-139]

C

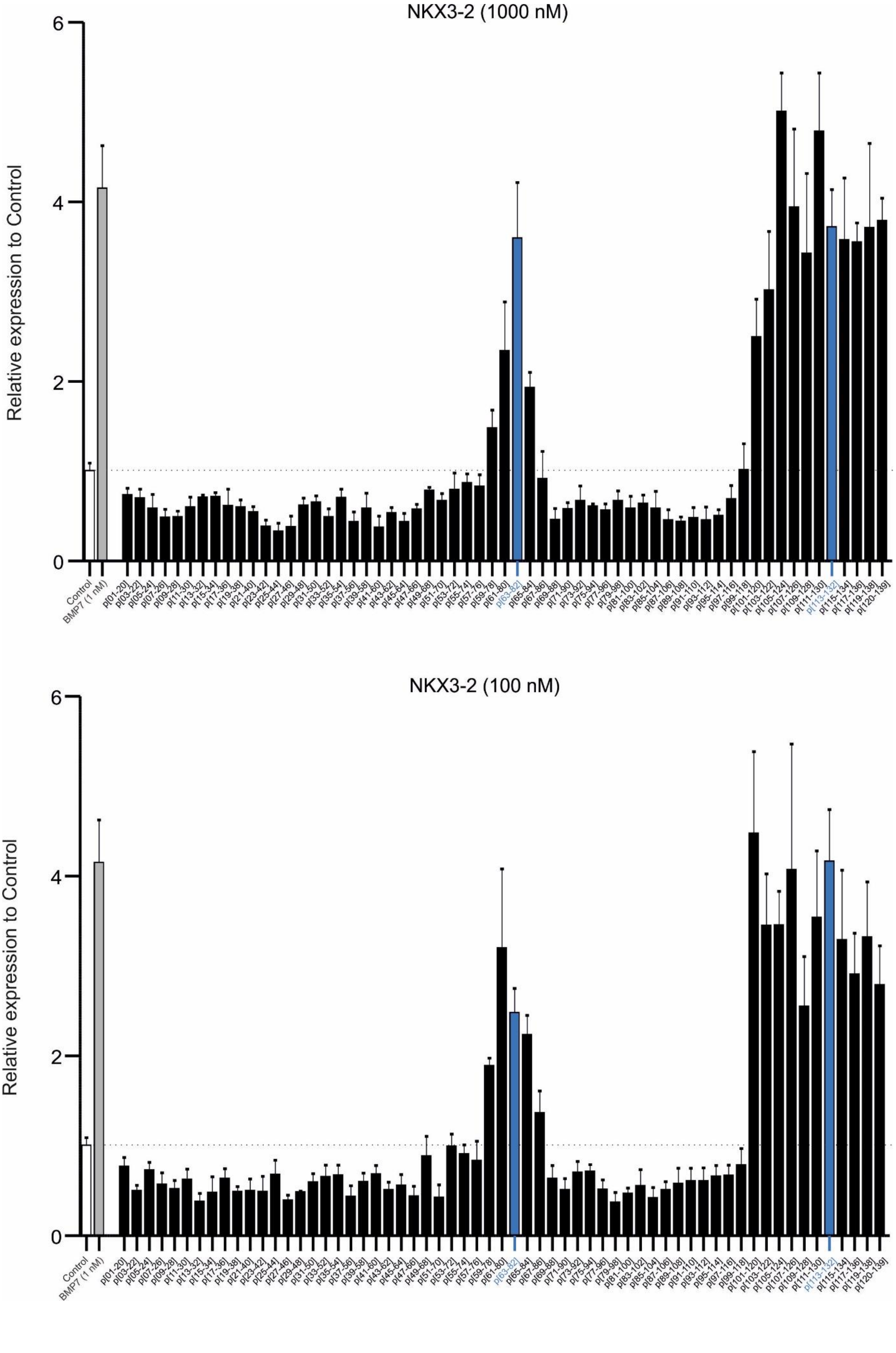
NKX3-2 (1000 nM)
Relative expression to Control
0
2
4
6
Control
BMP7 (1 nM)
p[01-20] p[03-22] p[05-24] p[07-26] p[09-28] p[11-30] p[13-32] p[15-34] p[17-36] p[19-38] p[21-40] p[23-42] p[25-44] p[27-46] p[29-48] p[31-50] p[33-52] p[35-54] p[37-56] p[39-58] p[41-60] p[43-62] p[45-64] p[47-66] p[49-68] p[51-70] p[53-72] p[55-74] p[57-76] p[59-78] p[61-80] p[63-82] p[65-84] p[67-86] p[69-88] p[71-90] p[73-92] p[75-94] p[77-96] p[79-98] p[81-100] p[83-102] p[85-104] p[87-106] p[89-108] p[91-110] p[93-112] p[95-114] p[97-116] p[99-118] p[101-120] p[103-122] p[105-124] p[107-126] p[109-128] p[111-130] p[113-132] p[115-134] p[117-136] p[119-138] p[120-139]
NKX3-2 (100 nM)
Relative expression to Control
0
2
4
6
Control
BMP7 (1 nM)
p[01-20] p[03-22] p[05-24] p[07-26] p[09-28] p[11-30] p[13-32] p[15-34] p[17-36] p[19-38] p[21-40] p[23-42] p[25-44] p[27-46] p[29-48] p[31-50] p[33-52] p[35-54] p[37-56] p[39-58] p[41-60] p[43-62] p[45-64] p[47-66] p[49-68] p[51-70] p[53-72] p[55-74] p[57-76] p[59-78] p[61-80] p[63-82] p[65-84] p[67-86] p[69-88] p[71-90] p[73-92] p[75-94] p[77-96] p[79-98] p[81-100] p[83-102] p[85-104] p[87-106] p[89-108] p[91-110] p[93-112] p[95-114] p[97-116] p[99-118] p[101-120] p[103-122] p[105-124] p[107-126] p[109-128] p[111-130] p[113-132] p[115-134] p[117-136] p[119-138] p[120-139]

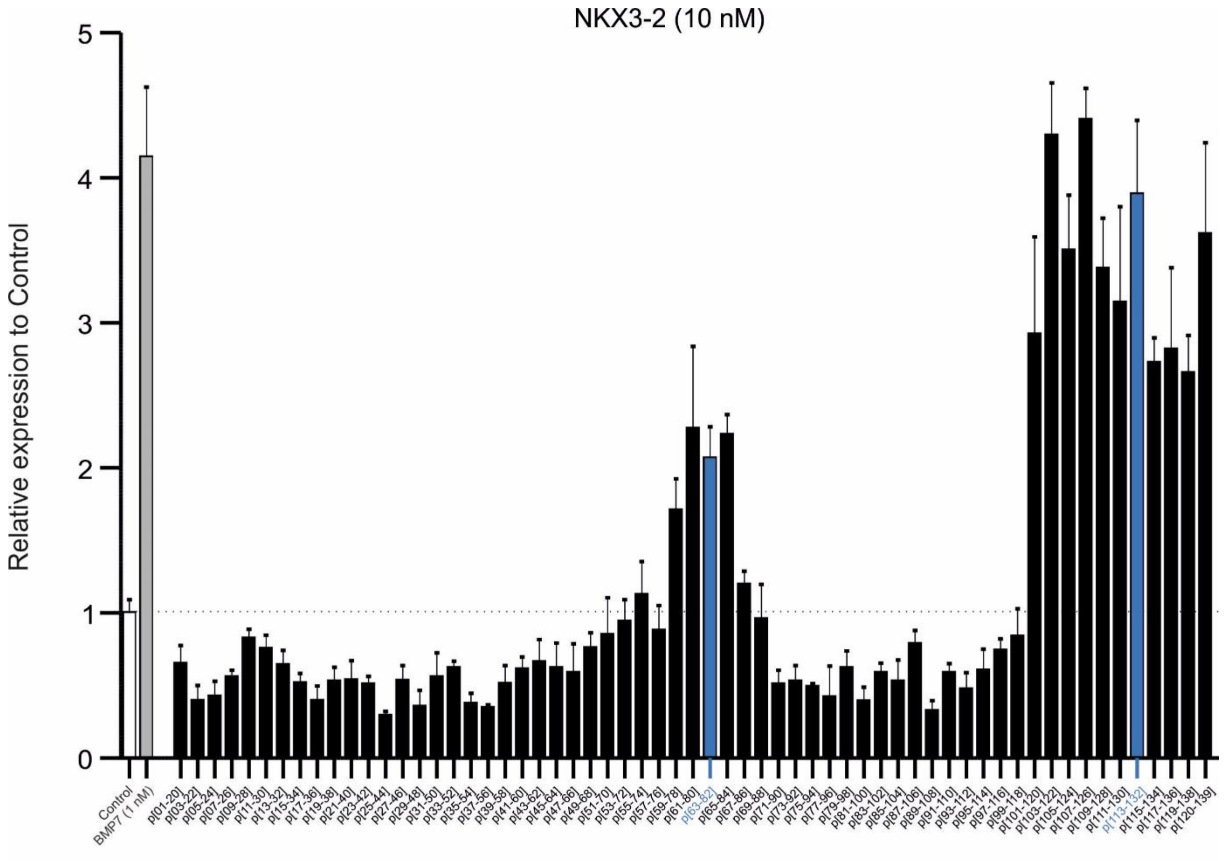
NKX3-2 (10 nM)
Relative expression to Control
0
1
2
3
4
5
Control
BMP7 (1 nM)
p[01-20]
p[03-22]
p[05-24]
p[07-26]
p[09-28]
p[11-30]
p[13-32]
p[15-34]
p[17-36]
p[19-38]
p[21-40]
p[23-42]
p[25-44]
p[27-46]
p[29-48]
p[31-50]
p[33-52]
p[35-54]
p[37-56]
p[39-58]
p[41-60]
p[43-62]
p[45-64]
p[47-66]
p[49-68]
p[51-70]
p[53-72]
p[55-74]
p[57-76]
p[59-78]
p[61-80]
p[63-82]
p[65-84]
p[67-86]
p[69-88]
p[71-90]
p[73-92]
p[75-94]
p[77-96]
p[79-98]
p[81-100]
p[83-102]
p[85-104]
p[87-106]
p[89-108]
p[91-110]
p[93-112]
p[95-114]
p[97-116]
p[99-118]
p[101-120]
p[103-122]
p[105-124]
p[107-126]
p[109-128]
p[111-130]
p[113-132]
p[115-134]
p[117-136]
p[119-138]
p[120-139]

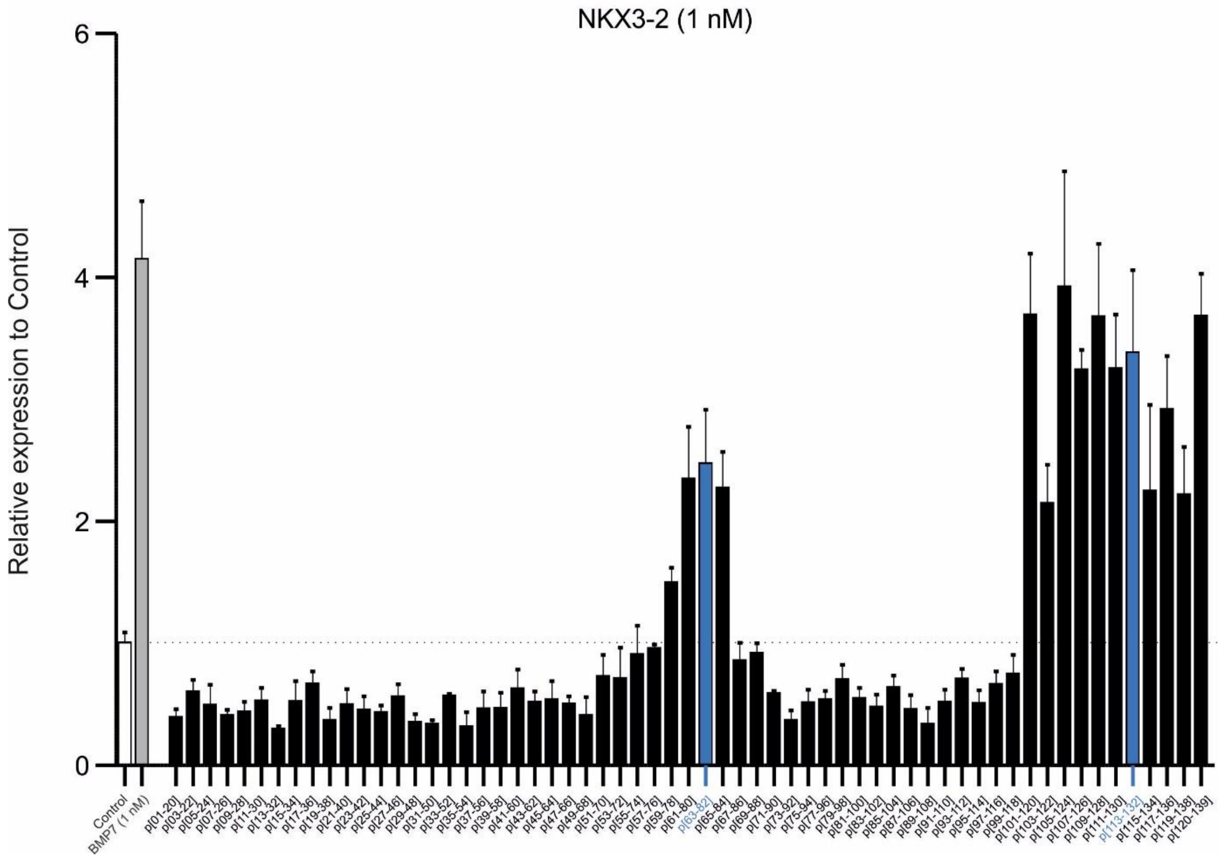
NKX3-2 (1 nM)
Relative expression to Control
0
2
4
6
Control
BMP7 (1 nM)
p[01-20]
p[03-22]
p[05-24]
p[07-26]
p[09-28]
p[11-30]
p[13-32]
p[15-34]
p[17-36]
p[19-38]
p[21-40]
p[23-42]
p[25-44]
p[27-46]
p[29-48]
p[31-50]
p[33-52]
p[35-54]
p[37-56]
p[39-58]
p[41-60]
p[43-62]
p[45-64]
p[47-66]
p[49-68]
p[51-70]
p[53-72]
p[55-74]
p[57-76]
p[59-78]
p[61-80]
p[63-82]
p[65-84]
p[67-86]
p[69-88]
p[71-90]
p[73-92]
p[75-94]
p[77-96]
p[79-98]
p[81-100]
p[83-102]
p[85-104]
p[87-106]
p[89-108]
p[91-110]
p[93-112]
p[95-114]
p[97-116]
p[99-118]
p[101-120]
p[103-122]
p[105-124]
p[107-126]
p[109-128]
p[111-130]
p[113-132]
p[115-134]
p[117-136]
p[119-138]
p[120-139]

D

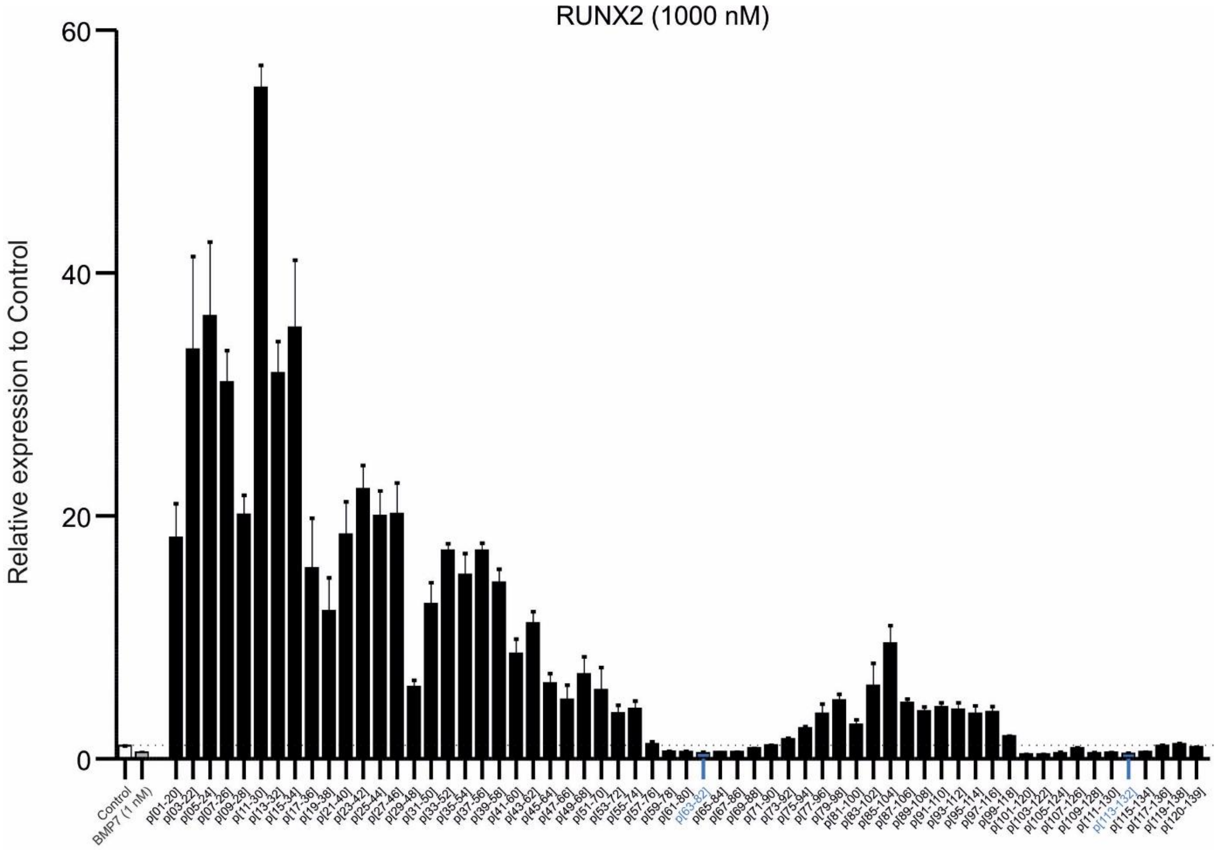
RUNX2 (1000 nM)
Relative expression to Control
60
40
20
0
Control
BMP7 (1 nM)

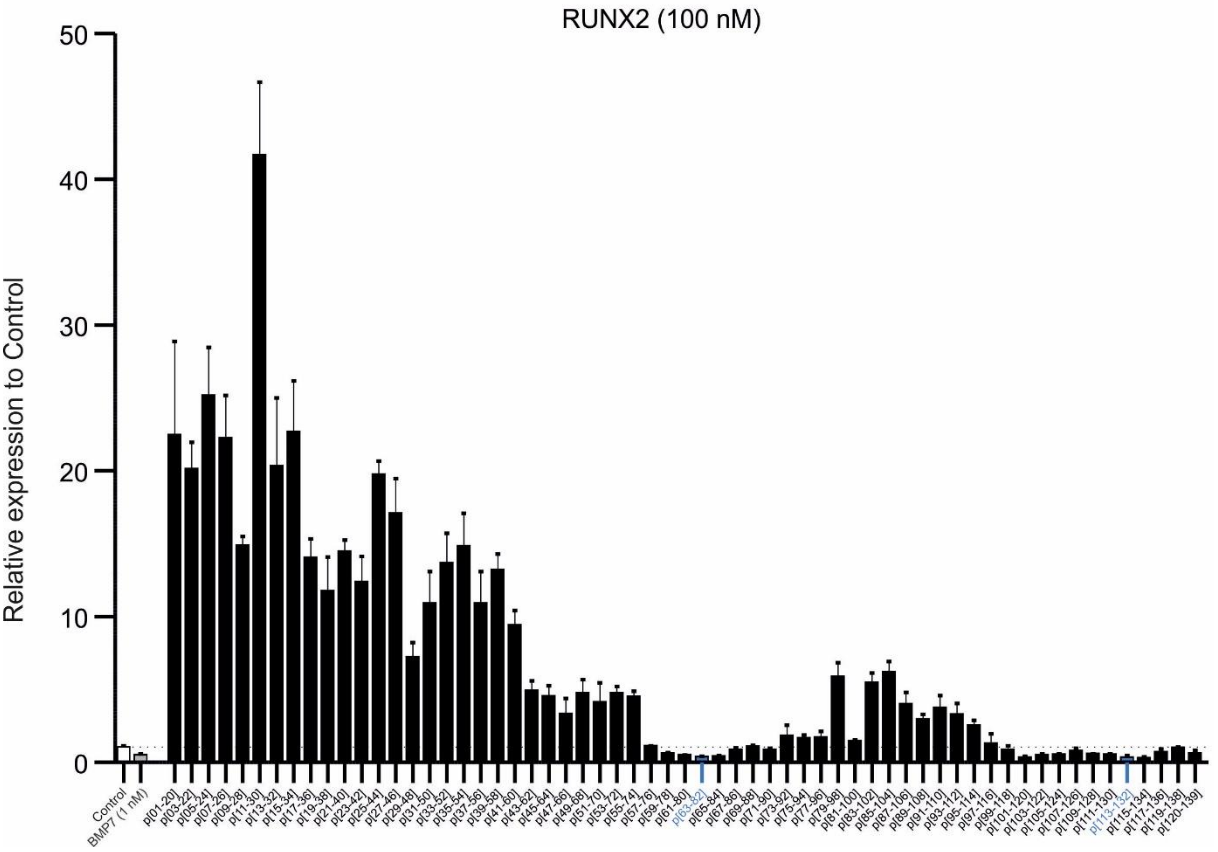
RUNX2 (100 nM)
Relative expression to Control
50
40
30
20
10
0
Control
BMP7 (1 nM)

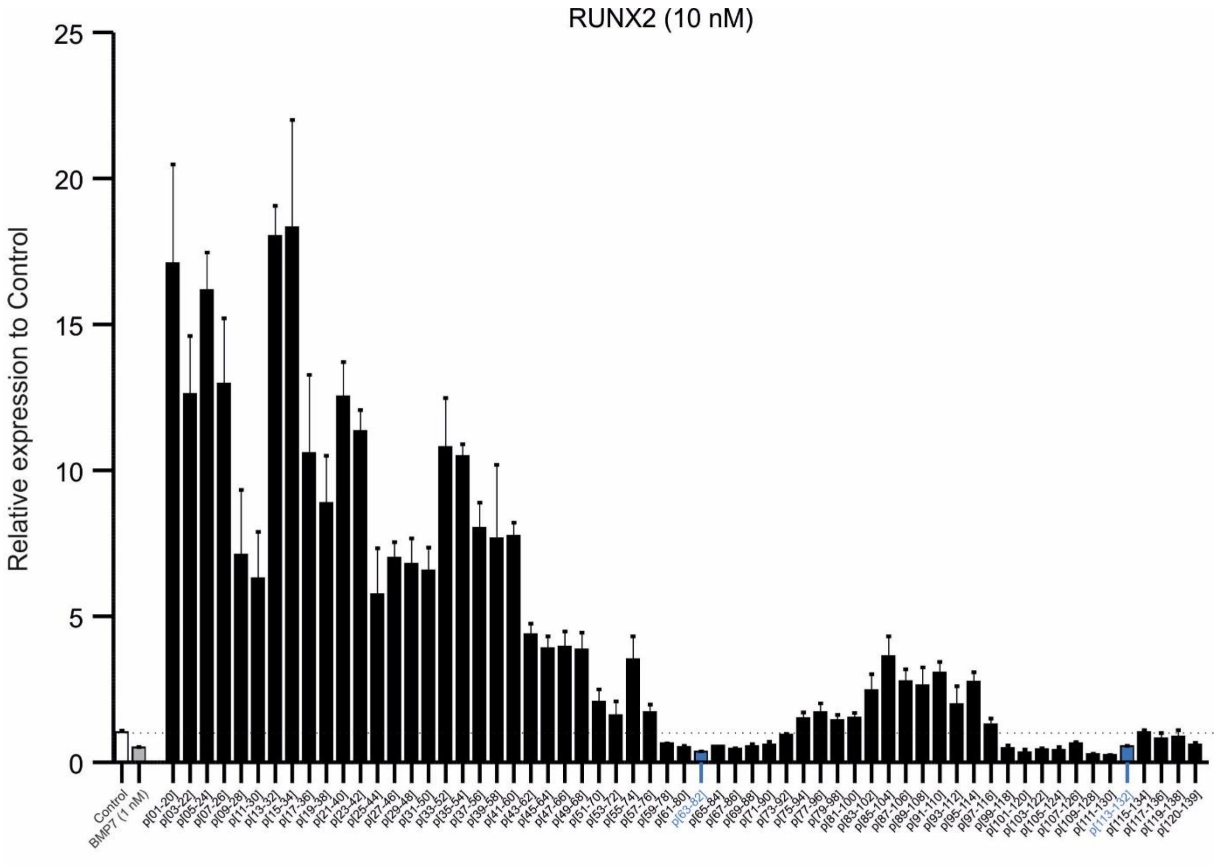
RUNX2 (10 nM)
Relative expression to Control
25
20
15
10
5
0
Control
BMP7 (1 nM)
p[01-20]
p[03-22]
p[05-24]
p[07-26]
p[09-28]
p[11-30]
p[13-32]
p[15-34]
p[17-36]
p[19-38]
p[21-40]
p[23-42]
p[25-44]
p[27-46]
p[29-48]
p[31-50]
p[33-52]
p[35-54]
p[37-56]
p[39-58]
p[41-60]
p[43-62]
p[45-64]
p[47-66]
p[49-68]
p[51-70]
p[53-72]
p[55-74]
p[57-76]
p[59-78]
p[61-80]
p[63-82]
p[65-84]
p[67-86]
p[69-88]
p[71-90]
p[73-92]
p[75-94]
p[77-96]
p[79-98]
p[81-100]
p[83-102]
p[85-104]
p[87-106]
p[89-108]
p[91-110]
p[93-112]
p[95-114]
p[97-116]
p[99-118]
p[101-120]
p[103-122]
p[105-124]
p[107-126]
p[109-128]
p[111-130]
p[113-132]
p[115-134]
p[117-136]
p[119-138]
p[120-139]

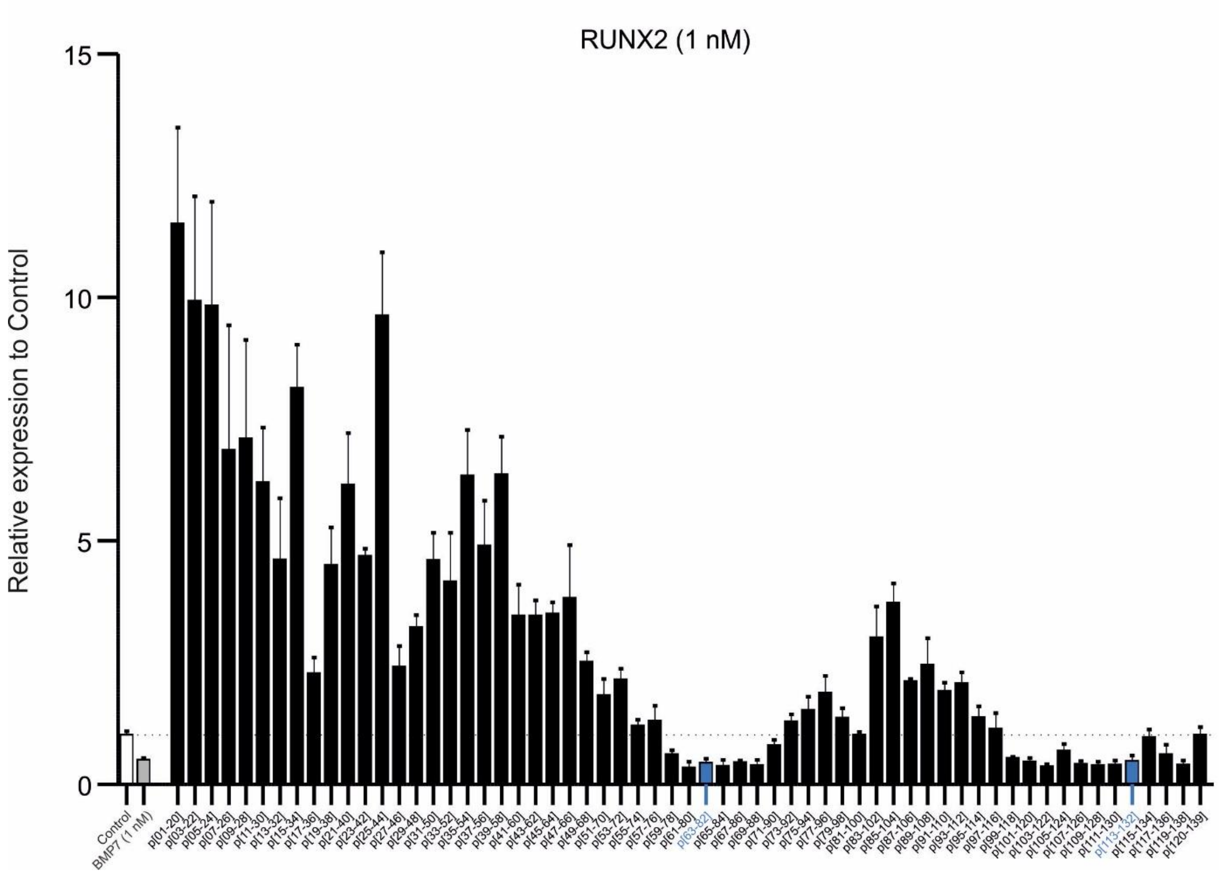
RUNX2 (1 nM)
Relative expression to Control
15
10
5
0
Control
BMP7 (1 nM)
p[01-20]
p[03-22]
p[05-24]
p[07-26]
p[09-28]
p[11-30]
p[13-32]
p[15-34]
p[17-36]
p[19-38]
p[21-40]
p[23-42]
p[25-44]
p[27-46]
p[29-48]
p[31-50]
p[33-52]
p[35-54]
p[37-56]
p[39-58]
p[41-60]
p[43-62]
p[45-64]
p[47-66]
p[49-68]
p[51-70]
p[53-72]
p[55-74]
p[57-76]
p[59-78]
p[61-80]
p[63-82]
p[65-84]
p[67-86]
p[69-88]
p[71-90]
p[73-92]
p[75-94]
p[77-96]
p[79-98]
p[81-100]
p[83-102]
p[85-104]
p[87-106]
p[89-108]
p[91-110]
p[93-112]
p[95-114]
p[97-116]
p[99-118]
p[101-120]
p[103-122]
p[105-124]
p[107-126]
p[109-128]
p[111-130]
p[113-132]
p[115-134]
p[117-136]
p[119-138]
p[120-139]

E

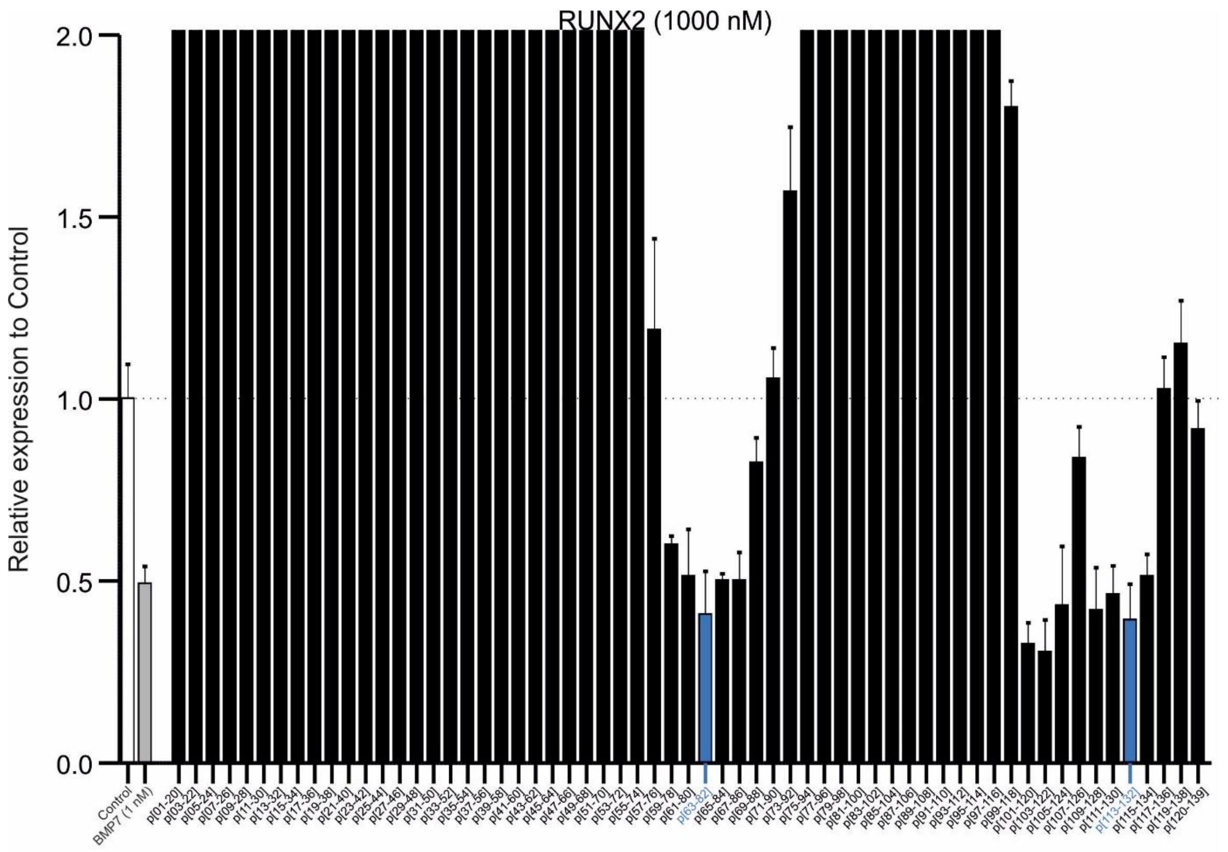
RUNX2 (1000 nM)
Relative expression to Control
2.0
1.5
1.0
0.5
0.0
Control
BMP7 (1 nM)
p[01-20] p[03-22] p[05-24] p[07-26] p[09-28] p[11-30] p[13-32] p[15-34] p[17-36] p[19-38] p[21-40] p[23-42] p[25-44] p[27-46] p[29-48] p[31-50] p[33-52] p[35-54] p[37-56] p[39-58] p[41-60] p[43-62] p[45-64] p[47-66] p[49-68] p[51-70] p[53-72] p[55-74] p[57-76] p[59-78] p[61-80] p[63-82] p[65-84] p[67-86] p[69-88] p[71-90] p[73-92] p[75-94] p[77-96] p[79-98] p[81-100] p[83-102] p[85-104] p[87-106] p[89-108] p[91-110] p[93-112] p[95-114] p[97-116] p[99-118] p[101-120] p[103-122] p[105-124] p[107-126] p[109-128] p[111-130] p[113-132] p[115-134] p[117-136] p[119-138] p[120-139]

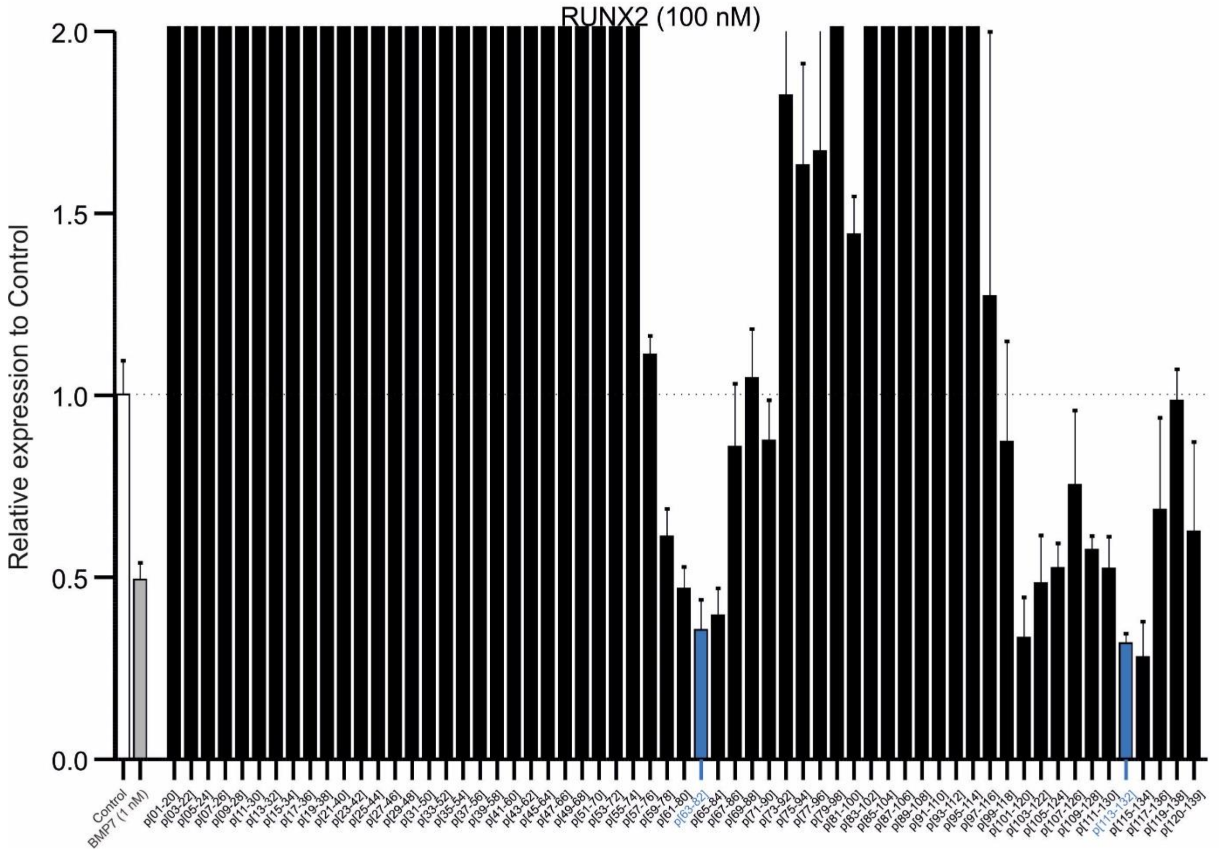
RUNX2 (100 nM)
Relative expression to Control
2.0
1.5
1.0
0.5
0.0
Control
BMP7 (1 nM)
p[01-20] p[03-22] p[05-24] p[07-26] p[09-28] p[11-30] p[13-32] p[15-34] p[17-36] p[19-38] p[21-40] p[23-42] p[25-44] p[27-46] p[29-48] p[31-50] p[33-52] p[35-54] p[37-56] p[39-58] p[41-60] p[43-62] p[45-64] p[47-66] p[49-68] p[51-70] p[53-72] p[55-74] p[57-76] p[59-78] p[61-80] p[63-82] p[65-84] p[67-86] p[69-88] p[71-90] p[73-92] p[75-94] p[77-96] p[79-98] p[81-100] p[83-102] p[85-104] p[87-106] p[89-108] p[91-110] p[93-112] p[95-114] p[97-116] p[99-118] p[101-120] p[103-122] p[105-124] p[107-126] p[109-128] p[111-130] p[113-132] p[115-134] p[117-136] p[119-138] p[120-139]

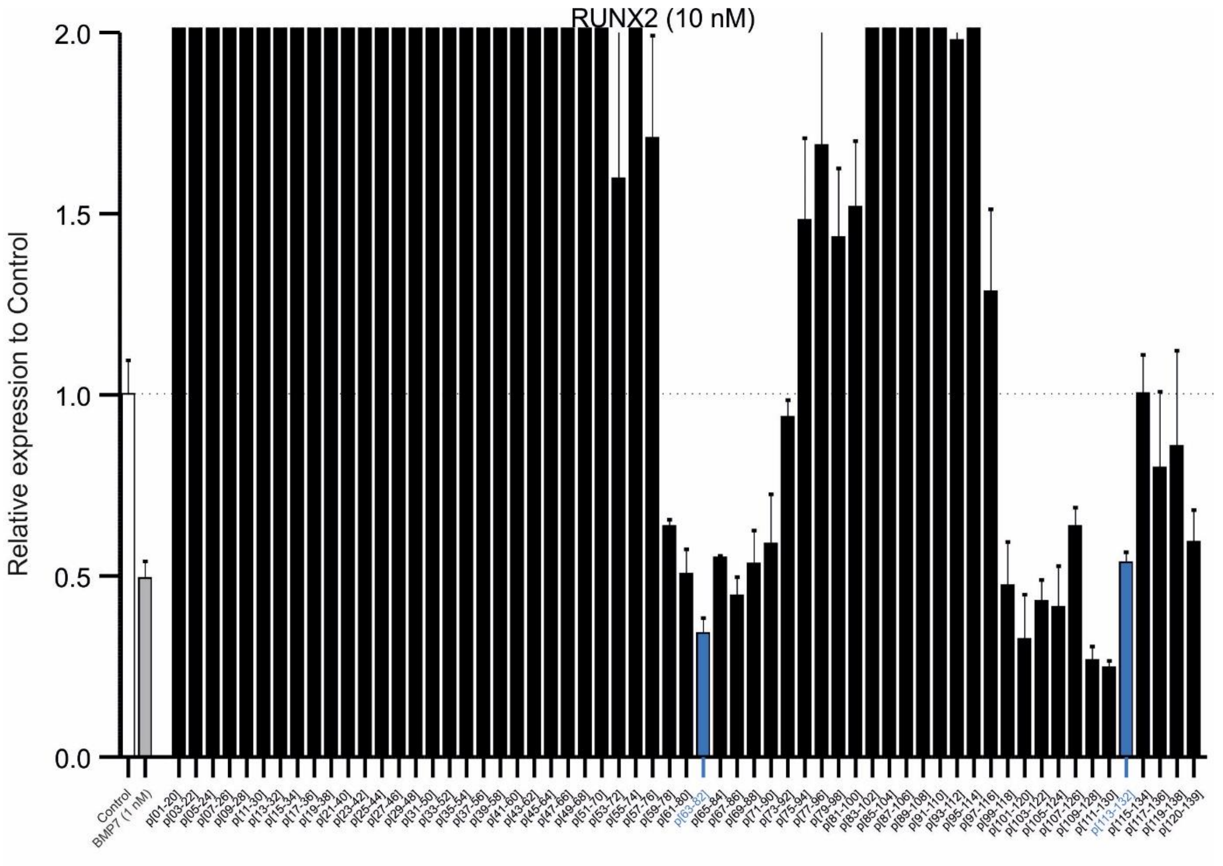
RUNX2 (10 nM)
Relative expression to Control
2.0
1.5
1.0
0.5
0.0
Control
BMP7 (1 nM)
p[01-20]
p[03-22]
p[05-24]
p[07-26]
p[09-28]
p[11-30]
p[13-32]
p[15-34]
p[17-36]
p[19-38]
p[21-40]
p[23-42]
p[25-44]
p[27-46]
p[29-48]
p[31-50]
p[33-52]
p[35-54]
p[37-56]
p[39-58]
p[41-60]
p[43-62]
p[45-64]
p[47-66]
p[49-68]
p[51-70]
p[53-72]
p[55-74]
p[57-76]
p[59-78]
p[61-80]
p[63-82]
p[65-84]
p[67-86]
p[69-88]
p[71-90]
p[73-92]
p[75-94]
p[77-96]
p[79-98]
p[81-100]
p[83-102]
p[85-104]
p[87-106]
p[89-108]
p[91-110]
p[93-112]
p[95-114]
p[97-116]
p[99-118]
p[101-120]
p[103-122]
p[105-124]
p[107-126]
p[109-128]
p[111-130]
p[113-132]
p[115-134]
p[117-136]
p[119-138]
p[120-139]

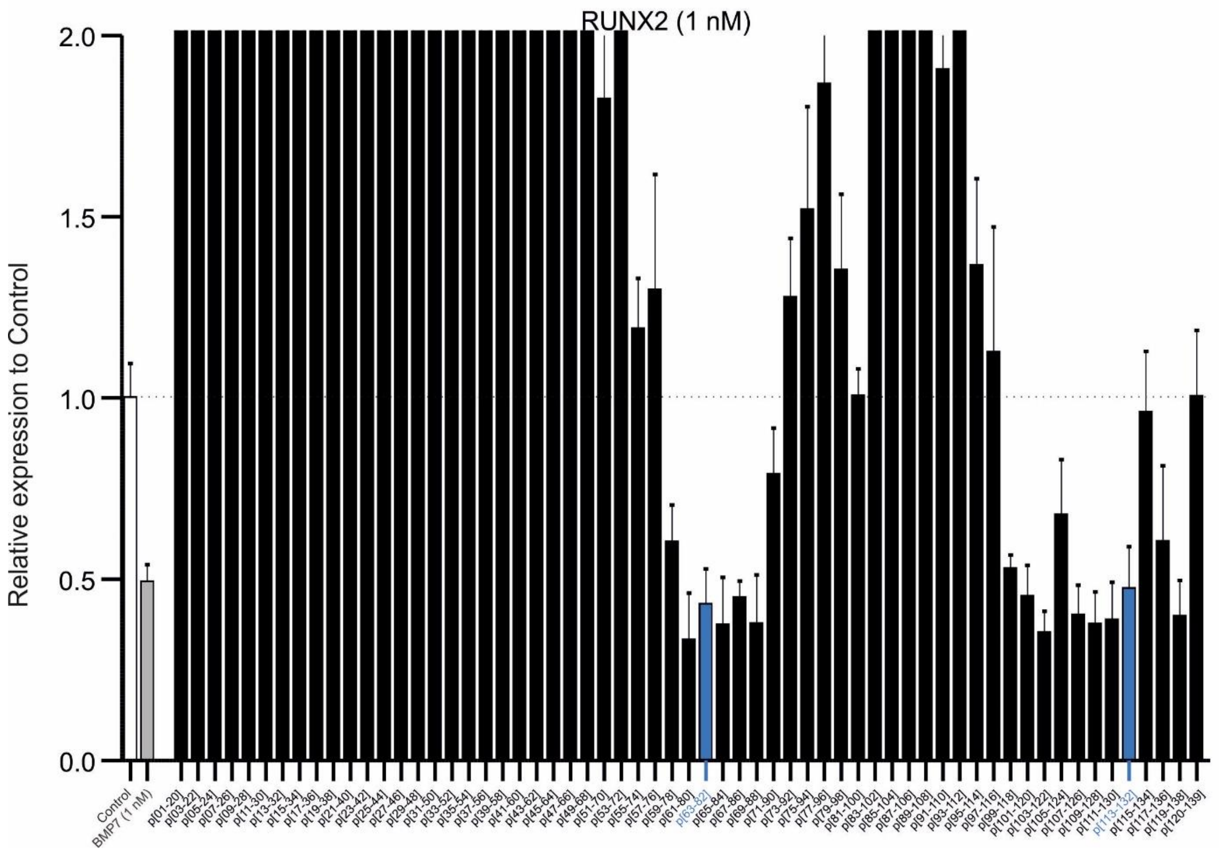
RUNX2 (1 nM)
Relative expression to Control
2.0
1.5
1.0
0.5
0.0
Control
BMP7 (1 nM)
p[01-20]
p[03-22]
p[05-24]
p[07-26]
p[09-28]
p[11-30]
p[13-32]
p[15-34]
p[17-36]
p[19-38]
p[21-40]
p[23-42]
p[25-44]
p[27-46]
p[29-48]
p[31-50]
p[33-52]
p[35-54]
p[37-56]
p[39-58]
p[41-60]
p[43-62]
p[45-64]
p[47-66]
p[49-68]
p[51-70]
p[53-72]
p[55-74]
p[57-76]
p[59-78]
p[61-80]
p[63-82]
p[65-84]
p[67-86]
p[69-88]
p[71-90]
p[73-92]
p[75-94]
p[77-96]
p[79-98]
p[81-100]
p[83-102]
p[85-104]
p[87-106]
p[89-108]
p[91-110]
p[93-112]
p[95-114]
p[97-116]
p[99-118]
p[101-120]
p[103-122]
p[105-124]
p[107-126]
p[109-128]
p[111-130]
p[113-132]
p[115-134]
p[117-136]
p[119-138]
p[120-139]

F

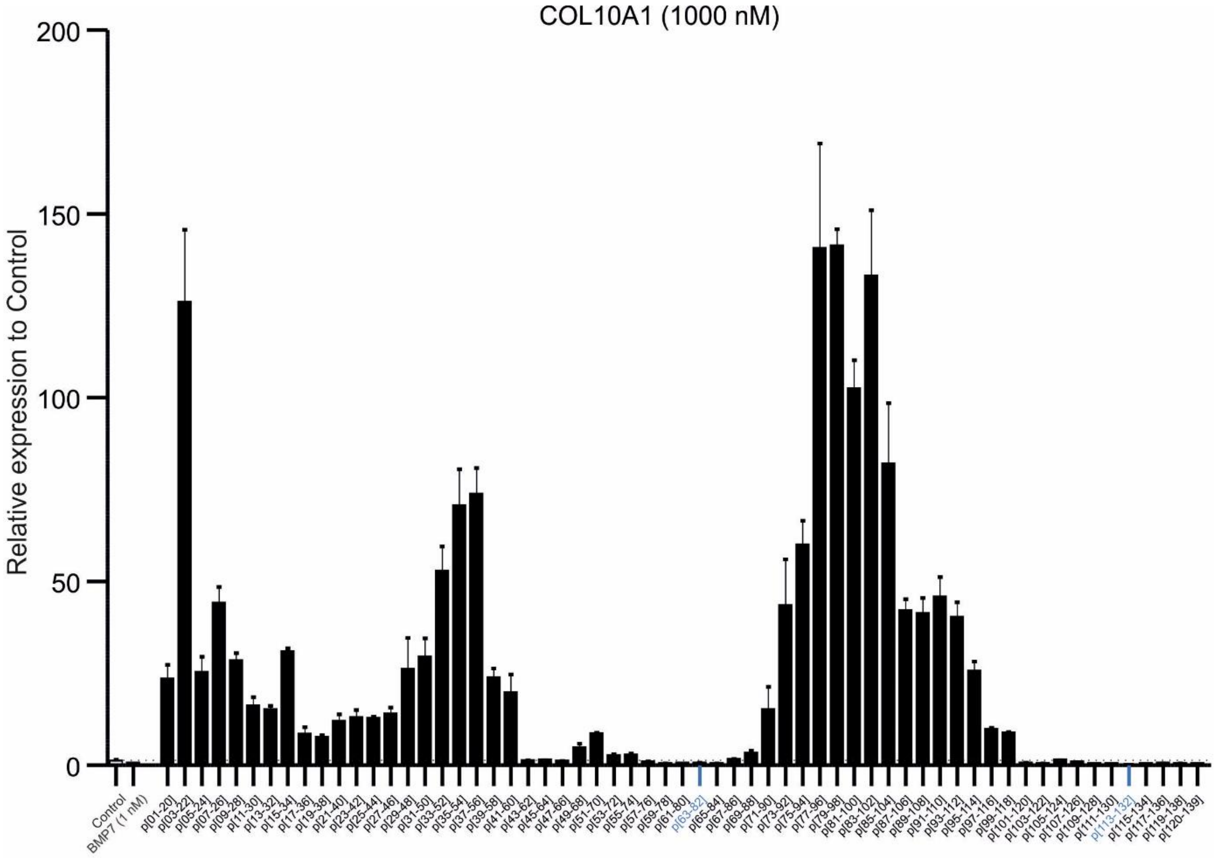
COL10A1 (1000 nM)
Relative expression to Control
200
150
100
50
0
Control
BMP7 (1 nM)

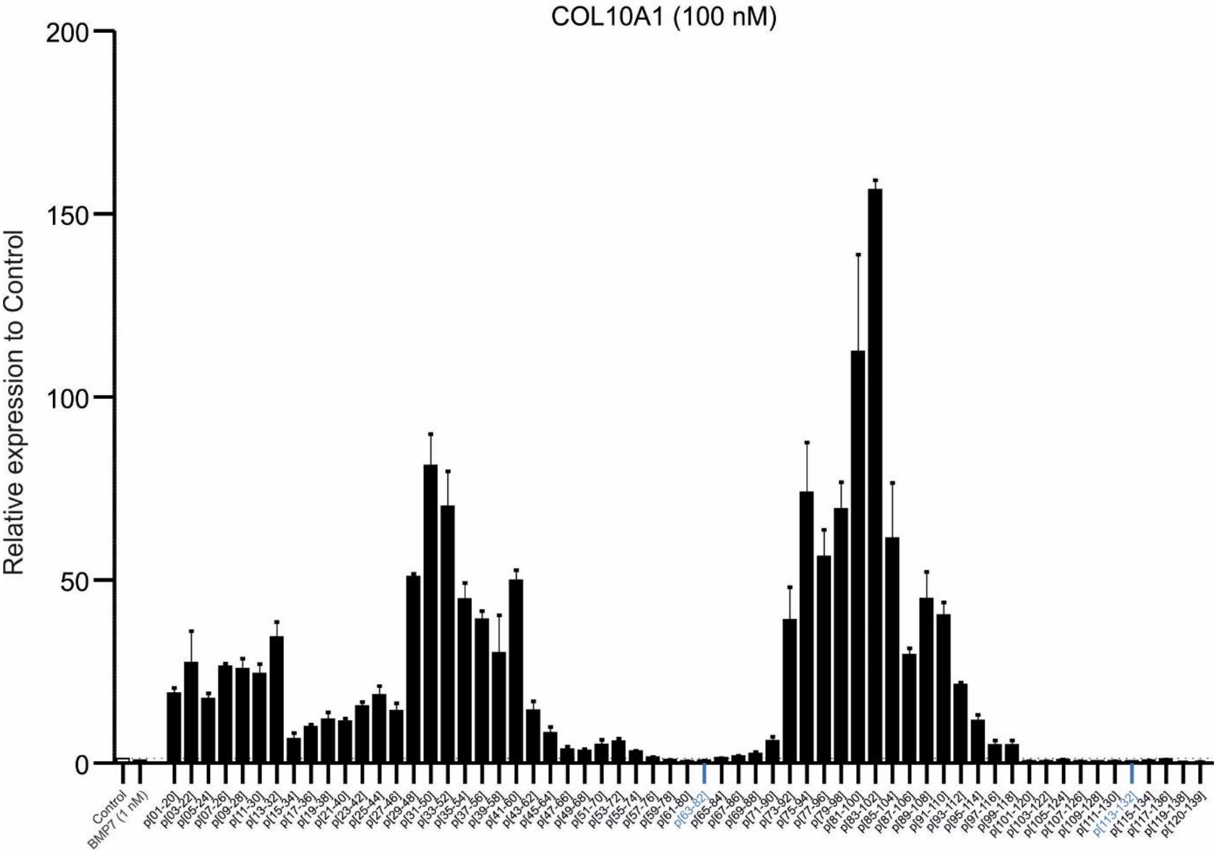
COL10A1 (100 nM)
Relative expression to Control
200
150
100
50
0
Control
BMP7 (1 nM)

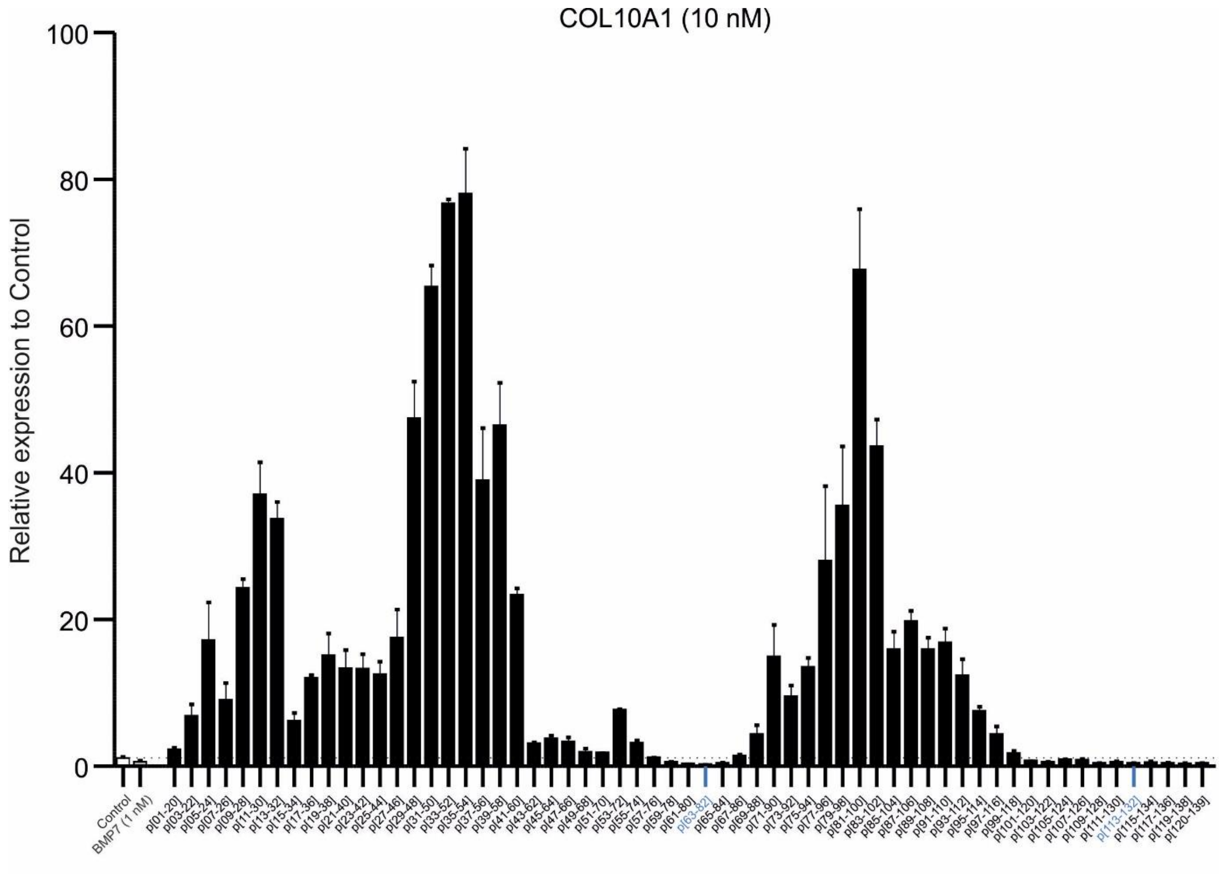
COL10A1 (10 nM)
Relative expression to Control
100
80
60
40
20
0
Control
BMP7 (1 nM)

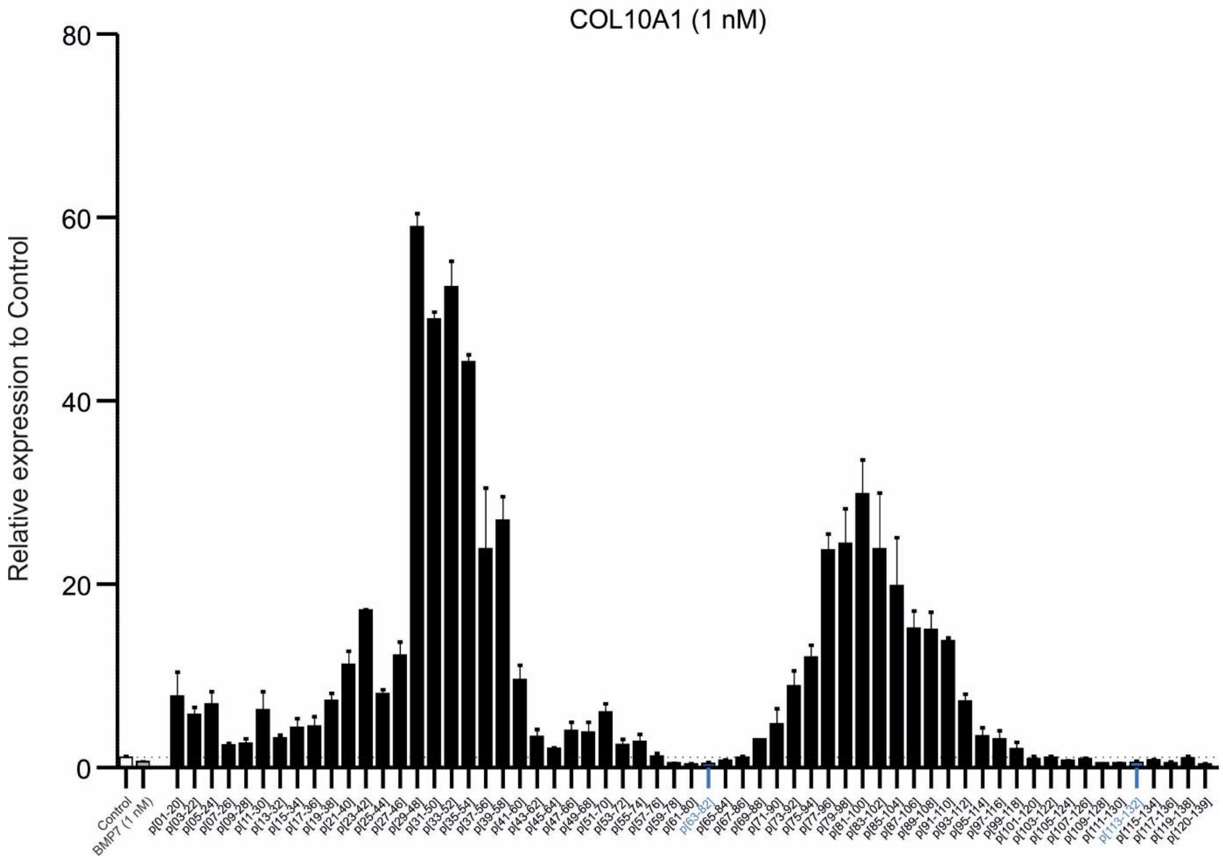
COL10A1 (1 nM)
Relative expression to Control
80
60
40
20
0
Control
BMP7 (1 nM)

G

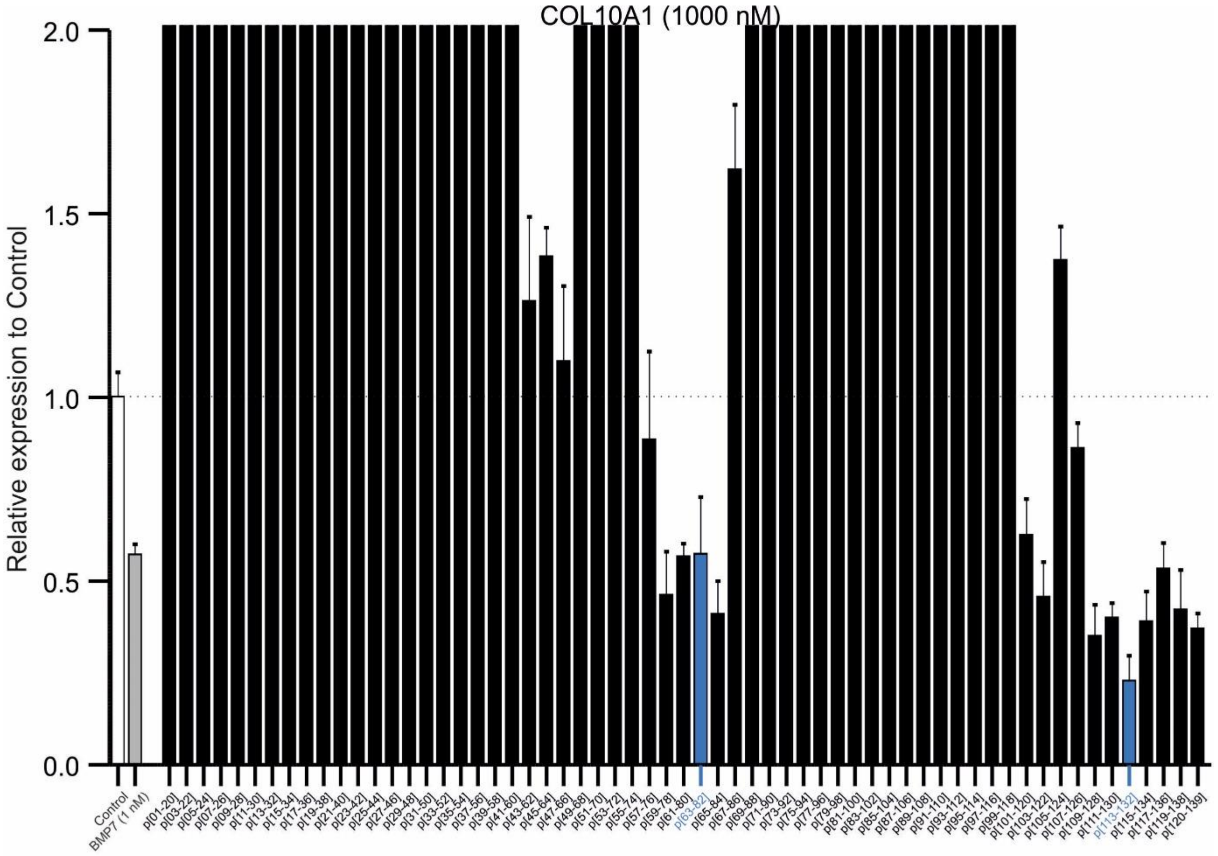

COL10A1 (1000 nM)
Relative expression to Control
2.0
1.5
1.0
0.5
0.0
Control
BMP7 (1 nM)

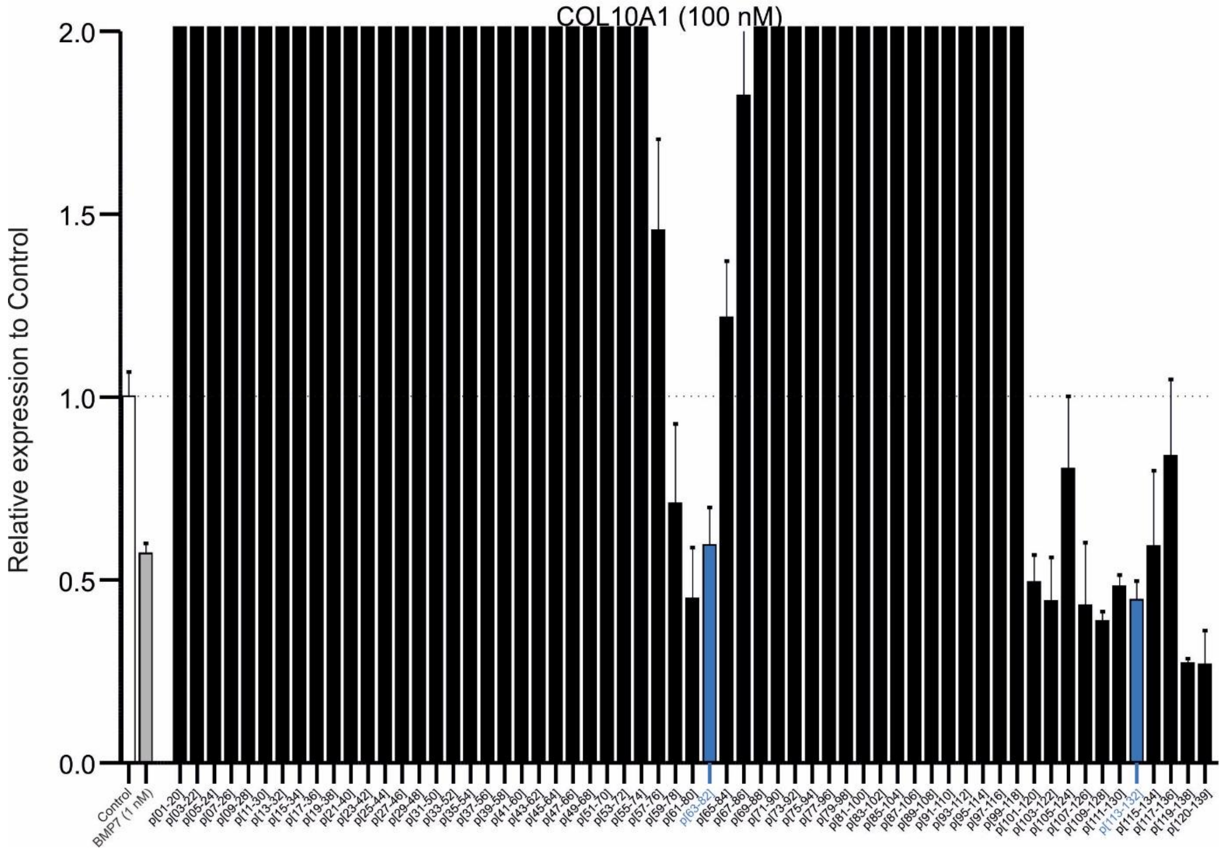

COL10A1 (100 nM)
Relative expression to Control
2.0
1.5
1.0
0.5
0.0
Control
BMP7 (1 nM)

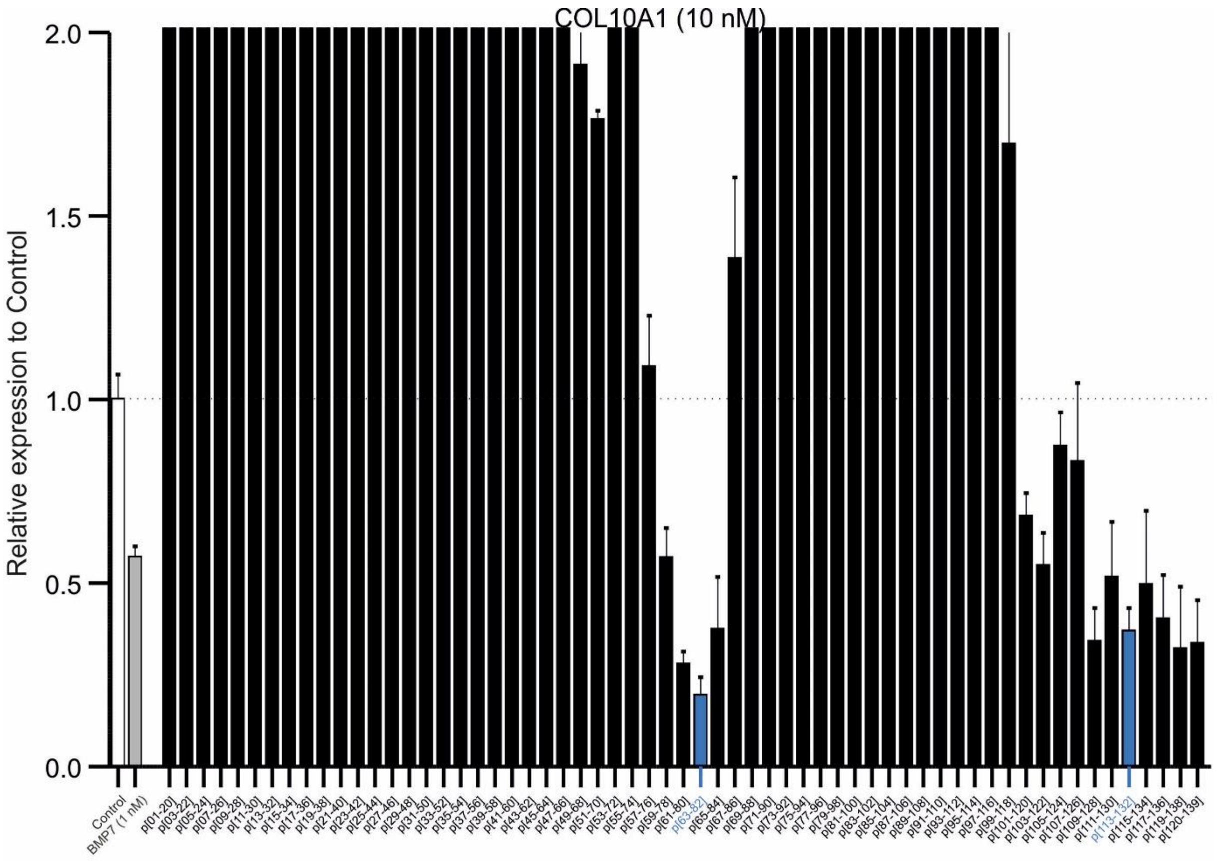
COL10A1 (10 nM)
Relative expression to Control
2.0
1.5
1.0
0.5
0.0
Control
BMP7 (1 nM)
p[01-20]
p[03-22]
p[05-24]
p[07-26]
p[09-28]
p[11-30]
p[13-32]
p[15-34]
p[17-36]
p[19-38]
p[21-40]
p[23-42]
p[25-44]
p[27-46]
p[29-48]
p[31-50]
p[33-52]
p[35-54]
p[37-56]
p[39-58]
p[41-60]
p[43-62]
p[45-64]
p[47-66]
p[49-68]
p[51-70]
p[53-72]
p[55-74]
p[57-76]
p[59-78]
p[61-80]
p[63-82]
p[65-84]
p[67-86]
p[69-88]
p[71-90]
p[73-92]
p[75-94]
p[77-96]
p[79-98]
p[81-100]
p[83-102]
p[85-104]
p[87-106]
p[89-108]
p[91-110]
p[93-112]
p[95-114]
p[97-116]
p[99-118]
p[101-120]
p[103-122]
p[105-124]
p[107-126]
p[109-128]
p[111-130]
p[113-132]
p[115-134]
p[117-136]
p[119-138]
p[120-139]

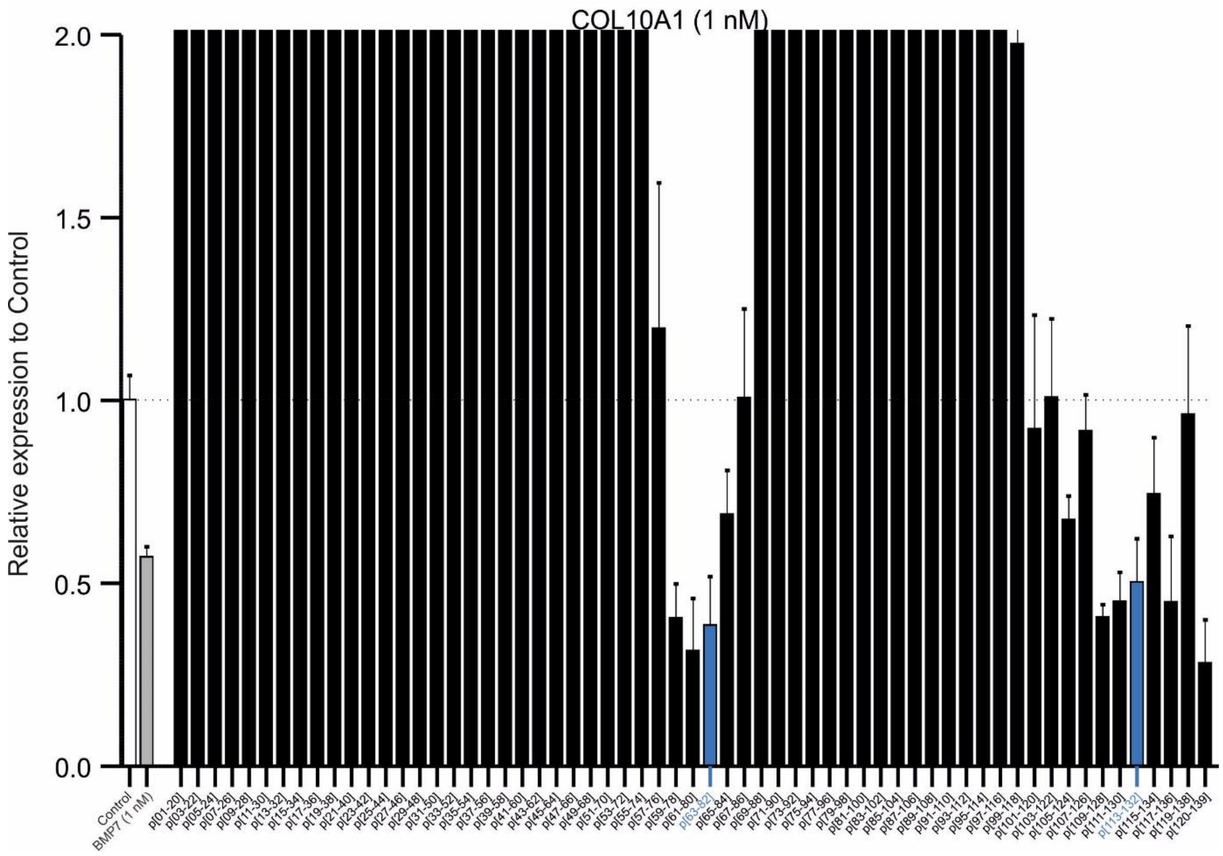
COL10A1 (1 nM)
Relative expression to Control
2.0
1.5
1.0
0.5
0.0
Control
BMP7 (1 nM)
p[01-20]
p[03-22]
p[05-24]
p[07-26]
p[09-28]
p[11-30]
p[13-32]
p[15-34]
p[17-36]
p[19-38]
p[21-40]
p[23-42]
p[25-44]
p[27-46]
p[29-48]
p[31-50]
p[33-52]
p[35-54]
p[37-56]
p[39-58]
p[41-60]
p[43-62]
p[45-64]
p[47-66]
p[49-68]
p[51-70]
p[53-72]
p[55-74]
p[57-76]
p[59-78]
p[61-80]
p[63-82]
p[65-84]
p[67-86]
p[69-88]
p[71-90]
p[73-92]
p[75-94]
p[77-96]
p[79-98]
p[81-100]
p[83-102]
p[85-104]
p[87-106]
p[89-108]
p[91-110]
p[93-112]
p[95-114]
p[97-116]
p[99-118]
p[101-120]
p[103-122]
p[105-124]
p[107-126]
p[109-128]
p[111-130]
p[113-132]
p[115-134]
p[117-136]
p[119-138]
p[120-139]

H

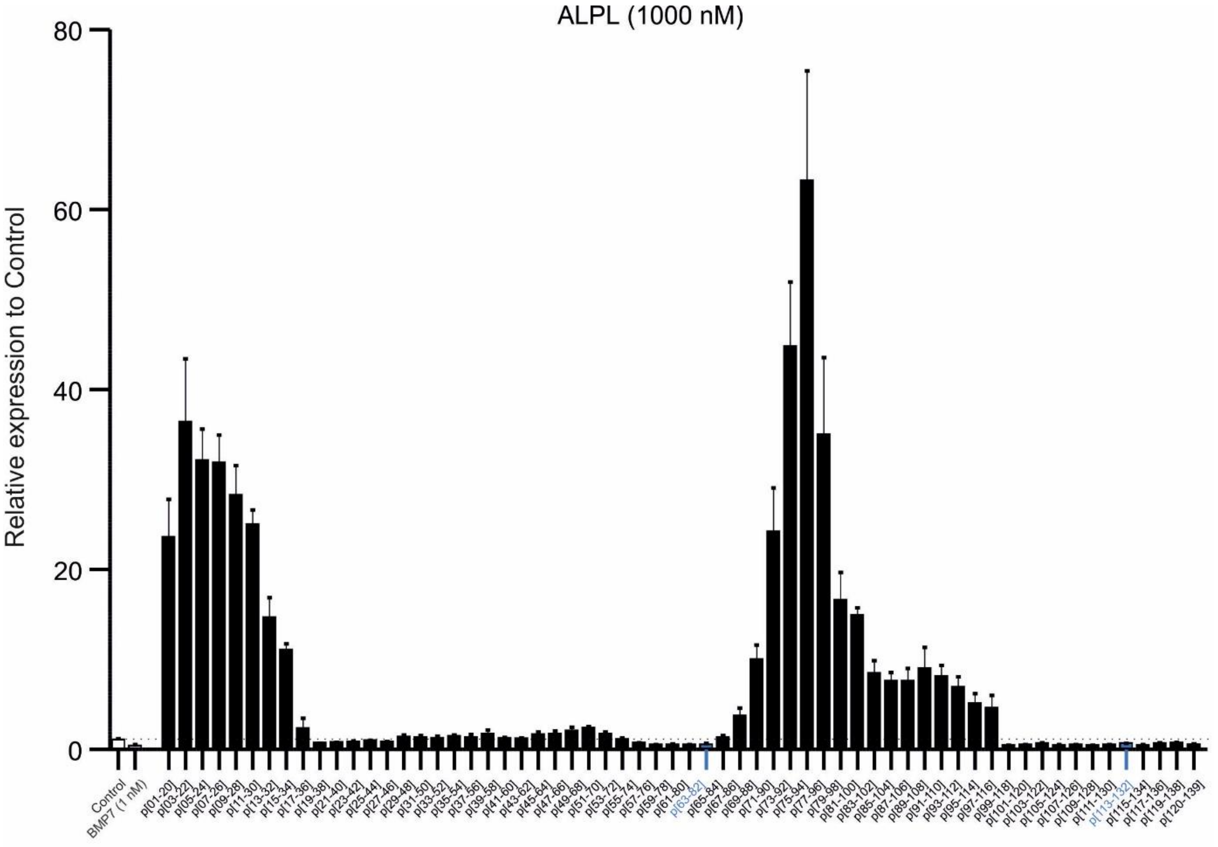
ALPL (1000 nM)
Relative expression to Control
0
20
40
60
80
Control
BMP7 (1 nM)

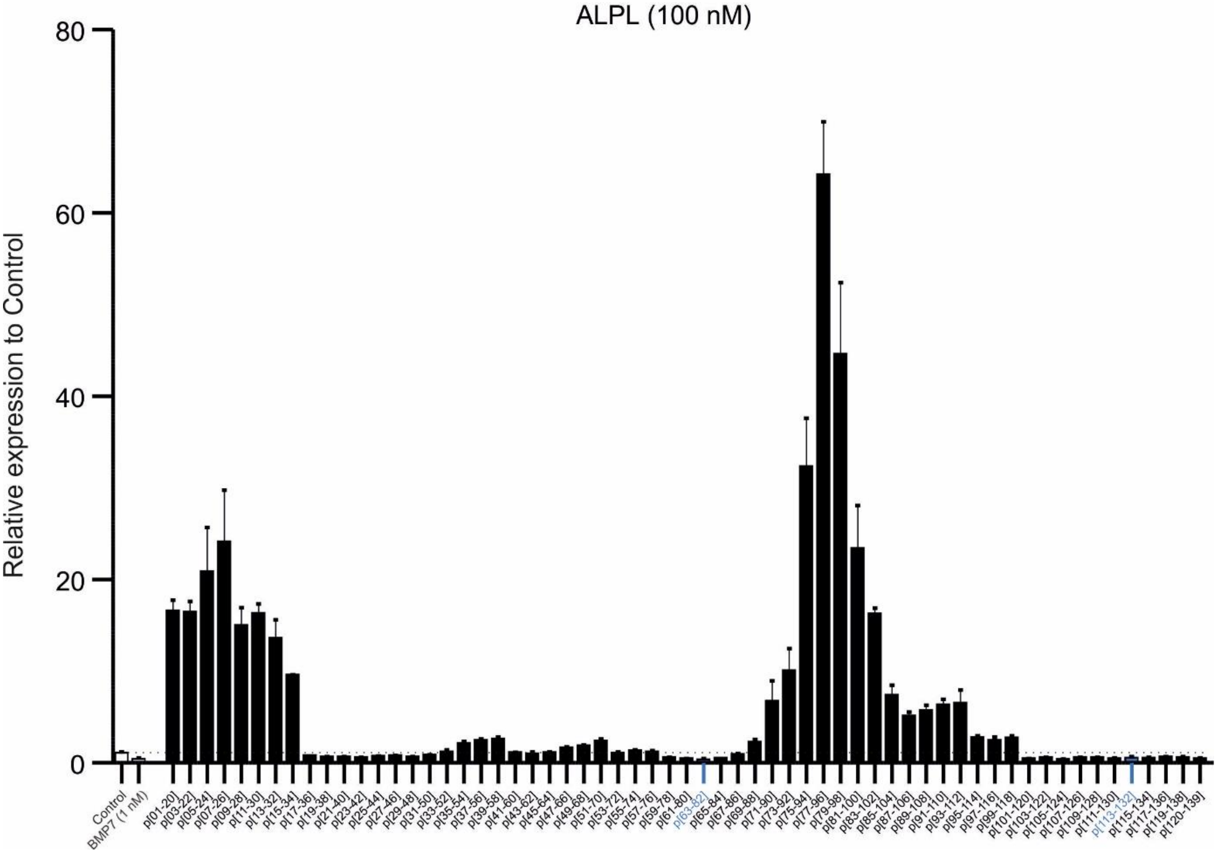
ALPL (100 nM)
Relative expression to Control
0
20
40
60
80
Control
BMP7 (1 nM)

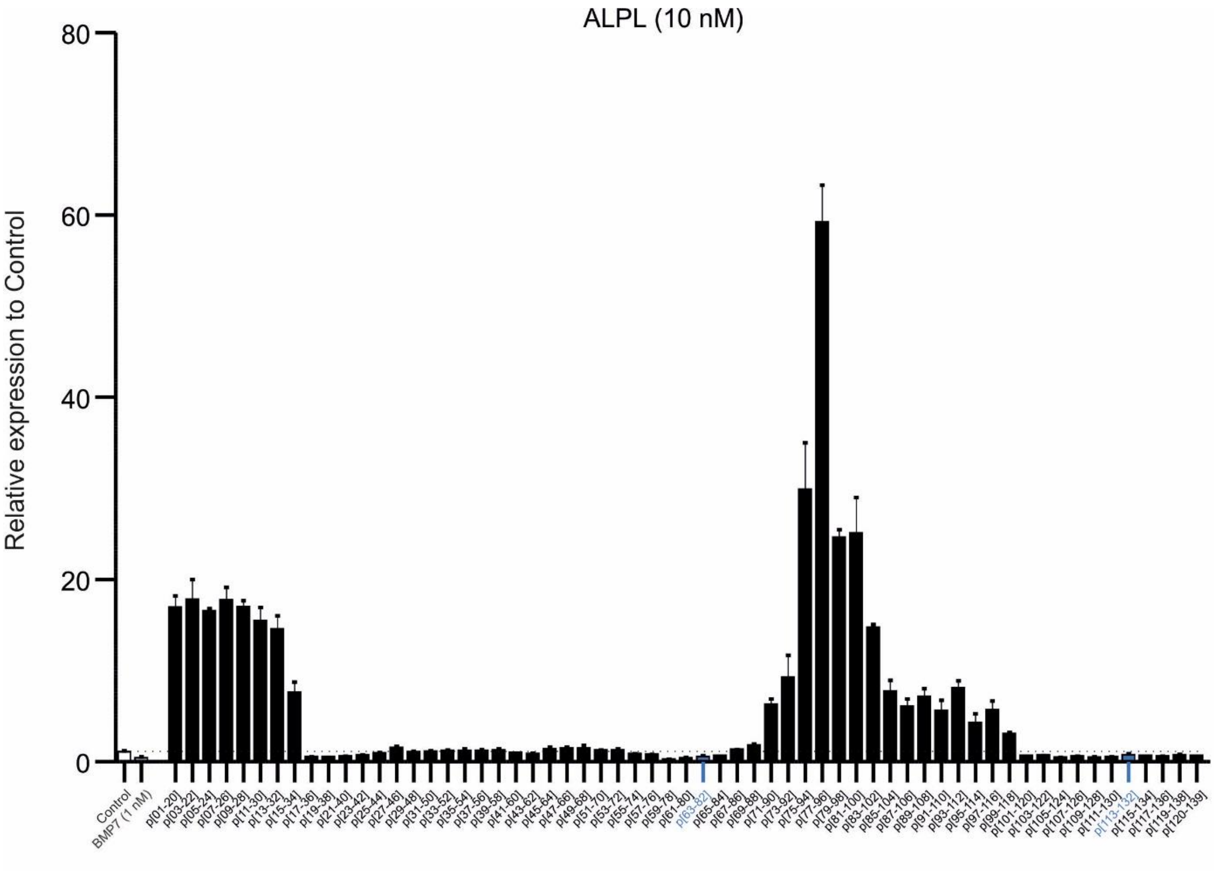
ALPL (10 nM)
Relative expression to Control
80
60
40
20
0
Control
BMP7 (1 nM)

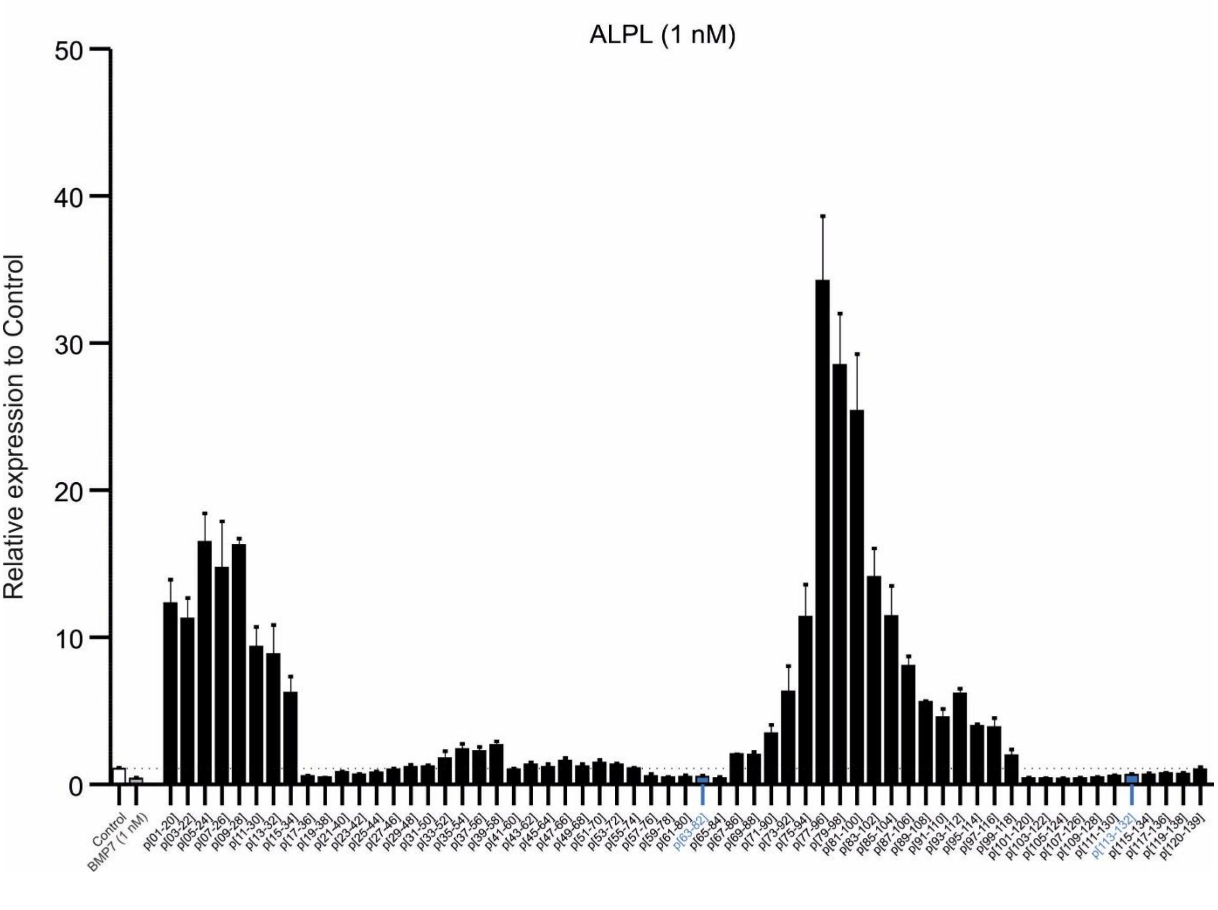
ALPL (1 nM)
Relative expression to Control
50
40
30
20
10
0
Control
BMP7 (1 nM)

I

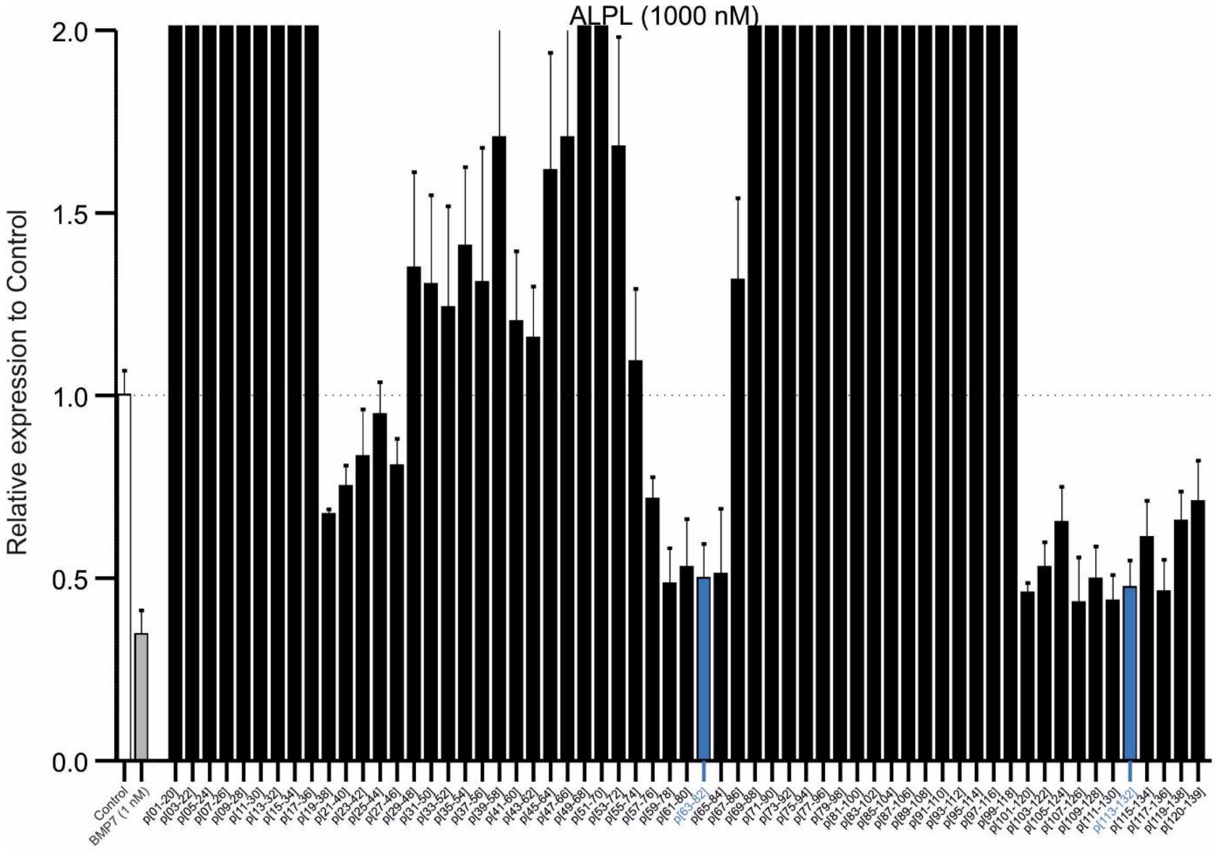
ALPL (1000 nM)
Relative expression to Control
2.0
1.5
1.0
0.5
0.0

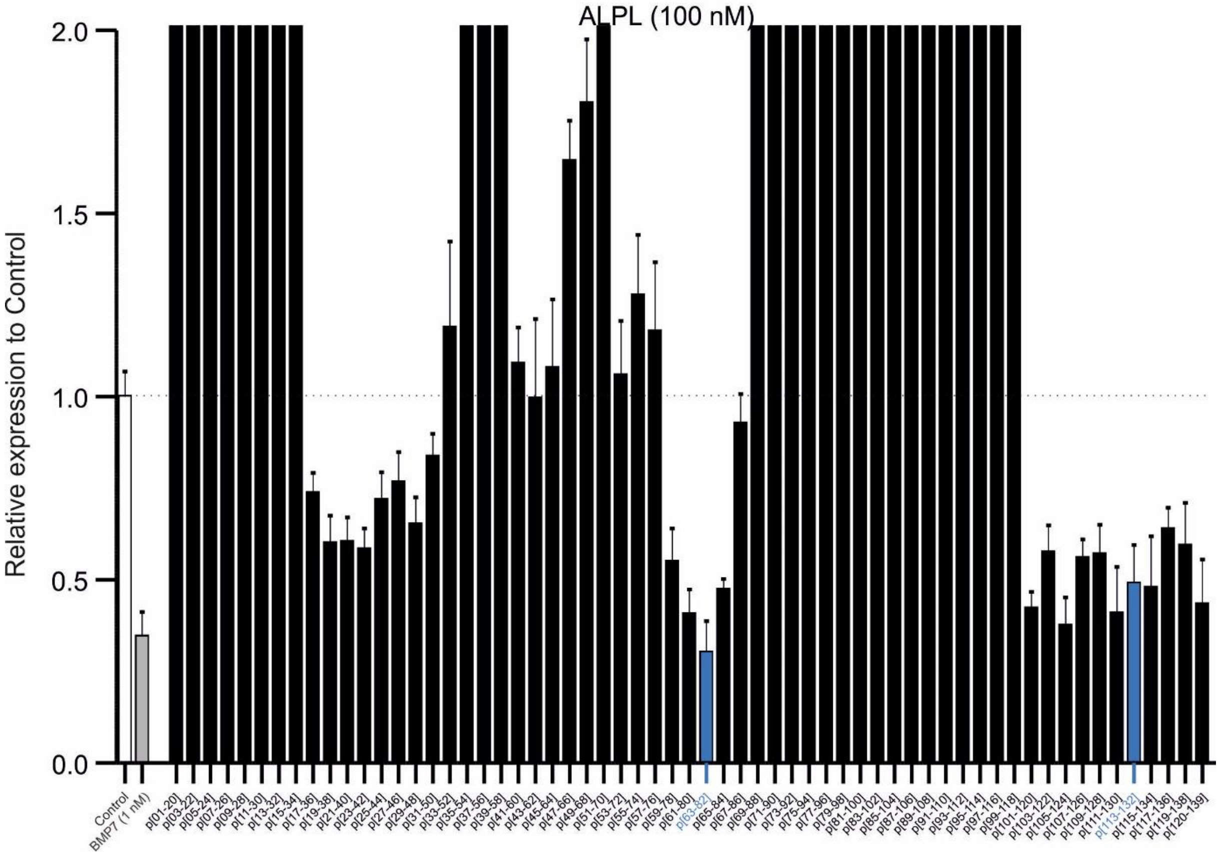
ALPL (100 nM)
Relative expression to Control
2.0
1.5
1.0
0.5
0.0

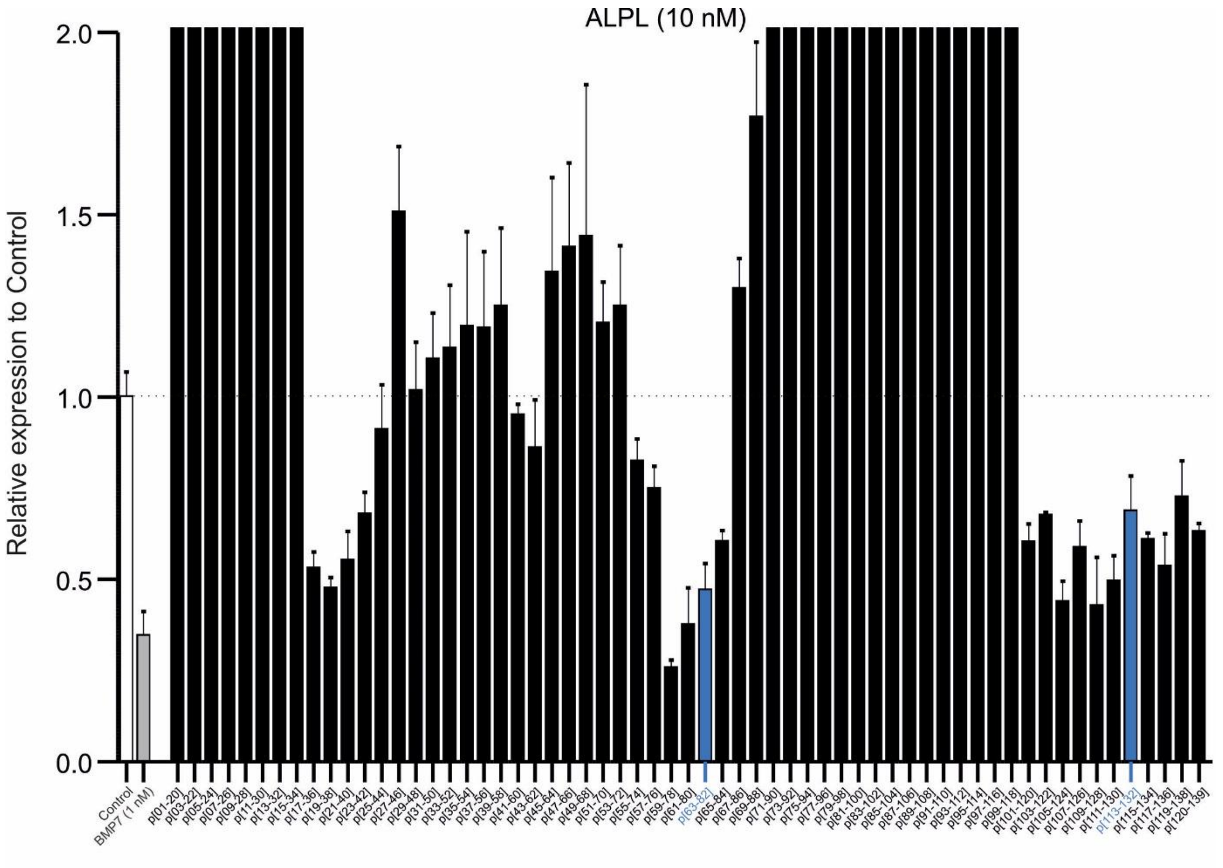
ALPL (10 nM)
Relative expression to Control
2.0
1.5
1.0
0.5
0.0
Control
BMP7 (1 nM)
p[01-20] p[03-22] p[05-24] p[07-26] p[09-28] p[11-30] p[13-32] p[15-34] p[17-36] p[19-38] p[21-40] p[23-42] p[25-44] p[27-46] p[29-48] p[31-50] p[33-52] p[35-54] p[37-56] p[39-58] p[41-60] p[43-62] p[45-64] p[47-66] p[49-68] p[51-70] p[53-72] p[55-74] p[57-76] p[59-78] p[61-80] p[63-82] p[65-84] p[67-86] p[69-88] p[71-90] p[73-92] p[75-94] p[77-96] p[79-98] p[81-100] p[83-102] p[85-104] p[87-106] p[89-108] p[91-110] p[93-112] p[95-114] p[97-116] p[99-118] p[101-120] p[103-122] p[105-124] p[107-126] p[109-128] p[111-130] p[113-132] p[115-134] p[117-136] p[119-138] p[120-139]

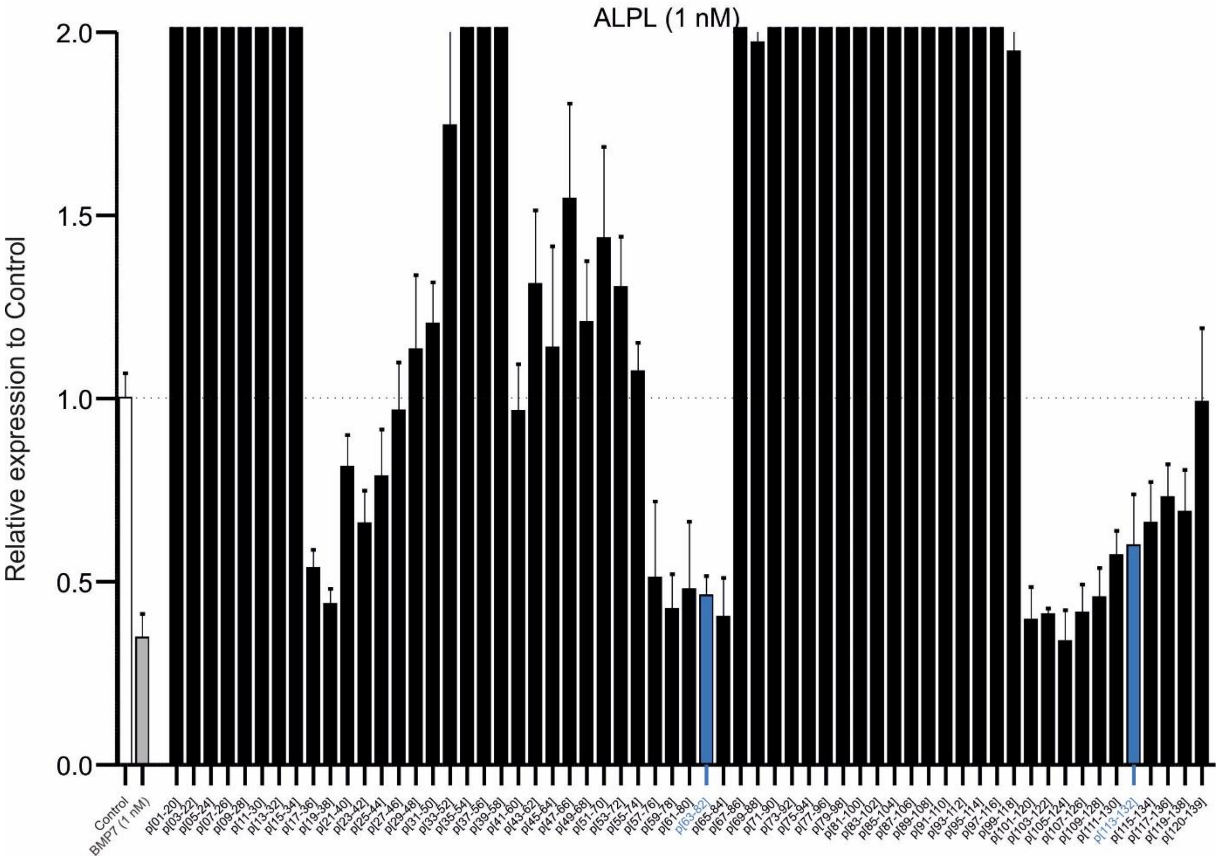
ALPL (1 nM)
Relative expression to Control
2.0
1.5
1.0
0.5
0.0
Control
BMP7 (1 nM)
p[01-20] p[03-22] p[05-24] p[07-26] p[09-28] p[11-30] p[13-32] p[15-34] p[17-36] p[19-38] p[21-40] p[23-42] p[25-44] p[27-46] p[29-48] p[31-50] p[33-52] p[35-54] p[37-56] p[39-58] p[41-60] p[43-62] p[45-64] p[47-66] p[49-68] p[51-70] p[53-72] p[55-74] p[57-76] p[59-78] p[61-80] p[63-82] p[65-84] p[67-86] p[69-88] p[71-90] p[73-92] p[75-94] p[77-96] p[79-98] p[81-100] p[83-102] p[85-104] p[87-106] p[89-108] p[91-110] p[93-112] p[95-114] p[97-116] p[99-118] p[101-120] p[103-122] p[105-124] p[107-126] p[109-128] p[111-130] p[113-132] p[115-134] p[117-136] p[119-138] p[120-139]

J

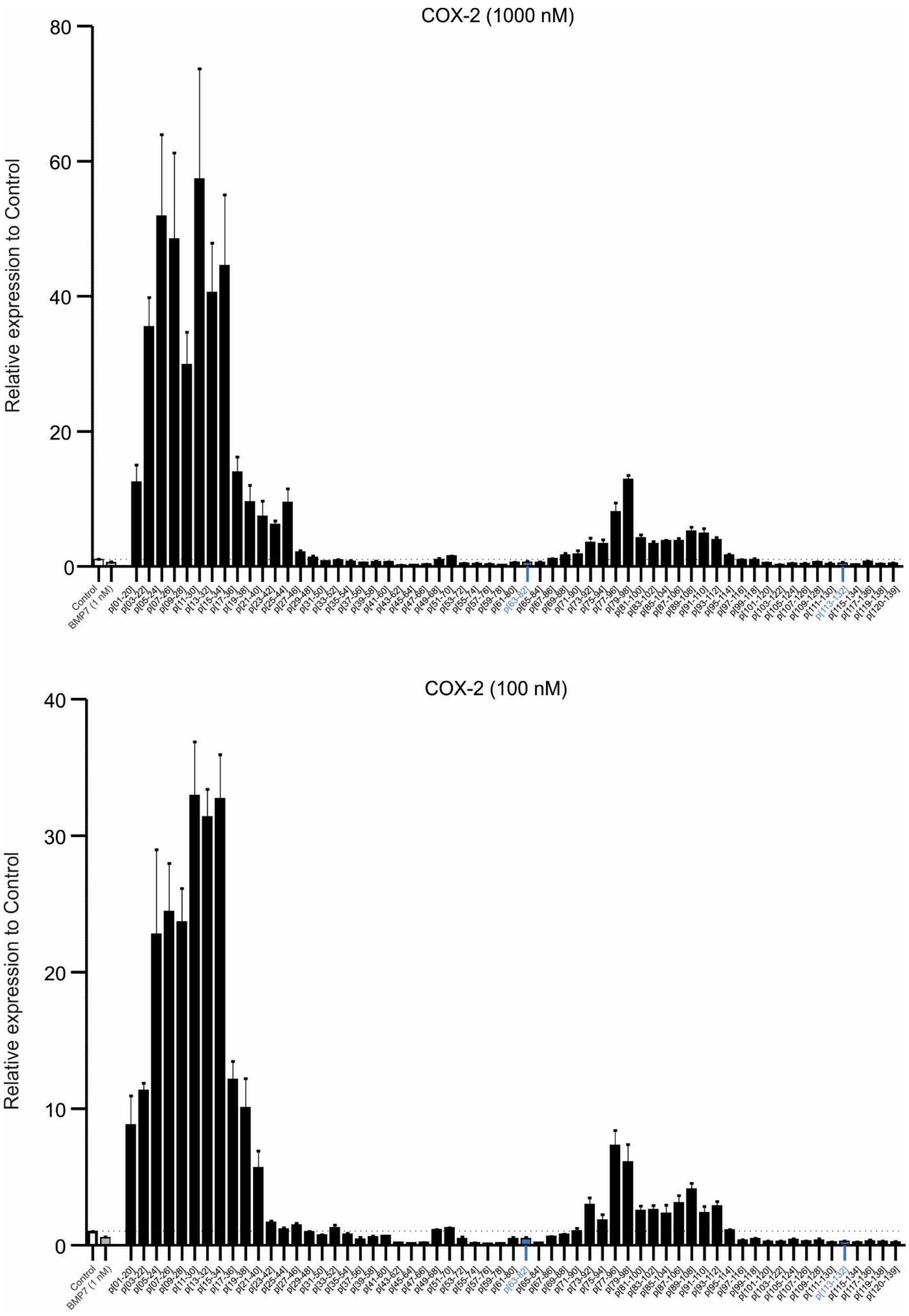
COX-2 (1000 nM)
Relative expression to Control
80
60
40
20
0
Control
BMP7 (1 nM)
COX-2 (100 nM)
Relative expression to Control
40
30
20
10
0
Control
BMP7 (1 nM)

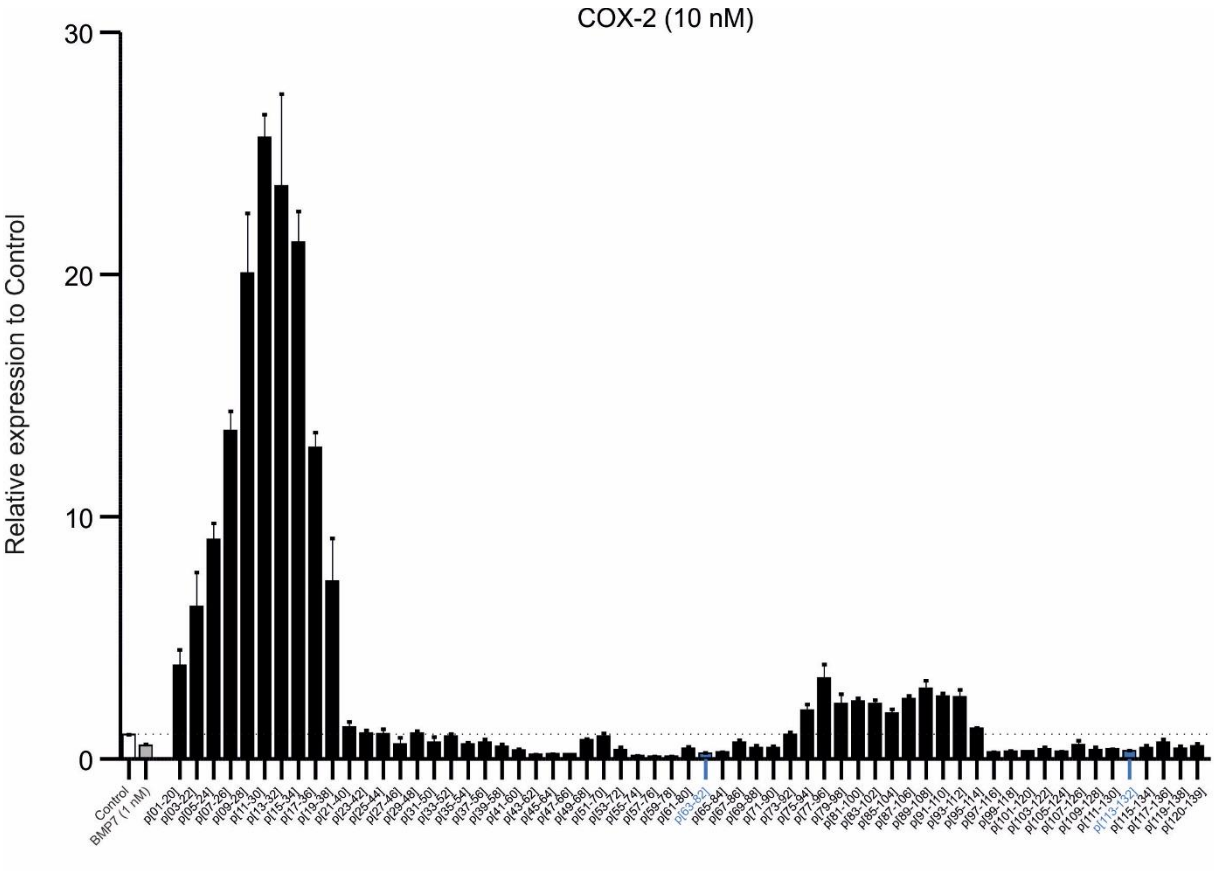
COX-2 (10 nM)
Relative expression to Control
30
20
10
0
Control
BMP7 (1 nM)
p[01-20] p[03-22] p[05-24] p[07-26] p[09-28] p[11-30] p[13-32] p[15-34] p[17-36] p[19-38] p[21-40] p[23-42] p[25-44] p[27-46] p[29-48] p[31-50] p[33-52] p[35-54] p[37-56] p[39-58] p[41-60] p[43-62] p[45-64] p[47-66] p[49-68] p[51-70] p[53-72] p[55-74] p[57-76] p[59-78] p[61-80] p[63-82] p[65-84] p[67-86] p[69-88] p[71-90] p[73-92] p[75-94] p[77-96] p[79-98] p[81-100] p[83-102] p[85-104] p[87-106] p[89-108] p[91-110] p[93-112] p[95-114] p[97-116] p[99-118] p[101-120] p[103-122] p[105-124] p[107-126] p[109-128] p[111-130] p[113-132] p[115-134] p[117-136] p[119-138] p[120-139]

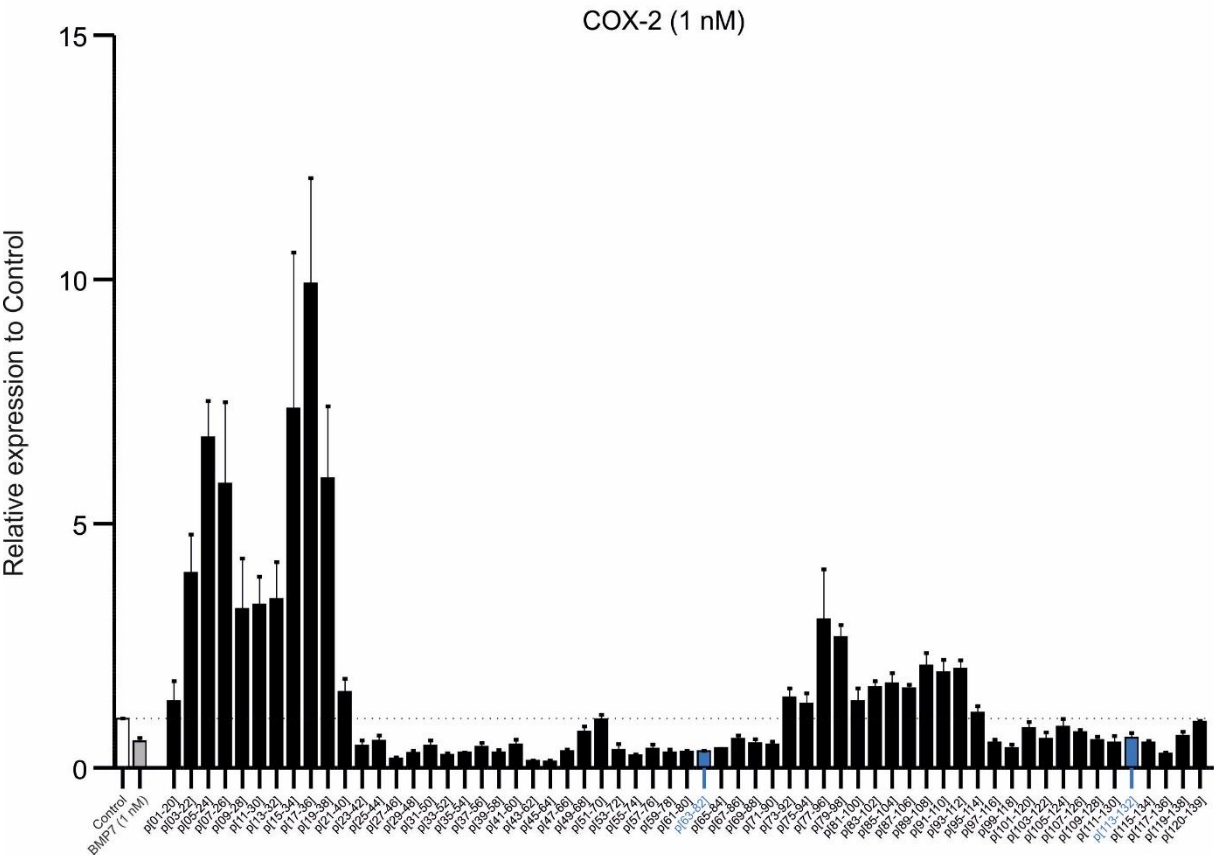
COX-2 (1 nM)
Relative expression to Control
15
10
5
0
Control
BMP7 (1 nM)
p[01-20] p[03-22] p[05-24] p[07-26] p[09-28] p[11-30] p[13-32] p[15-34] p[17-36] p[19-38] p[21-40] p[23-42] p[25-44] p[27-46] p[29-48] p[31-50] p[33-52] p[35-54] p[37-56] p[39-58] p[41-60] p[43-62] p[45-64] p[47-66] p[49-68] p[51-70] p[53-72] p[55-74] p[57-76] p[59-78] p[61-80] p[63-82] p[65-84] p[67-86] p[69-88] p[71-90] p[73-92] p[75-94] p[77-96] p[79-98] p[81-100] p[83-102] p[85-104] p[87-106] p[89-108] p[91-110] p[93-112] p[95-114] p[97-116] p[99-118] p[101-120] p[103-122] p[105-124] p[107-126] p[109-128] p[111-130] p[113-132] p[115-134] p[117-136] p[119-138] p[120-139]

K

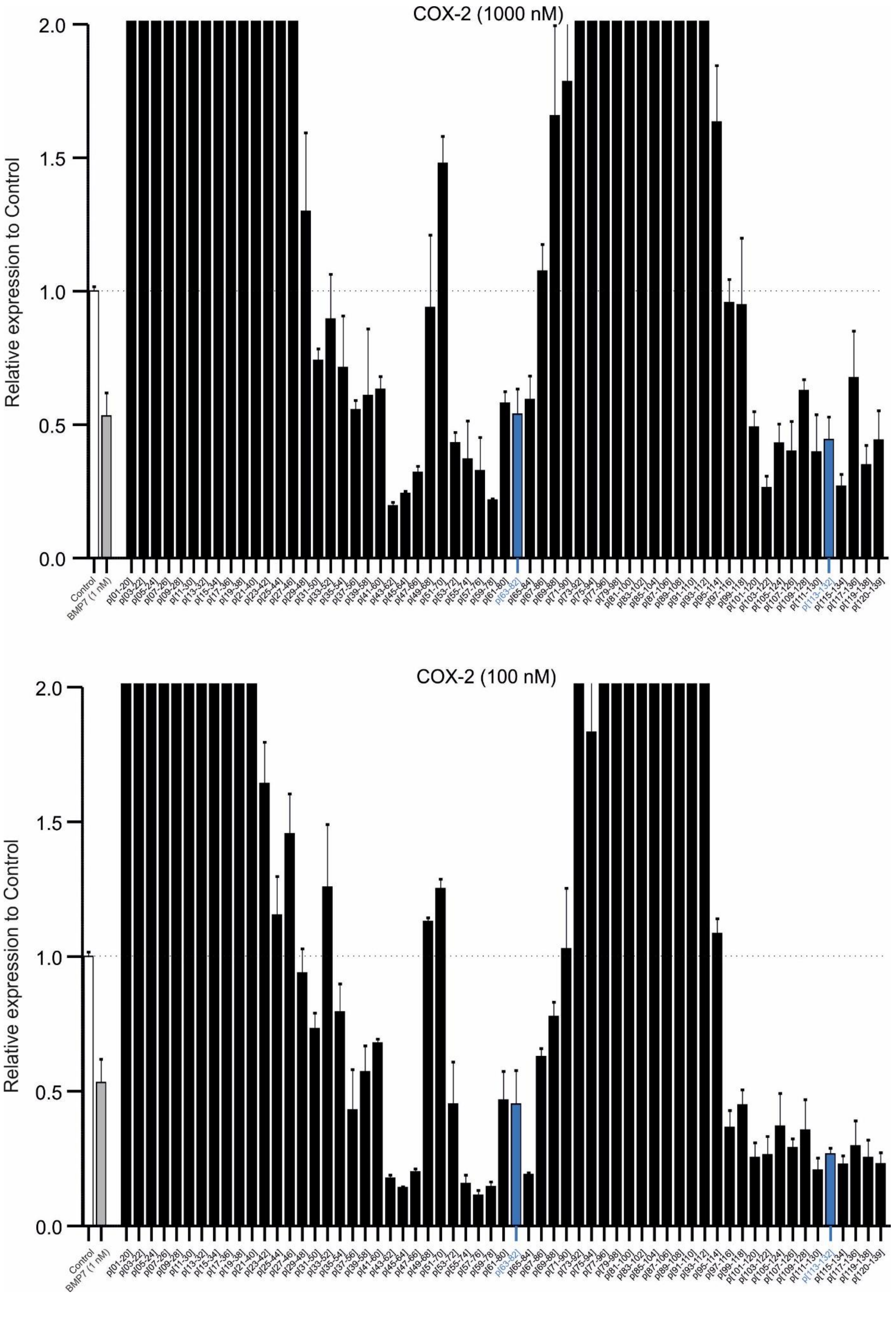
COX-2 (1000 nM)
Relative expression to Control
0.0
0.5
1.0
1.5
2.0
Control
BMP7 (1 nM)
p[01-20]
p[03-22]
p[05-24]
p[07-26]
p[09-28]
p[11-30]
p[13-32]
p[15-34]
p[17-36]
p[19-38]
p[21-40]
p[23-42]
p[25-44]
p[27-46]
p[29-48]
p[31-50]
p[33-52]
p[35-54]
p[37-56]
p[39-58]
p[41-60]
p[43-62]
p[45-64]
p[47-66]
p[49-68]
p[51-70]
p[53-72]
p[55-74]
p[57-76]
p[59-78]
p[61-80]
p[63-82]
p[65-84]
p[67-86]
p[69-88]
p[71-90]
p[73-92]
p[75-94]
p[77-96]
p[79-98]
p[81-100]
p[83-102]
p[85-104]
p[87-106]
p[89-108]
p[91-110]
p[93-112]
p[95-114]
p[97-116]
p[99-118]
p[101-120]
p[103-122]
p[105-124]
p[107-126]
p[109-128]
p[111-130]
p[113-132]
p[115-134]
p[117-136]
p[119-138]
p[120-139]
COX-2 (100 nM)
Relative expression to Control
0.0
0.5
1.0
1.5
2.0
Control
BMP7 (1 nM)
p[01-20]
p[03-22]
p[05-24]
p[07-26]
p[09-28]
p[11-30]
p[13-32]
p[15-34]
p[17-36]
p[19-38]
p[21-40]
p[23-42]
p[25-44]
p[27-46]
p[29-48]
p[31-50]
p[33-52]
p[35-54]
p[37-56]
p[39-58]
p[41-60]
p[43-62]
p[45-64]
p[47-66]
p[49-68]
p[51-70]
p[53-72]
p[55-74]
p[57-76]
p[59-78]
p[61-80]
p[63-82]
p[65-84]
p[67-86]
p[69-88]
p[71-90]
p[73-92]
p[75-94]
p[77-96]
p[79-98]
p[81-100]
p[83-102]
p[85-104]
p[87-106]
p[89-108]
p[91-110]
p[93-112]
p[95-114]
p[97-116]
p[99-118]
p[101-120]
p[103-122]
p[105-124]
p[107-126]
p[109-128]
p[111-130]
p[113-132]
p[115-134]
p[117-136]
p[119-138]
p[120-139]

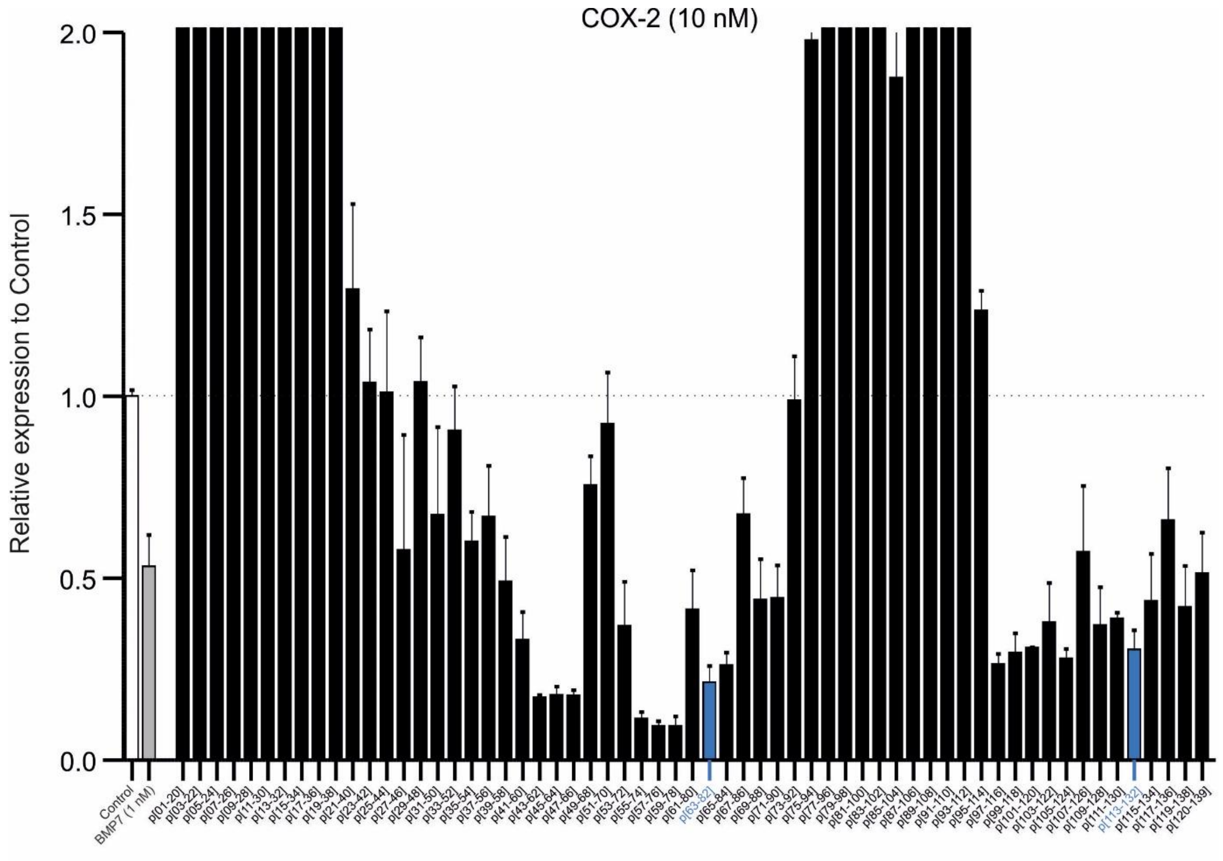
COX-2 (10 nM)
Relative expression to Control
2.0
1.5
1.0
0.5
0.0
Control
BMP7 (1 nM)

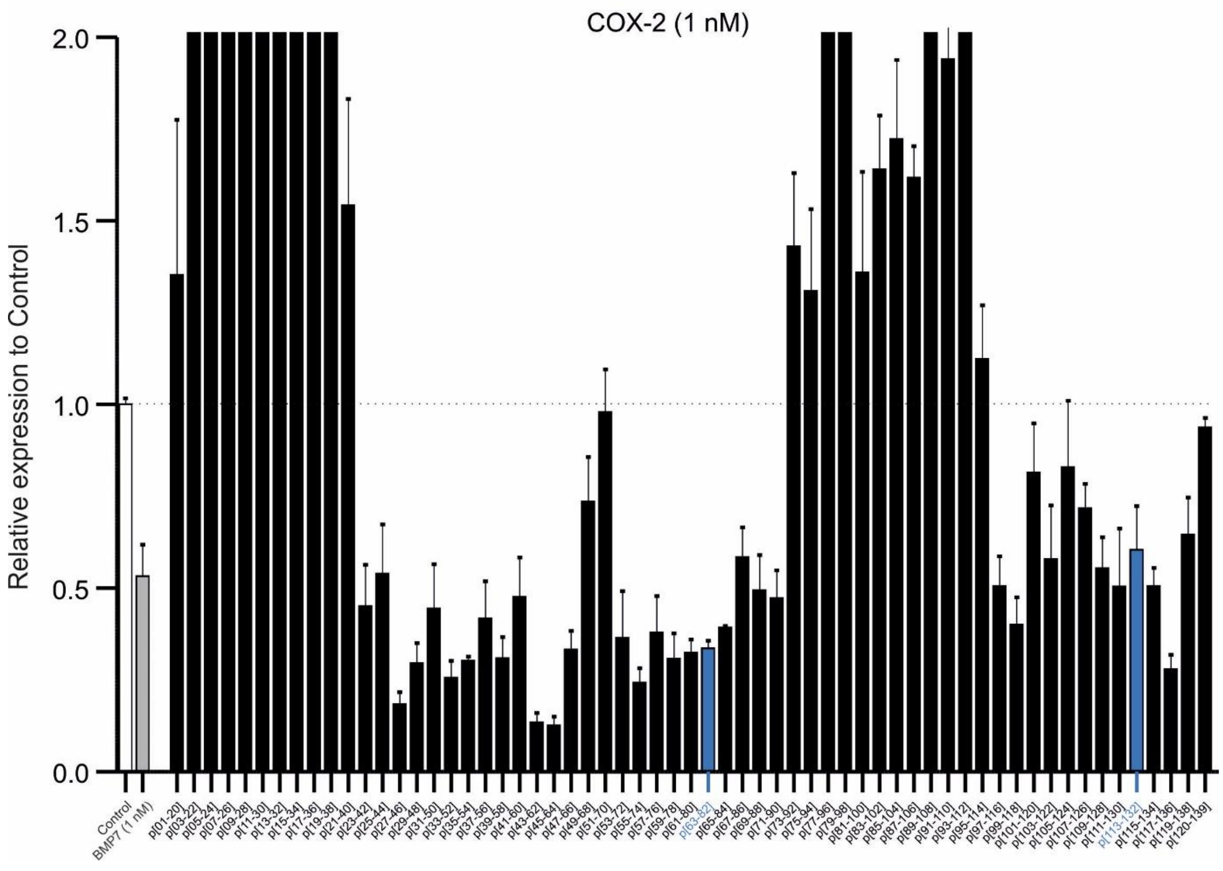
COX-2 (1 nM)
Relative expression to Control
2.0
1.5
1.0
0.5
0.0
Control
BMP7 (1 nM)

L

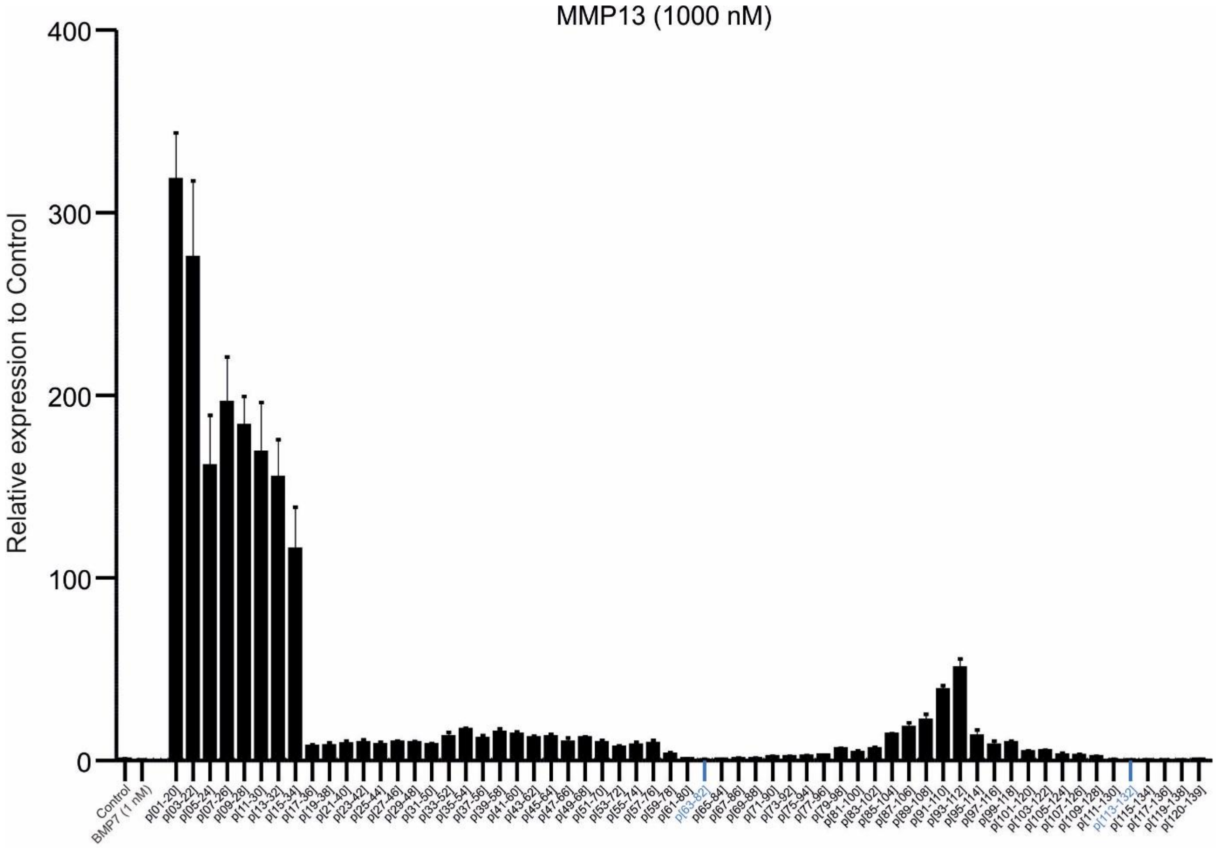
MMP13 (1000 nM)
Relative expression to Control
400
300
200
100
0
Control
BMP7 (1 nM)

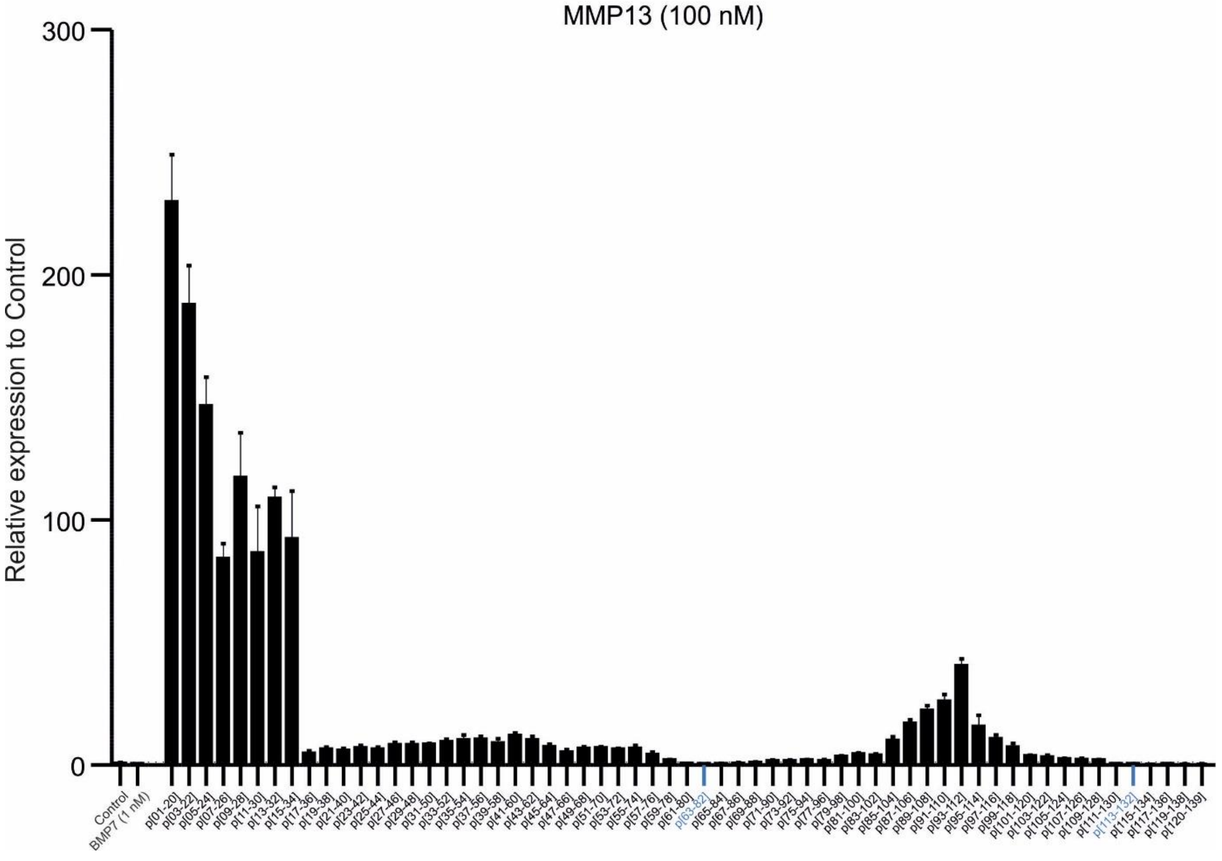
MMP13 (100 nM)
Relative expression to Control
300
200
100
0
Control
BMP7 (1 nM)

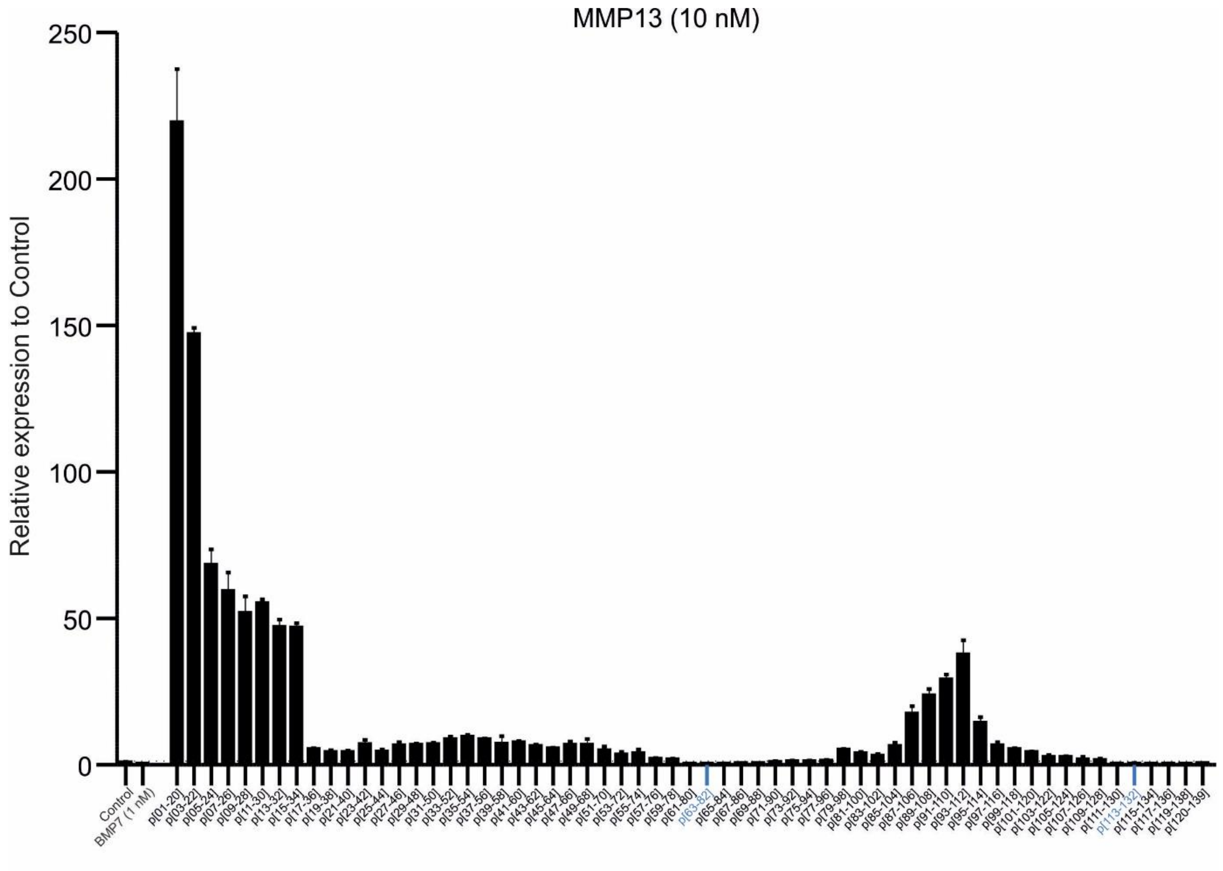

MMP13 (10 nM)
Relative expression to Control
250
200
150
100
50
0
Control
BMP7 (1 nM)
p[01-20]
p[03-22]
p[05-24]
p[07-26]
p[09-28]
p[11-30]
p[13-32]
p[15-34]
p[17-36]
p[19-38]
p[21-40]
p[23-42]
p[25-44]
p[27-46]
p[29-48]
p[31-50]
p[33-52]
p[35-54]
p[37-56]
p[39-58]
p[41-60]
p[43-62]
p[45-64]
p[47-66]
p[49-68]
p[51-70]
p[53-72]
p[55-74]
p[57-76]
p[59-78]
p[61-80]
p[63-82]
p[65-84]
p[67-86]
p[69-88]
p[71-90]
p[73-92]
p[75-94]
p[77-96]
p[79-98]
p[81-100]
p[83-102]
p[85-104]
p[87-106]
p[89-108]
p[91-110]
p[93-112]
p[95-114]
p[97-116]
p[99-118]
p[101-120]
p[103-122]
p[105-124]
p[107-126]
p[109-128]
p[111-130]
p[113-132]
p[115-134]
p[117-136]
p[119-138]
p[120-139]

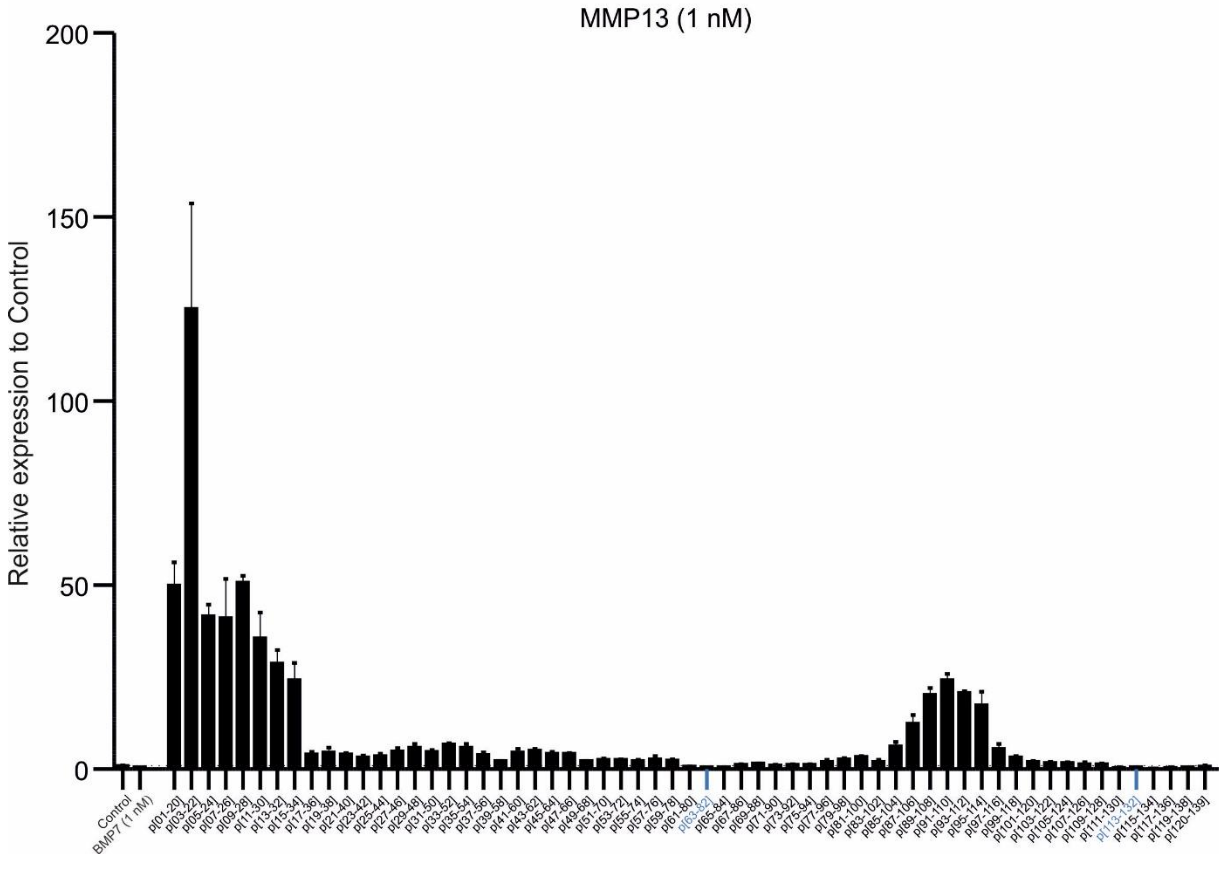

MMP13 (1 nM)
Relative expression to Control
200
150
100
50
0
Control
BMP7 (1 nM)
p[01-20]
p[03-22]
p[05-24]
p[07-26]
p[09-28]
p[11-30]
p[13-32]
p[15-34]
p[17-36]
p[19-38]
p[21-40]
p[23-42]
p[25-44]
p[27-46]
p[29-48]
p[31-50]
p[33-52]
p[35-54]
p[37-56]
p[39-58]
p[41-60]
p[43-62]
p[45-64]
p[47-66]
p[49-68]
p[51-70]
p[53-72]
p[55-74]
p[57-76]
p[59-78]
p[61-80]
p[63-82]
p[65-84]
p[67-86]
p[69-88]
p[71-90]
p[73-92]
p[75-94]
p[77-96]
p[79-98]
p[81-100]
p[83-102]
p[85-104]
p[87-106]
p[89-108]
p[91-110]
p[93-112]
p[95-114]
p[97-116]
p[99-118]
p[101-120]
p[103-122]
p[105-124]
p[107-126]
p[109-128]
p[111-130]
p[113-132]
p[115-134]
p[117-136]
p[119-138]
p[120-139]

M

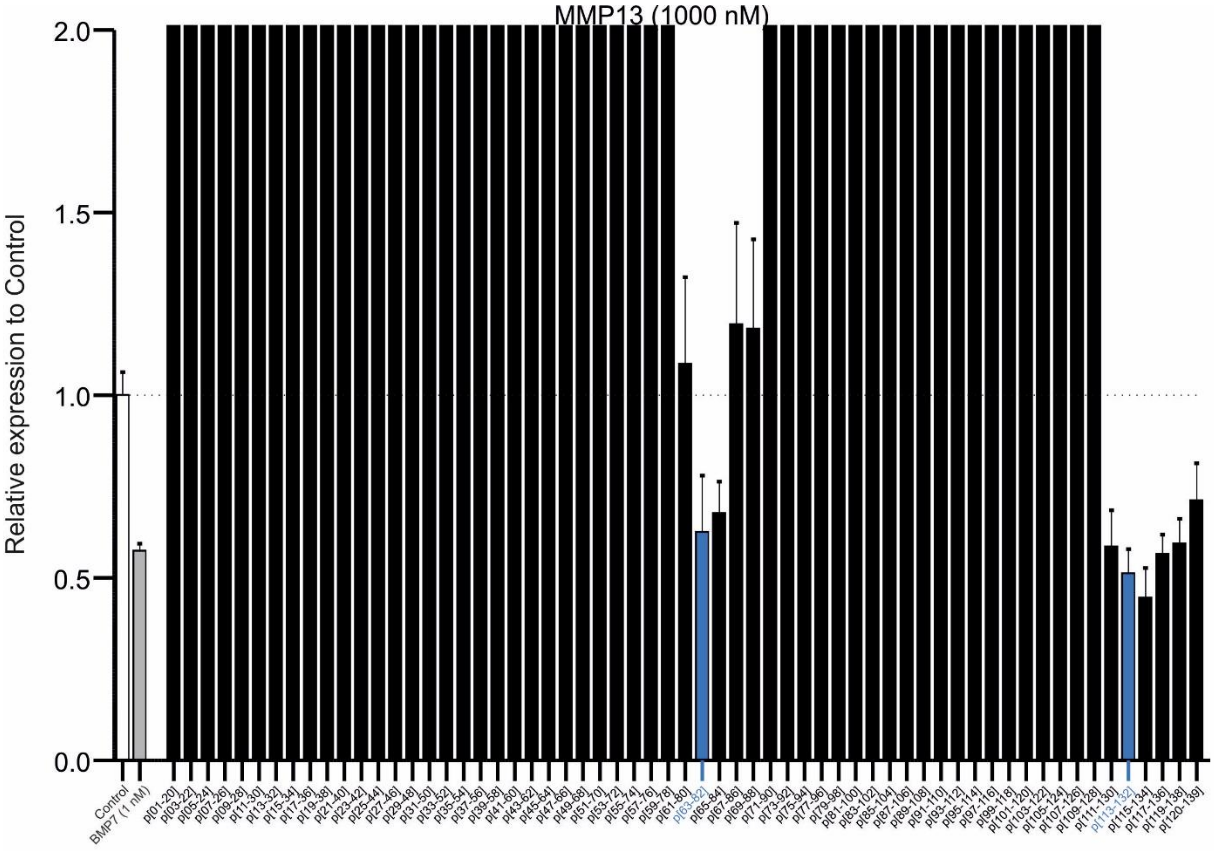
MMP13 (1000 nM)
Relative expression to Control
2.0
1.5
1.0
0.5
0.0
Control
BMP7 (1 nM)

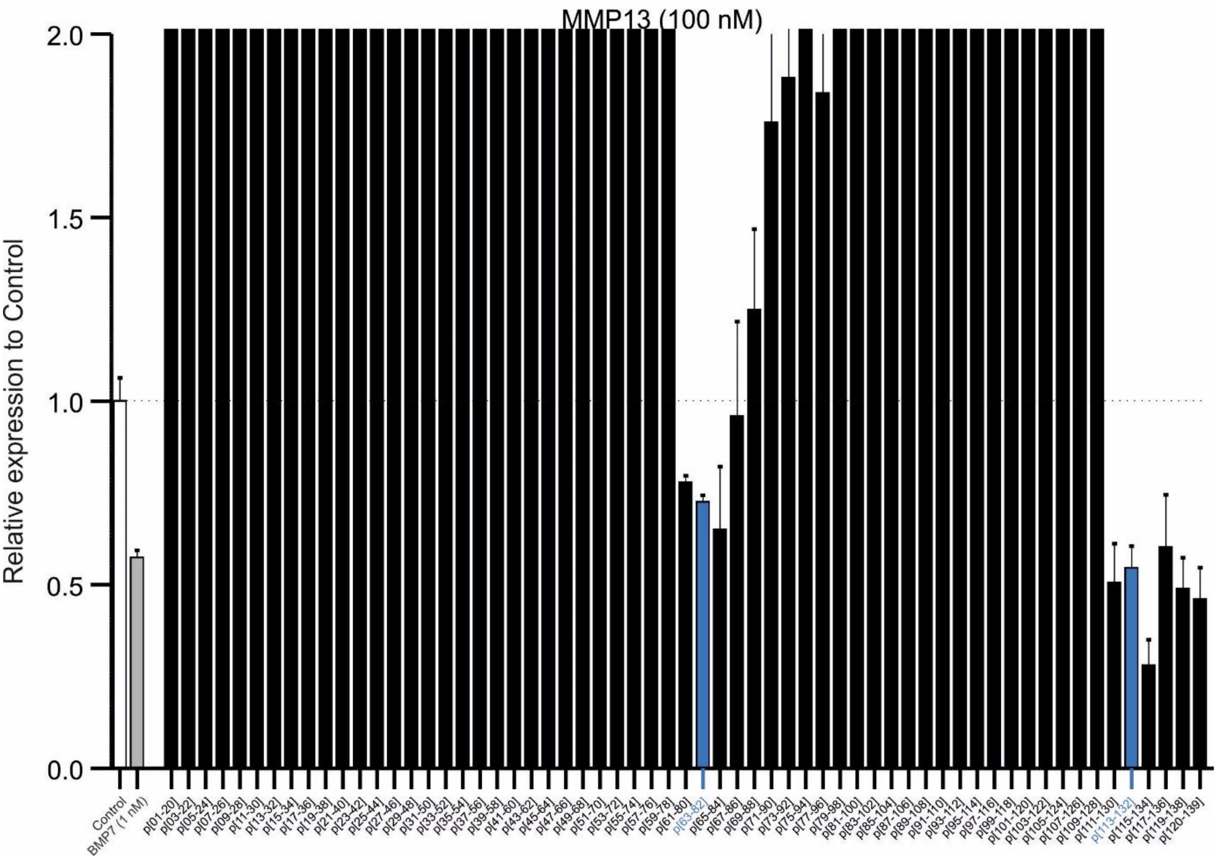
MMP13 (100 nM)
Relative expression to Control
2.0
1.5
1.0
0.5
0.0
Control
BMP7 (1 nM)

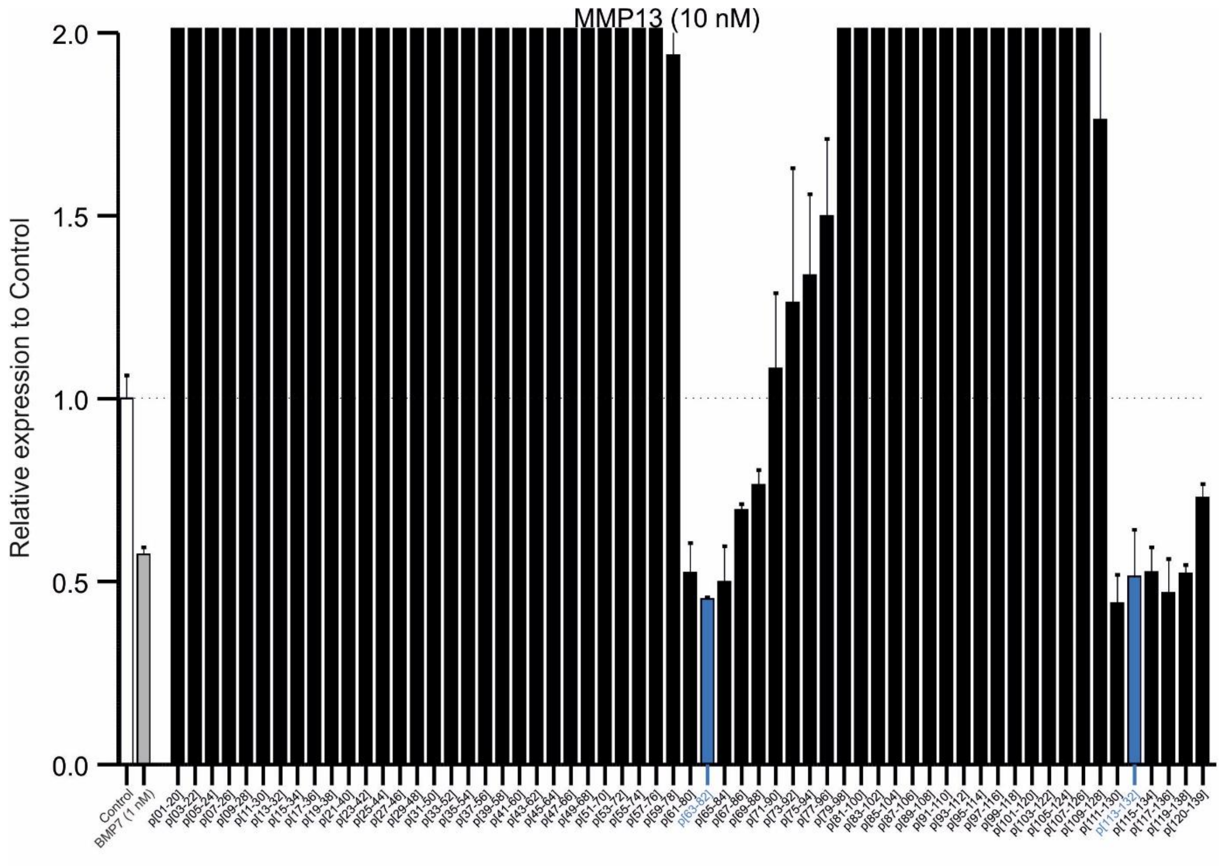
MMP13 (10 nM)
Relative expression to Control
2.0
1.5
1.0
0.5
0.0
Control
BMP7 (1 nM)
p[01-20]
p[03-22]
p[05-24]
p[07-26]
p[09-28]
p[11-30]
p[13-32]
p[15-34]
p[17-36]
p[19-38]
p[21-40]
p[23-42]
p[25-44]
p[27-46]
p[29-48]
p[31-50]
p[33-52]
p[35-54]
p[37-56]
p[39-58]
p[41-60]
p[43-62]
p[45-64]
p[47-66]
p[49-68]
p[51-70]
p[53-72]
p[55-74]
p[57-76]
p[59-78]
p[61-80]
p[63-82]
p[65-84]
p[67-86]
p[69-88]
p[71-90]
p[73-92]
p[75-94]
p[77-96]
p[79-98]
p[81-100]
p[83-102]
p[85-104]
p[87-106]
p[89-108]
p[91-110]
p[93-112]
p[95-114]
p[97-116]
p[99-118]
p[101-120]
p[103-122]
p[105-124]
p[107-126]
p[109-128]
p[111-130]
p[113-132]
p[115-134]
p[117-136]
p[119-138]
p[120-139]

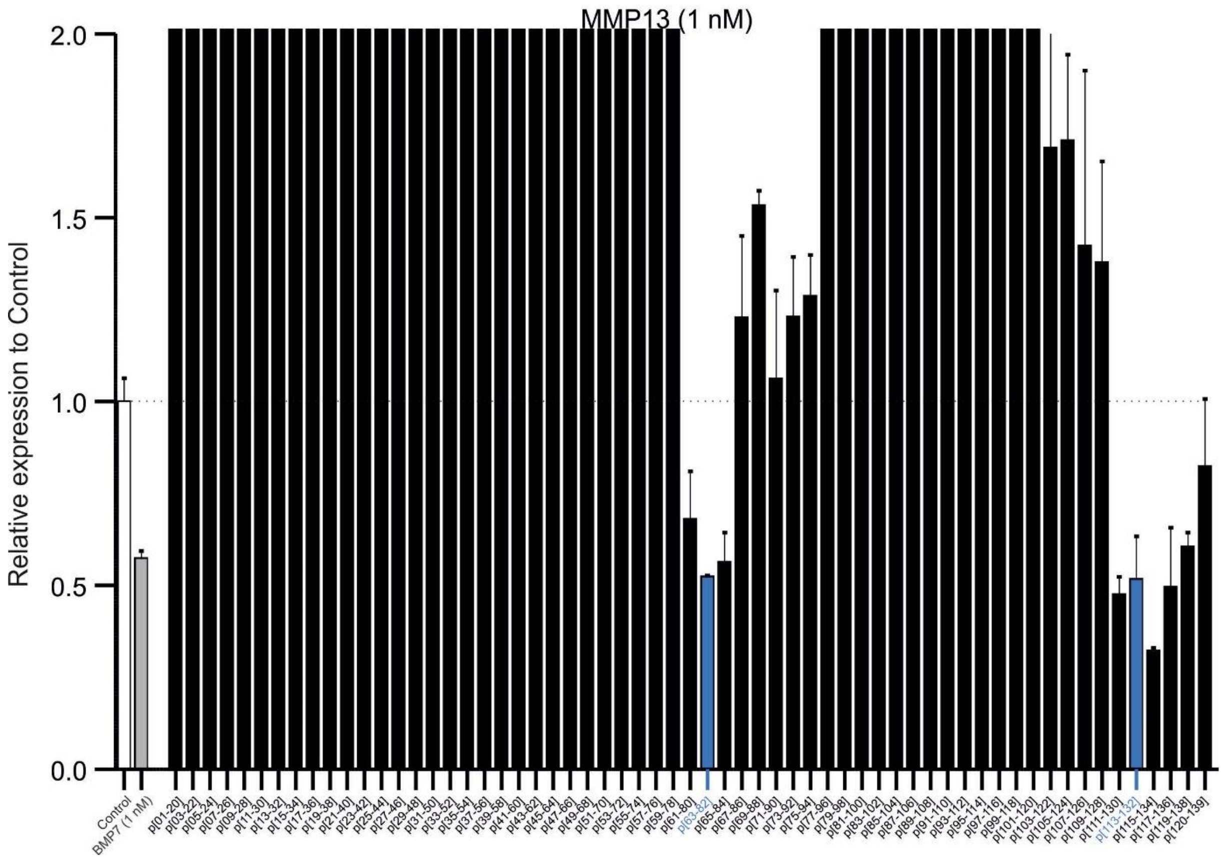
MMP13 (1 nM)
Relative expression to Control
2.0
1.5
1.0
0.5
0.0
Control
BMP7 (1 nM)
p[01-20]
p[03-22]
p[05-24]
p[07-26]
p[09-28]
p[11-30]
p[13-32]
p[15-34]
p[17-36]
p[19-38]
p[21-40]
p[23-42]
p[25-44]
p[27-46]
p[29-48]
p[31-50]
p[33-52]
p[35-54]
p[37-56]
p[39-58]
p[41-60]
p[43-62]
p[45-64]
p[47-66]
p[49-68]
p[51-70]
p[53-72]
p[55-74]
p[57-76]
p[59-78]
p[61-80]
p[63-82]
p[65-84]
p[67-86]
p[69-88]
p[71-90]
p[73-92]
p[75-94]
p[77-96]
p[79-98]
p[81-100]
p[83-102]
p[85-104]
p[87-106]
p[89-108]
p[91-110]
p[93-112]
p[95-114]
p[97-116]
p[99-118]
p[101-120]
p[103-122]
p[105-124]
p[107-126]
p[109-128]
p[111-130]
p[113-132]
p[115-134]
p[117-136]
p[119-138]
p[120-139]

N

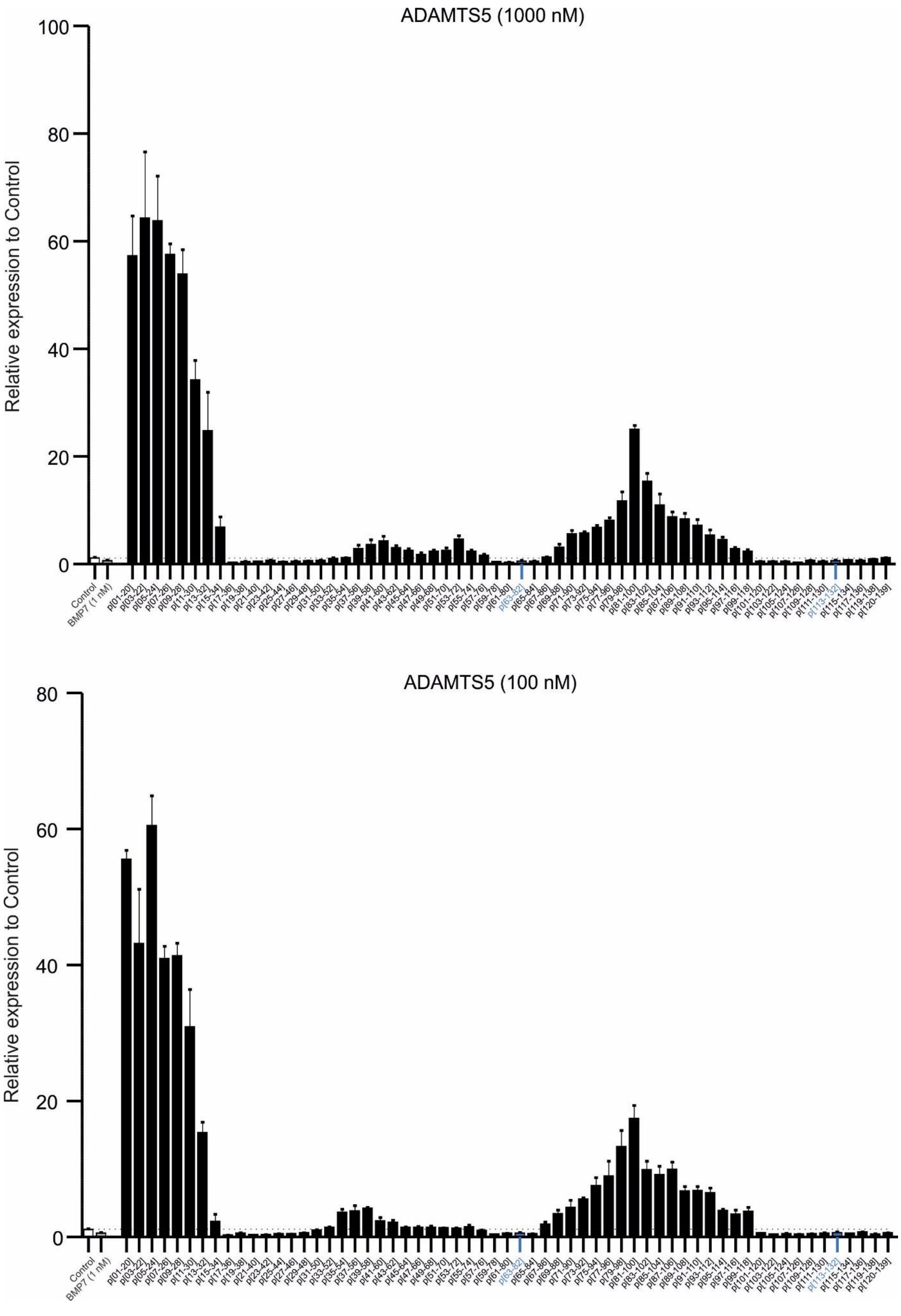
ADAMTS5 (1000 nM)
Relative expression to Control
100
80
60
40
20
0
Control
BMP7 (1 nM)
p[01-20] p[03-22] p[05-24] p[07-26] p[09-28] p[11-30] p[13-32] p[15-34] p[17-36] p[19-38] p[21-40] p[23-42] p[25-44] p[27-46] p[29-48] p[31-50] p[33-52] p[35-54] p[37-56] p[39-58] p[41-60] p[43-62] p[45-64] p[47-66] p[49-68] p[51-70] p[53-72] p[55-74] p[57-76] p[59-78] p[61-80] p[63-82] p[65-84] p[67-86] p[69-88] p[71-90] p[73-92] p[75-94] p[77-96] p[79-98] p[81-100] p[83-102] p[85-104] p[87-106] p[89-108] p[91-110] p[93-112] p[95-114] p[97-116] p[99-118] p[101-120] p[103-122] p[105-124] p[107-126] p[109-128] p[111-130] p[113-132] p[115-134] p[117-136] p[119-138] p[120-139]
ADAMTS5 (100 nM)
Relative expression to Control
80
60
40
20
0
Control
BMP7 (1 nM)
p[01-20] p[03-22] p[05-24] p[07-26] p[09-28] p[11-30] p[13-32] p[15-34] p[17-36] p[19-38] p[21-40] p[23-42] p[25-44] p[27-46] p[29-48] p[31-50] p[33-52] p[35-54] p[37-56] p[39-58] p[41-60] p[43-62] p[45-64] p[47-66] p[49-68] p[51-70] p[53-72] p[55-74] p[57-76] p[59-78] p[61-80] p[63-82] p[65-84] p[67-86] p[69-88] p[71-90] p[73-92] p[75-94] p[77-96] p[79-98] p[81-100] p[83-102] p[85-104] p[87-106] p[89-108] p[91-110] p[93-112] p[95-114] p[97-116] p[99-118] p[101-120] p[103-122] p[105-124] p[107-126] p[109-128] p[111-130] p[113-132] p[115-134] p[117-136] p[119-138] p[120-139]

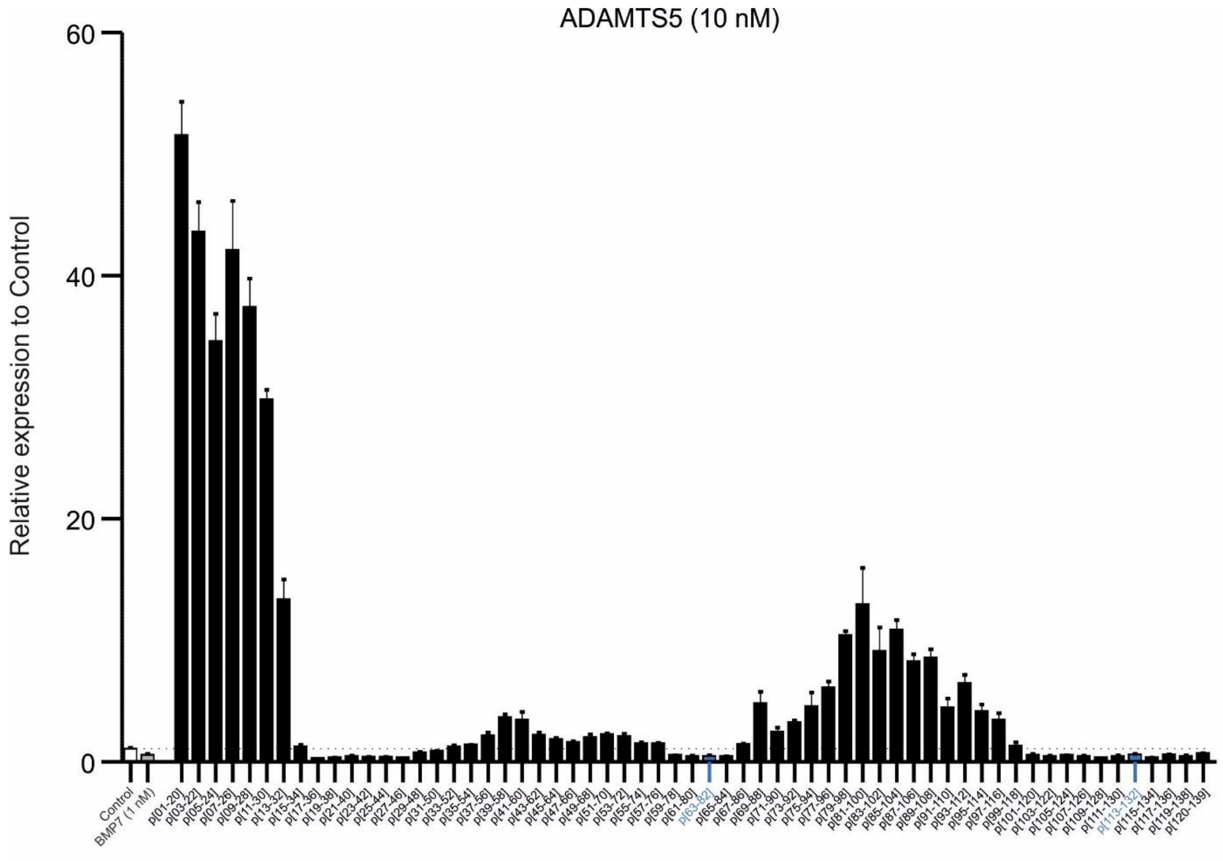
ADAMTS5 (10 nM)
Relative expression to Control
60
40
20
0
Control
BMP7 (1 nM)

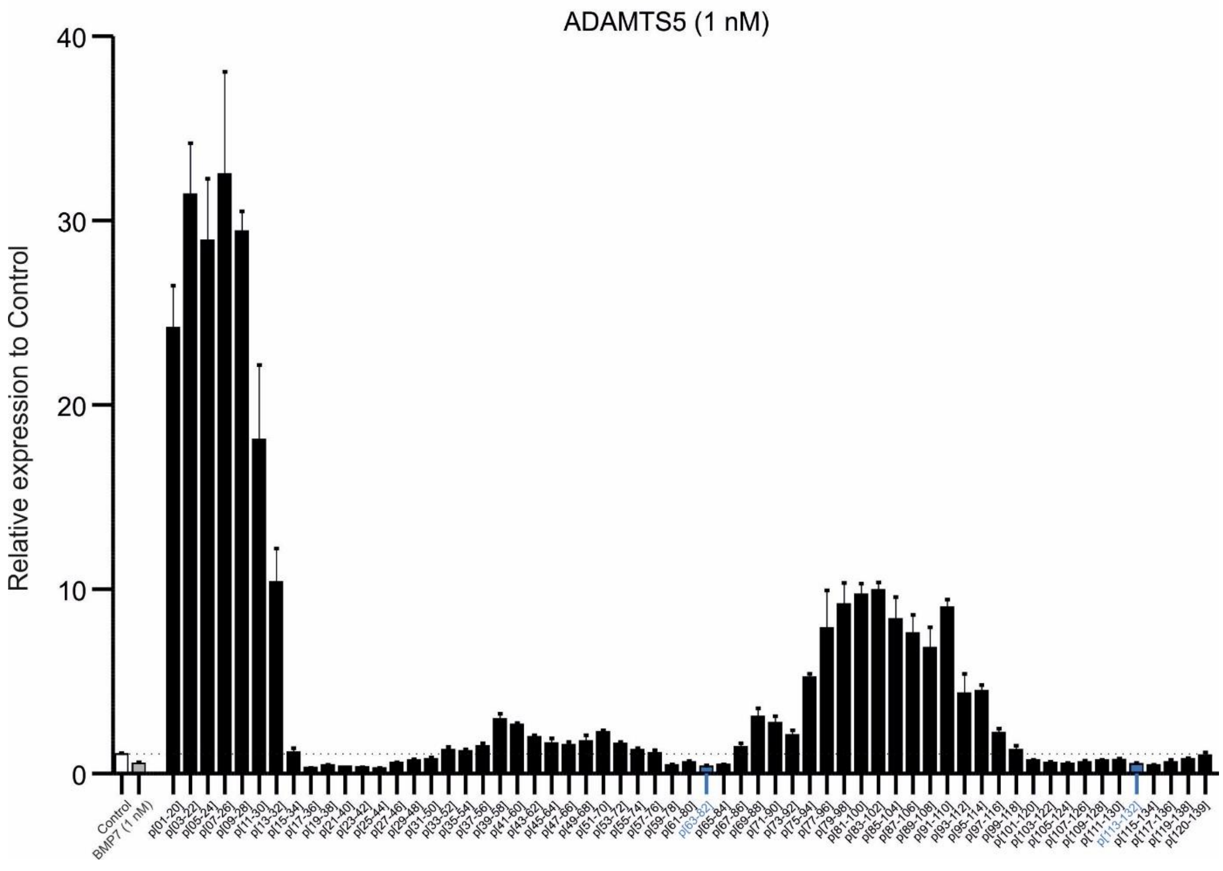
ADAMTS5 (1 nM)
Relative expression to Control
40
30
20
10
0
Control
BMP7 (1 nM)

O

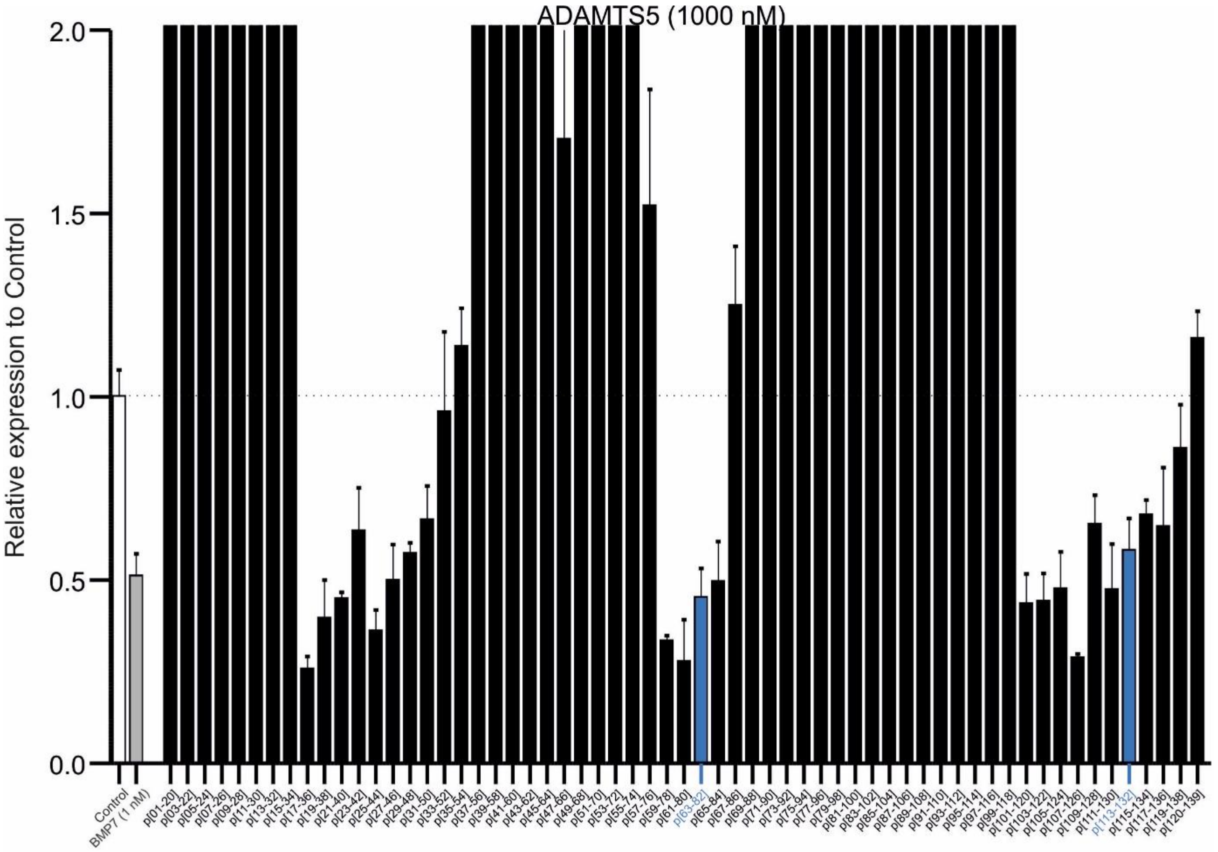
ADAMTS5 (1000 nM)
Relative expression to Control
0.0
0.5
1.0
1.5
2.0
Control
BMP7 (1 nM)
p[01-20]
p[03-22]
p[05-24]
p[07-26]
p[09-28]
p[11-30]
p[13-32]
p[15-34]
p[17-36]
p[19-38]
p[21-40]
p[23-42]
p[25-44]
p[27-46]
p[29-48]
p[31-50]
p[33-52]
p[35-54]
p[37-56]
p[39-58]
p[41-60]
p[43-62]
p[45-64]
p[47-66]
p[49-68]
p[51-70]
p[53-72]
p[55-74]
p[57-76]
p[59-78]
p[61-80]
p[63-82]
p[65-84]
p[67-86]
p[69-88]
p[71-90]
p[73-92]
p[75-94]
p[77-96]
p[79-98]
p[81-100]
p[83-102]
p[85-104]
p[87-106]
p[89-108]
p[91-110]
p[93-112]
p[95-114]
p[97-116]
p[99-118]
p[101-120]
p[103-122]
p[105-124]
p[107-126]
p[109-128]
p[111-130]
p[113-132]
p[115-134]
p[117-136]
p[119-138]
p[120-139]

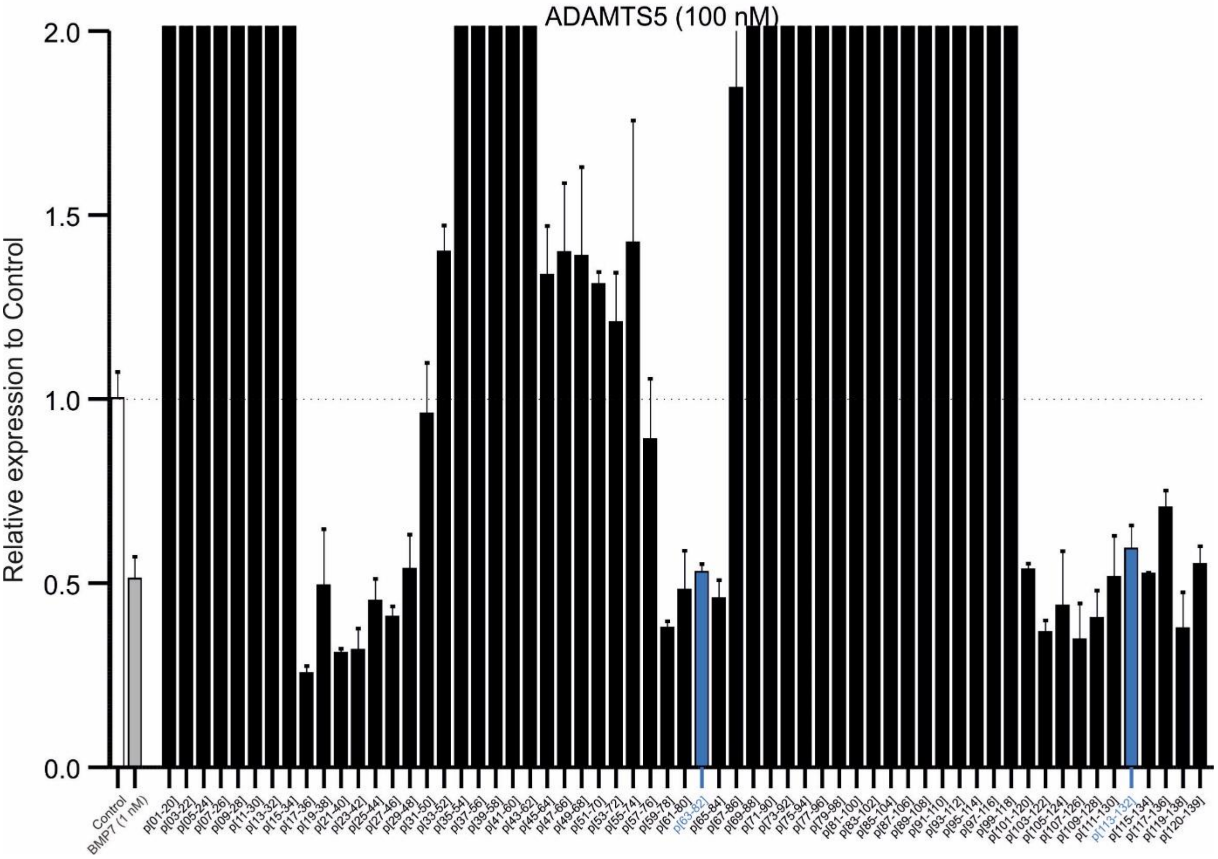
ADAMTS5 (100 nM)
Relative expression to Control
0.0
0.5
1.0
1.5
2.0
Control
BMP7 (1 nM)
p[01-20]
p[03-22]
p[05-24]
p[07-26]
p[09-28]
p[11-30]
p[13-32]
p[15-34]
p[17-36]
p[19-38]
p[21-40]
p[23-42]
p[25-44]
p[27-46]
p[29-48]
p[31-50]
p[33-52]
p[35-54]
p[37-56]
p[39-58]
p[41-60]
p[43-62]
p[45-64]
p[47-66]
p[49-68]
p[51-70]
p[53-72]
p[55-74]
p[57-76]
p[59-78]
p[61-80]
p[63-82]
p[65-84]
p[67-86]
p[69-88]
p[71-90]
p[73-92]
p[75-94]
p[77-96]
p[79-98]
p[81-100]
p[83-102]
p[85-104]
p[87-106]
p[89-108]
p[91-110]
p[93-112]
p[95-114]
p[97-116]
p[99-118]
p[101-120]
p[103-122]
p[105-124]
p[107-126]
p[109-128]
p[111-130]
p[113-132]
p[115-134]
p[117-136]
p[119-138]
p[120-139]

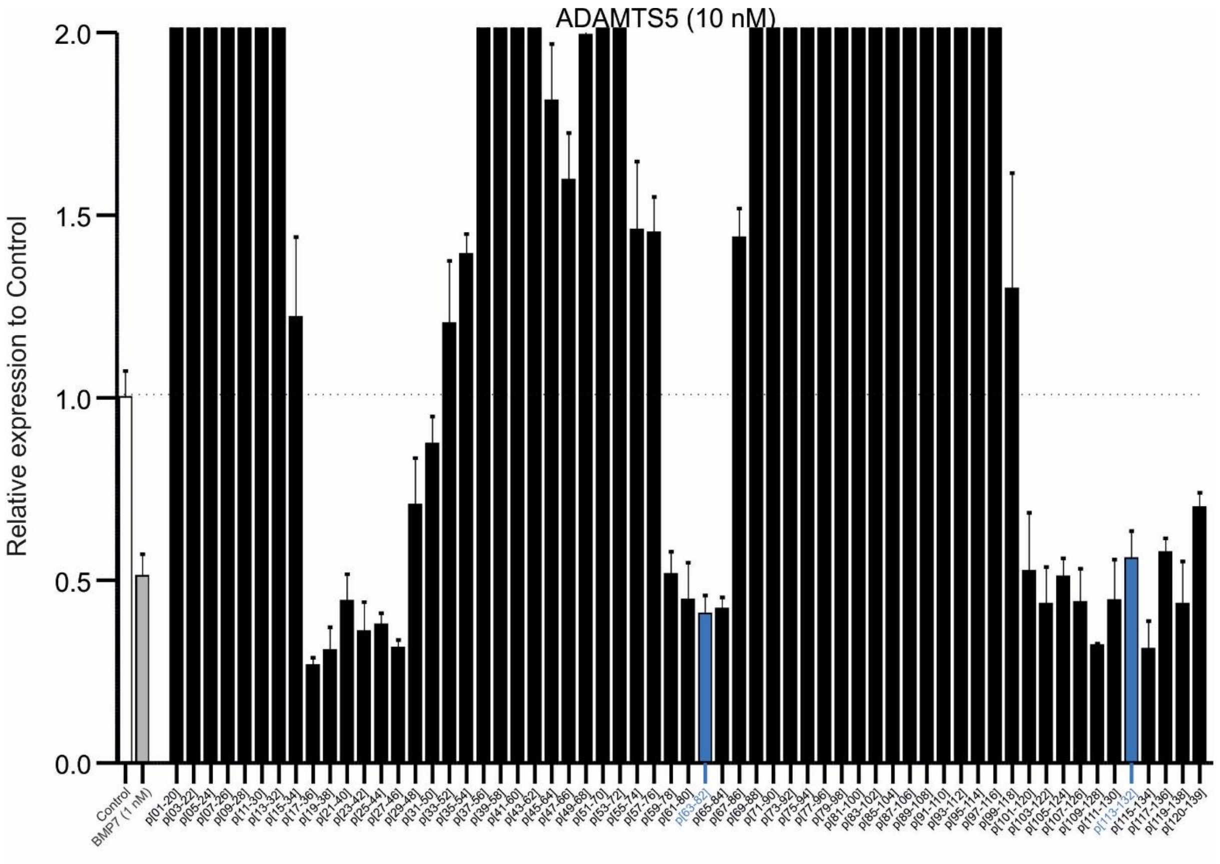
ADAMTS5 (10 nM)
Relative expression to Control
2.0
1.5
1.0
0.5
0.0
Control
BMP7 (1 nM)

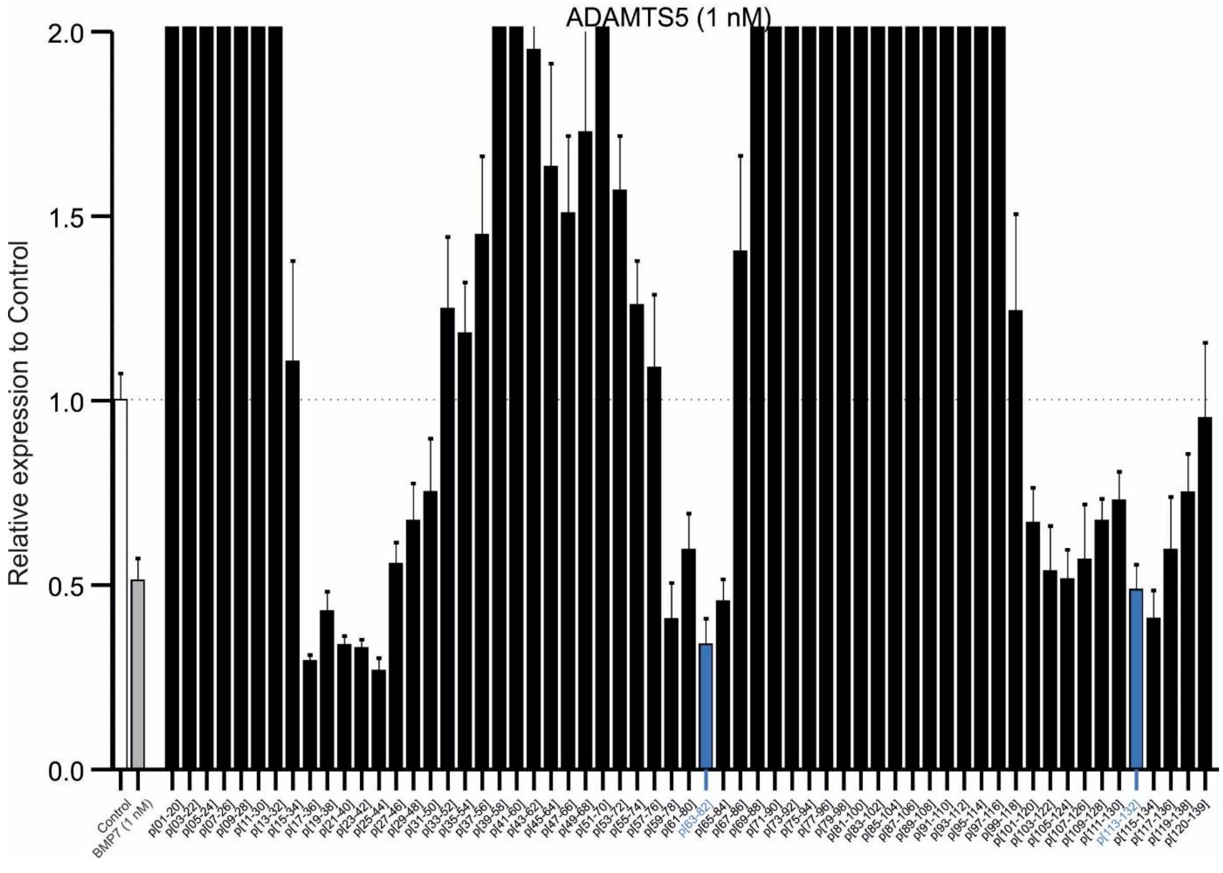
ADAMTS5 (1 nM)
Relative expression to Control
2.0
1.5
1.0
0.5
0.0
Control
BMP7 (1 nM)

P

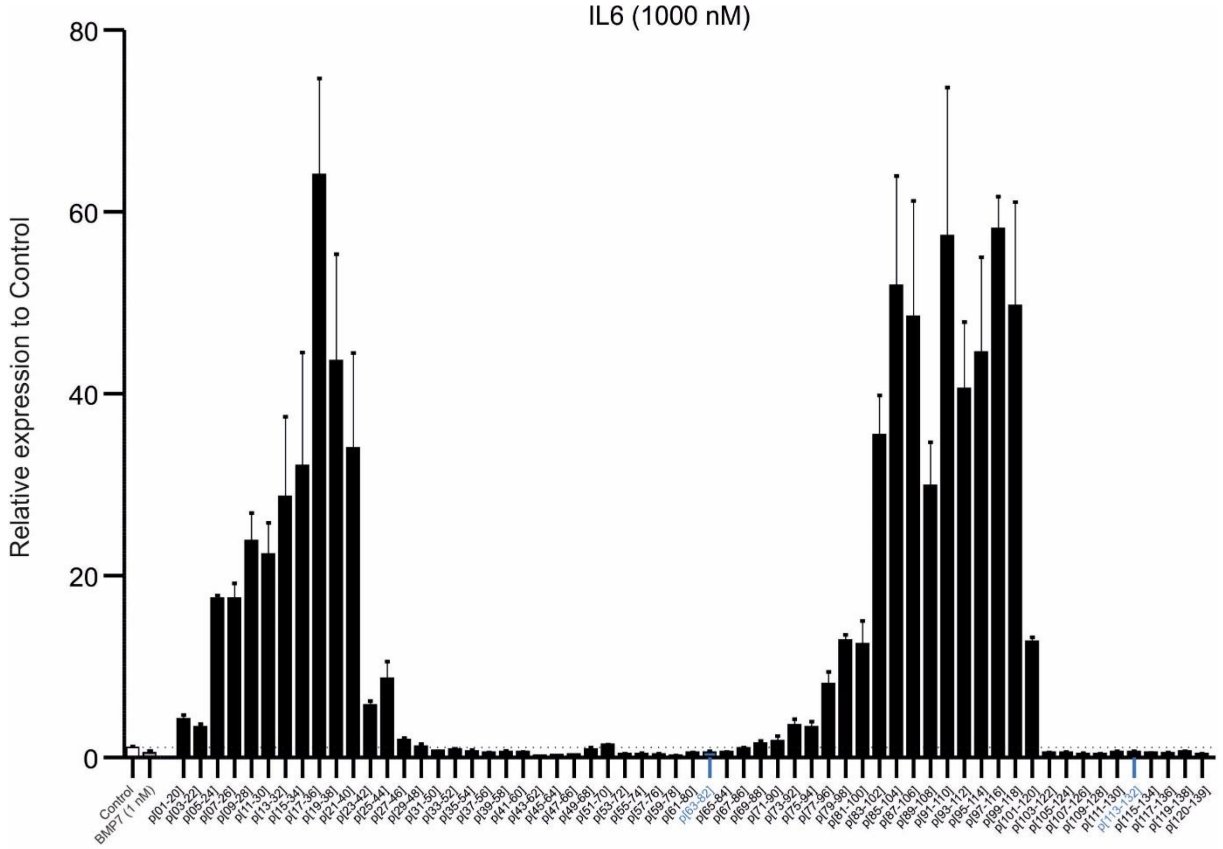
IL6 (1000 nM)
Relative expression to Control
0
20
40
60
80
Control
BMP7 (1 nM)
p[01-20]
p[03-22]
p[05-24]
p[07-26]
p[09-28]
p[11-30]
p[13-32]
p[15-34]
p[17-36]
p[19-38]
p[21-40]
p[23-42]
p[25-44]
p[27-46]
p[29-48]
p[31-50]
p[33-52]
p[35-54]
p[37-56]
p[39-58]
p[41-60]
p[43-62]
p[45-64]
p[47-66]
p[49-68]
p[51-70]
p[53-72]
p[55-74]
p[57-76]
p[59-78]
p[61-80]
p[63-82]
p[65-84]
p[67-86]
p[69-88]
p[71-90]
p[73-92]
p[75-94]
p[77-96]
p[79-98]
p[81-100]
p[83-102]
p[85-104]
p[87-106]
p[89-108]
p[91-110]
p[93-112]
p[95-114]
p[97-116]
p[99-118]
p[101-120]
p[103-122]
p[105-124]
p[107-126]
p[109-128]
p[111-130]
p[113-132]
p[115-134]
p[117-136]
p[119-138]
p[120-139]

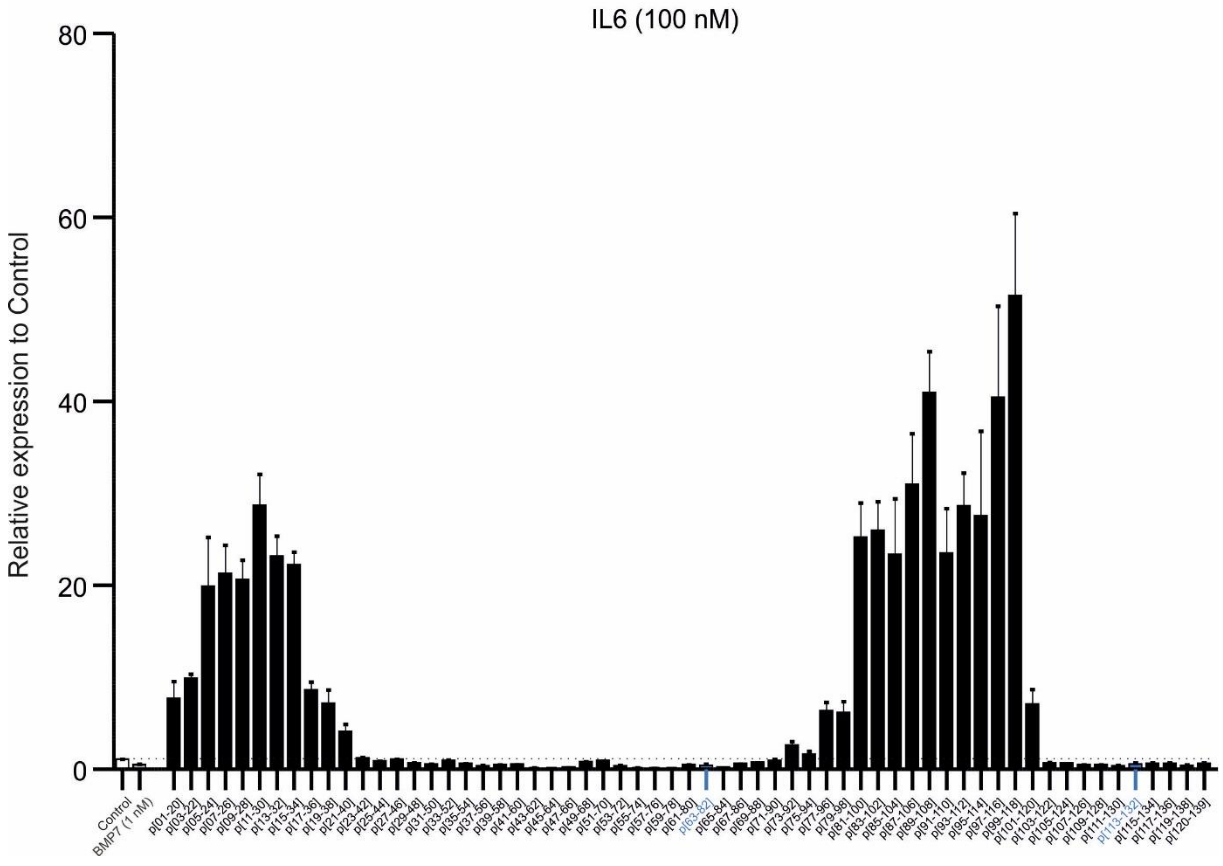
IL6 (100 nM)
Relative expression to Control
0
20
40
60
80
Control
BMP7 (1 nM)
p[01-20]
p[03-22]
p[05-24]
p[07-26]
p[09-28]
p[11-30]
p[13-32]
p[15-34]
p[17-36]
p[19-38]
p[21-40]
p[23-42]
p[25-44]
p[27-46]
p[29-48]
p[31-50]
p[33-52]
p[35-54]
p[37-56]
p[39-58]
p[41-60]
p[43-62]
p[45-64]
p[47-66]
p[49-68]
p[51-70]
p[53-72]
p[55-74]
p[57-76]
p[59-78]
p[61-80]
p[63-82]
p[65-84]
p[67-86]
p[69-88]
p[71-90]
p[73-92]
p[75-94]
p[77-96]
p[79-98]
p[81-100]
p[83-102]
p[85-104]
p[87-106]
p[89-108]
p[91-110]
p[93-112]
p[95-114]
p[97-116]
p[99-118]
p[101-120]
p[103-122]
p[105-124]
p[107-126]
p[109-128]
p[111-130]
p[113-132]
p[115-134]
p[117-136]
p[119-138]
p[120-139]

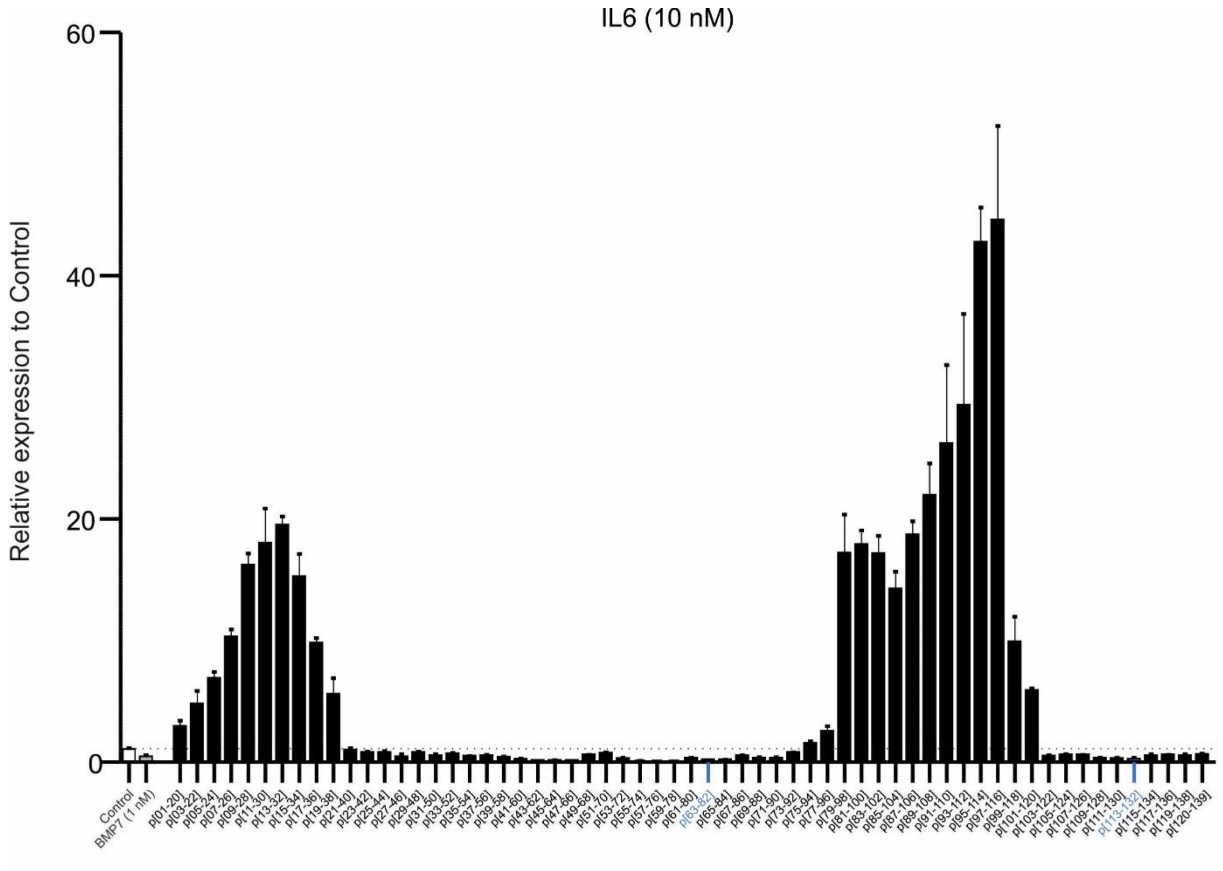
IL6 (10 nM)
Relative expression to Control
60
40
20
0
Control
BMP7 (1 nM)
p[01-20]
p[03-22]
p[05-24]
p[07-26]
p[09-28]
p[11-30]
p[13-32]
p[15-34]
p[17-36]
p[19-38]
p[21-40]
p[23-42]
p[25-44]
p[27-46]
p[29-48]
p[31-50]
p[33-52]
p[35-54]
p[37-56]
p[39-58]
p[41-60]
p[43-62]
p[45-64]
p[47-66]
p[49-68]
p[51-70]
p[53-72]
p[55-74]
p[57-76]
p[59-78]
p[61-80]
p[63-82]
p[65-84]
p[67-86]
p[69-88]
p[71-90]
p[73-92]
p[75-94]
p[77-96]
p[79-98]
p[81-100]
p[83-102]
p[85-104]
p[87-106]
p[89-108]
p[91-110]
p[93-112]
p[95-114]
p[97-116]
p[99-118]
p[101-120]
p[103-122]
p[105-124]
p[107-126]
p[109-128]
p[111-130]
p[113-132]
p[115-134]
p[117-136]
p[119-138]
p[120-139]

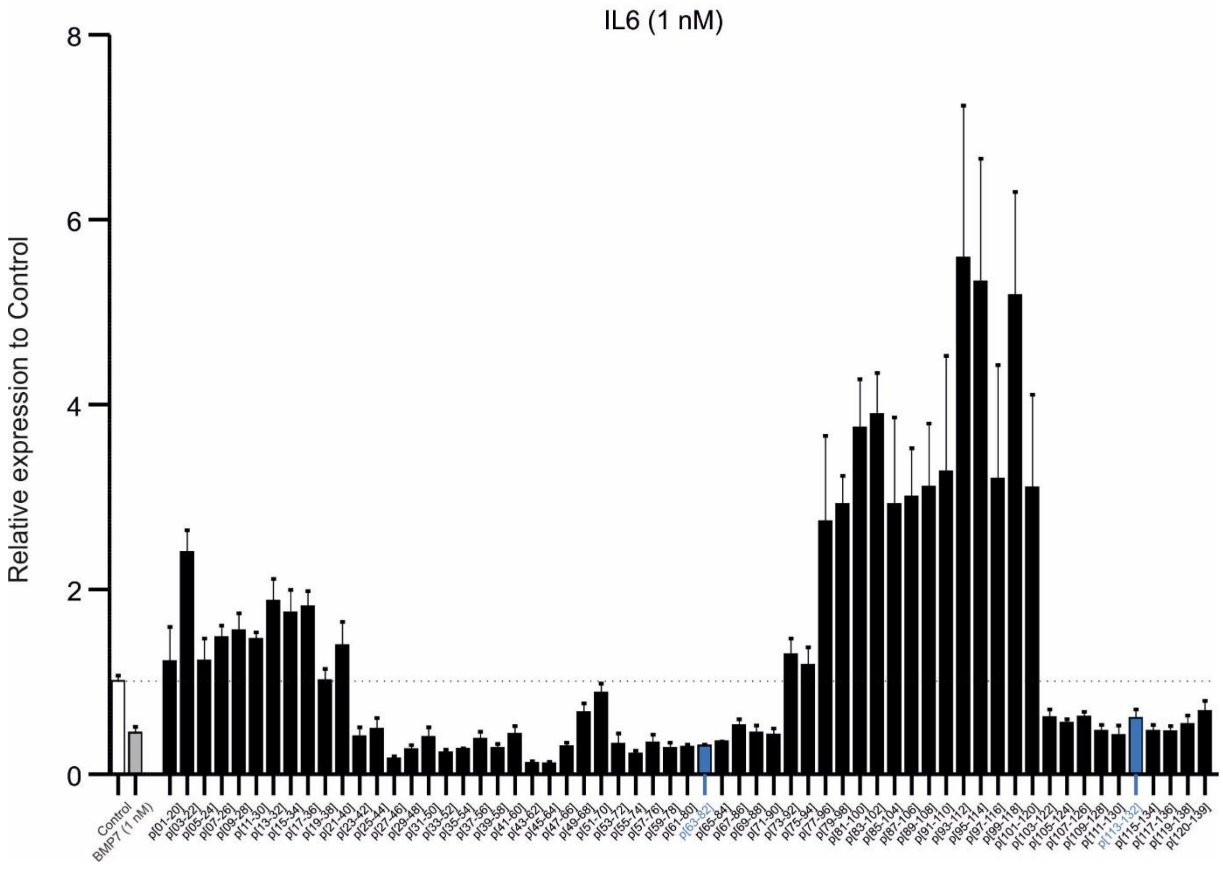
IL6 (1 nM)
Relative expression to Control
8
6
4
2
0
Control
BMP7 (1 nM)
p[01-20]
p[03-22]
p[05-24]
p[07-26]
p[09-28]
p[11-30]
p[13-32]
p[15-34]
p[17-36]
p[19-38]
p[21-40]
p[23-42]
p[25-44]
p[27-46]
p[29-48]
p[31-50]
p[33-52]
p[35-54]
p[37-56]
p[39-58]
p[41-60]
p[43-62]
p[45-64]
p[47-66]
p[49-68]
p[51-70]
p[53-72]
p[55-74]
p[57-76]
p[59-78]
p[61-80]
p[63-82]
p[65-84]
p[67-86]
p[69-88]
p[71-90]
p[73-92]
p[75-94]
p[77-96]
p[79-98]
p[81-100]
p[83-102]
p[85-104]
p[87-106]
p[89-108]
p[91-110]
p[93-112]
p[95-114]
p[97-116]
p[99-118]
p[101-120]
p[103-122]
p[105-124]
p[107-126]
p[109-128]
p[111-130]
p[113-132]
p[115-134]
p[117-136]
p[119-138]
p[120-139]

Q

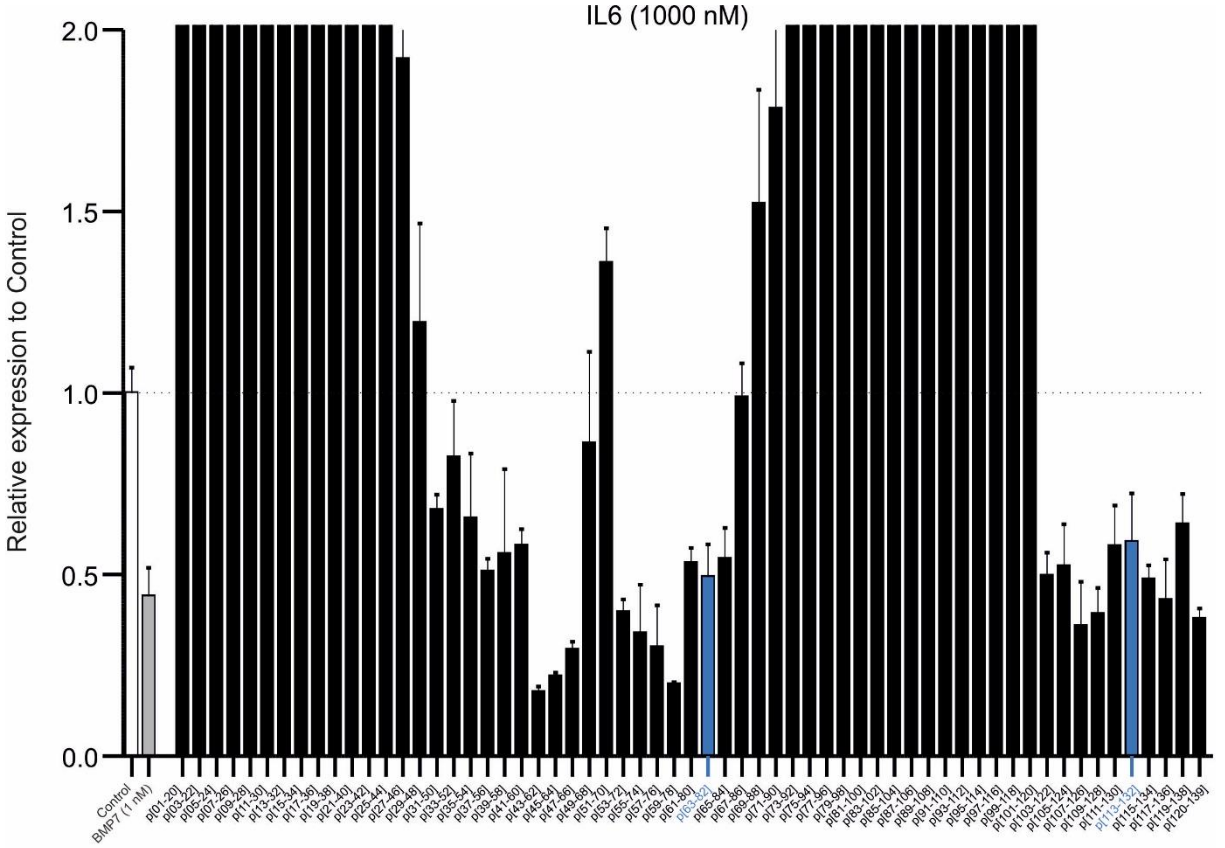
IL6 (1000 nM)
Relative expression to Control
2.0
1.5
1.0
0.5
0.0
Control
BMP7 (1 nM)

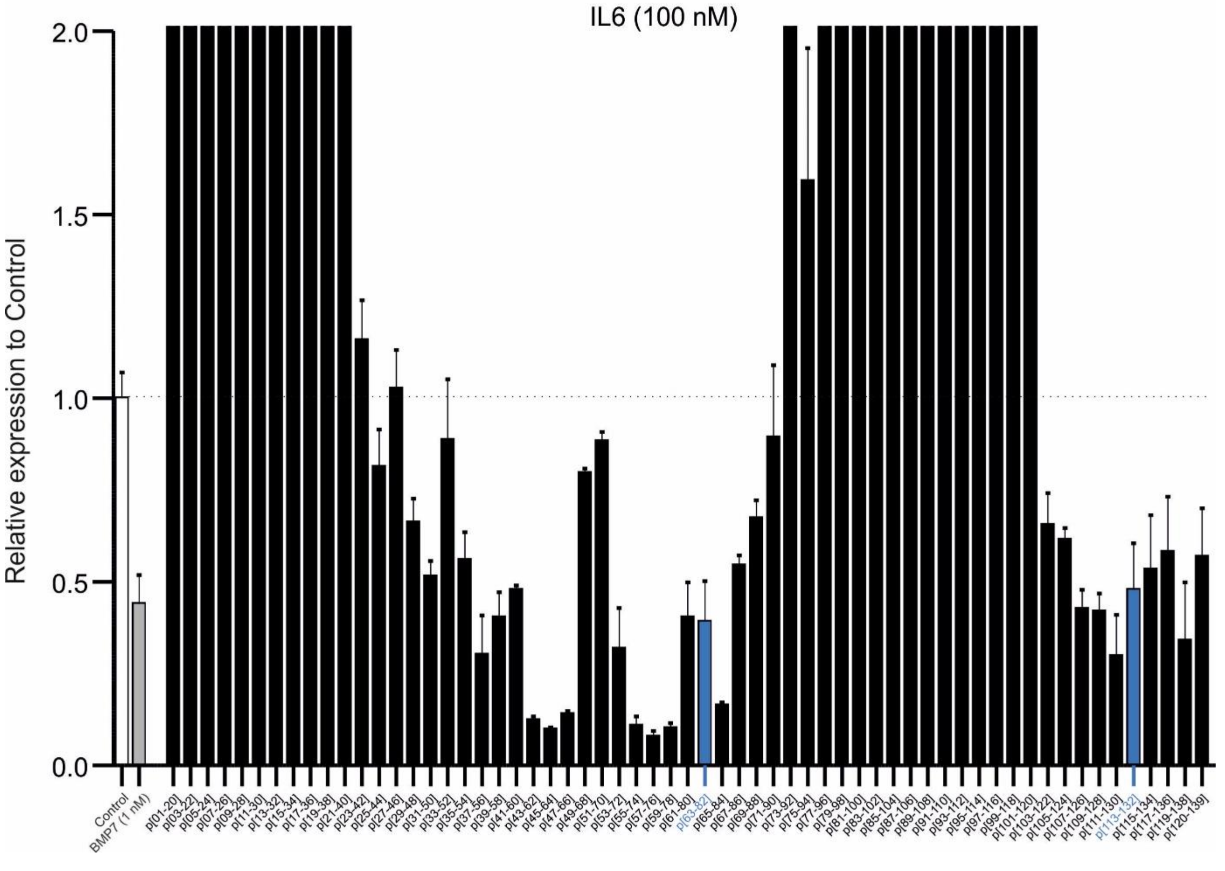
IL6 (100 nM)
Relative expression to Control
2.0
1.5
1.0
0.5
0.0
Control
BMP7 (1 nM)

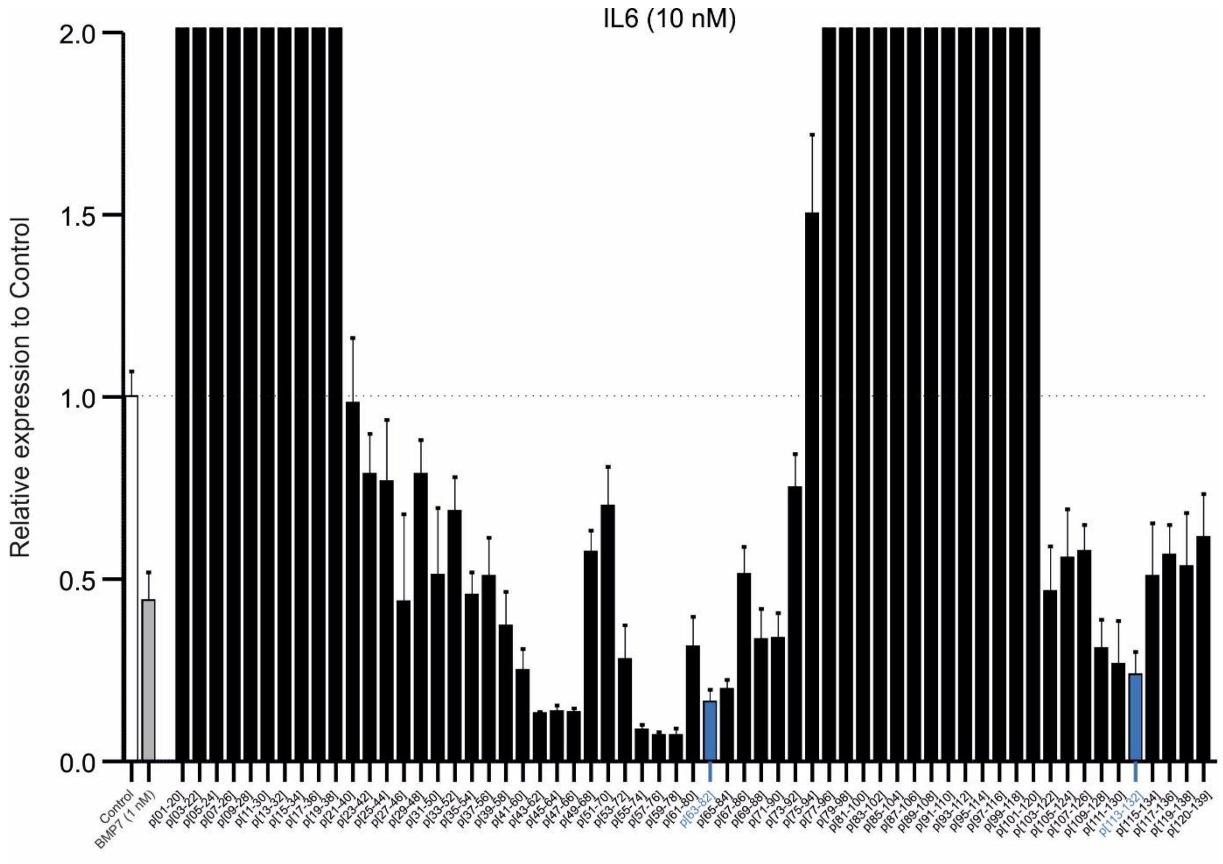
IL6 (10 nM)
Relative expression to Control
2.0
1.5
1.0
0.5
0.0
Control
BMP7 (1 nM)

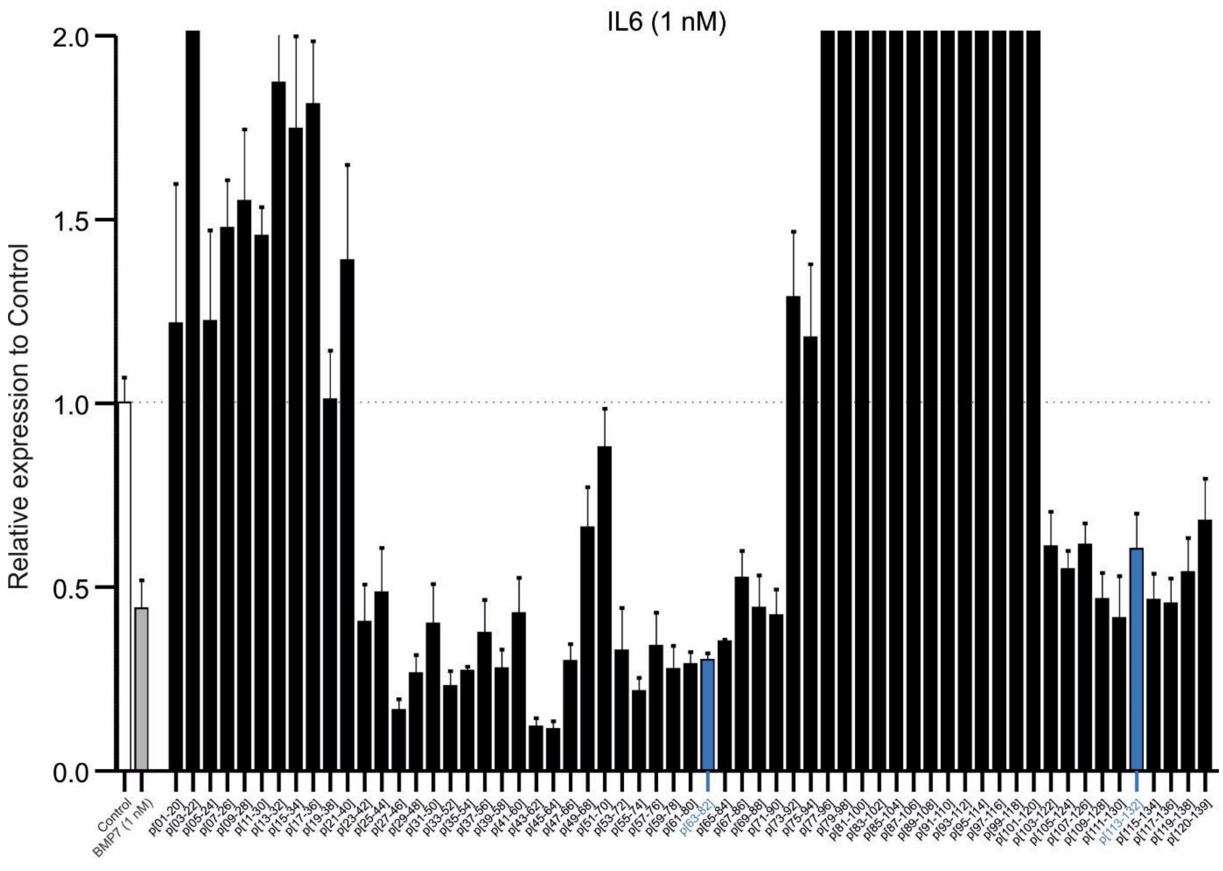
IL6 (1 nM)
Relative expression to Control
2.0
1.5
1.0
0.5
0.0
Control
BMP7 (1 nM)

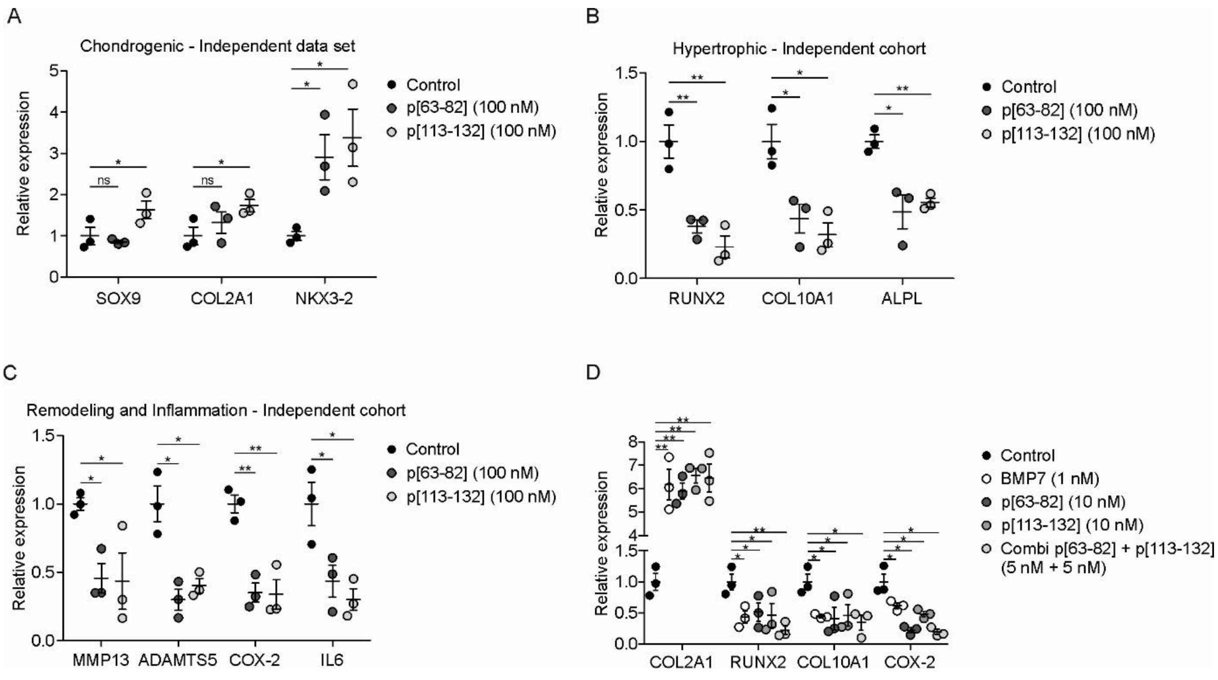

Supplementary Figure 3: Bioactivity of peptides p[63-82] and p[113-132] on independent OA-HAC cohort. A: Chondrogenic gene expression (SOX9, COL2A1, NKX3-2) in independent cohort of 3 individual OA-HAC donors. **B:** Hypertrophic gene expression (RUNX2, COL10A1, ALPL) expression in independent cohort of 3 individual OA-HAC donors. **C:** Remodelling (MMP13, ADAMTS5) and Inflammation (COX-2, IL6) gene expression in independent cohort of 3 individual OA-HAC donors. Conditions were: Control, p[63-82] and p[113-132]. Peptides were used at 100 nM each. **D:** Chondrogenic, hypertrophic and inflammatory gene expression (COL2A1, RUNX2, COL10A1, COX-2) in independent cohort of 3 individual OA-HAC donors. Conditions were: BMP7 (1 nM), Control, p[63-82] (10 nM), p[113-132] (10 nM), combination of p[63-82] + p[113-132] (5 + 5 nM). All OA-HAC cultures were exposed to peptides for 24 hours. Gene expression was normalized for 28S rRNA expression and set relative to control condition. Error bars represent mean ± SEM and statistical significance for peptide conditions versus control condition as determined by unpaired two-tailed student's t-test is represented as: * is $p<0.05$, ** is $p<0.01$, *** is $p<0.001$ and ns is=not significant.

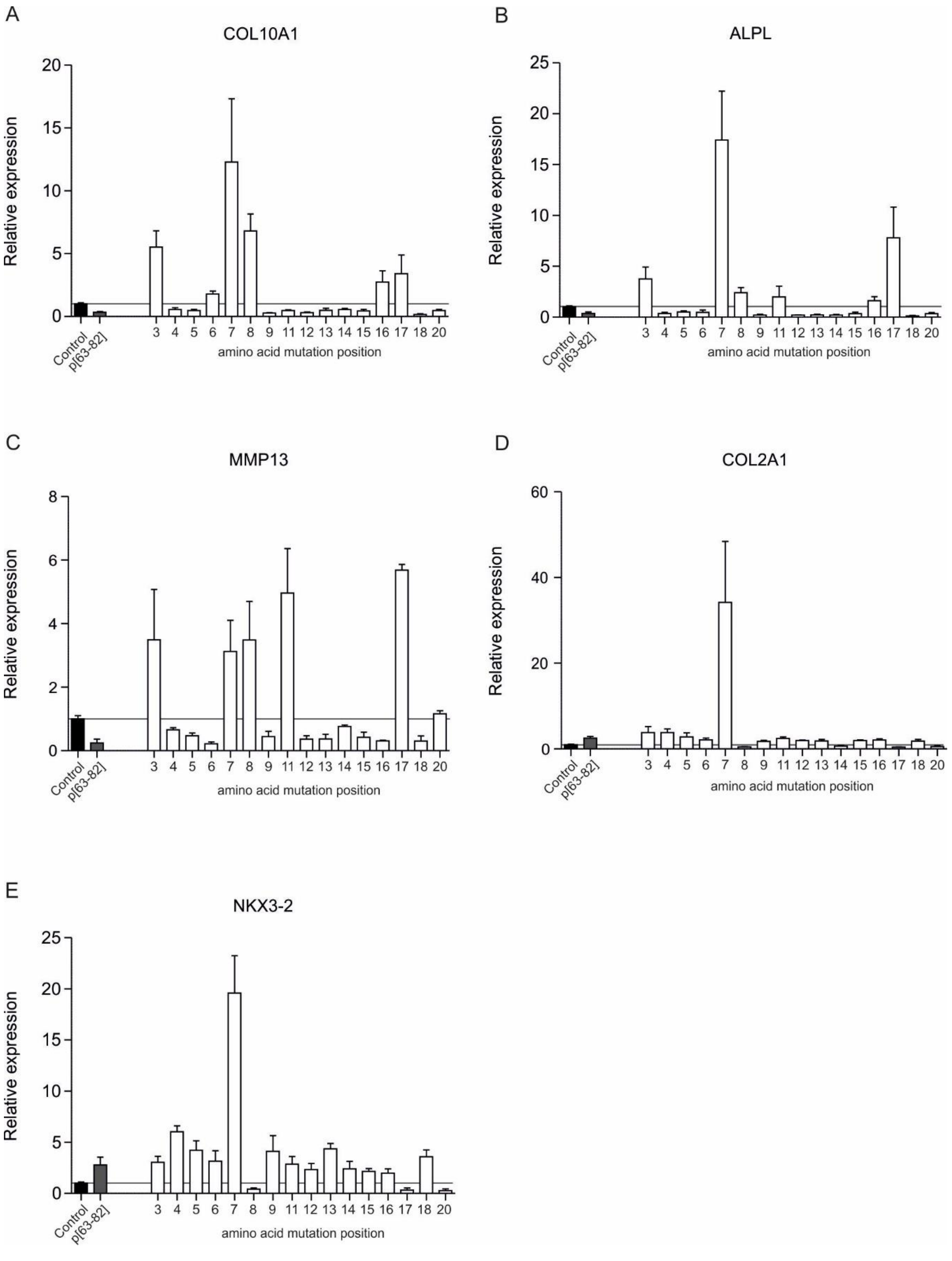

Supplementary Figure 4: Alanine scanning of peptide p[63-82]. A: COL10A1 mRNA expression in OA-HAC pool (n=18 individual donors). **B**: ALPL mRNA expression in OA-HAC pool (n=18). **C:** MMP13 mRNA expression in OA-HAC pool (n=18). **D:** COL2A1 mRNA expression in OA-HAC pool (n=18). **E:** NKX3-2 mRNA expression in OA-HAC pool

(n=18). Conditions were: Control, p[63-82] and peptides with amino acid substitution to alanine at indicated positions in p[63-82]. Peptides were used at 100 nM each. All OA-HAC cultures were exposed to peptides for 24 hours. Gene expression was normalized for 28S rRNA expression and set relative to control condition. Error bars represent mean ± SEM.

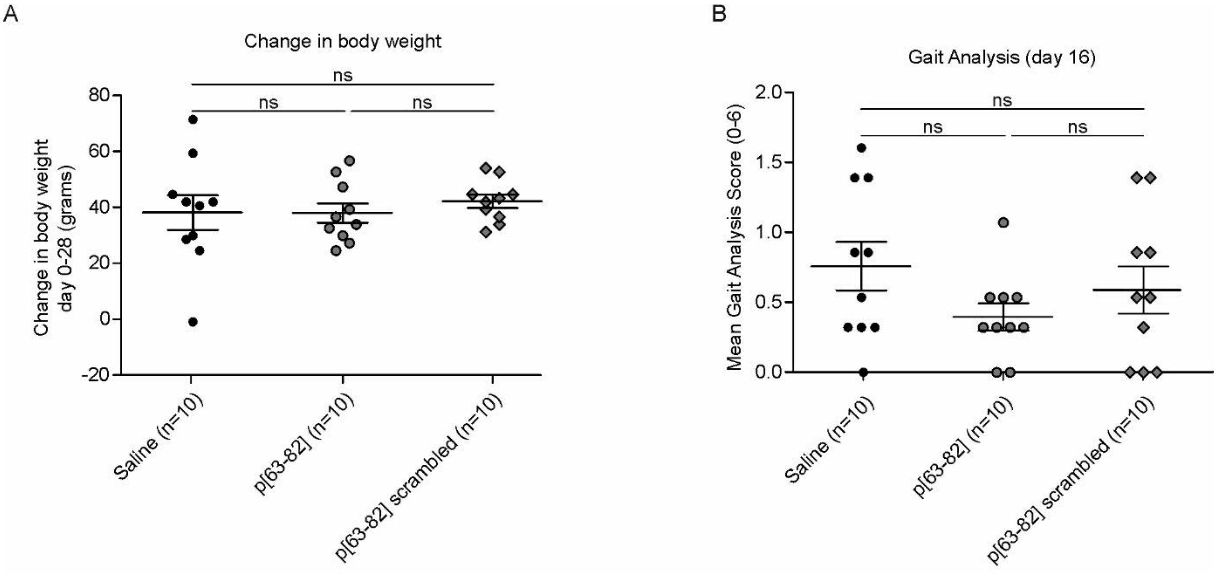

C

Parameter | Mean (SE)		Saline (n=10)	p[63-82] (n=10)	p[63-82] scrambled (n=10)	% Change p[63-82] vs Saline
**Medial Tibia Cartilage Degeneration Score**					
	Zone 1 (outside)	3,8 (0,2)	**2,6 (0,4)***	3,3 (0,4)	32%
	Zone 2 (middle)	1,2 (0,1)	1,0 (0,1)	1,2 (0,2)	17%
	Zone 3 (inside)	0,2 (0,1)	0,4 (0,1)	0,4 (0,1)	-100%
	Total	5,2 (0,2)	**4,0 (0,5)***	4,8 (0,6)	23%
**Medial Tibia Depth Ratio**					
	Zone 1 (outside)	0,93 (0,03)	**0,58 (0,11)****	0,74 (0,07)	38%
	Zone 2 (middle)	0,16 (0,05)	0,15 (0,03)	0,16 (0,05)	6%
	Zone 3 (inside)	0,01 (0,01)	0,02 (0,01)	0,02 (0,01)	-100%
	Total	0,37 (0,02)	**0,25 (0,03)***	0,30 (0,04)	32%
**Tibial Cartilage Degeneration Width (um)**					
	Substantial	595,0 (29,3)	370,0 (88,3)	495,0 (62,1)	38%
	Total	1500,0 (136,6)	1690,0 (116,9)	1660,0 (122,2)	-13%
**Total Medial Tibia Bone Score**					
	Damage	2,8 (0,2)	2,2 (0,3)	2,8 (0,3)	22%
	Sclerosis	2,8 (0,2)	2,5 (0,2)	2,6 (0,3)	11%
**Medial Femur Cartilage Degeneration Score**					
	Zone 1 (outside)	1,15 (0,3)	0,45 (0,1)	0,90 (0,2)	61%
	Zone 2 (middle)	0,40 (0,3)	0,05 (0,1)	0,10 (0,1)	88%
	Zone 3 (inside)	0,35 (0,3)	0,05 (0,1)	0,30 (0,3)	86%
	Total	1,90 (0,4)	**0,55 (0,1)****	1,30 (0,3)	71%
**Medial Tibia Osteophytes**					
	Measure (um)	489,0 (34,1)	500,0 (31,6)	494,0 (37,8)	-2%
	Score	3,8 (0,4)	3,8 (0,3)	3,7 (0,3)	0%
**Total Joint Score**					
	Without femur	9,0 (0,4)	7,9 (0,7)	8,5 (0,6)	12%
	With femur	11,1 (0,5)	**8,3 (0,9)***	9,9 (0,7)	25%
**Synovitis Score**					
		0,70 (0,1)	0,80 (0,2)	0,80 (0,1)	-14%

D

Parameter | Mean (SE)		Saline (n=10)	p[63-82] (n=10)	p[63-82] scrambled (n=10)	% Change p[63-82] vs Saline
Collagen Degeneration by Severity (% to total surface)					
	Total	46,2 (2,2)	48,5 (1,8)	43,3 (2,4)	-5%
	Severe	9,9 (2,5)	**3,0 (1,5)***	1,5 (1,5)	70%
	Marked	2,8 (1,3)	1,0 (0,7)	1,5 (1,1)	64%
	Moderate	1,3 (0,7)	0,5 (0,5)	3,0 (1,1)	62%
	Mild	6,1 (2,9)	9,2 (4,0)	11,4 (1,9)	-51%
	Minimal	26,5 (1,7)	35,0 (4,1)	25,9 (1,8)	-32%
Collagen Combined Degeneration (%)					
	Severe to Mild	20,0 (1,8)	13,7 (4,1)	17,4 (3,6)	32%
	Severe to Moderate	13,9 (2,9)	**4,5 (2,4)***	6,0 (2,7)	68%
	Mild to Minimal	32,4 (3,5)	**44,0 (3,0)***	37,3 (1,7)	-36%
	Severe to Marked	12,7 (2,7)	**4,0 (2,1)***	3,0 (2,1)	69%
	Moderate to Mild	33,7 (3,3)	**44,5 (2,6)***	40,3 (2,1)	-32%
Growth Plate Thickness (um)					
	Difference (Medial - Lateral)	28,0 (0,1)	40,0 (10,3)	32,0 (8,0)	-43%
Medial Collateral Ligament Thickness (um)					
		585,0 (20,1)	580,0 (12,3)	564,0 (27,0)	-1%

Supplementary Figure 5: BMP7-derived peptide p[63-82] in rat MMT model. The potency of BMP7 peptide p[63-82] to delay the progression of trauma-induced cartilage degeneration

was tested in the rat MMT model. One week post-MMT-surgery, rats were two times per week intra-articularly injected with saline, 100 ng peptide p[63-82] in saline or 100 ng scrambled peptide p[63-82] in saline (10 rats per group). All injection volumes were 50 µl. At four weeks post-MMT-surgery rats were sacrificed for histopathological scoring of the MMT knee joints. **A:** Change in body weight of the rats during the experiment (day 0-28). Conditions are indicated. **B:** Gait analysis scores at day 16 in the experiment **A/B**: Error bars represent mean ± SEM. **C:** Medial tibia cartilage degeneration score, Medial tibia depth ratio, Tibial cartilage degeneration width, Total medial tibia bone score, Medial femur cartilage degeneration score, Medial tibia osteophytes, Total joint score and Synovitis score are indicated in the table as mean (± SEM) for indicated conditions and percentage change of p[63-82] versus saline is presented in the last column. **D:** Collagen degeneration by severity, Collagen combined degeneration, Growth plate thickness and Medial collateral ligament thickness are indicated in the table as mean (± SEM) for indicated conditions and percentage change of p[63-82] versus saline is presented in the last column. Statistical differences were calculated between groups (Mann-Whitney U test) and * is $p<0.05$ ** is $p<0.01$, *** is $p<0.001$, and ns is not significant. Indicated scores are based on references: 1. Bendele, A.M., *Animal models of osteoarthritis.* J Musculoskelet Neuronal Interact, 2001. 1(4): p. 363-76. 2. Bendele, A.M., *Animal models of osteoarthritis in an era of molecular biology.* J Musculoskelet Neuronal Interact, 2002. 2(6): p. 501-3. 3. Gerwin, N., et al., *The OARSI histopathology initiative - recommendations for histological assessments of osteoarthritis in the rat.* Osteoarthritis Cartilage, 2010. 18 Suppl 3: p. S24-34.

Saline

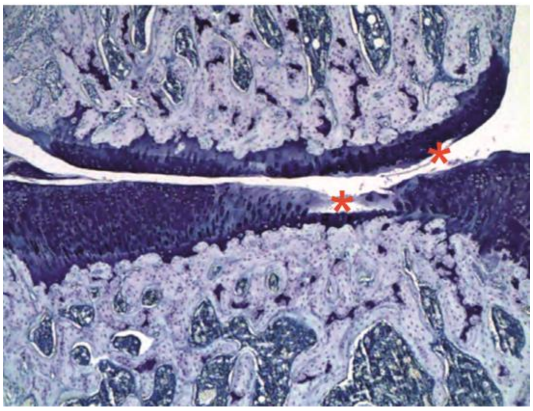

Supplementary Figure 6: Predominant cartilage lesion sites in the rat MMT experiment. Representative micrographs of toluidine blue-stained sections of medial aspects of the MMT knee joints. The predominant sites where lesions formed in this model in the tibial and femoral cartilage are indicated with an “*”.

SUPPLEMENTARY TABLES

Supplementary Table 1: Amino acid sequences of BMP7 peptide libraries

Peptide	Sequence	Peptide	Sequence
p[01-20]	H-STGSKQRSQNRSKTPKNQEA-OH	p[65-84]	H-YYSEGESAFPLNSYMNATNH-OH
p[03-22]	H-GSKQRSQNRSKTPKNQEALR-OH	p[67-86]	H-SEGESAFPLNSYMNATNHAI-OH
p[05-24]	H-KQRSQNRSKTPKNQEALRMA-OH	p[69-88]	H-GESAFPLNSYMNATNHAIVQ-OH
p[07-26]	H-RSQNRSKTPKNQEALRMANV-OH	p[71-90]	H-SAFPLNSYMNATNHAIVQTL-OH
p[09-28]	H-QNRSKTPKNQEALRMANVAE-OH	p[73-92]	H-FPLNSYMNATNHAIVQTLVH-OH
p[11-30]	H-RSKTPKNQEALRMANVAENS-OH	p[75-94]	H-LNSYMNATNHAIVQTLVHFI-OH
p[13-32]	H-KTPKNQEALRMANVAENSSS-OH	p[77-96]	H-SYMNATNHAIVQTLVHFINP-OH
p[15-34]	H-PKNQEALRMANVAENSSSDQ-OH	p[79-98]	H-MNATNHAIVQTLVHFINPET-OH
p[17-36]	H-NQEALRMANVAENSSSDQRQ-OH	p[81-100]	H-ATNHAIVQTLVHFINPETVP-OH
p[19-38]	H-EALRMANVAENSSSDQRQAS-OH	p[83-102]	H-NHAIVQTLVHFINPETVPKP-OH
p[21-40]	H-LRMANVAENSSSDQRQASKK-OH	p[85-104]	H-AIVQTLVHFINPETVPKPSS-OH
p[23-42]	H-MANVAENSSSDQRQASKKHE-OH	p[87-106]	H-VQTLVHFINPETVPKPSSAP-OH
p[25-44]	H-NVAENSSSDQRQASKKHELY-OH	p[89-108]	H-TLVHFINPETVPKPSSAPTQ-OH
p[27-46]	H-AENSSSDQRQASKKHELYVS-OH	p[91-110]	H-VHFINPETVPKPSSAPTQLN-OH
p[29-48]	H-NSSSDQRQASKKHELYVSFR-OH	p[93-112]	H-FINPETVPKPSSAPTQLNAI-OH
p[31-50]	H-SSDQRQASKKHELYVSFRDL-OH	p[95-114]	H-NPETVPKPSSAPTQLNAISV-OH
p[33-52]	H-DQRQASKKHELYVSFRDLGW-OH	p[97-116]	H-ETVPKPSSAPTQLNAISVLY-OH
p[35-54]	H-RQASKKHELYVSFRDLGWQD-OH	p[99-118]	H-VPKPSSAPTQLNAISVLYFD-OH
p[37-56]	H-ASKKHELYVSFRDLGWQDWI-OH	p[101-120]	H-KPSSAPTQLNAISVLYFDDS-OH
p[39-58]	H-KKHELYVSFRDLGWQDWIIA-OH	p[103-122]	H-SSAPTQLNAISVLYFDDSSN-OH
p[41-60]	H-HELYVSFRDLGWQDWIIAPE-OH	p[105-124]	H-APTQLNAISVLYFDDSSNVI-OH
p[43-62]	H-LYVSFRDLGWQDWIIAPEGY-OH	p[107-126]	H-TQLNAISVLYFDDSSNVILK-OH
p[45-64]	H-VSFRDLGWQDWIIAPEGYAA-OH	p[109-128]	H-LNAISVLYFDDSSNVILKKY-OH
p[47-66]	H-FRDLGWQDWIIAPEGYAAYY-OH	p[111-130]	H-AISVLYFDDSSNVILKKYRN-OH
p[49-68]	H-DLGWQDWIIAPEGYAAYYSE-OH	p[113-132]	H-SVLYFDDSSNVILKKYRNMV-OH
p[51-70]	H-GWQDWIIAPEGYAAYYSEGE-OH	p[115-134]	H-LYFDDSSNVILKKYRNMVVR-OH
p[53-72]	H-QDWIIAPEGYAAYYSEGESA-OH	p[117-136]	H-FDDSSNVILKKYRNMVVRAS-OH
p[55-74]	H-WIIAPEGYAAYYSEGESAFP-OH	p[119-138]	H-DSSNVILKKYRNMVVRASGS-OH
p[57-76]	H-IAPEGYAAYYSEGESAFPLN-OH	p[120-139]	H-SSNVILKKYRNMVVRASGSH-OH
p[59-78]	H-PEGYAAYYSEGESAFPLNSY-OH	p[63-82] scrambled	H-SEASPAMYYLNATANFYESG-OH
p[61-80]	H-GYAAYYSEGESAFPLNSYMN-OH	p[113-132] scrambled	H-VYISNVDDYSNKFLLKVRSM-OH
p[63-82]	H-AAYYSEGESAFPLNSYMNAT-OH		

Substitution position	Sequence	Substitution position	Sequence
Wildtype p[63-82]	H-AAYYSEGESAFPLNSYMNAT-OH	12	H-AAYYSEGESAFALNSYMNAT-OH
03	H-AAAYSEGESAFPLNSYMNAT-OH	13	H-AAYYSEGESAFPANSYMNAT-OH
04	H-AAYASEGESAFPLNSYMNAT-OH	14	H-AAYYSEGESAFPLASYMNAT-OH
05	H-AAYYAEGESAFPLNSYMNAT-OH	15	H-AAYYSEGESAFPLNAYMNAT-OH
06	H-AAYYSAGESAFPLNSYMNAT-OH	16	H-AAYYSEGESAFPLNSAMNAT-OH
07	H-AAYYSEAESAFPLNSYMNAT-OH	17	H-AAYYSEGESAFPLNSYANAT-OH
08	H-AAYYSEGASAFPLNSYMNAT-OH	18	H-AAYYSEGESAFPLNSYMAAT-OH
09	H-AAYYSEGEAAFPLNSYMNAT-OH	20	H-AAYYSEGESAFPLNSYMNAA-OH
11	H-AAYYSEGESAAPLNSYMNAT-OH		

Supplementary Table 2: siRNA sequences

mRNA	sense	antisense
NKX3-2	CCGAGACGCAGGUGAAAAUdTdT	AUUUUCACGUGCGUCUCGGdTdT

The oligonucleotide sequences are shown from 5’ to 3’. The 3’ termini were modified with two deoxythymidine nucleotides.

Supplementary Table 3: Oligonucleotide DNA sequences for RT-qPCR

(m)RNA	forward	reverse
28S rRNA	GCCATGGTAATCCTGCTCAGTAC	GCTCCTCAGCCAAGCACATAC
ADAMTS5	GTGGCTCACGAAATCGGACAT	GCGCTTATCTTCTGTGGAACCA
ALPL	AATGTCATCATGTTCCTGGGAGAT	TGGTGGAGCTGACCCTTGAG
NKX3-2	ACCTGGCAGCTTCGCTGAA	AGGTCGGCGGCCATCT
COL2A1	TGGGTGTTCTATTTATTTATTGTCTTCCT	GCGTTGGACTCACACCAGTTAGT
COL10A1	ATGATGAATACACCAAAGGCTACCT	ACGCACACCTGGTCATTTTCTG
COX-2	ACCAACATGATCTTTGCATTCTTT	GGTCCCCGCTTAAGATCTGTCT
IL6	TGTAGCCGCCCCACACA	GGATGTACCGAATTTGTTTGTCAA
MMP13	CTTCACGATGGCATTGCTGAC	CGCCATGCTCCTTAATTCCA
RUNX2	TGATGACACTGCCACCTCTGA	GCACCTGCCTGGCTCTTCT
SOX9	AGTACCCGCACCTGCACAAC	CGCTTCTCGCTCTCGTTCAG

DNA oligonucleotide sequences are shown from 5’ to 3’.

Supplementary Table 4: Medial Tibial and Femoral General Cartilage Degeneration Score

Score	Observation
0	No degeneration
0.5	Very minimal degeneration, within the zone less than 5% of the matrix has PG loss mainly with minor chondrocyte loss and little if any collagen matrix loss or damage
1	Minimal degeneration, within the zone 5-10% of the matrix appears non-viable as a result of significant chondrocyte loss (greater than 50% of normal cell density). PG loss is usually present in these areas of cell loss and collagen matrix loss may be present.
2	Mild degeneration, within the zone 11-25% of the matrix appears non-viable as a result of significant chondrocyte loss (greater than 50% of normal cell density). PG loss is usually present in these areas of cell loss and collagen matrix loss may be present.
3	Moderate degeneration, within the zone 26-50% of the matrix appears non-viable as a result of significant chondrocyte loss (greater than 50% of normal cell density). PG loss is usually present in these areas of cell loss and collagen matrix loss may be present.
4	Marked degeneration, within the zone 51-75% of the matrix appears non-viable as a result of significant chondrocyte loss (greater than 50% of normal cell density). PG loss is usually present in these areas of cell loss and collagen matrix loss may be present.
5	Severe degeneration, within the zone 76-100% of the matrix appears non-viable as a result of significant chondrocyte loss (greater than 50% of normal cell density). PG loss is usually present in these areas of cell loss and collagen matrix loss may be present.

General cartilage degeneration includes chondrocyte death/loss, proteoglycan (PG) loss, and collagen loss or fibrillation. Zones were scored individually according to the criteria in table and a sum of all three zones was calculated [1-3].

Supplementary Table 5: Medial Tibial and Femoral General Cartilage Degeneration Score

Medial Tibial Total Cartilage Degeneration Width	The width of the cartilage affected by any degeneration (cell loss, proteoglycan loss or collagen damage) was measured by ocular micrometer. This measurement extends from the origination of the osteophyte with adjacent cartilage degeneration (outside 1/3) across the surface to the point where tangential layer and underlying cartilage appear histologically normal [1-3].
Medial Tibial Substantial Cartilage Degeneration Width	Substantial Cartilage Degeneration was identified by chondrocyte and proteoglycan loss extending through greater than 50% of the cartilage thickness and was measured by ocular micrometer. In general, the collagen damage is mild (25% depth) or greater for this parameter but chondrocyte and proteoglycan loss extend to at least 50% or greater of the cartilage depth, indicating regions in which permanent structural changes have occurred [1-3].

Supplementary Table 6: Osteophyte Score and Measurement

**Score**	**Observation**	
0	None	Less than 200 μm
1	Small	200-299 μm
2	Medium	300-399 μm
3	Large	400-499 μm
4	Very large	500-599 μm
5	Very large	≥600 μm

Osteophyte thickness (tidemark to furthest point extending toward synovium) was measured with an ocular micrometer. Scores were assigned to the largest osteophyte in each section (typically found on the tibia) according to the criteria in the table [1-3].

Supplementary Table 7: Medial Tibial Bone Sclerosis Score

**Score**	**Observation**	
0	Normal	No observable difference in subchondral or epiphyseal trabecular bone thickness in medial vs. lateral.
1	Minimal	5-10% increase in subchondral or epiphyseal trabecular bone thickness in medial vs. lateral.
2	Mild	11-25% increase in subchondral or epiphyseal trabecular bone thickness in medial vs. lateral
3	Moderate	26-50% increase in subchondral or epiphyseal trabecular bone thickness in medial vs. lateral, obvious reduction in marrow spaces in outer 3/4 of medial tibia
4	Marked	51-75% increase in subchondral or epiphyseal trabecular bone thickness in medial vs. lateral, generally has very little marrow space in outer 3/4 of medial tibia, marrow spaces remain adjacent to cruciates.
5	Servere	76-100% increase in subchondral or epiphyseal trabecular bone thickness in medial vs. lateral, generally has very little marrow space remains in medial tibia

Medial tibial subchondral/epiphyseal bone thickening/sclerosis was scored based on the criteria in the table using a comparison to the lateral tibia and/or normal left medial tibias [1-3].

Supplementary Table 8: Total Joint Scores

The three-zone sums of the tibial and femoral cartilage degeneration scores, and the osteophyte score were summed to determine a total joint score. A sum of tibial cartilage degeneration and osteophyte scores without the femur was also calculated [1-3]

Supplementary Table 9: Synovitis Score

Score	Observation
0	Normal synovium
0.5	Very minimal synovitis (generally focal or scattered minimal diffuse).
1	Minimal synovitis (generally focal or scattered minimal diffuse).
2	Mild synovitis (multifocal to confluent areas of mild mononuclear cell infiltration).
3	Moderate synovitis (confluent areas of moderate mononuclear cell infiltration).
4	Marked synovitis (confluent areas of marked mononuclear cell infiltration).
5	Severe synovitis (confluent areas of severe mononuclear cell infiltration).

Synovial inflammation (mainly mononuclear cell infiltration concentrated on the medial side) was scored as indicated in Table 9 [1-3].

Supplementary Table 10: Medial Tibial Collagen Degeneration Severity

Any Damage	Fibrillation ranging from superficial to full thickness loss.
Severe Damage	Total or near total loss of collagen to tidemark, >90% thickness.
Marked Damage	Extends through 61-90% of the cartilage thickness.
Moderate Damage	Extends through 31-60% of the cartilage thickness.
Mild Damage	Extends through 11-30% of the cartilage thickness.
Minimal Damage	Very superficial, affecting upper 10% only.

Collagen damage across the medial tibial plateau (most severely affected section of the two halves) was quantified by measuring the total width of the collagen degeneration severities indicated in the table using an ocular micrometer. Measurements were expressed as a percentage of the total tibial surface width [1-3].

Supplementary Table 11: Growth Plate Thickness

Growth plate thickness was measured in all knees on medial and lateral sides (2 measurements per joint) at the approximate midpoint of the medial and lateral physis (assuming a non-tangential area of the section) using an ocular micrometer. The lateral thickness was also subtracted from the medial to determine the difference between the two [1-3].

Supplementary Table 12: Medial Collateral Ligament/Synovial Repair

Measurements were made of the thickness of the medial synovial/collateral ligament repair in a non-tangential area of the section using an ocular micrometer [1-3]

Supplementary Table 13: Gait analysis

Score	Observation
0	Normal, approximately equal ink staining to normal paw.
1	Slight limp/pain. Reduced inking area relative to the normal paw, but no full regions or structures are missing.
2	Mild limp/pain. Print extends to the end or near to the end of the “curlicue” structure. If normal paw has very little heel staining (rat walks mainly on toes/ball of foot), then slightly less staining.
3	Moderate limp/pain. Toes and full ball of foot, extending to the top of the “curlicue” structure are present. If normal paw has very little heel staining (rat walks mainly on toes/ball of foot), then toes with small portion of ball of foot.
4	Marked limp/pain. Toes and partial ball of foot, no heel or posterior foot. If normal paw has very little heel staining (rat walks mainly on toes/ball of foot), then toes only.
5	Severe limp/pain. Toes only, no ball of foot, no heel. If normal paw has very little heel staining (rat walks mainly on toes/ball of foot), then partial toes or non-specific marks.
6	Hopping. Carrying leg, no footprint is evident.

Gait analysis was performed on day 16 to confirm expected animal mobility post-surgery. Gait was evaluated by applying ink to the ventral surface of the foot and documenting weight bearing during movement (footprints) across paper. Rear feet of rats were placed in ink, then rats were placed on paper and allowed to walk the full length. This process was repeated as necessary to generate 4 clear, evenly inked footprint pairs representing the overall pattern of gait. Gait was scored visually as described in the table (descriptions refer to diseased leg) [1-3].

References

1. Bendele, A.M., *Animal models of osteoarthritis.* J Musculoskelet Neuronal Interact, 2001. **1**(4): p. 363-76.
2. Bendele, A.M., *Animal models of osteoarthritis in an era of molecular biology.* J Musculoskelet Neuronal Interact, 2002. **2**(6): p. 501-3.
3. Gerwin, N., et al., *The OARSI histopathology initiative - recommendations for histological assessments of osteoarthritis in the rat.* Osteoarthritis Cartilage, 2010. **18 Suppl 3**: p. S24-34.
