# Supplementary material for: Discovery of bone morphogenetic protein 7-derived peptide sequences that attenuate the human osteoarthritic chondrocyte phenotype
Source: Mol Ther Methods Clin Dev. 2021 Mar 17;21:247–61. doi: 10.1016/j.omtm.2021.03.009 (PMC8022858; doi:10.1016/j.omtm.2021.03.009)
Supplement: Document S2. Article plus supplemental information [file mmc2.pdf]

Molecular Therapy
Methods & Clinical Development
Original Article

Discovery of bone morphogenetic protein 7-derived peptide sequences that attenuate the human osteoarthritic chondrocyte phenotype

Marjolein M.J. Caron,[1] Ellen G.J. Ripmeester,[1] Guus van den Akker,[1] Nina K.A. P. Wijnands,[1] Jessica Steijns,[1] Don A.M. Surtel,[1] Andy Cremers,[1] Pieter J. Emans,[1,2] Lodewijk W. van Rhijn,[1,2] and Tim J.M. Welting[1,2]

[1]Laboratory for Experimental Orthopedics, Department of Orthopedic Surgery, Maastricht University, Universiteitssingel 50, 6229 ER Maastricht, the Netherlands; [2]Laboratory for Experimental Orthopedics, Department of Orthopedic Surgery, Maastricht University Medical Center, P.O. Box 5800, 6202 AZ Maastricht, the Netherlands

Treatment of osteoarthritis (OA) is mainly symptomatic by alleviating pain to postpone total joint replacement. Bone morphogenetic protein 7 (BMP7) is a candidate morphogen for experimental OA treatment that favorably alters the chondrocyte and cartilage phenotype. Intra-articular delivery and sustained release of a recombinant growth factor for treating OA are challenging, whereas the use of peptide technology potentially circumvents many of these challenges. In this study, we screened a high-resolution BMP7 peptide library and discovered several overlapping peptide sequences from two regions in BMP7 with nanomolar bioactivity that attenuated the pathological OA chondrocyte phenotype. A single exposure of OA chondrocytes to peptides p[63–82] and p[113–132] ameliorated the OA chondrocyte phenotype for up to 8 days, and peptides were bioactive on chondrocytes in OA synovial fluid. Peptides p[63–82] and p[113–132] required NKX3-2 for their bioactivity on chondrocytes and provoke changes in SMAD signaling activity. The bioactivity of p[63–82] depended on specific evolutionary conserved sequence elements common to BMP family members. Intra-articular injection of a rat medial meniscal tear (MMT) model with peptide p[63–82] attenuated cartilage degeneration. Together, this study identified two regions in BMP7 from which bioactive peptides are able to attenuate the OA chondrocyte phenotype. These BMP7-derived peptides provide potential novel disease-modifying treatment options for OA.

INTRODUCTION

Osteoarthritis (OA) is the most common degenerative joint disorder worldwide and presents with degradation of articular cartilage, leading to loss of joint mobility and function, accompanied by chronic pain.[1] Biochemically, OA is characterized by uncontrolled synthesis of extracellular matrix-degrading enzymes, such as aggrecanases (a disintegrin and metalloprotease with thrombospondin motifs [ADAMTSs]) and matrix metalloproteinases (MMPs), resulting in the active breakdown of the cartilage tissue matrix.[2] Important risk factors for developing OA are joint overloading (misalignment, obesity, work related); diabetes; aging; articular cartilage damage due to, e.g., trauma;[3] and others. The analogy between endochondral ossification and OA progression has been widely recognized, and many of the cartilage-degrading enzymes that are secreted by hypertrophic chondrocytes in the growth plate are also involved in OA development.[4–6] This places, next to the local joint inflammatory condition,[7] the chondrocyte/cartilage differentiation status central in the progression or even cause of OA.[8] Current treatments for OA are mainly symptomatic by alleviating pain and interfering with the cartilage-degenerative processes to postpone total joint replacement.

A number of growth factor-based treatment options for OA are currently under investigation.[9] One growth factor that modifies the chondrocyte and cartilage differentiation status is bone morphogenetic protein 7 (BMP7; also called osteogenic protein 1 [OP-1]). Studies addressing the disease-modifying properties of BMP7 showed that it decreases MMP13 expression in interleukin (IL)-1β-exposed chondrocytes,[10] stimulates proteoglycan synthesis in OA chondrocytes,[11] counteracts inflammatory cytokines (e.g., IL-1β), and induces an overall anabolic response in healthy chondrocytes.[12,13] Intra-articular (IA) administration of BMP7 protects against OA development in rabbits[14] and delays the progression of OA in rats.[15] A phase 1 clinical trial has been completed for BMP7 in end-stage OA patients and reported no serious adverse events after the intra-articular injection of BMP7.[16] In concert with these reports, our previous work[17] unveiled that BMP7 suppresses the pathological chondrocyte phenotype associated with OA[8] via NK3 Homeobox 2 (NKX3-2) and by inhibiting chondrocyte catabolism and hypertrophy.

Challenges that come with intra-articular delivery of a recombinant growth factor for treating OA are its formulation for sustained release from a drug carrier, stability in the hydrolytic and proteolytic OA

Received 28 November 2020; accepted 9 March 2021;
https://doi.org/10.1016/j.omtm.2021.03.009.

Correspondence: Tim J. M. Welting, PhD, Professor of Molecular Cartilage Biology, Laboratory for Experimental Orthopedics, Department of Orthopedic Surgery, Maastricht University Medical Center, P.O. Box 5800, 6202 AZ Maastricht, the Netherlands.
E-mail: t.welting@maastrichtuniversity.nl

This is an open access article under the CC BY license (http://creativecommons.org/licenses/by/4.0/).

synovial fluid (SF) environment,[18–20] and high production costs of the recombinant growth factor. Mimicking the growth factor-initiated OA chondrocyte biological responses with the use of peptides is a promising area that was recently applied to growth hormone/somatostatin, preventing cartilage degradation in a rat model for OA.[21] The biomolecular synthesis of short linear peptides is relatively straightforward, and in general, peptides are less susceptible to conformational inactivation as compared to recombinant growth factors. Consequently, the use of peptide technology potentially circumvents many of the challenges associated with full-length recombinant growth factors.[22] Peptides from BMP7 have previously been reported to support osteogenesis[23–25] and prevent fibrosis in acute and chronic kidney injury.[26] Whether peptides derived from BMP7 are able to suppress the pathological OA chondrocyte phenotype[27] was unknown. Although bioactive peptides from BMP7 have been reported, the action of BMPs and BMP-derived peptides is highly cell-type dependent.[28] Therefore, a screening was performed of an overlapping sequential BMP7 peptide library to discover peptide sequences with a bioactivity that specifically attenuates the pathological OA chondrocyte phenotype. We postulate that the potential identification of a peptide from BMP7 will hold the future promise to be more compatible, and biochemically modifiable, for incorporation into macromolecular sustained release systems that may potentially be used in the intra-articular treatment of OA.

RESULTS

Peptide sequences derived from BMP7 that attenuate the OA chondrocyte phenotype

To investigate whether BMP7 harbors potential peptide sequences that improve the OA chondrocyte phenotype, a peptide library was designed from the mature 139 amino acid-long human BMP7 sequence (Table S2). The peptide library was designed as 20-mer peptides with 2 amino acid intervals (18 amino acid overlap) between individual peptides and with all cysteine residues substituted by serine residues to avoid uncontrolled oxidation of cysteine groups. This yielded 61 individual peptides covering the complete mature human BMP7 sequence (Figure 1A). The peptide library was screened using a pool of primary human OA articular chondrocytes (OA-HACs) from 18 individual donors. Prior to pooling, individual OA-HAC isolates were tested for their responsiveness to BMP7 (Figure S1). The screening was conducted by exposing the OA-HAC pool for 24 h to a concentration series (1, 10, 100, or 1,000 nM) of each peptide from the library. The chondrocyte response to each condition was established by measuring a set of OA chondrocyte phenotype genes in which their expression was previously shown to significantly improve by BMP7 treatment.[27] This is defined as reduced expression of collagen type X alpha 1 chain (*COL10A1*), alkaline phosphatase (*ALPL*), Runt-related transcription factor 2 (*RUNX2*), *ADAMTS5*, *MMP13*, prostaglandin-endoperoxide synthase 2 or cyclooxygenase-2 (*COX-2*), and interleukin 6 (*IL6*) and increased expression of SRY-box transcription factor 9 (*SOX9*), *COL2A1*, and *NKX3-2*. Library screening data of the 100-nM condition for *COL10A1* expression are presented in Figure 1B (for other genes and peptide concentrations, see Figure S2) and show that the majority of the peptides worsened the chondrocyte phenotype and provoked chondrocyte hypertrophy (*COL10A1* expression). However, peptides derived from the central region of BMP7, as well as from the C terminus, exhibited bioactivity that improved the expression of the predefined OA chondrocyte phenotype gene set. Representative peptides inducing the above gene expression characteristics in OA-HACs were selected from both regions for further investigation. These were peptide p[63−82] and p[113−132] from the library, covering amino acids 63−82 and 113−132 of the mature BMP7 amino acid sequence. The complete quantitative real-time PCR data for peptides p[63−82] and p[113−132] (Figure 2) show that these peptides induced expression of *SOX9*, *COL2A1*, and *NKX3-2* (Figure 2A) and inhibited expression of *RUNX2*, *COL10A1*, *ALPL*, *MMP13*, *ADAMTS5*, *COX-2*, and *IL6* (Figures 2B and 2C). It is noteworthy that these peptides exhibited bioactivity in the low nanomolar range. For the two candidate peptides, we validated data using an independent cohort of three OA-HACs exposed to 100 nM of p[63−82] or p[113−132] or a combination of both peptides. Gene expression analyses of *COL10A1*, *ALPL*, *RUNX2*, *COL2A1*, *SOX9*, *NKX3-2*, *ADAMTS5*, *MMP13*, *COX-2*, and *IL6* expression showed similar responses as above (Figure S3). Peptide p[63−82] increased the sulfated glycosaminoglycan (sGAG) content of OA-HAC cultures (Figure 2D), and both peptides caused a significant decrease in alkaline phosphatase (ALP) activity (Figure 2E) and prostaglandin E_2 (PGE_2) levels (Figure 2F) in these cultures. To investigate sequence specificity of the bioactivity of peptides p[63−82] and p[113−132], scrambled version of these peptides were tested, and neither one of them was able to recapitulate the bioactivity that was observed for candidate peptides p[63−82] and p[113−132] on the expression of *COL2A1*, *RUNX2*, *COL10A1*, and *COX-2* (Figure 2G). Taken together, two regions in BMP7 were identified delivering peptide sequences that are able to improve the OA chondrocyte phenotype.

BMP7-derived peptides retain bioactivity on HACs cultured in OA SF

To further corroborate the potential OA-protective properties of peptides p[63−82] and p[113−132], we mimicked the OA environment by exposing non-OA-HACs to OA SF. Culturing non-OA-HACs in the presence of OA SF reduced their *SOX9*, *COL2A1*, and *NKX3-2* expression (Figure 3A), whereas expression of *RUNX2*, *COL10A1*, *ALPL*, *MMP13*, *ADAMTS5*, *COX-2*, and *IL6* was induced by OA SF (Figures 3B and 3C). This was accompanied by increased ALP activity and PGE_2 secretion (Figures 3E and 3F). The decline of *SOX9* and *COL2A1* expression was counteracted to some extent by peptides p[63−82] and p[113−132] (Figure 3A), and expression of *NKX3-2* was induced by the peptides to levels higher than in the control condition (Figure 3A). The OA SF-induced expression of *RUNX2*, *COL10A1*, *ALPL*, *MMP13*, *ADAMTS5*, *COX-2*, and *IL6* was, without exception, mitigated by peptides p[63−82] and p[113−132] (Figures 3B and 3C). sGAG content of HAC cultures was unaltered by the peptides (Figure 3D). Retainment of peptide p[63−82] and p[113−132] bioactivity on HACs in an OA SF environment was also confirmed for ALP activity (Figure 3E) and PGE_2 secretion (Figure 3F). Data demonstrate that bioactivity of peptide p[63−82] and p[113−132] on HACs is to a certain extent compatible with OA SF.

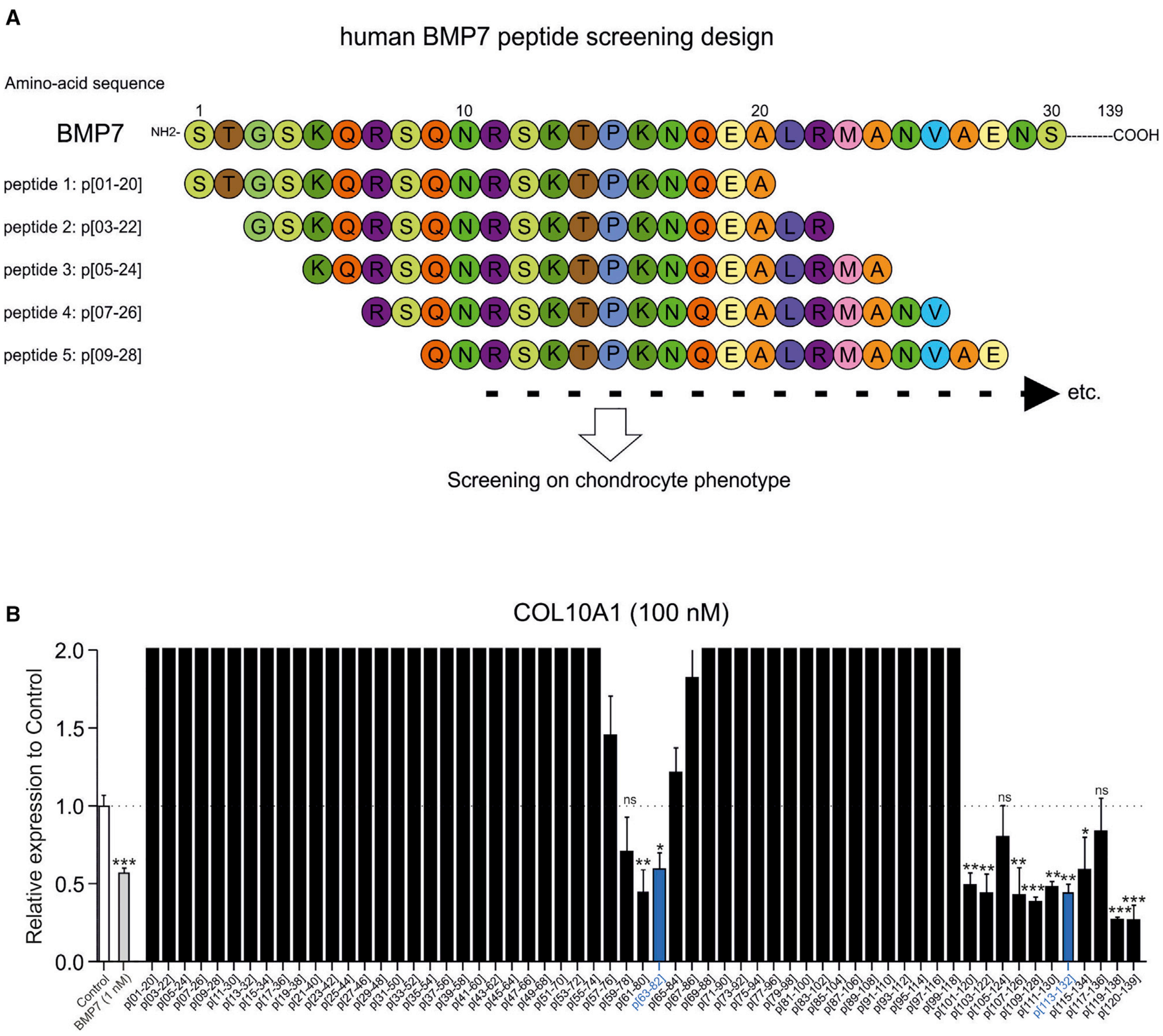

Figure 1. BMP7 peptide library screening on primary human OA articular chondrocytes (OA-HACs)

(A) Schematic representation of the design of the human BMP7 peptide library. (B) COL10A1 mRNA expression in samples from the library screening on a pool of 18 OA-HACs individually exposed for 24 h to the peptides (100 nM) in the library. Data are shown as compared to the control condition (white bar) and full-length BMP7 (1 nM; gray bar). The two blue bars represent candidate peptides p[63–82] and p[113–132]. Data from these two peptides showed significantly downregulated COL10A1 expression but also showed reduced expression of RUNX2, ALPL, COX-2, IL-6, ADAMTS5, and MMP13 and increased expression of SOX9, COL2A1, and NKX3-2 in all tested peptide concentrations. Error bars represent mean ± SEM, and statistical significance for peptide condition or BMP7 versus control condition as determined by unpaired two-tailed Student's t test is represented as *p < 0.05, **p < 0.01, and ***p < 0.001; NS, not significant. Data for other measured mRNAs and peptide concentrations are provided in Figure S2.

Duration of chondrocyte phenotype modulation by BMP7-derived peptides

The duration of chondrocyte phenotypic change induced by candidate peptides p[63–82] and p[113–132] was determined by exposing OA-HACs to a single dose of peptide or continuously keeping the peptides present during OA-HAC culture. In both cases, the phenotype of the OA-HACs was analyzed every other day during a 10-day follow-up. Non-OA-HACs were used as a reference for chondrocyte phenotype status. Continuous exposure of OA-HACs to peptide p[63–82] or p[113–132] by repeated addition of the peptide to the culture media every other day resulted in an overtime increasing mRNA expression of *COL2A1* levels (Figure 4A). A reciprocal response was observed for *COL10A1* gene expression, which steadily decreased over time (Figure 4B). Inhibition of *MMP13* and *COX-2*

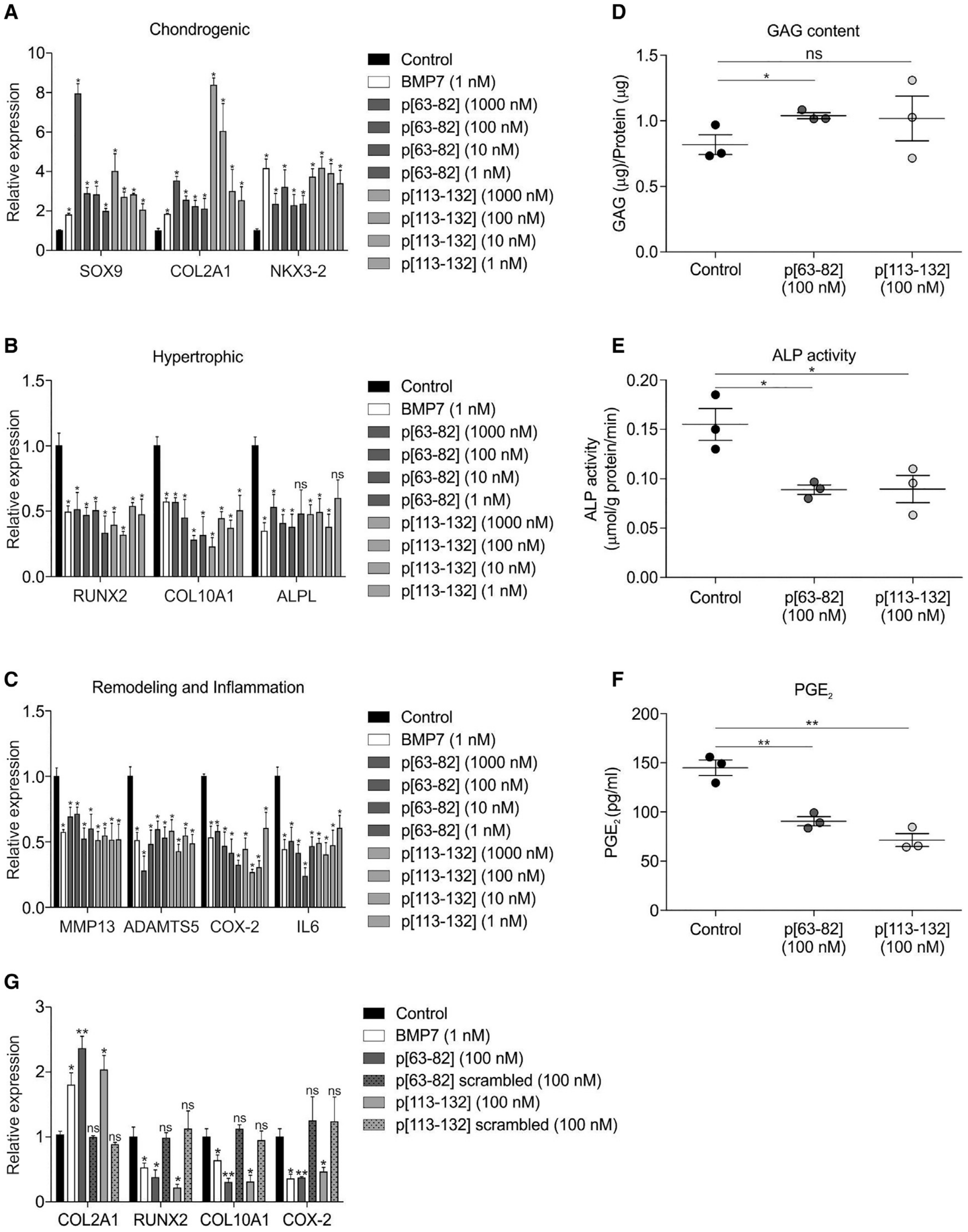

Figure 2. Bioactivity of BMP7-derived peptides p[63–82] and p[113–132] on OA-HACs

Complete peptide library screening quantitative real-time PCR dataset for peptides p[63−82] and p[113−132] is presented. (A) SOX9, COL2A1, and NKX3-2 mRNA expression in the 18 OA-HAC pool exposed to 1,000, 100, 10, or 1 nM of peptide p[63−82] and p[113−132] is shown and presented relative to control (black bars) and alongside full-length BMP7 (white bars). (B) Similar to (A) but for chondrocyte hypertrophy-associated genes RUNX2, COL10A1, and ALPL. (C) Similar to (A) but for cartilage extracellular matrix remodeling-associated genes MMP13 and ADAMTS5 and inflammation-related genes COX-2 and IL-6. (D) Glycosaminoglycan (GAG) content (normalized to total protein content) on an independent cohort of three individual OA-HAC donors treated with 100 nM peptides p[63−82] and p[113−132] for 24 h.

(legend continued on next page)

expression was continuously observed during the treatment period (Figures 4C and 4D). Follow-up of the single-dose treatment of OA-HACs with either peptide p[63−82] or p[113−132] revealed inhibition of *COL10A1*, *MMP13*, and *COX-2* gene expression up to and including day 8, reaching similar levels of expression of these genes in non-OA-HACs. At day 10, expression of *COL10A1*, *MMP13*, and *COX-2* in the single-dose condition was indistinguishable from non-treated OA HACs. This timing was similar for *COL2A1*, although the action of peptide p[63−82] was not detectable anymore from day 8 onward. Together, data show that a single exposure of OA-HACs to peptides p[63−82] or p[113−132] leads to detectable changes in the chondrocyte gene expression phenotype up to 8 days.

Molecular characterization of peptide bioactivity

A chondrocyte phenotypic consequence of exposure to peptides p[63−82] or p[113−132] is an overall attenuation of hypertrophy. Previously, we[17,27] and others[29,30] recognized NKX3-2 (BAPX1) as an important negative regulator of chondrocyte hypertrophy, and the hypertrophy-inhibiting action of BMP7 on OA-HACs is, at least in part, mediated via NKX3-2.[27] Taking into consideration that the ability to induce *NKX3-2* expression was one of the screening criteria for the identification of the here-described, BMP7-derived peptides (Figures 2 and S2), we next investigated whether NKX3-2 is involved in the bioactivity of peptides p[63−82] and p[113−132]. By means of small interfering (si)RNA transfection, NKX3-2 expression was reduced in a pool of OA-HACs. Knockdown of NKX3-2 expression was confirmed (Figure 5A), and in concert with our previous findings,[27] expression of chondrocyte hypertrophy-associated genes was sharply induced (Figure 5A). Treatment of control conditions (scrambled siRNA) with peptide p[63−82] or p[113−132]confirmed the peptides' chondrocyte hypertrophy-inhibiting bioactivity (Figure 5A). OA-HACs with NKX3-2 knockdown and treated with either peptide p[63−82] or p[113−132] did not show rescue of the hypertrophy-inducing consequences of abrogated NKX3-2 expression (Figure 5A). This suggests that the chondrocyte hypertrophy-inhibiting bioactivity of these peptides is NKX3-2 dependent.

Since peptides p[63−82] and p[113−132] are BMP7 derived, and transcription-repressing activity of NKX3-2 depends on BMP signaling,[31] we next determined whether these peptides can modulate SMAD (small mothers against decapentaplegic) transcriptional activity. Activity of the BRE reporter (SMAD1/5/8 dependent[32]) and the CAGA12 reporter (SMAD3 dependent[33]) was measured in SW1353 chondrocytes under influence of either one of the peptides. Data showed that both peptides were able to induce the activity of the BRE reporter, whereas CAGA12-reporter activity was diminished (Figure 5B). This indicates that BMP7-derived peptides p[63−82] and p[113−132] can alter SMAD signaling activity in chondrocytes.

We aimed to further understand the sequence dependency of the peptide bioactivity. Peptide p[63−82] was derived from the central region within BMP7, and since this yielded a limited series of bioactive peptides (Figures 1B and S2), we further focused our analyses on peptide p[63−82]. To determine which amino acids are important for the bioactivity of peptide p[63−82], an alanine-scanning peptide library was synthesized, based on systematic alanine substitution of consecutive amino acids in the peptide p[63−82] sequence (Table S1). This alanine-scanning library was then screened by exposing an OA-HAC pool (the same pool that was used for the full library scanning in Figure 1) for 24 h to 100 nM of each peptide in the alanine-scanning library. The bioactivity of each peptide was determined by measuring gene expression of *COL10A1*, *ALPL*, *MMP13*, *COL2A1*, and *NKX3-2* (Figure S4). Changes in the bioactivity of the alanine-scanning library peptides were combined into a "bioactivity score" of each individual substituted amino acid in the parental p[63−82] peptide (Figure 5C). From the bioactivity score, it became evident that Y3A, G7A, E8A, F11A, Y16A, and M17A represent alanine substitutions with an important impact on peptide p[63−82] bioactivity. From these substitutions, G7A had the largest impact on the chondrocyte phenotype-modulating action of the peptide, with a reciprocal response on chondrocyte hypertrophy (Figure S4). The region in BMP7 from which peptide p[63−82] was derived and the G7 position in it are evolutionary highly conserved, as determined by sequence alignment of BMP7 sequences from various animal species (Figure 5D).

Collectively, these data demonstrate that NKX3-2 is important for the bioactivity of peptides p[63−82] and p[113−132] on HACs and that the peptides led to the activation of BRE- and inhibition of CAGA12-reporter activity in chondrocytic cells. The identity of specific amino acids with the p[63−82] peptide sequence is important for its bioactivity, with the glycine (G) residue at position 7 playing an important role in p[63−82] peptide bioactivity on HACs.

Peptide activity on OA knee joint tissues

To determine whether peptide p[63−82] has the potency to alter chondrocyte behavior in cartilage tissue, rather than isolated HACs, cartilage biopsies from OA knees were cultured in the presence of peptide p[63−82], alongside its scrambled version and full-length BMP7. In agreement with our observations in isolated OA-HACs, we found that peptide p[63−82] was specifically able to induce *COL2A1* while inhibiting *COL10A1* and *COX-2* expression in cartilage explants (Figure 6A). This action was comparable to full-length recombinant BMP7. The scrambled version of peptide p[63−82] lacked this activity. Treatment of cartilage explants with BMP7 or peptide p[63−82] had no inhibitory effect on sGAG release (Figure 6B).

Corresponding gene expression data are shown in Figure S3. (E) Alkaline phosphatase (ALP) activity (normalized to total protein content) in similar samples from (D). (F) Prostaglandin E_2 (PGE_2) levels in culture supernatant in similar samples from (D). (G) In the same OA-HAC pool of 18 OA-HAC donors from the peptide library screening, sequence dependency of peptides p[63−82] and p[113−132] was determined by exposing the OA-HAC pool to scrambled versions of peptides p[63−82] and p[113−132] (100 nM, 24 h). Expression of COL2A1, RUNX2, COL10A1, and COX-2 mRNAs is shown. Error bars represent mean ± SEM, and statistical significance for peptide conditions or BMP7 versus control condition as determined by unpaired two-tailed Student's t test is represented as *$p < 0.05$, **$p < 0.01$, and ***$p < 0.001$.

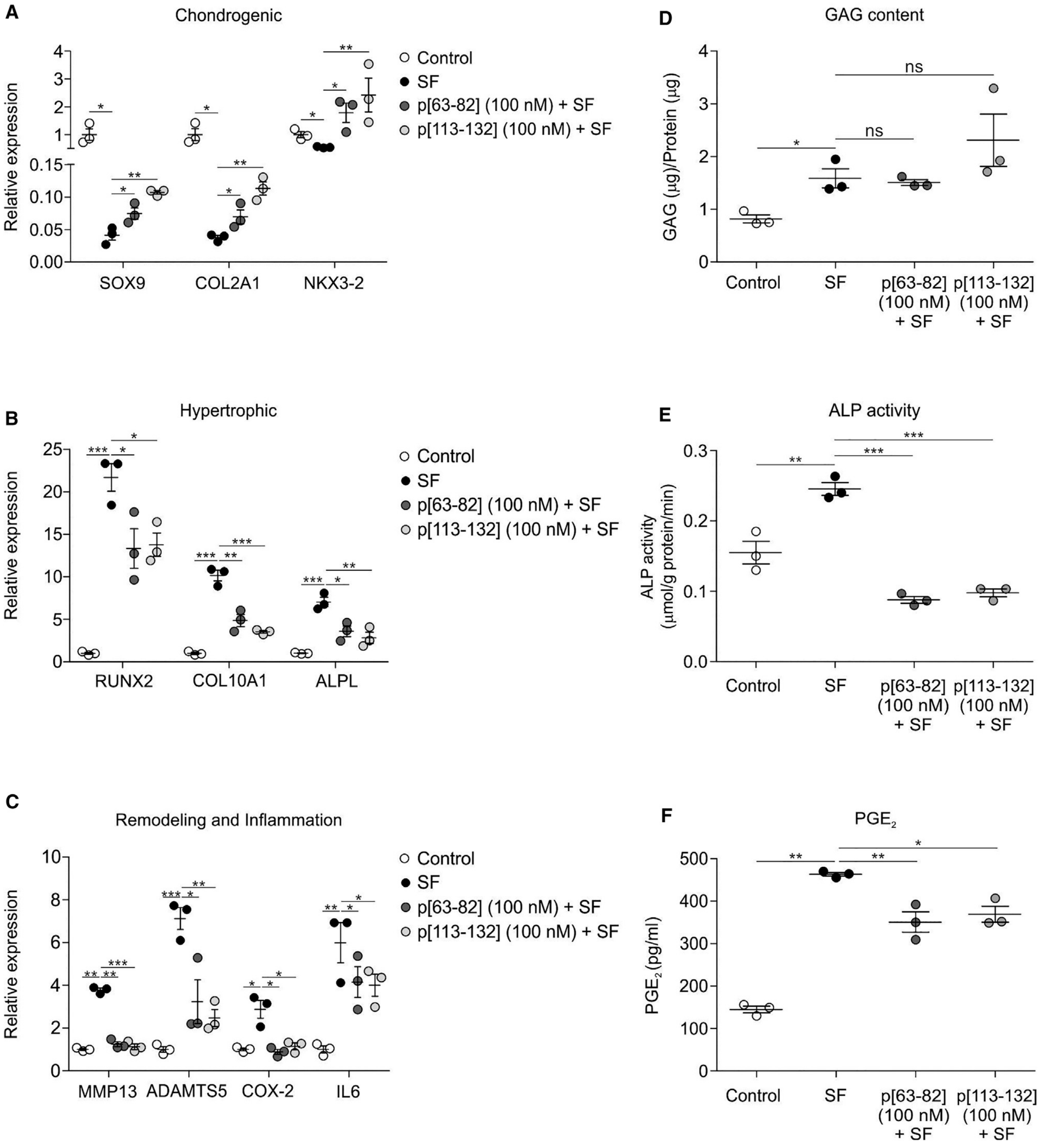

Figure 3. Bioactivity of peptides p[63–82] and p[113–132] on OA articular chondrocytes in OA synovial fluid (SF)

OA-HACs (n = 3 individual donors, tested in triplicate) were exposed to 100 nM peptide p[63−82] or p[113−132] in the presence of OA SF (20% [v/v]; equal ratio mix from five individual donors). Control is 20% (v/v) 0.9% NaCl. Cells were cultured in these conditions for 24 h and analyzed for mRNA expression of indicated genes (normalized for 28S rRNA expression and relative to control condition). (A) Expression of SOX9, COL2A1, and NKX3-2 mRNAs. (B) Expression of RUNX2, COL10A1, and ALPL mRNAs. (C) Expression of MMP13, ADAMTS5, COX-2, and IL-6 mRNAs. (D) GAG content (normalized to total protein content) was determined in the same conditions. (E) ALP enzyme activity in cell lysates of the same conditions was determined and normalized for total protein content. (F) PGE_2 levels in culture supernatants of the same conditions. Error bars represent mean ± SEM, and statistical significance for peptide conditions or control versus SF condition as determined by unpaired two-tailed Student's t test is represented as *p < 0.05, **p < 0.01, and ***p < 0.001.

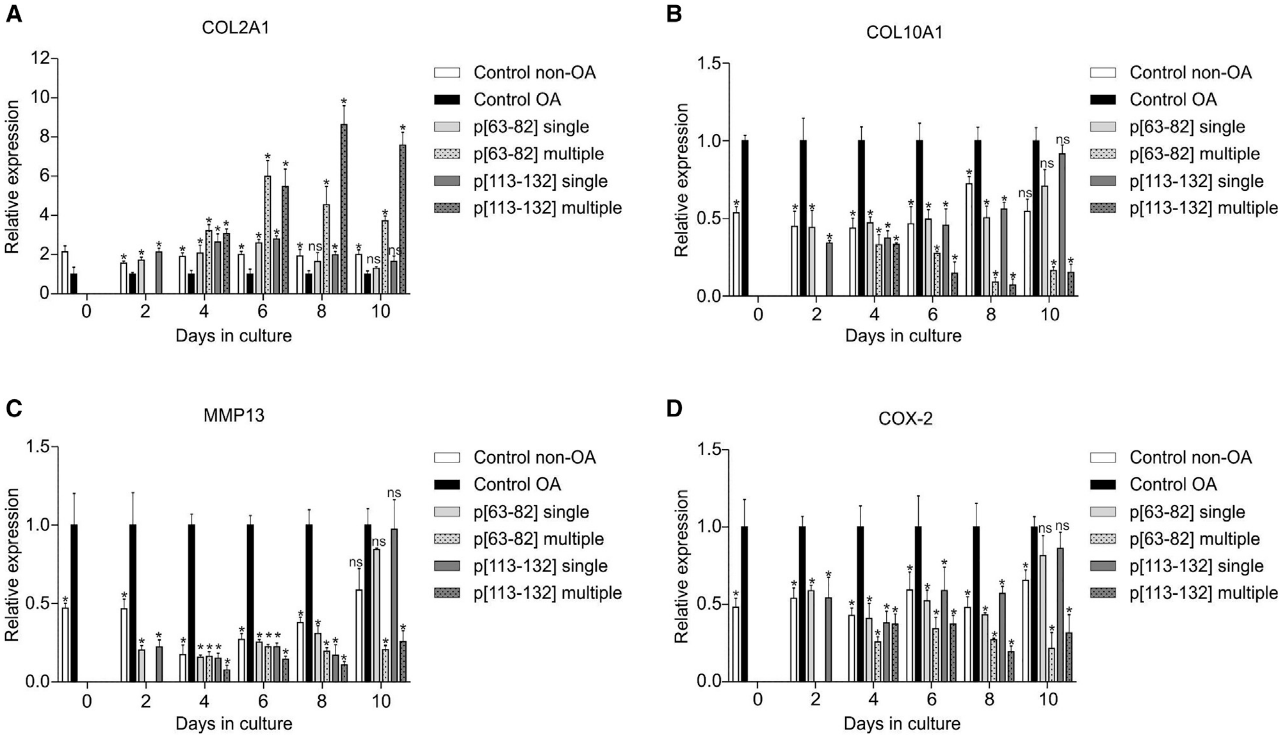

Figure 4. Duration of chondrocyte phenotype modulation by BMP7-derived peptides

A pool of OA-HACs (n = 3 donors) was cultured in the presence or absence of 100 nM peptides p[63–82] or p[113–132] and compared to a pool of human articular chondrocytes from a non-OA source. The medium was changed every 2 days. Peptides were added at the start of the experiment and subsequently at every medium change (multiple) or only at the start of the experiment and not during every subsequent medium change (single). At days 0, 2, 4, 6, 8, and 10 in culture, samples were harvested and analyzed for the expression of the indicated mRNAs by quantitative real-time PCR (normalized for 28S rRNA expression) (A) COL2A1 mRNA expression. (B) COL10A1 mRNA expression. (C) MMP13 mRNA expression. (D) COX-2 mRNA expression. In graphs, error bars represent mean ± SEM; data are presented relative to the "control OA" condition of each time point. Statistical differences were calculated to control conditions. Error bars represent mean ± SEM, and statistical significance for peptide conditions or control non-OA versus control OA condition as determined by unpaired two-tailed Student's t test is represented as *p < 0.05, **p < 0.01, and ***p < 0.001.

Intra-articularly administered molecules for the treatment of OA are expected to get in contact with multiple intra-articular tissues. In this context, it should be considered that besides a chondrocyte hypertrophy-modulating bioactivity, peptide p[63–82] also influences the inflammatory behavior of OA-HACs (Figures 2, 3, and 4). Therefore, we next tested the anti-inflammatory potential of peptide p[63–82] on cartilage, synovium, infra-patellar fat pad (IPFP), and meniscus explants from knee OA patients. PGE_2 levels in culture supernatants of synovial explants were not significantly changed by the peptide nor by BMP7 (Figure 6C). However, PGE_2 levels from cartilage, IPFP, and meniscus were reduced by peptide p[63–82] and to a similar extent as by BMP7 (Figures 6D–6F). Together, these data indicate that peptide p[63–82] is able to alter the phenotype of the chondrocyte in cartilage tissue and *ex vivo* reduces the inflammatory status of knee OA tissues by reducing PGE_2 levels.

Attenuation of medial meniscal tear (MMT)-induced cartilage damage

We next tested whether peptide p[63–82] has the potency to delay the progression of trauma-induced cartilage degeneration in the rat MMT model.[34,35] MMT damage was unilaterally initiated. Starting at 1 week post-MMT surgery, rats were two times per week intra-articularly injected with saline, 100 ng peptide p[63–82] in saline, or 100 ng scrambled peptide p[63–82] in saline. At 4 weeks post-MMT surgery, rats were sacrificed for histopathological scoring of the MMT knee joints. Visual assessment of frontal sections of the knee joints demonstrated the loss of toluidine blue staining and chondrocyte death, as well as frequent development of a dominant full-thickness cartilage defect in the lateral compartment of the medial tibia plateau in the saline-injected group (Figure 7A, left panel). Histology of the peptide p[63–82]-injected group revealed a certain loss of toluidine blue-staining intensity and decreased articular chondrocyte cellularity in the lateral aspect of the medial tibia plateau. However, cartilage-degenerative changes were less apparent in the p[63–82] group (Figure 7A, middle panel). In contrast, histology of the group injected with scrambled peptide p[63–82] was characterized by diminished toluidine blue staining and lower chondrocyte cellularity at the lateral side of the medial tibia plateau, as well as cartilage degeneration (Figure 7A, right panel). Histopathology scoring[35] revealed degenerative changes of the medial tibia articular cartilage in

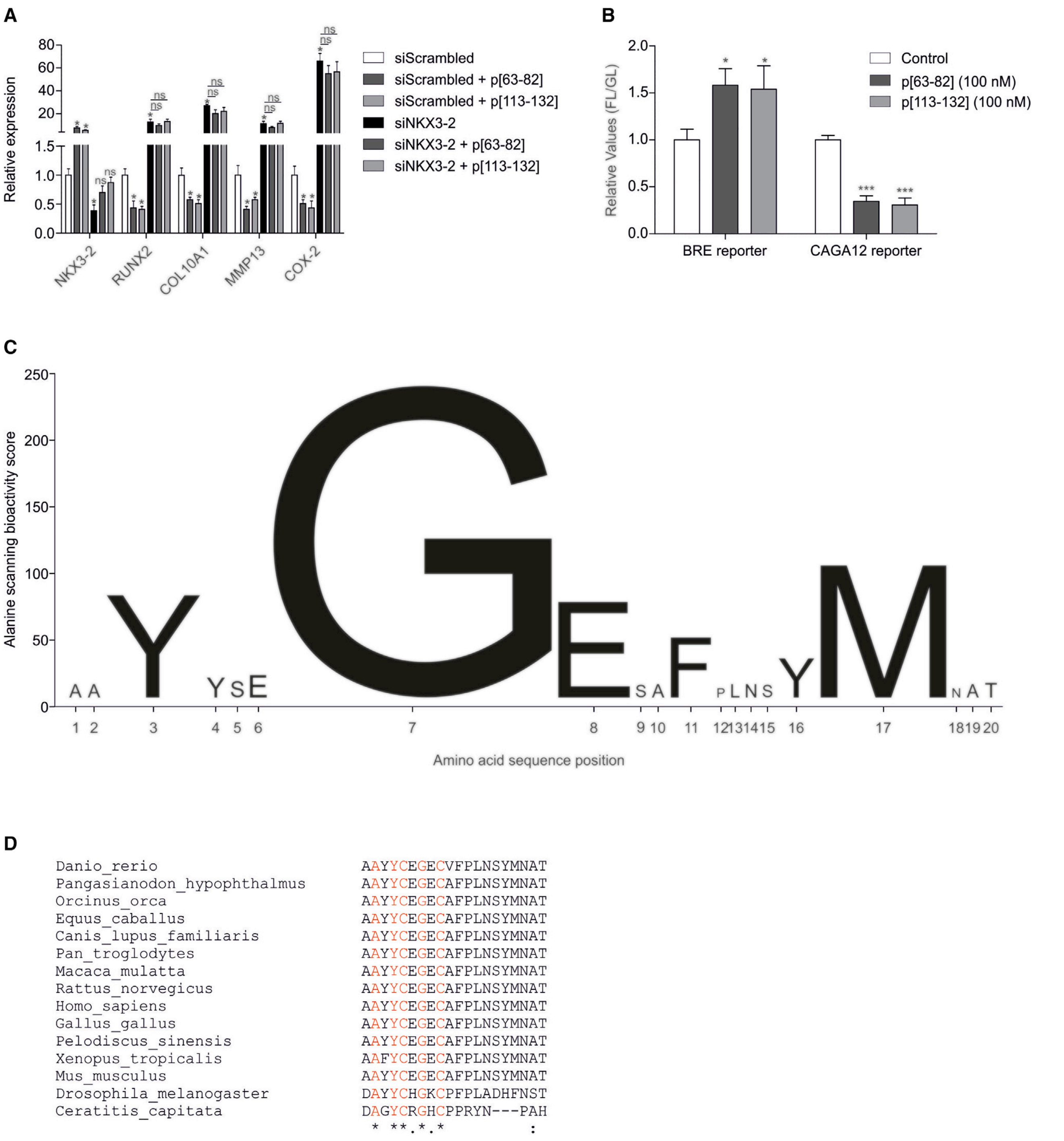

Figure 5. Molecular characterization of peptides p[63–82] and p[113–132]

(A) A pool of OA-HACs (n = 4 donors) was transfected with a scrambled siRNA or with an NKX3-2 siRNA (100 nM siRNA) and cultured for 24 h. Cultures were then exposed to peptide p[63−82] or p[113−132] (100 nM peptide). Samples were harvested 24 h later and analyzed for the expression of the indicated mRNAs (normalized to 28S rRNA). (B) SW1353 chondrocytic cells were transfected with BRE (SMAD1/5/8 reporter) or CAGA12 (SMAD2/3 reporter) firefly luciferase-reporter plasmids. A CMV-Gaussia plasmid was cotransfected as a transfection control. Cells were then cultured for 24 h and subsequently exposed to control, p[63−82], or p[113−132] for 8 h. Firefly and Gaussia bioluminescence were measured in cells and medium (respectively), and relative light units (RLUs) of firefly luciferase were normalized for the Gaussia luciferase signal. The normalized firefly luciferase signal of the control condition was set at 1, and peptide conditions were calculated relative to the control condition (FL/GL). Error bars represent mean ± SEM; statistical significance for peptide conditions versus siScrambled or siNKX3-2 (A) or control condition (B) as determined by unpaired two-tailed Student's t test is

(legend continued on next page)

the saline-injected group, with increasing signs of cartilage degeneration from zone 3 (inside) toward zone 1 (outside). The femoral compartment also developed cartilage degeneration but less pronounced. This compartment is also more variable in its degenerative changes.[35] There were no statistically significant differences between cartilage-degeneration scores of the tibia or femur in MMT rats injected with saline or scrambled p[63−82] in any of the 3 zones (Figures 7B and 7C). Neither was there a significant difference between saline and scrambled p[63−82] in the total cartilage-degeneration scores (Figures 7B and 7C). Histopathology scoring of the group injected with the bioactive p[63−82] peptide revealed significantly lower medial tibia cartilage-degeneration scores in zone 1 (the most severely affected zone in the MMT model) (Figure 7B) and a trend toward a statistically significant lower medial femur cartilage-degeneration score in zone 1, as compared to the saline group (Figure 7C). Cartilage-degeneration scores in zones 2 and 3 were generally low, as is known for the rat MMT model.[35] Combined (total) tibia and femur cartilage-degeneration scores of MMT rats injected with the bioactive p[63−82] peptide were also significantly lower than the saline group (Figures 7B and 7C). A comparison of cartilage-degeneration scores between groups injected with p[63−82] or scrambled p[63−82] did not reveal differences that were statistically significant (Figures 7B and 7C). The scores for depth ratio of tibia cartilage degeneration followed a similar pattern as the tibia cartilage-degeneration scores, with a significantly lower depth ratio in the p[63−82] group compared to saline in zone 1, as well as the total depth ratio score (Figure 7D). In line with these attenuated cartilage-degenerative aspects, the total joint score[35] was significantly lower in the group injected with p[63−82] as compared to the saline-injected group (Figure 7E). The average joint score of the group injected with scrambled p[63−82] peptide was in between the values for saline and p[63−82] peptide but was not statistically different compared to either one of the saline or p[63−82] conditions. Other scored histopathological items can be found in Figure S5. Together, these data demonstrate that the p[63−82] peptide, compared to saline control, is effective in reducing cartilage-degenerative changes in the rat MMT model.

DISCUSSION

Here, we report on the first high-resolution BMP peptide library screening for the identification of peptide sequences derived from BMP7 that attenuate the pathological chondrocyte phenotype associated with OA. Previous attempts to develop peptides derived from BMP family members were based on low-resolution library screening[23,24] or focused on mimicking the canonical ligand-receptor interaction.[23,26,36] The action of BMPs and BMP-derived peptides is cell-type dependent.[28] Therefore, our peptide library screening was not based on *a priori* knowledge on BMP ligand epitopes or activation of receptor signaling but on the identification of BMP7-derived peptide bioactivity capable of attenuating the articular chondrocyte cell phenotype associated with OA.[8,37]

Although a majority of the peptides in the library induced an unfavorable chondrocyte hypertrophy-provoking cellular response, peptides from the central and C-terminal regions of BMP7 were active in altering the OA chondrocyte phenotype in a similar fashion as full-length BMP7 on this cell type. The origin of this region-dependent functional separation of BMP7 peptide bioactivity remains unclear at this point. Earlier work reports on a dominant domain in mature BMP2, BMP7, and BMP9 from which peptides are able to support osteogenesis,[23,24,38,39] promote chondrogenesis,[40] and prevent kidney fibrosis.[26] In these cases, peptide sequences were derived from the C-terminal part of the parental BMPs. This region contains the knuckle epitope, which is involved in BMP signaling via the type II BMP receptor (BMPR-II).[41] The bioactivity of peptides from the knuckle domain can probably be attributed to the binding of the peptides to the BMPR-II, although knuckle peptide-driven activation of ALK3 (a type I receptor) has also been reported.[26] The C-terminal BMP7-derived peptides identified in the present work (represented by p[113−132]) are located in this knuckle domain and overlap with previously reported bioactive peptides from this region of different BMP family members. This indicates that peptides derived from the BMP7 C-terminal domain are likely universally active on a diversity of cell types.

The central region of BMP7 harbors the wrist epitope. This wrist epitope is a key domain shared by BMP protein species and is involved in binding and activating the type IA BMP receptor (BMPR-IA).[41] The here-identified bioactive peptides with OA chondrocyte phenotype-modulating activity derived from the central region of BMP7 (represented by p[63−82]) are only partially located within this wrist domain. A peptide covering the complete wrist domain of BMP9 was recently reported to activate BMP signaling in a cell-type-dependent manner.[28] However, BMP7-derived peptides present in our library and homologous to the previously described BMP9 wrist domain peptide (p[69−88]−p[79−98]) did not act favorably in altering the OA chondrocyte phenotype. Instead, these and C-terminally adjacent peptides showed induced chondrocyte hypertrophy, which is bioactivity that we were not seeking in this study. A potentially unique aspect of the p[63−82] peptide is the notion that its C-terminal part covers a portion of the wrist domain, whereas its N-terminal part is located within the CXGXC motif of BMP proteins. The CXGXC motif is a major part of the ten-membered cystine knot that is involved in the homodimerization of BMP family members and essential for their action as a receptor ligand.[42] Mutation of the G in CXGXC motifs abrogates efficient folding of the domain, impairing dimer formation, as shown previously for the G residue in the

represented as *p < 0.05, **p < 0.01, and ***p < 0.001. (C) An alanine scanning library of peptide p[63−82] was synthesized, and the pool of 18 OA-HACs originally used for the full library scanning (Figure 1) was exposed to individual peptides from the alanine scanning library (100 nM peptide) for 24 h. An alanine-scanning bioactivity score (sum of the fold change gene expression of COL10A1, ALPL, MMP13, and COL2A1 plus NKX3-2 of the alanine-substituted peptides versus the wild-type p[63−82] peptide) was calculated for each alanine substitution in the amino acid sequence of peptide p[63−82] and is represented by the height of each letter on the y axis (data in Figure S4). (D) Multiple sequence alignment of the human BMP7 p[63−82] peptide with the homologous region in BMP7 of other species.

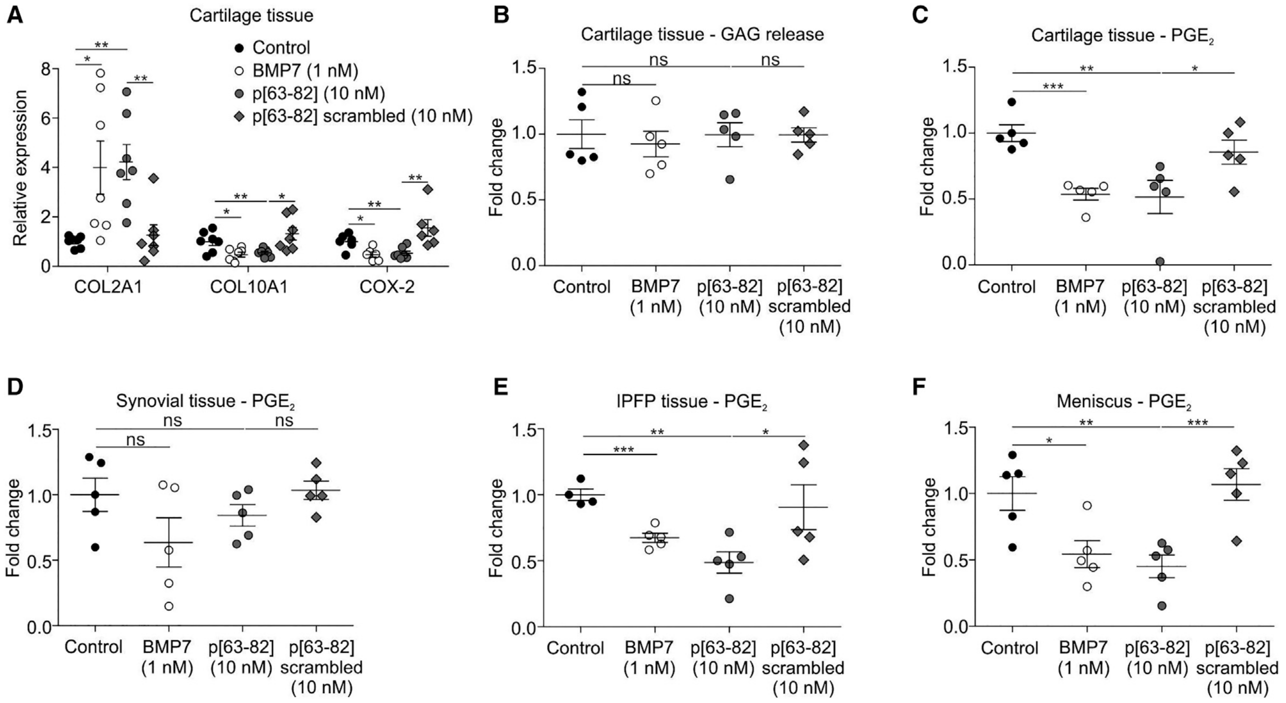

Figure 6. Peptide p[63–82] activity on OA knee joint tissues

Human OA knee joint tissues were cultured for 24 h in the presence of BMP7, peptide p[63−82], or the scrambled version of peptide p[63−82] and compared to control conditions. (A) Cartilage tissues (n = 7 individual donors) were exposed to shown conditions and analyzed for COL2A1, COL10A1, and COX-2 mRNA expression by quantitative real-time PCR (normalized for 28S rRNA expression and relative to control conditions). (B) GAG release in medium from cartilage tissues (n = 5 individual donors) was determined, and data were calculated relative to the control condition. (C) PGE_2 levels in culture supernatant from synovium (n = 5 donors). (D) PGE_2 levels in culture supernatant from cartilage tissues from (B) (n = 5 donors). (E) PGE_2 levels in culture supernatant from IPFP (n = 5 donors). (F) PGE_2 levels in culture supernatant from the meniscus. Synovium, IPFP, and meniscus tissues were from the same 5 donors as the cartilage tissues in (B) and (C). PGE_2 levels in conditions were calculated relative to controls for each donor. In graphs, error bars represent mean ± SEM; data are presented relative to the control condition for each time point. Statistical differences (unpaired two-tailed Student's t test) were calculated to control condition: *p < 0.05, **p < 0.01, and ***p < 0.001.

CXGXC motif of the human chorionic gonadotropin protein.[43] In keeping with this notion, the alanine substitution for which we observed the largest impact on the bioactivity of peptide p[63−82] was G7A, which is the G in the CXGXC motif in the peptide. Although potential disulfide-bridge formation of the peptide with BMP subunits is excluded due to the substitution of all cysteine residues to serine in the library, the major impact of the G7A substitution on peptide p[63−82] function provides a basis for the involvement of functional dimerization of BMP subunits as a potential mechanism for the peptide's action.

A temporary exposure of OA chondrocytes to the peptides leads to a modulation of the cellular phenotype for up to 8 days. The peptides also provoke a general hypertrophy-suppressive response in other chondrocytic cell models (SW1353, C28I2, ATDC5; data not shown) and activated SMAD1/5/8-dependent BRE-reporter activity while reducing the activity of the SMAD2/3-dependent CAGA12 reporter in SW1353 cells. This is unexpected, since activation of SMAD1/5/8 is generally associated with an OA chondrocyte phenotype and its terminal differentiation.[44] In contrast to its clear hypertrophy-suppressive action on OA chondrocytes,[27] BMP7 has previously been shown to activate SMAD1/5/8 signaling[45,46] and inhibit SMAD3 activity.[47] How signaling downstream of different BMPs (e.g., BMP2, BMP4, BMP7, BMP9, etc.) differentiates to BMP-specific target gene expression is largely elusive. As a consequence, it remains to be determined how the action of both peptides alters SMAD-dependent signaling responses related to the chondrocyte phenotype. Our data also reveal that the bioactivity of peptides p[63−82] and p[113−132] depends on the presence of NKX3-2, a well-known BMP-dependent[31] transcriptional regulator for chondrocyte hypertrophy.[29] This recapitulates the molecular mechanism we previously identified for the BMP7-specific attenuation of the (OA) chondrocyte phenotype.[17,27]

The BMP7-driven favorable alterations in the (OA) chondrocyte phenotype not only encompass changes in chondrocyte hypertrophy but are additionally accompanied, among others, by dampening of the chondrocyte's inflammatory status.[10,27,48] This is highlighted by BMP7-driven attenuation of chondrocyte COX-2, IL-6, and PGE_2 levels in the present work, with similar actions for peptides p[63−82] and p[113−132]. BMP7 has previously also been reported

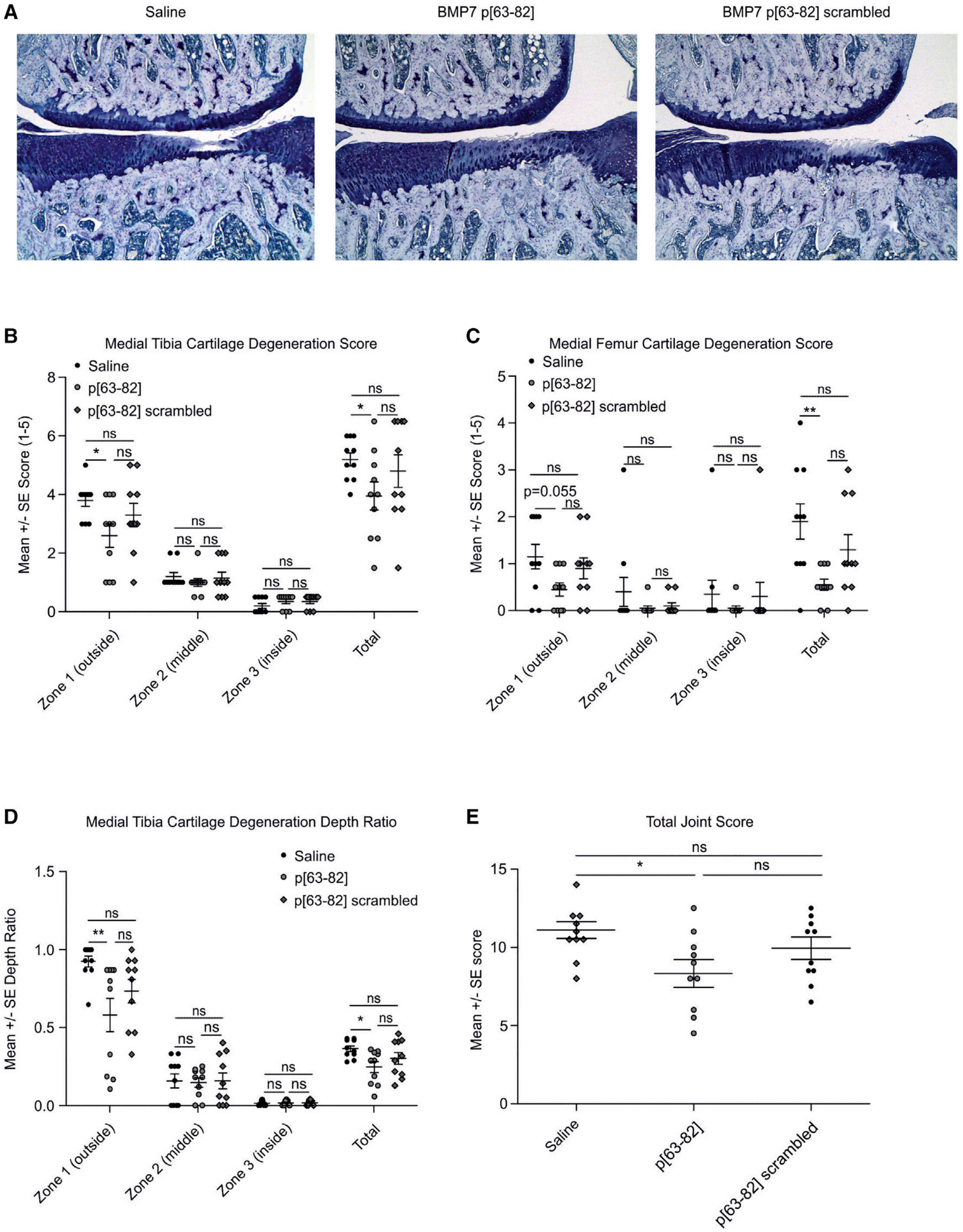

Figure 7. BMP7-derived peptide p[63–82] in rat MMT model

The potency of BMP7 peptide p[63–82] to delay the progression of trauma-induced cartilage degeneration was tested in the rat MMT model. 1 week post-MMT-surgery, rats were two times per week intra-articularly injected with saline, 100 ng peptide p[63–82] in saline, or 100 ng scrambled peptide p[63–82] in saline (10 rats per group). All injection volumes were 50 μL. At 4 weeks post-MMT surgery, rats were sacrificed for histopathological scoring of the MMT knee joints.

(legend continued on next page)

as a morphogen with anti-inflammatory properties in other tissues and cell types like kidney,[49] heart,[50] blood vessels,[50] and macrophages.[51] The observation that these peptides attenuate PGE_2 release from intra-articular tissue other than cartilage (IPFP, meniscus, and not significantly from synovium) is in line with the previously reported tissue-wide anti-inflammatory actions of BMP7.

When applied intra-articularly, OA disease-modifying molecules will have to reach their target tissue via the SF. Regardless of the method of delivery, peptides should thus be able to survive the OA SF environment to a certain extent. In this respect, it is noteworthy that we found that peptides p[63−82] and p[113−132] are able to ameliorate, at least in part, the negative effects of OA SF on the chondrocyte phenotype. Confirming bioactivity in an *in vivo* intra-articular environment, we could indeed demonstrate that the progression of cartilage degeneration in the rat MMT model was attenuated by peptide p[63−82] when compared to injection with saline. Histopathology scores of the group injected with a scrambled version of peptide p[63−82] were never significantly different from the group injected with the bioactive peptide p [63−82]. However, no statistically significant differences between scores of the group injected with saline or the scrambled p [62−83] peptide were found either. The average histopathology scores of the scrambled peptide group were mostly in between the scores from the bioactive p[63−83] peptide and the saline group. Together, this indicates that the scrambled p[62−82] peptide may have some influence on cartilage degeneration in this model, but only the bioactive p[62−83] peptide had the potency to harness a significant inhibiting action on cartilage degeneration. The rat MMT model has been used before to determine the potency of a broad-spectrum MMP inhibitor[34] and the efficacy of fibroblast growth factor (FGF)18.[52] Although the MMT model is generally regarded as a rapidly progressive model for traumatic OA, peptide p[62−83] was able to significantly improve the total joint score in this model to a similar extent as broad-spectrum MMP inhibition[34] or MMP13 inhibition.[53] Besides the attenuated progression of structural cartilage degeneration detected in the present work, pre-clinical follow-up work should address whether the intra-articular administration of peptide p[62−83] dampens the functional consequences of traumatic OA models by determining gait and load bearing.

In conclusion, this study reports on the first high-resolution peptide library screening of a BMP. The articular chondrocyte phenotypic screening identified two regions in BMP7 from which bioactive peptide sequences are able to attenuate the OA chondrocyte phenotype. The p[62−83] peptide spans the conserved CXGXC motif in BMP7 and reaches into its wrist domain with its exact biomolecular mechanism of action to be dissected. These BMP7-derived peptides may represent novel lead molecules for the development of a disease-modifying treatment of OA.

MATERIALS AND METHODS

Cell and tissue culture

Cartilage was obtained from total knee arthroplasty of end-stage (K&L grades 3−4) OA patients and from resected knee cartilage from cartilage repair procedures of non-OA patients. Medical ethical permission was received from the Maastricht University Medical Center Medical Ethical Committee (approval number 2017-0183). Chondrocytes were isolated using collagenase as described earlier.[27] The HACs were cultured until passage two in DMEM/F12 (Life Technologies), 10% fetal calf serum (FCS; Sigma-Aldrich), 1% antibiotic/antimycotic (Life Technologies), and 1% non-essential amino acids (Life Technologies) in a humidified atmosphere at 37°C, 5% CO_2. SW1353 cells (ATCC HTB-94, short tandem repeat [STR] profiled)[54] were cultured in identical conditions. For experiments, cells were plated in technical triplicates at 30,000 cells/cm^2. BMP7 (R&D Systems) was used at 1 nM. OA SF was perioperatively collected from OA patients (same medical ethics statement [2017-0183] as for cartilage above) and was centrifuged to remove cells/debris. For experiments, an SF pool from five OA patients was prepared (equal volume ratios) and applied on cells in a 20% (v/v) concentration. For RNAi, scrambled and NKX3-2 siRNA duplexes (Table S1) were custom made (Eurogentec). Transfection (100 nM siRNA) in HACs was performed using HiPerFect (QIAGEN) according to the manufacturer's protocol. The BRE reporter,[32] CAGA12 reporter,[33] or cytomegalovirus (CMV)-Gaussia as transfection control[55] were transfected (100 ng/μL) in SW1353 cells using FuGENE (Promega) according to the manufacturer's protocol. The bioluminescent readout was performed by lysing cells in Passive Lysis Buffer (Promega) and measuring luciferase activity using the Berthold $TriStar^2$ LB 942 Modular Multimode Microplate Reader using the Luciferase Assay System for firefly luciferase (BRE and CAGA12) (Promega) and the Gaussia Luciferase Kit (New England Biolabs). Cartilage from femoral condyles and tibial plateaus, synovial tissue, the inner parts of the IPFP, or the inner parts of the meniscus (carefully avoiding to obtain synovial tissue present at the outer edges of the meniscus) obtained from OA patients (same medical ethical statement [2017-0183] as above for cartilage) were cut into small pieces, washed thoroughly with 0.9% NaCl (Sigma-Aldrich) three times, and cultured in suspension for 24 h in a concentration of 100 mg tissue/mL in DMEM-F12 low glucose, supplemented with 1% insulin-transferrin-selenite (ITS; Invitrogen) and 1% antibiotic/antimycotic.[56] After overnight culture, the medium was replaced with peptides or BMP7 for 24 h, after which tissue explants were snap frozen in liquid nitrogen, or conditioned medium was harvested and stored at −80°C.

(A) Representative micrographs of toluidine blue-stained sections of medial aspects of the MMT knee joints. Conditions are indicated. (B) Medial tibia cartilage-degeneration scores. (C) Medial femur cartilage-degeneration scores. (D) Medial tibia cartilage-degeneration depth ratios. Scores and ratios are shown for zones 1, 2, and 3 individually.[35] The total scores/ratio are cumulative for zone 1, 2, and 3. (E) Total joint scores. Individual data points represent individual rats. Error bars represent mean ± SEM. Statistical differences were calculated between groups (Mann-Whitney U test), and *p < 0.05, **p < 0.01, and ***p < 0.001. Other data from this MMT experiment are shown in Figure S5.

Peptides

The peptide library was designed from the mature 139-amino acid-long human BMP7 sequence (https://www.ncbi.nlm.nih.gov/protein/4502427; NCBI reference sequence NP_001710). The alanine-scanning library was designed from the peptide sequence covering BMP7 amino acid position 63–82 (p[63–82] from the library). Scrambled sequences were designed from p[63–82] and p[113–132]. All peptides were custom designed and synthesized (Pepscan, Lelystad, the Netherlands) and purified to >90% purity. Peptide sequence identity was confirmed by mass spectrometry. To prevent potential oxidation of free cysteines, all cysteine residues in peptides were substituted for serine residues. Peptide sequences are shown in Table S2.

Gene expression analysis

For the peptide-screening and alanine-scanning experiments, cDNA was prepared using the Cells-to-Ct kit (Invitrogen) according to the manufacturer's protocol. For the other experiments, cells or homogenized tissues (Mikro-Dismembrator; Braun Biotech International) were lysed in TRIzol (Life Technologies) and RNA isolation and cDNA synthesis were performed as described earlier.[27,57] Quantitative real-time PCR was performed using Takyon qPCR Master Mix plus blue for SYBR Green (Eurogentec). A CFX96 Real-Time PCR Detection System (Bio-Rad) was used for amplification: initial denaturation 95°C for 10 min, followed by 40 cycles of amplification (denaturing 15 s at 95°C and annealing 1 min at 60°C). Validated primer sequences are shown in Table S3. Data were analyzed using the standard curve method, mRNA expression was normalized to a reference gene (28S rRNA), and gene expression was calculated as fold change as compared to control conditions.

sGAG assay

The sGAG content was measured using a modified dimethyl methylene blue (DMB) assay.[58] The absorbance of samples was read at 540 and 595 nm using a spectrophotometer (Multiskan FC; Thermo Fisher Scientific). GAG concentrations were calculated using a standard curve of chondroitin sulfate (Sigma-Aldrich). GAGs were normalized for total protein content with a bicinchoninic acid assay (BCA) assay (Sigma-Aldrich).

ALP activity assay

Cells were lysed in 1.5 M Tris-HCl (pH 9.0) and 2% (v/v) Triton X-100 and homogenized by sonication (Soniprep 150 MSE). Insoluble material was removed by centrifugation (5 min; 13,000 × *g*; 4°C). Total protein concentration was determined by a BCA assay. ALP enzyme activity in time was measured by ALP-dependent enzymatic conversion of p-nitrophenyl phosphate to p-nitrophenol in buffer containing 1.5 M Tris-HCl (pH 9.0), 1 mM $ZnCl_2$, 1 mM $MgCl_2$, and 7.5 mM p-nitrophenyl phosphate. Substrate conversion was spectrophotometrically quantified at 405 nm, and p-nitrophenol concentrations were determined via a p-nitrophenol calibration series. Values were normalized to total protein concentration, and ALP enzyme activity was calculated in units (1 U = 1 μmol/g/min).

PGE_2 ELISA

PGE_2 levels were determined in the culture supernatant. PGE_2 concentration was determined by a standardized enzyme immunoassay (EIA) according to the manufacturer's protocol (Cayman Chemical).

Animal study

The study was conducted via a contract research project at Bolder BioPATH (Boulder, CO, USA); study designs and animal usage were approved by their Institutional Animal Care and Use Committee (IACUC) prior to study initiation (IACUC protocols BBP13-029 and BBP12-004). Animal care, including room, cage, and equipment sanitation, conformed to accepted guidelines cited in the Guide for the Care and Use of Laboratory Animals (the Guide; 2011) and the applicable Bolder BioPATH standard operating procedures (SOPs). Male Lewis rats (n = 48; ~283 g; Envigo RMS, Indianapolis, IN, USA) underwent a unilateral MMT on study day 0, as established earlier by Bendele et al.[59,60] Rats were intra-articularly injected on days 7, 10, 14, 17, 21, and 24 with saline (0.9% NaCl, 50 μL/injection/animal), peptide p[63–82] (100 ng [in 50 μL saline]/injection/animal), or scrambled peptide p[63–82] (100 ng [in 50 μL saline]/injection/animal). The animals were euthanized for necropsy 28 days post-surgery.[59,60] Right (surgery) knees from all animals were collected, trimmed of muscle (patella removed), and placed in 10% neutral-buffered formalin and then transferred to 70% ethanol for processing for microscopy. Operated joints were cut into two approximately equal halves in the frontal plane and embedded in paraffin. One section was cut from each knee and stained with toluidine blue. The worst-case scenario for the two halves on each slide was determined and used for evaluation. The tissues were analyzed microscopically by a veterinary pathologist as described earlier[35] (Tables S4–S13). An example of the predominant sites where lesions formed in this model in the tibial and femoral cartilage is indicated in Figure S6.

Statistics

Statistical significance for *in vitro* and *ex vivo* experiments presented in Figures 1, 2, 3, 4, 5, and 6 was determined by two-tailed Student's t tests using GraphPad PRISM 5.0 (La Jolla, CA, USA). With the consideration of small sample sizes (Figures 1, 2, 3, 4, and 5, n = 3 samples; Figure 6, n = 5 samples), normal distribution of input data was assumed. For histopathology from the animal study, peptide p[63–82], scrambled peptide p[63–82], and saline groups were compared using a Mann-Whitney U test, as not all input data passed the D'Agostino-Pearson omnibus normality tests. Significance for all tests was set at $p \leq 0.05$. Error bars in graphs represent mean ± standard error of the mean.

SUPPLEMENTAL INFORMATION

Supplemental information can be found online at https://doi.org/10.1016/j.omtm.2021.03.009.

ACKNOWLEDGMENTS

The authors thank Eke van Zwol for her contributions to Figure 6. This work was financially supported by grants from the Dutch

Arthritis Association (13-2-201, 15-3-403, and LLP14) and Stichting De Weijerhorst.

AUTHOR CONTRIBUTIONS

Substantial contributions to research design, M.M.J.C., E.G.J.R., G.v.d.A., N.K.A.P.W., L.W.v.R., P.J.E., and T.J.M.W.; substantial contributions to the acquisition of samples, M.M.J.C., E.G.J.R., G.v.d.A., N.K.A.P.W., J.S., D.A.M.S., A.C., and T.J.M.W.; substantial contributions to analysis, M.M.J.C., E.G.J.R., G.v.d.A., N.K.A.P.W., J.S., D.A.M.S., A.C., and T.J.M.W.; substantial contributions to the interpretation of data, M.M.J.C., E.G.J.R., G.v.d.A., N.K.A.P.W., J.S., D.A.M.S., A.C., P.J.E., L.W.v.R., and T.J.M.W.; drafting the paper, M.M.J.C., E.G.J.R., G.v.d.A., and T.J.M.W.; revising paper critically, M.M.J.C., E.G.J.R., G.v.d.A., N.K.A.P.W., J.S., D.A.M.S., A.C., P.J.E., L.W.v.R., and T.J.M.W.; approval of the submitted and final versions, M.M.J.C., E.G.J.R., G.v.d.A., N.K.A.P.W., J.S., D.A.M.S., A.C., P.J.E., L.W.v.R., and T.J.M.W. All authors have read and approved the final submitted manuscript.

DECLARATION OF INTERESTS

The study sponsors had no involvement in study design, collection, analysis, and interpretation of data; the writing of the manuscript; or the decision to submit the manuscript for publication. M.M.J.C. and T.J.M.W. are inventors on patents WO2017178251 and WO2017178253 (owned by Chondropeptix). P.J.E., L.W.v.R., and T.J.M.W. are shareholders in Chondropeptix and are CMO, CDO, and CSO of Chondropeptix, respectively. The other authors declare no competing interests.

REFERENCES

1. Loeser, R.F., Goldring, S.R., Scanzello, C.R., and Goldring, M.B. (2012). Osteoarthritis: a disease of the joint as an organ. Arthritis Rheum. *64*, 1697–1707.
2. Sofat, N. (2009). Analysing the role of endogenous matrix molecules in the development of osteoarthritis. Int. J. Exp. Pathol. *90*, 463–479.
3. Felson, D.T., Lawrence, R.C., Dieppe, P.A., Hirsch, R., Helmick, C.G., Jordan, J.M., Kington, R.S., Lane, N.E., Nevitt, M.C., Zhang, Y., et al. (2000). Osteoarthritis: new insights. Part 1: the disease and its risk factors. Ann. Intern. Med. *133*, 635–646.
4. Dreier, R. (2010). Hypertrophic differentiation of chondrocytes in osteoarthritis: the developmental aspect of degenerative joint disorders. Arthritis Res. Ther. *12*, 216.
5. Tchetina, E.V. (2011). Developmental mechanisms in articular cartilage degradation in osteoarthritis. Arthritis (Egypt) *2011*, 683970.
6. van der Kraan, P.M., and van den Berg, W.B. (2012). Chondrocyte hypertrophy and osteoarthritis: role in initiation and progression of cartilage degeneration? Osteoarthritis Cartilage *20*, 223–232.
7. Berenbaum, F., and van den Berg, W.B. (2015). Inflammation in osteoarthritis: changing views. Osteoarthritis Cartilage *23*, 1823–1824.
8. Ji, Q., Zheng, Y., Zhang, G., Hu, Y., Fan, X., Hou, Y., Wen, L., Li, L., Xu, Y., Wang, Y., and Tang, F. (2019). Single-cell RNA-seq analysis reveals the progression of human osteoarthritis. Ann. Rheum. Dis. *78*, 100–110.
9. Hunter, D.J. (2011). Pharmacologic therapy for osteoarthritis–the era of disease modification. Nat. Rev. Rheumatol. *7*, 13–22.
10. Im, H.J., Pacione, C., Chubinskaya, S., Van Wijnen, A.J., Sun, Y., and Loeser, R.F. (2003). Inhibitory effects of insulin-like growth factor-1 and osteogenic protein-1 on fibronectin fragment- and interleukin-1beta-stimulated matrix metalloproteinase-13 expression in human chondrocytes. J. Biol. Chem. *278*, 25386–25394.
11. Stöve, J., Schneider-Wald, B., Scharf, H.P., and Schwarz, M.L. (2006). Bone morphogenetic protein 7 (bmp-7) stimulates proteoglycan synthesis in human osteoarthritic chondrocytes in vitro. Biomed. Pharmacother. *60*, 639–643.
12. Chubinskaya, S., Otten, L., Soeder, S., Borgia, J.A., Aigner, T., Rueger, D.C., and Loeser, R.F. (2011). Regulation of chondrocyte gene expression by osteogenic protein-1. Arthritis Res. Ther. *13*, R55.
13. Chubinskaya, S., Hurtig, M., and Rueger, D.C. (2007). OP-1/BMP-7 in cartilage repair. Int. Orthop. *31*, 773–781.
14. Hayashi, M., Muneta, T., Ju, Y.J., Mochizuki, T., and Sekiya, I. (2008). Weekly intra-articular injections of bone morphogenetic protein-7 inhibits osteoarthritis progression. Arthritis Res. Ther. *10*, R118.
15. Sekiya, I., Tang, T., Hayashi, M., Morito, T., Ju, Y.J., Mochizuki, T., and Muneta, T. (2009). Periodic knee injections of BMP-7 delay cartilage degeneration induced by excessive running in rats. J. Orthop. Res. *27*, 1088–1092.
16. Hunter, D.J., Pike, M.C., Jonas, B.L., Kissin, E., Krop, J., and McAlindon, T. (2010). Phase 1 safety and tolerability study of BMP-7 in symptomatic knee osteoarthritis. BMC Musculoskelet. Disord. *11*, 232.
17. Caron, M.M., Emans, P.J., Cremers, A., Surtel, D.A., Coolsen, M.M., van Rhijn, L.W., and Welting, T.J. (2013). Hypertrophic differentiation during chondrogenic differentiation of progenitor cells is stimulated by BMP-2 but suppressed by BMP-7. Osteoarthritis Cartilage *21*, 604–613.
18. Lohmander, L.S., Hoerrner, L.A., and Lark, M.W. (1993). Metalloproteinases, tissue inhibitor, and proteoglycan fragments in knee synovial fluid in human osteoarthritis. Arthritis Rheum. *36*, 181–189.
19. Mantle, D., Falkous, G., and Walker, D. (1999). Quantification of protease activities in synovial fluid from rheumatoid and osteoarthritis cases: comparison with antioxidant and free radical damage markers. Clin. Chim. Acta *284*, 45–58.
20. Tchetverikov, I., Lohmander, L.S., Verzijl, N., Huizinga, T.W., TeKoppele, J.M., Hanemaaijer, R., and DeGroot, J. (2005). MMP protein and activity levels in synovial fluid from patients with joint injury, inflammatory arthritis, and osteoarthritis. Ann. Rheum. Dis. *64*, 694–698.
21. Montjean, R., Escaich, S., Paolini, R., Carelli, C., Pirson, S., Neutelings, T., Henrotin, Y., and Vêtu, C. (2020). REG-O3 chimeric peptide combining growth hormone and somatostatin sequences improves joint function and prevents cartilage degradation in rat model of traumatic knee osteoarthritis. PLoS ONE *15*, e0231240.
22. Liu, Q., Jia, Z., Duan, L., Xiong, J., Wang, D., and Ding, Y. (2018). Functional peptides for cartilage repair and regeneration. Am. J. Transl. Res. *10*, 501–510.
23. Kirkwood, K., Rheude, B., Kim, Y.J., White, K., and Dee, K.C. (2003). In vitro mineralization studies with substrate-immobilized bone morphogenetic protein peptides. J. Oral Implantol. *29*, 57–65.
24. Chen, Y., and Webster, T.J. (2009). Increased osteoblast functions in the presence of BMP-7 short peptides for nanostructured biomaterial applications. J. Biomed. Mater. Res. A *91*, 296–304.
25. Kim, H.K., Lee, J.S., Kim, J.H., Seon, J.K., Park, K.S., Jeong, M.H., and Yoon, T.R. (2017). Bone-forming peptide-2 derived from BMP-7 enhances osteoblast differentiation from multipotent bone marrow stromal cells and bone formation. Exp. Mol. Med. *49*, e328.
26. Sugimoto, H., LeBleu, V.S., Bosukonda, D., Keck, P., Taduri, G., Bechtel, W., Okada, H., Carlson, W., Jr., Bey, P., Rusckowski, M., et al. (2012). Activin-like kinase 3 is important for kidney regeneration and reversal of fibrosis. Nat. Med. *18*, 396–404.
27. Caron, M.M.J., Emans, P.J., Surtel, D.A.M., van der Kraan, P.M., van Rhijn, L.W., and Welting, T.J.M. (2015). BAPX-1/NKX-3.2 acts as a chondrocyte hypertrophy molecular switch in osteoarthritis. Arthritis Rheumatol. *67*, 2944–2956.
28. Tong, Z., Guo, J., Glen, R.C., Morrell, N.W., and Li, W. (2019). A Bone Morphogenetic Protein (BMP)-derived Peptide Based on the Type I Receptor-binding Site Modifies Cell-type Dependent BMP Signalling. Sci. Rep. *9*, 13446.
29. Provot, S., Kempf, H., Murtaugh, L.C., Chung, U.I., Kim, D.W., Chyung, J., Kronenberg, H.M., and Lassar, A.B. (2006). Nkx3.2/Bapx1 acts as a negative regulator of chondrocyte maturation. Development *133*, 651–662.
30. Yamashita, S., Andoh, M., Ueno-Kudoh, H., Sato, T., Miyaki, S., and Asahara, H. (2009). Sox9 directly promotes Bapx1 gene expression to repress Runx2 in chondrocytes. Exp. Cell Res. *315*, 2231–2240.

31. Kim, D.W., and Lassar, A.B. (2003). Smad-dependent recruitment of a histone deacetylase/Sin3A complex modulates the bone morphogenetic protein-dependent transcriptional repressor activity of Nkx3.2. Mol. Cell. Biol. *23*, 8704–8717.

32. Korchynskyi, O., and ten Dijke, P. (2002). Identification and functional characterization of distinct critically important bone morphogenetic protein-specific response elements in the Id1 promoter. J. Biol. Chem. *277*, 4883–4891.

33. Dennler, S., Itoh, S., Vivien, D., ten Dijke, P., Huet, S., and Gauthier, J.M. (1998). Direct binding of Smad3 and Smad4 to critical TGF beta-inducible elements in the promoter of human plasminogen activator inhibitor-type 1 gene. EMBO J. *17*, 3091–3100.

34. Janusz, M.J., Bendele, A.M., Brown, K.K., Taiwo, Y.O., Hsieh, L., and Heitmeyer, S.A. (2002). Induction of osteoarthritis in the rat by surgical tear of the meniscus: Inhibition of joint damage by a matrix metalloproteinase inhibitor. Osteoarthritis Cartilage *10*, 785–791.

35. Gerwin, N., Bendele, A.M., Glasson, S., and Carlson, C.S. (2010). The OARSI histopathology initiative - recommendations for histological assessments of osteoarthritis in the rat. Osteoarthritis Cartilage *18* (*Suppl 3*), S24–S34.

36. Saito, A., Suzuki, Y., Ogata, Si., Ohtsuki, C., and Tanihara, M. (2003). Activation of osteo-progenitor cells by a novel synthetic peptide derived from the bone morphogenetic protein-2 knuckle epitope. Biochim. Biophys. Acta *1651*, 60–67.

37. Ripmeester, E.G.J., Timur, U.T., Caron, M.M.J., and Welting, T.J.M. (2018). Recent Insights into the Contribution of the Changing Hypertrophic Chondrocyte Phenotype in the Development and Progression of Osteoarthritis. Front. Bioeng. Biotechnol. *6*, 18.

38. Bergeron, E., Senta, H., Mailloux, A., Park, H., Lord, E., and Faucheux, N. (2009). Murine preosteoblast differentiation induced by a peptide derived from bone morphogenetic proteins-9. Tissue Eng. Part A *15*, 3341–3349.

39. Suzuki, Y., Tanihara, M., Suzuki, K., Saitou, A., Sufan, W., and Nishimura, Y. (2000). Alginate hydrogel linked with synthetic oligopeptide derived from BMP-2 allows ectopic osteoinduction in vivo. J. Biomed. Mater. Res. *50*, 405–409.

40. Renner, J.N., Kim, Y., and Liu, J.C. (2012). Bone morphogenetic protein-derived peptide promotes chondrogenic differentiation of human mesenchymal stem cells. Tissue Eng. Part A *18*, 2581–2589.

41. Miyazono, K., Kamiya, Y., and Morikawa, M. (2010). Bone morphogenetic protein receptors and signal transduction. J. Biochem. *147*, 35–51.

42. Groppe, J., Greenwald, J., Wiater, E., Rodriguez-Leon, J., Economides, A.N., Kwiatkowski, W., Affolter, M., Vale, W.W., Izpisua Belmonte, J.C., and Choe, S. (2002). Structural basis of BMP signalling inhibition by the cystine knot protein Noggin. Nature *420*, 636–642.

43. Kinoshita, K., Kusunoki, M., and Miyai, K. (2006). Analysis of the three dimensional structure of the CXGXC motif in the CMGCC and CAGYC regions of alpha-and beta-subunits of human chorionic gonadotropin: importance of glycine residue (G) in the motif. Endocr. J. *53*, 51–58.

44. van der Kraan, P.M., Blaney Davidson, E.N., Blom, A., and van den Berg, W.B. (2009). TGF-beta signaling in chondrocyte terminal differentiation and osteoarthritis: modulation and integration of signaling pathways through receptor-Smads. Osteoarthritis Cartilage *17*, 1539–1545.

45. Chang, H.M., Cheng, J.C., and Leung, P.C. (2013). Theca-derived BMP4 and BMP7 down-regulate connexin43 expression and decrease gap junction intercellular communication activity in immortalized human granulosa cells. J. Clin. Endocrinol. Metab. *98*, E437–E445.

46. Huang, X., Zhong, L., Post, J.N., and Karperien, M. (2018). Co-treatment of TGF-β3 and BMP7 is superior in stimulating chondrocyte redifferentiation in both hypoxia and normoxia compared to single treatments. Sci. Rep. *8*, 10251.

47. Luo, D.D., Phillips, A., and Fraser, D. (2010). Bone morphogenetic protein-7 inhibits proximal tubular epithelial cell Smad3 signaling via increased SnoN expression. Am. J. Pathol. *176*, 1139–1147.

48. Fan, Z., Chubinskaya, S., Rueger, D.C., Bau, B., Haag, J., and Aigner, T. (2004). Regulation of anabolic and catabolic gene expression in normal and osteoarthritic adult human articular chondrocytes by osteogenic protein-1. Clin. Exp. Rheumatol. *22*, 103–106.

49. Li, R.X., Yiu, W.H., Wu, H.J., Wong, D.W.L., Chan, L.Y.Y., Lin, M., Leung, J.C.K., Lai, K.N., and Tang, S.C.W. (2015). BMP7 reduces inflammation and oxidative stress in diabetic tubulopathy. Clin. Sci. (Lond.) *128*, 269–280.

50. Aluganti Narasimhulu, C., and Singla, D.K. (2020). The Role of Bone Morphogenetic Protein 7 (BMP-7) in Inflammation in Heart Diseases. Cells *9*, 280.

51. Singla, D.K., Singla, R., and Wang, J. (2016). BMP-7 Treatment Increases M2 Macrophage Differentiation and Reduces Inflammation and Plaque Formation in Apo E-/- Mice. PLoS ONE *11*, e0147897.

52. Moore, E.E., Bendele, A.M., Thompson, D.L., Littau, A., Waggie, K.S., Reardon, B., and Ellsworth, J.L. (2005). Fibroblast growth factor-18 stimulates chondrogenesis and cartilage repair in a rat model of injury-induced osteoarthritis. Osteoarthritis Cartilage *13*, 623–631.

53. Baragi, V.M., Becher, G., Bendele, A.M., Biesinger, R., Bluhm, H., Boer, J., Deng, H., Dodd, R., Essers, M., Feuerstein, T., et al. (2009). A new class of potent matrix metalloproteinase 13 inhibitors for potential treatment of osteoarthritis: Evidence of histologic and clinical efficacy without musculoskeletal toxicity in rat models. Arthritis Rheum. *60*, 2008–2018.

54. Gebauer, M., Saas, J., Sohler, F., Haag, J., Söder, S., Pieper, M., Bartnik, E., Beninga, J., Zimmer, R., and Aigner, T. (2005). Comparison of the chondrosarcoma cell line SW1353 with primary human adult articular chondrocytes with regard to their gene expression profile and reactivity to IL-1β. Osteoarthritis Cartilage *13*, 697–708.

55. Groot, A.J., Habets, R., Yahyanejad, S., Hodin, C.M., Reiss, K., Saftig, P., Theys, J., and Vooijs, M. (2014). Regulated proteolysis of NOTCH2 and NOTCH3 receptors by ADAM10 and presenilins. Mol. Cell. Biol. *34*, 2822–2832.

56. Timur, U.T., Caron, M.M.J., Bastiaansen-Jenniskens, Y.M., Welting, T.J.M., van Rhijn, L.W., van Osch, G.J.V.M., and Emans, P.J. (2018). Celecoxib-mediated reduction of prostanoid release in Hoffa's fat pad from donors with cartilage pathology results in an attenuated inflammatory phenotype. Osteoarthritis Cartilage *26*, 697–706.

57. Caron, M.M., Emans, P.J., Surtel, D.A., Cremers, A., Voncken, J.W., Welting, T.J., and van Rhijn, L.W. (2012). Activation of NF-κB/p65 facilitates early chondrogenic differentiation during endochondral ossification. PLoS ONE *7*, e33467.

58. Farndale, R.W., Sayers, C.A., and Barrett, A.J. (1982). A direct spectrophotometric microassay for sulfated glycosaminoglycans in cartilage cultures. Connect. Tissue Res. *9*, 247–248.

59. Bendele, A.M. (2001). Animal models of osteoarthritis. J. Musculoskelet. Neuronal Interact. *1*, 363–376.

60. Bendele, A.M. (2002). Animal models of osteoarthritis in an era of molecular biology. J. Musculoskelet. Neuronal Interact. *2*, 501–503.

OMTM, Volume *21*

Supplemental information

Discovery of bone morphogenetic protein 7-derived peptide sequences that attenuate the human osteoarthritic chondrocyte phenotype

Marjolein M.J. Caron, Ellen G.J. Ripmeester, Guus van den Akker, Nina K.A. P. Wijnands, Jessica Steijns, Don A.M. Surtel, Andy Cremers, Pieter J. Emans, Lodewijk W. van Rhijn, and Tim J.M. Welting

SUPPLEMENTARY FIGURES

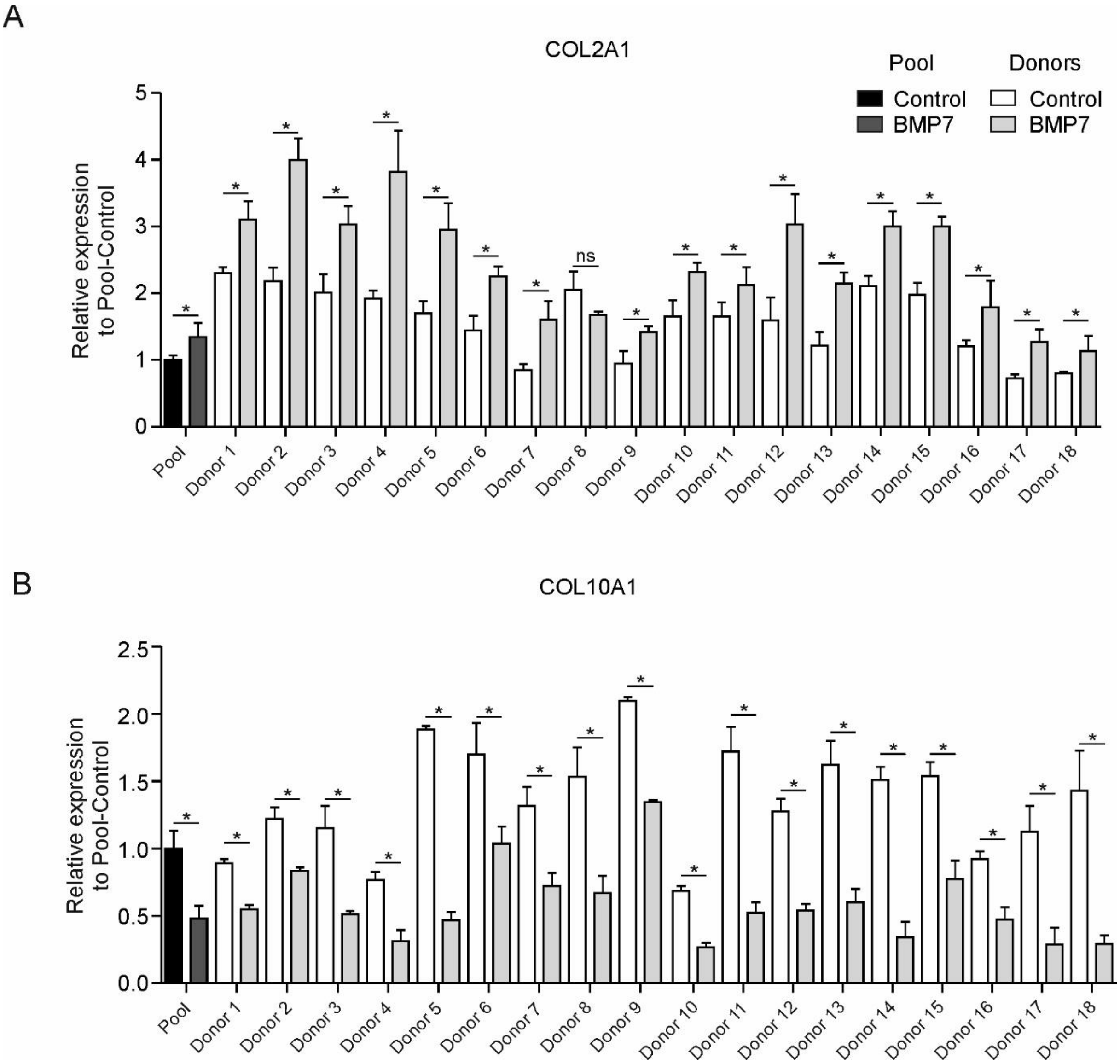

Supplementary Figure 1: Validation of BMP7 responsiveness of OA-HACs and pool. A: COL2A1 mRNA expression of OA-HAC pool and its individual donors in response to BMP7 (1 nM) for 24 hours. **B:** COL10A1 mRNA expression of OA-HAC pool and its individual donors in response to BMP7 (1 nM) for 24 hours. Gene expression was normalized for 28S rRNA expression and set relative to Pool Control condition. Error bars represent mean ± SEM and statistical significance for BMP7 versus control condition for each donor and the pool as determined by unpaired two-tailed student's t-test is represented as: * is p<0.05, ** is p<0.01, *** is p<0.001 and ns is=not significant.

Supplementary Figure 2: Full RT-qPCR library screening data for SOX9, COL2A1, NKX3-2, RUNX2, COL10A1, ALPL, COX-2, MMP13, ADAMTS5, IL6 mRNAs and for all peptide concentrations tested. **A:** SOX9, **B:** COL2A1, **C:** NKX3-2 mRNAs expression in OA-HAC pool following 24 hours exposure to individual BMP7 library peptides at concentrations of 1000, 10, 10 or 1 nM. Controls represent vehicle and full-length recombinant BMP7 (1 nM). Peptide identities are indicated and are organized from the N-terminus toward C-terminus of BMP7. **D/E:** RUNX2, **F/G:** COL10A1, **H/I:** ALPL, **J/K:** COX-2, **L/M:** MMP13, **N/O:** ADAMTS5, **P/Q:** IL6; same as above, but shown with or without y-axis interruption. Gene expression was normalized for 28S rRNA expression and set relative to Pool Control condition. Error bars represent mean ± SEM.

A

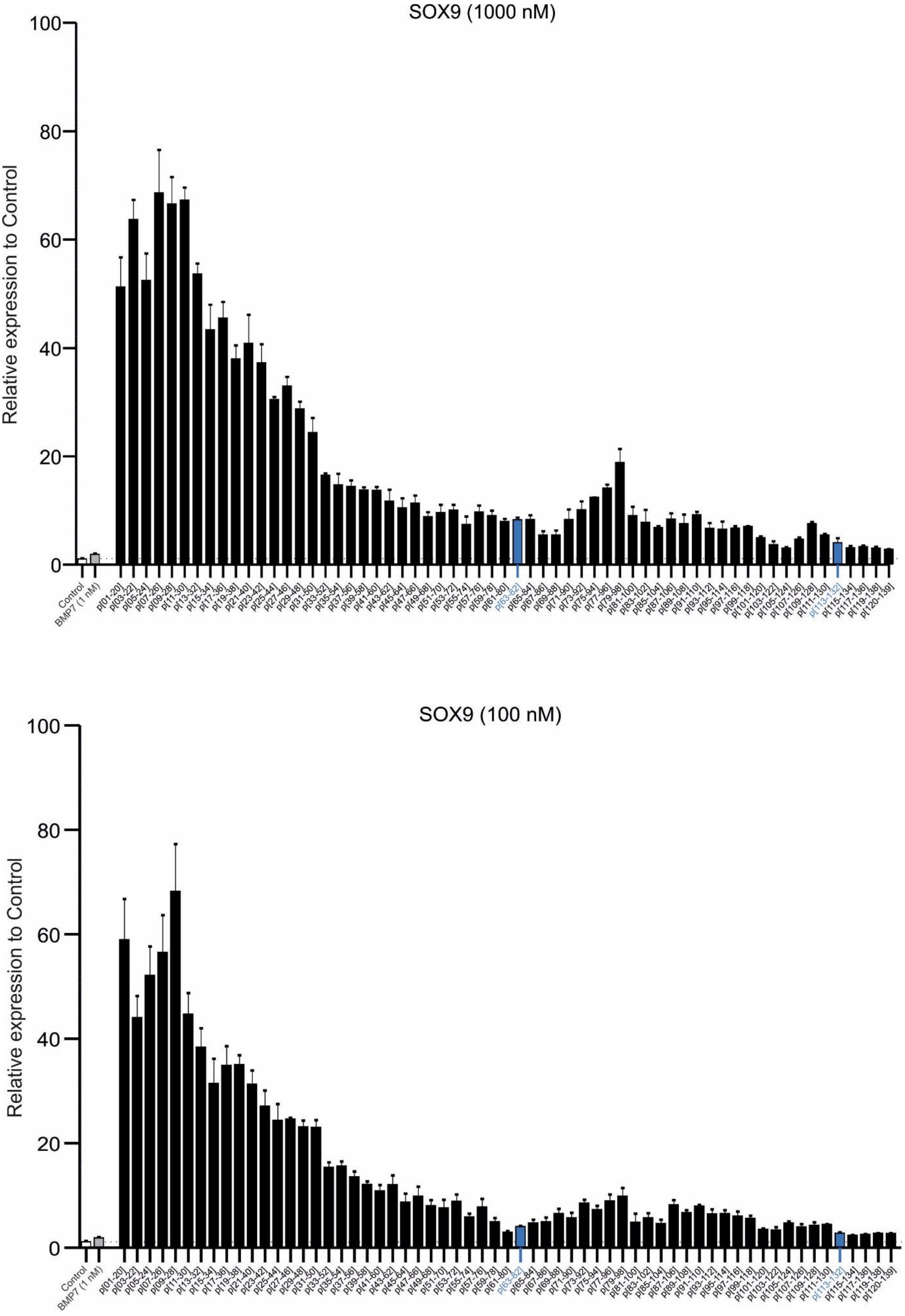

SOX9 (1000 nM)
Relative expression to Control
100
80
60
40
20
0
Control
BMP7 (1 nM)
SOX9 (100 nM)
Relative expression to Control
100
80
60
40
20
0
Control
BMP7 (1 nM)

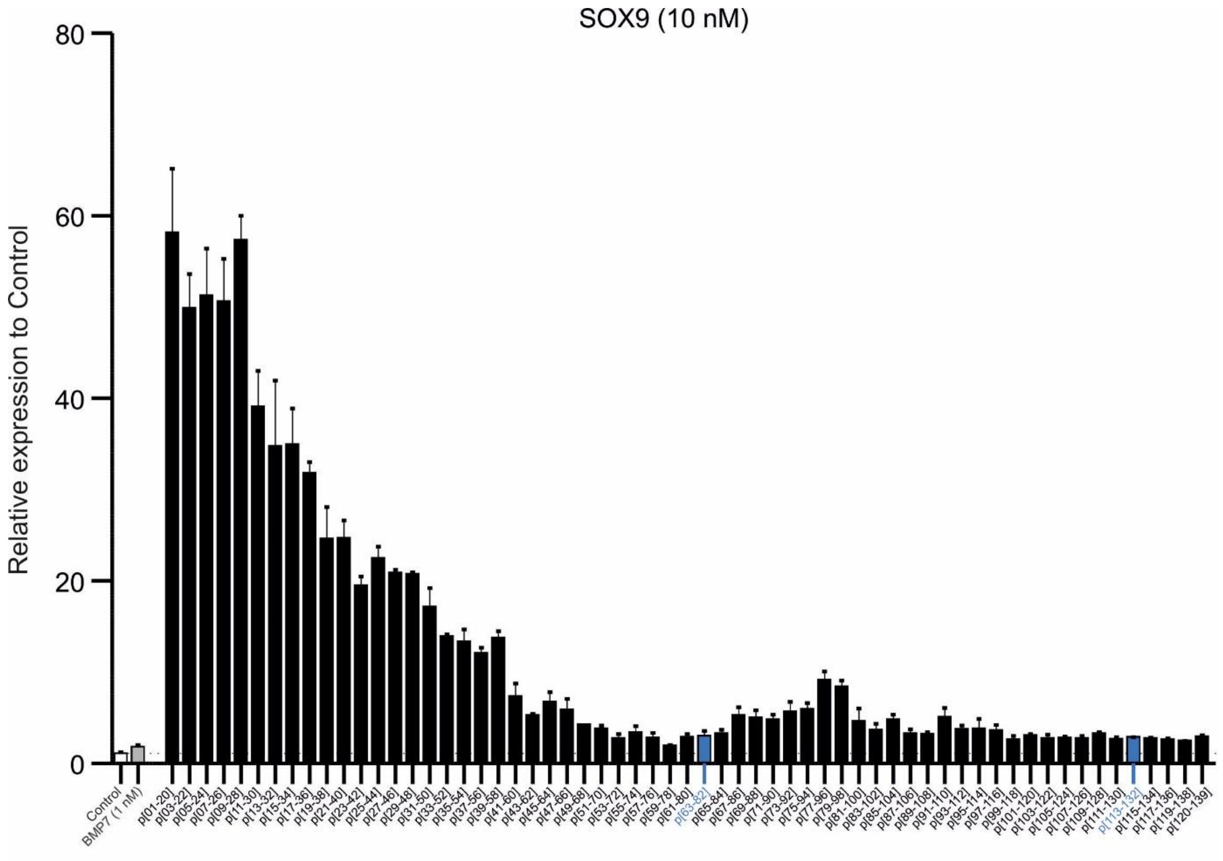
SOX9 (10 nM)
Relative expression to Control
80
60
40
20
0
Control
BMP7 (1 nM)
p[01-20]
p[03-22]
p[05-24]
p[07-26]
p[09-28]
p[11-30]
p[13-32]
p[15-34]
p[17-36]
p[19-38]
p[21-40]
p[23-42]
p[25-44]
p[27-46]
p[29-48]
p[31-50]
p[33-52]
p[35-54]
p[37-56]
p[39-58]
p[41-60]
p[43-62]
p[45-64]
p[47-66]
p[49-68]
p[51-70]
p[53-72]
p[55-74]
p[57-76]
p[59-78]
p[61-80]
p[63-82]
p[65-84]
p[67-86]
p[69-88]
p[71-90]
p[73-92]
p[75-94]
p[77-96]
p[79-98]
p[81-100]
p[83-102]
p[85-104]
p[87-106]
p[89-108]
p[91-110]
p[93-112]
p[95-114]
p[97-116]
p[99-118]
p[101-120]
p[103-122]
p[105-124]
p[107-126]
p[109-128]
p[111-130]
p[113-132]
p[115-134]
p[117-136]
p[119-138]
p[120-139]

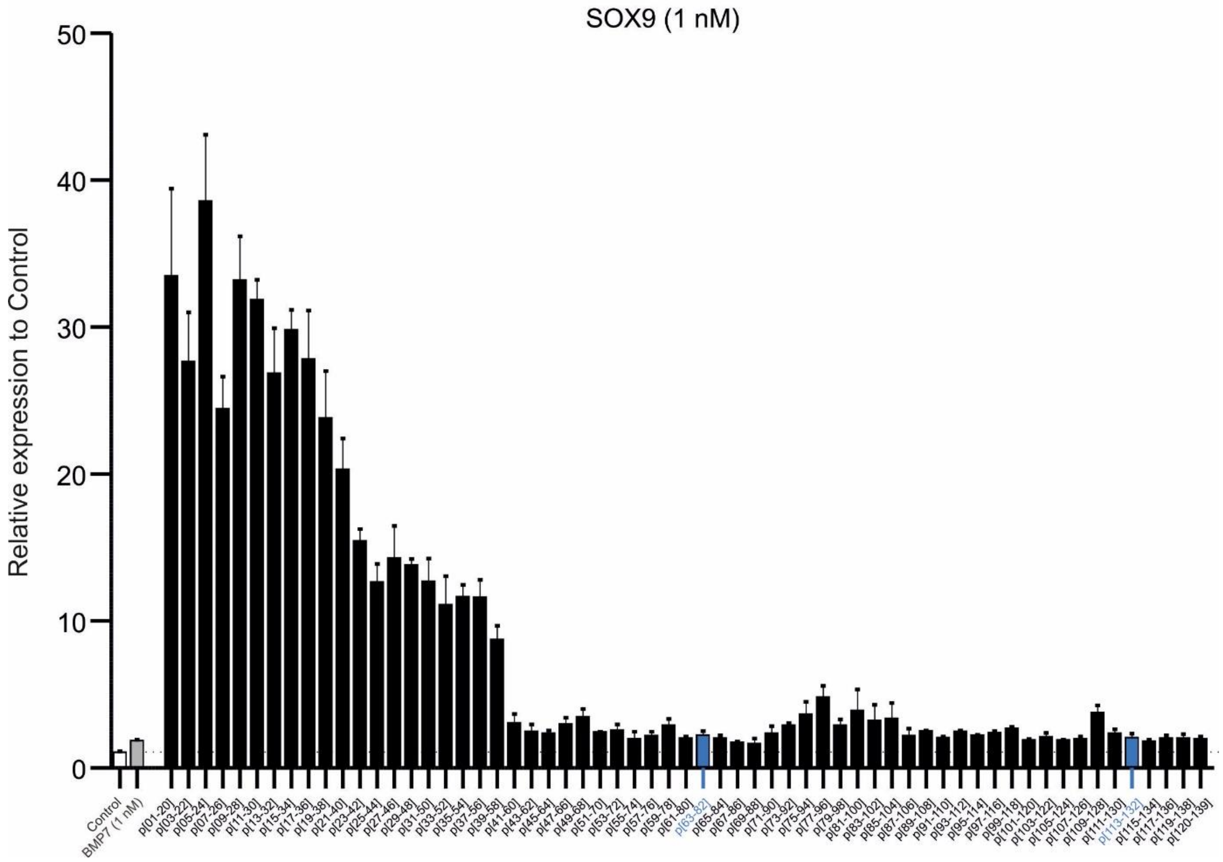
SOX9 (1 nM)
Relative expression to Control
50
40
30
20
10
0
Control
BMP7 (1 nM)
p[01-20]
p[03-22]
p[05-24]
p[07-26]
p[09-28]
p[11-30]
p[13-32]
p[15-34]
p[17-36]
p[19-38]
p[21-40]
p[23-42]
p[25-44]
p[27-46]
p[29-48]
p[31-50]
p[33-52]
p[35-54]
p[37-56]
p[39-58]
p[41-60]
p[43-62]
p[45-64]
p[47-66]
p[49-68]
p[51-70]
p[53-72]
p[55-74]
p[57-76]
p[59-78]
p[61-80]
p[63-82]
p[65-84]
p[67-86]
p[69-88]
p[71-90]
p[73-92]
p[75-94]
p[77-96]
p[79-98]
p[81-100]
p[83-102]
p[85-104]
p[87-106]
p[89-108]
p[91-110]
p[93-112]
p[95-114]
p[97-116]
p[99-118]
p[101-120]
p[103-122]
p[105-124]
p[107-126]
p[109-128]
p[111-130]
p[113-132]
p[115-134]
p[117-136]
p[119-138]
p[120-139]

B

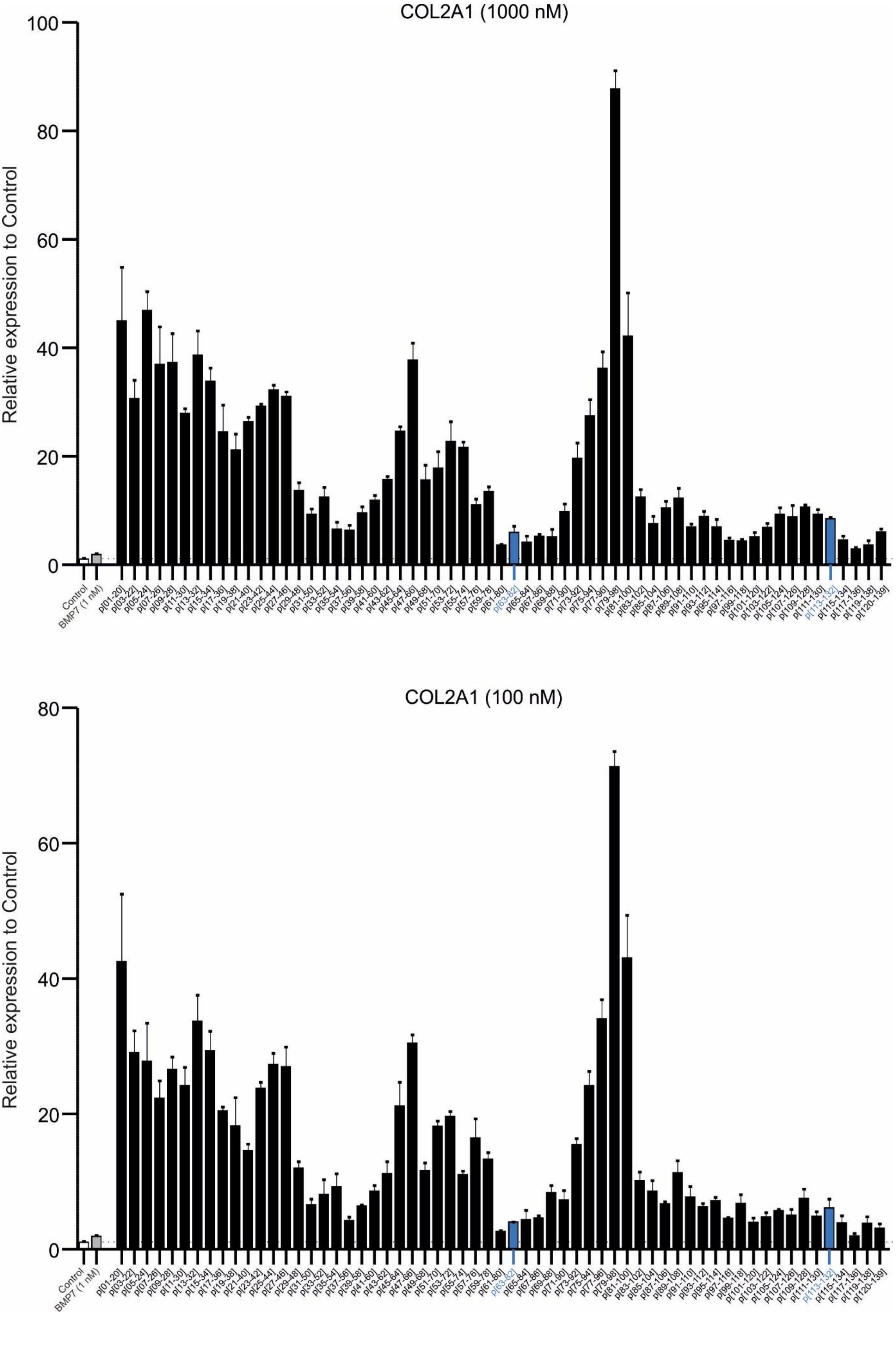
COL2A1 (1000 nM)
Relative expression to Control
100
80
60
40
20
0
COL2A1 (100 nM)
Relative expression to Control
80
60
40
20
0
Control
BMP7 (1 nM)

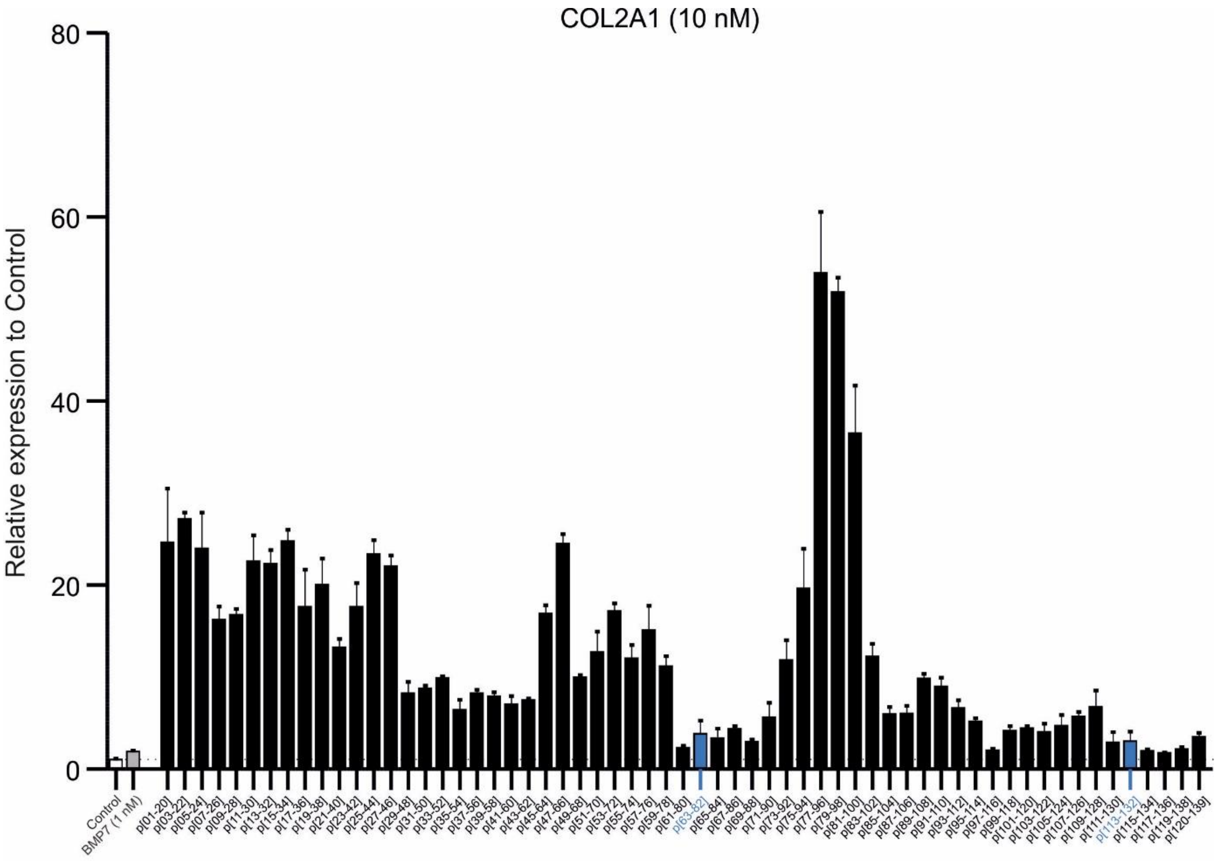
COL2A1 (10 nM)
Relative expression to Control
80
60
40
20
0
Control
BMP7 (1 nM)
p[01-20]
p[03-22]
p[05-24]
p[07-26]
p[09-28]
p[11-30]
p[13-32]
p[15-34]
p[17-36]
p[19-38]
p[21-40]
p[23-42]
p[25-44]
p[27-46]
p[29-48]
p[31-50]
p[33-52]
p[35-54]
p[37-56]
p[39-58]
p[41-60]
p[43-62]
p[45-64]
p[47-66]
p[49-68]
p[51-70]
p[53-72]
p[55-74]
p[57-76]
p[59-78]
p[61-80]
p[63-82]
p[65-84]
p[67-86]
p[69-88]
p[71-90]
p[73-92]
p[75-94]
p[77-96]
p[79-98]
p[81-100]
p[83-102]
p[85-104]
p[87-106]
p[89-108]
p[91-110]
p[93-112]
p[95-114]
p[97-116]
p[99-118]
p[101-120]
p[103-122]
p[105-124]
p[107-126]
p[109-128]
p[111-130]
p[113-132]
p[115-134]
p[117-136]
p[119-138]
p[120-139]

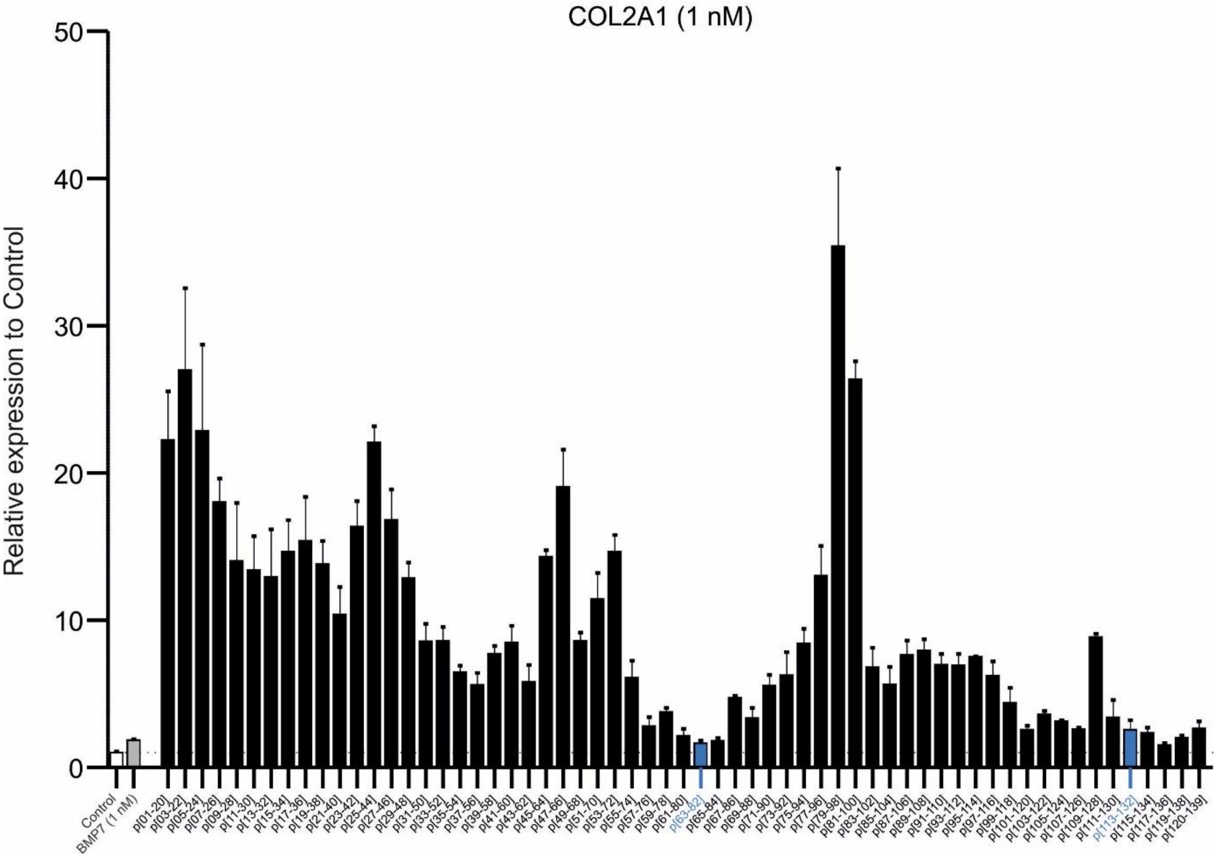
COL2A1 (1 nM)
Relative expression to Control
50
40
30
20
10
0
Control
BMP7 (1 nM)
p[01-20]
p[03-22]
p[05-24]
p[07-26]
p[09-28]
p[11-30]
p[13-32]
p[15-34]
p[17-36]
p[19-38]
p[21-40]
p[23-42]
p[25-44]
p[27-46]
p[29-48]
p[31-50]
p[33-52]
p[35-54]
p[37-56]
p[39-58]
p[41-60]
p[43-62]
p[45-64]
p[47-66]
p[49-68]
p[51-70]
p[53-72]
p[55-74]
p[57-76]
p[59-78]
p[61-80]
p[63-82]
p[65-84]
p[67-86]
p[69-88]
p[71-90]
p[73-92]
p[75-94]
p[77-96]
p[79-98]
p[81-100]
p[83-102]
p[85-104]
p[87-106]
p[89-108]
p[91-110]
p[93-112]
p[95-114]
p[97-116]
p[99-118]
p[101-120]
p[103-122]
p[105-124]
p[107-126]
p[109-128]
p[111-130]
p[113-132]
p[115-134]
p[117-136]
p[119-138]
p[120-139]

C

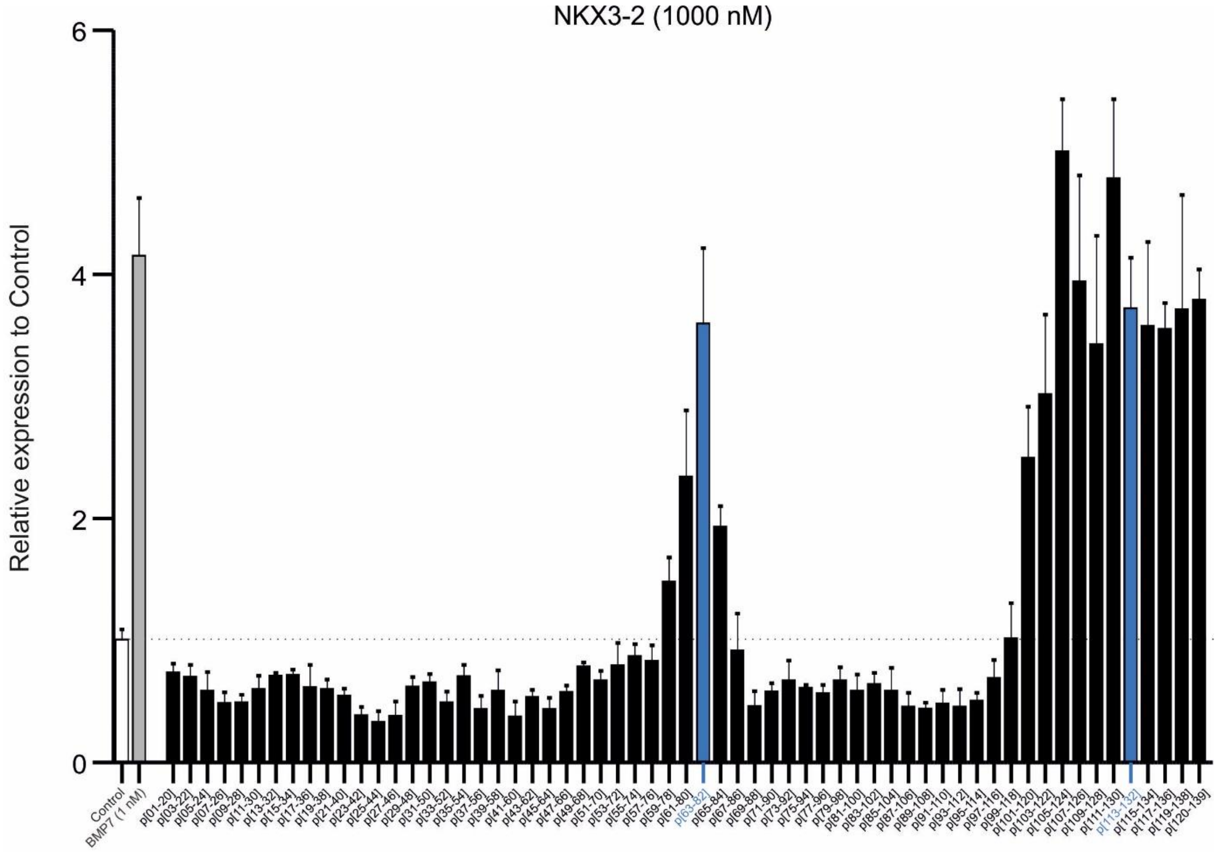
NKX3-2 (1000 nM)
Relative expression to Control
6
4
2
0
Control
BMP7 (1 nM)

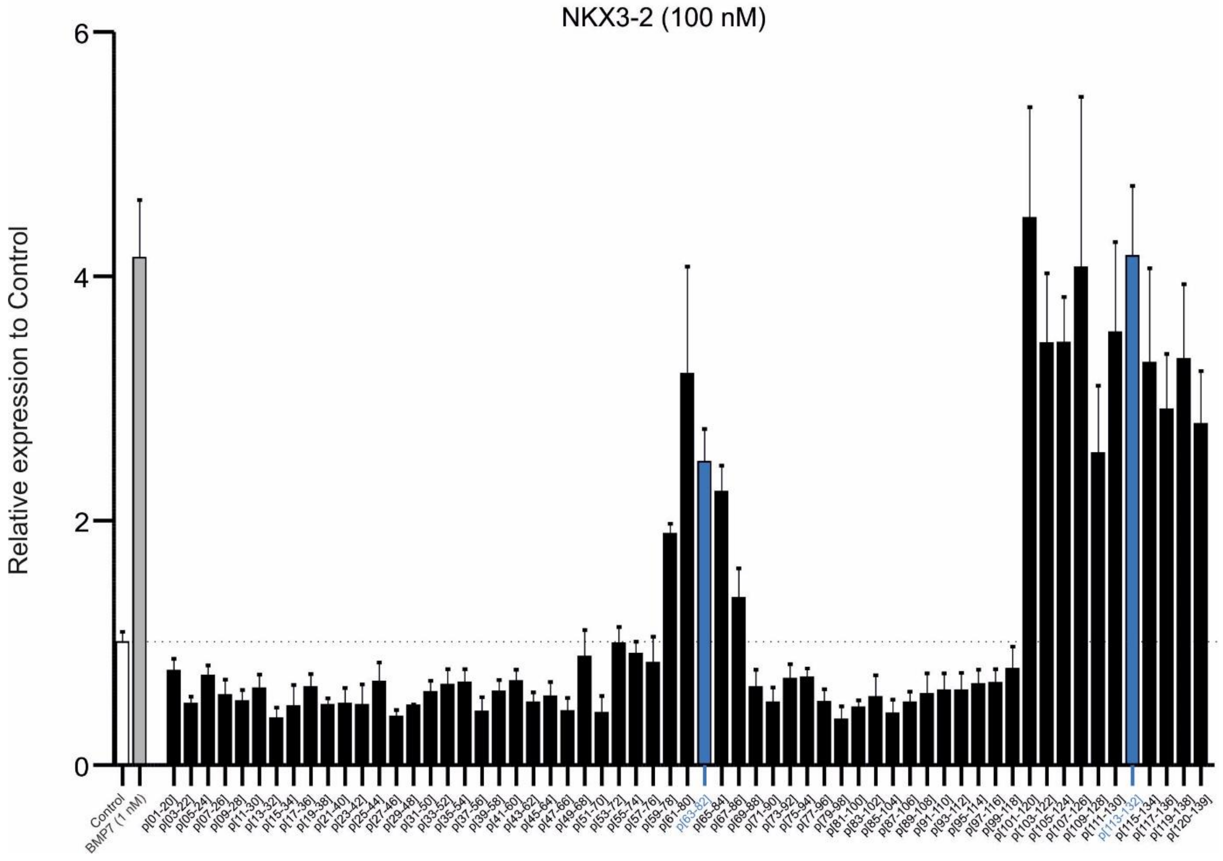
NKX3-2 (100 nM)
Relative expression to Control
6
4
2
0
Control
BMP7 (1 nM)

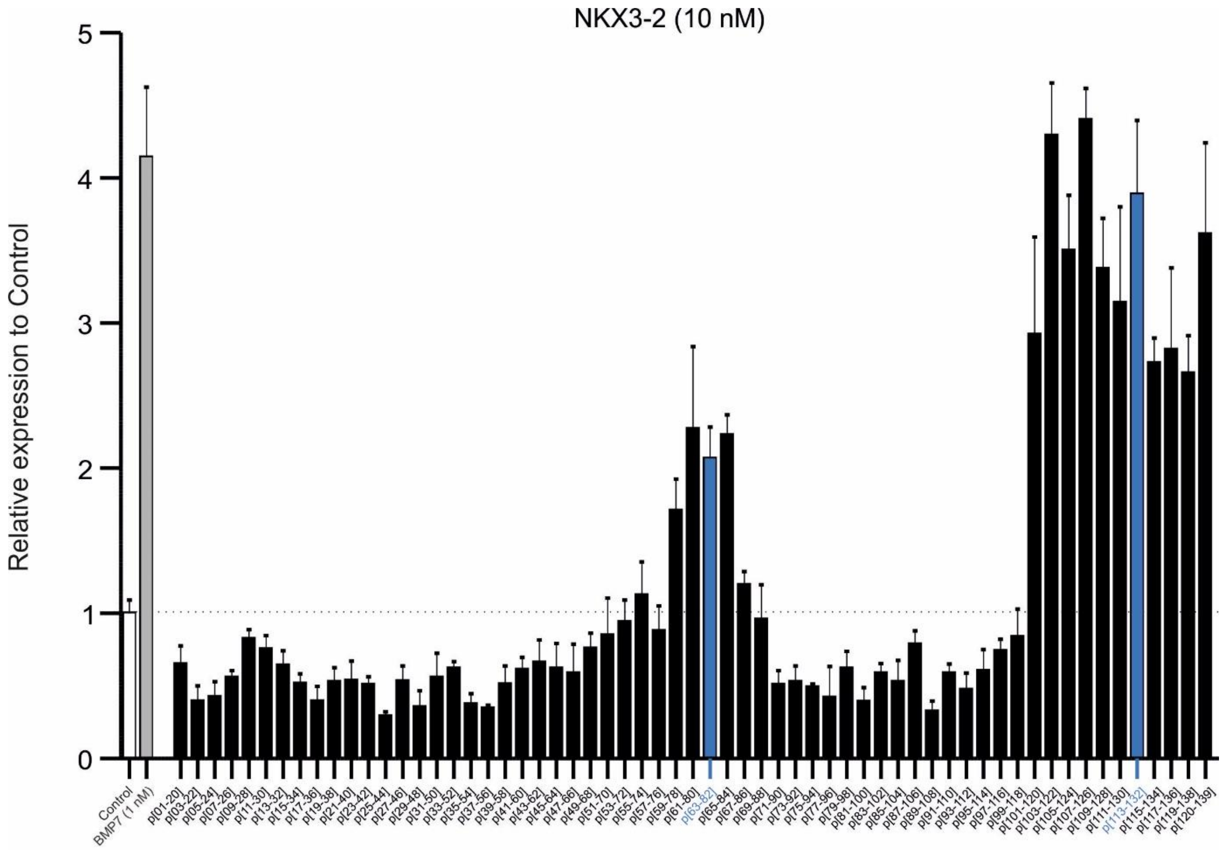
NKX3-2 (10 nM)
Relative expression to Control
0
1
2
3
4
5

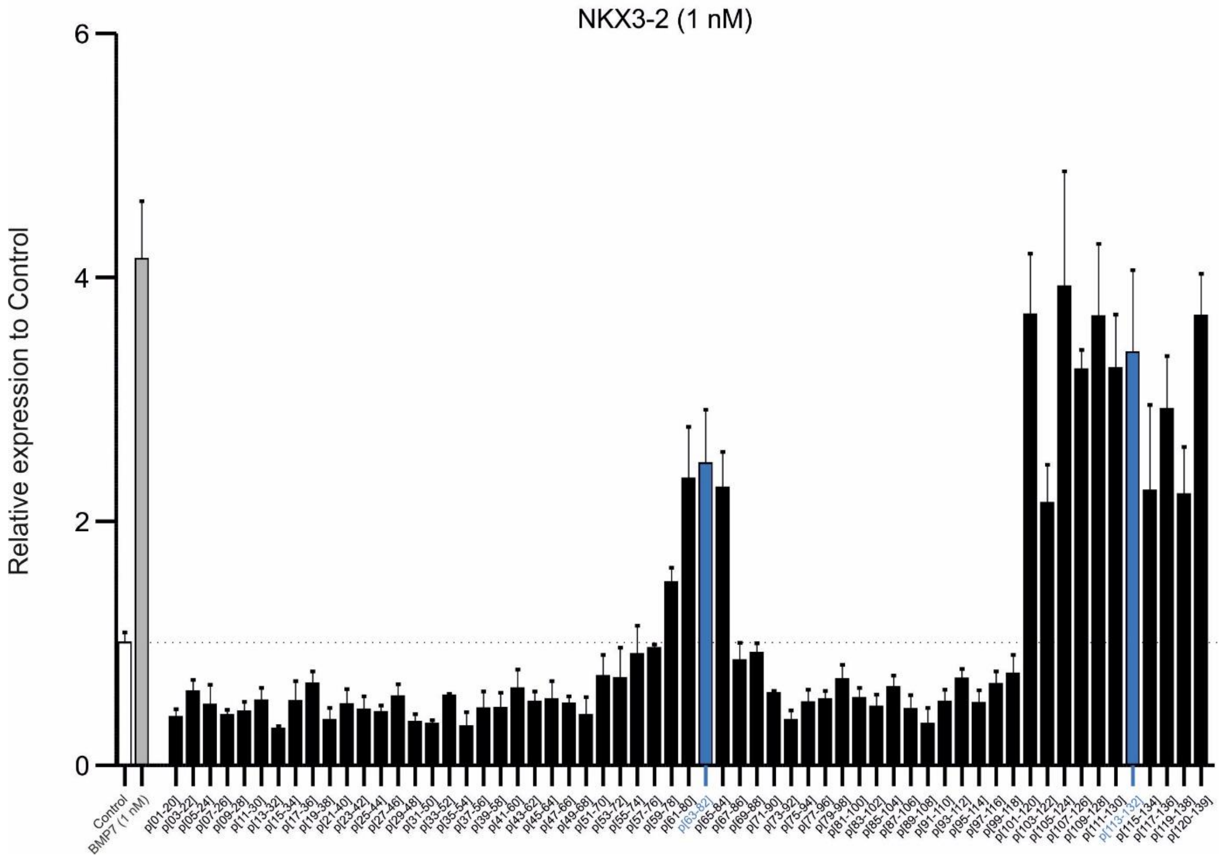
NKX3-2 (1 nM)
Relative expression to Control
0
2
4
6

D

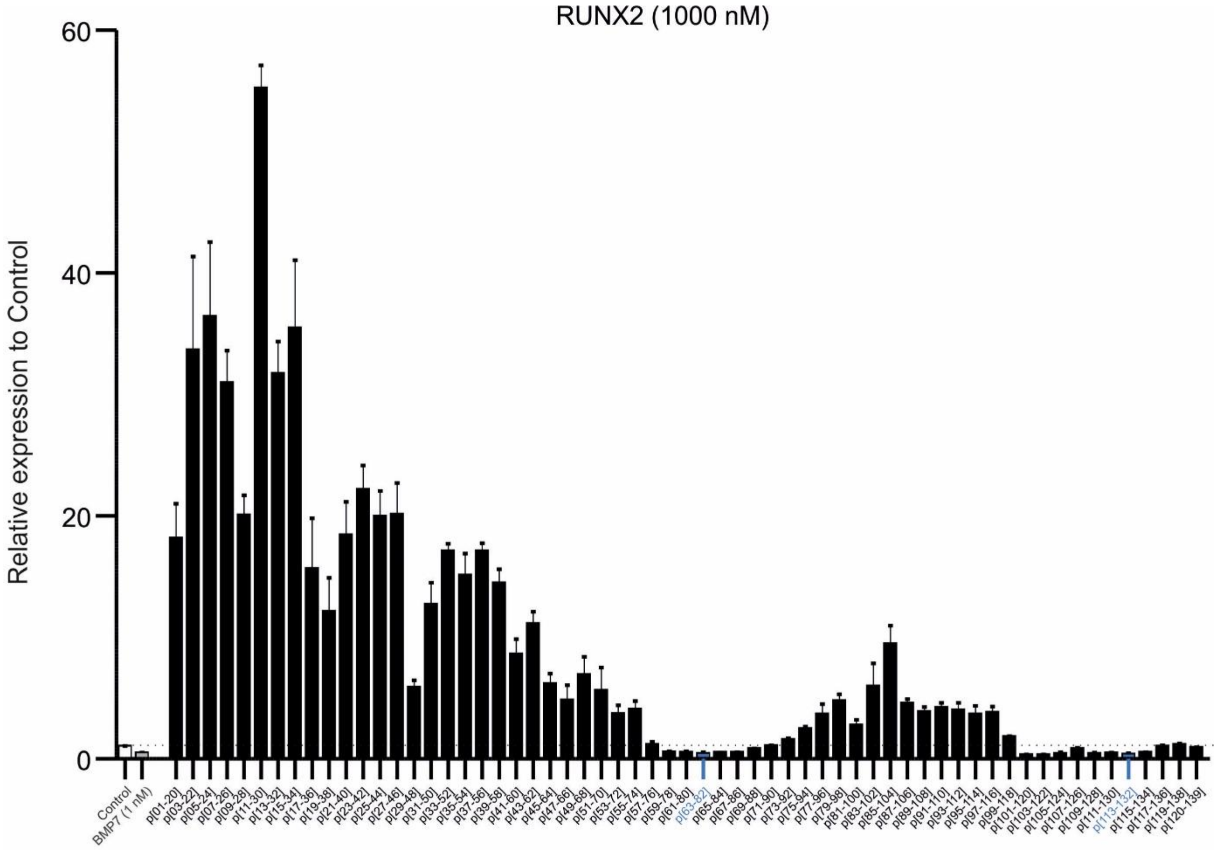

RUNX2 (1000 nM)
Relative expression to Control
60
40
20
0

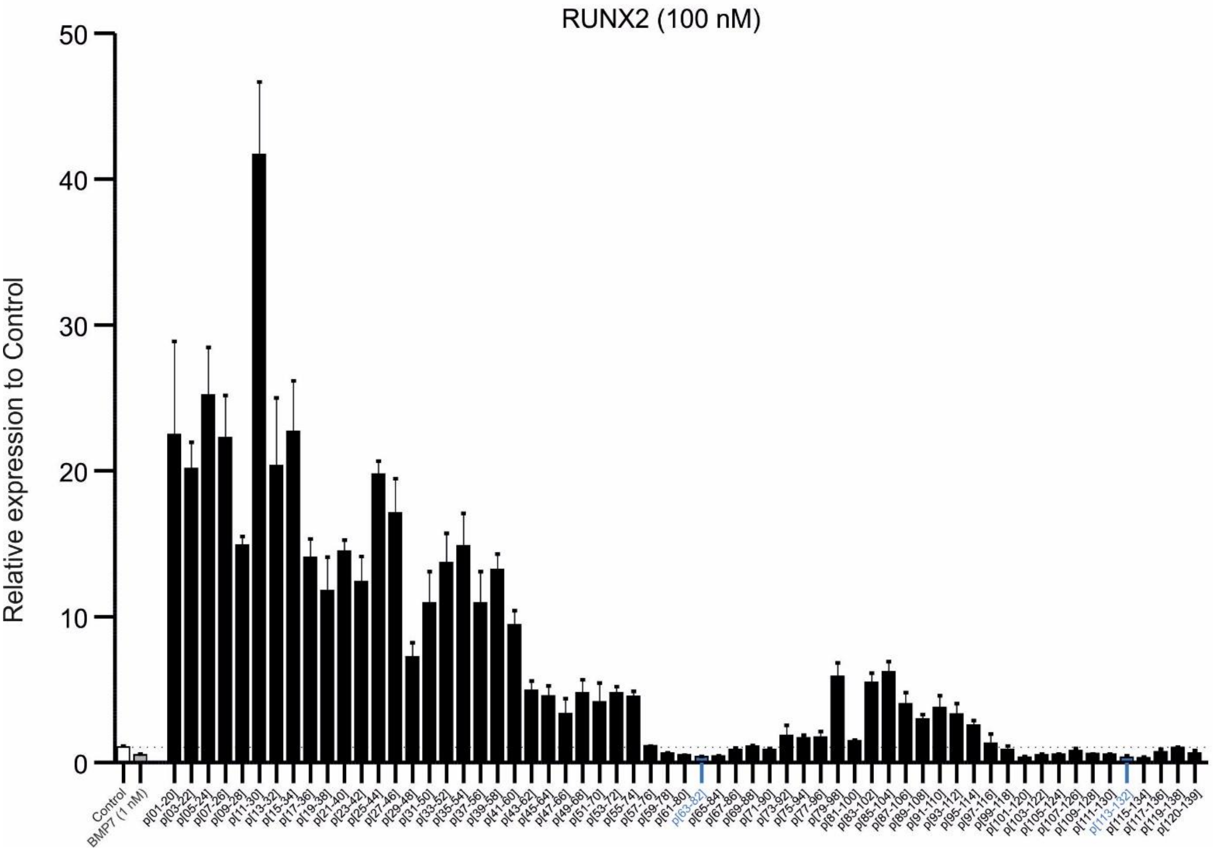

RUNX2 (100 nM)
Relative expression to Control
50
40
30
20
10
0

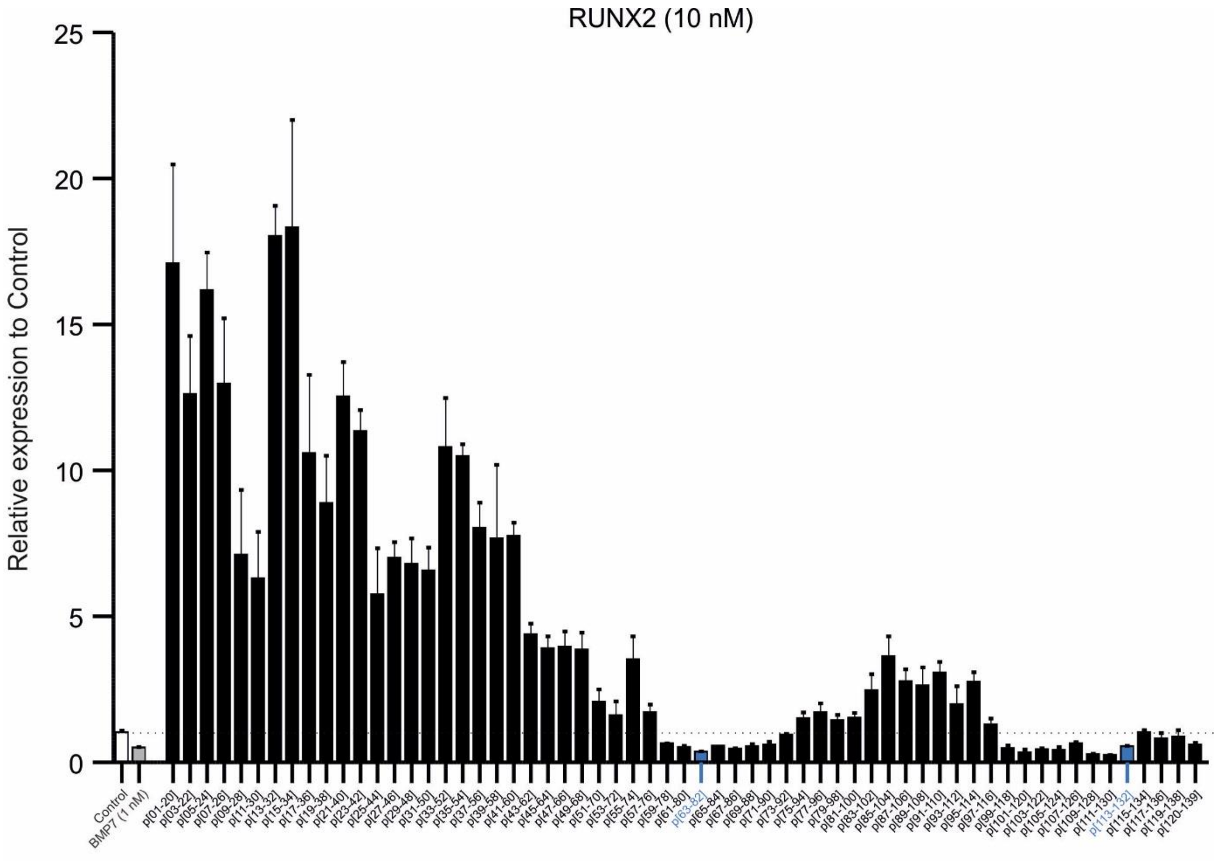
RUNX2 (10 nM)
Relative expression to Control
25
20
15
10
5
0
Control
BMP7 (1 nM)

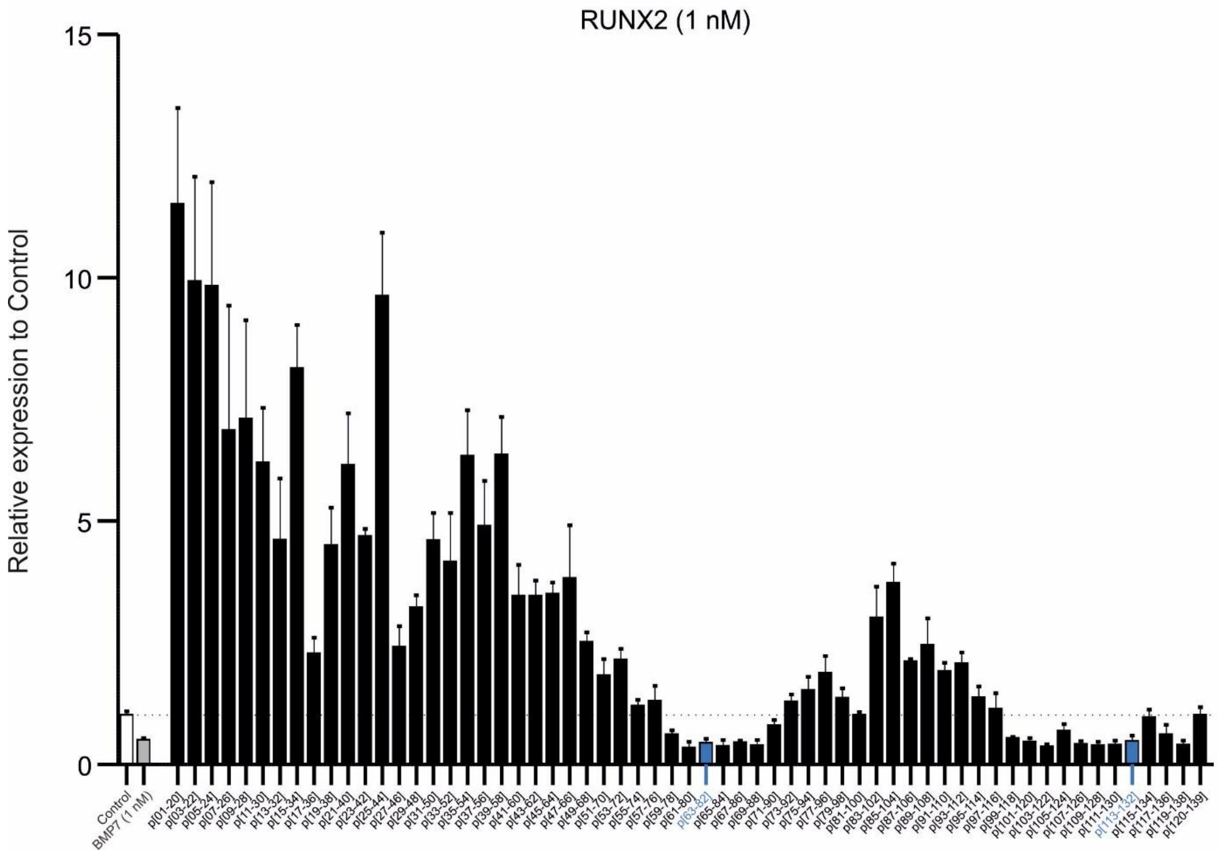
RUNX2 (1 nM)
Relative expression to Control
15
10
5
0
Control
BMP7 (1 nM)

E

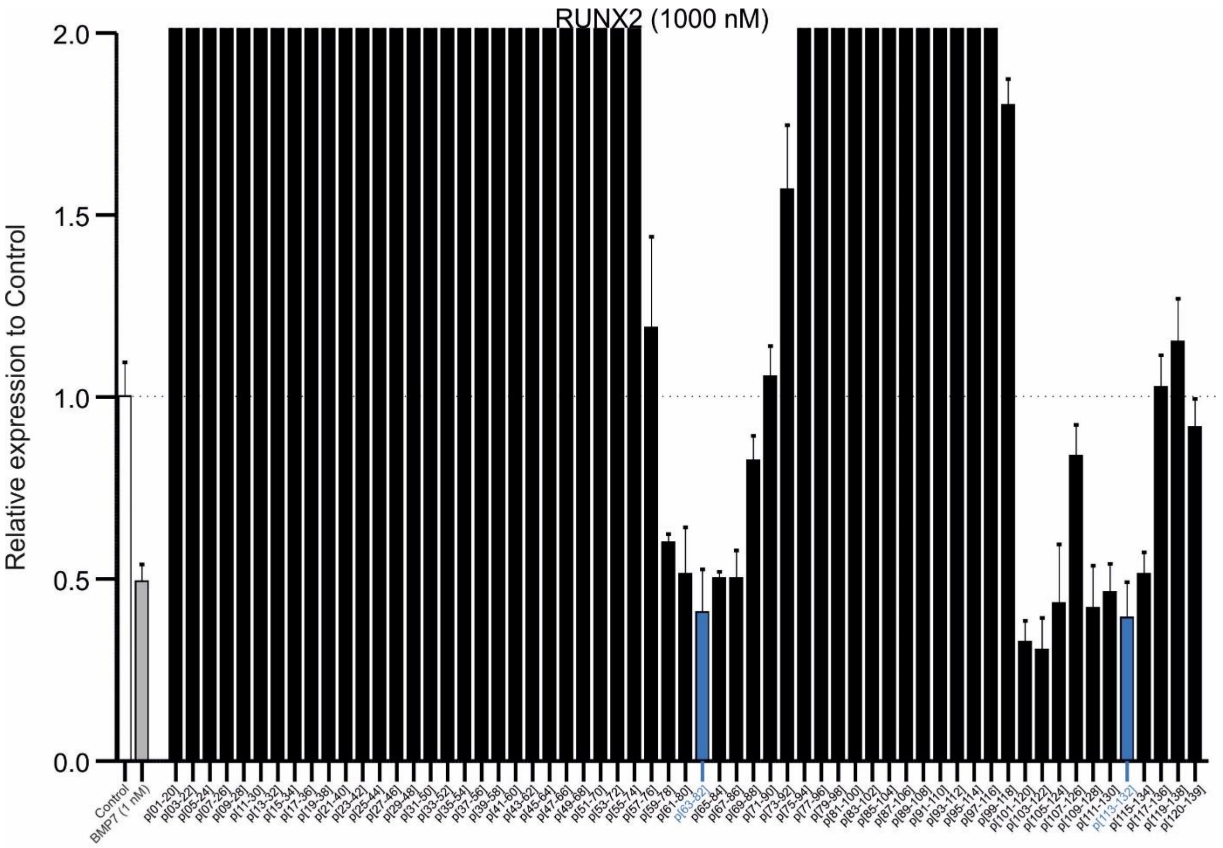
RUNX2 (1000 nM)
Relative expression to Control
2.0
1.5
1.0
0.5
0.0
Control
BMP7 (1 nM)

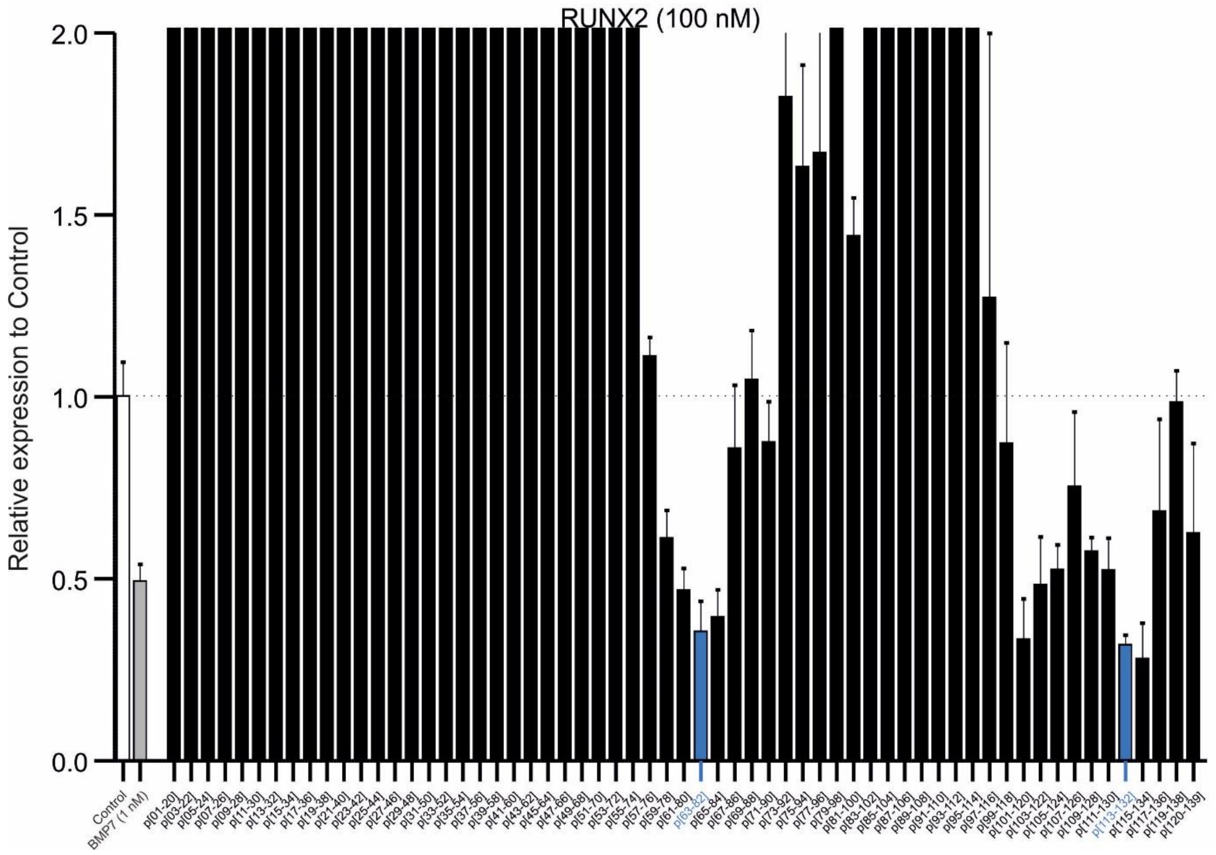
RUNX2 (100 nM)
Relative expression to Control
2.0
1.5
1.0
0.5
0.0
Control
BMP7 (1 nM)

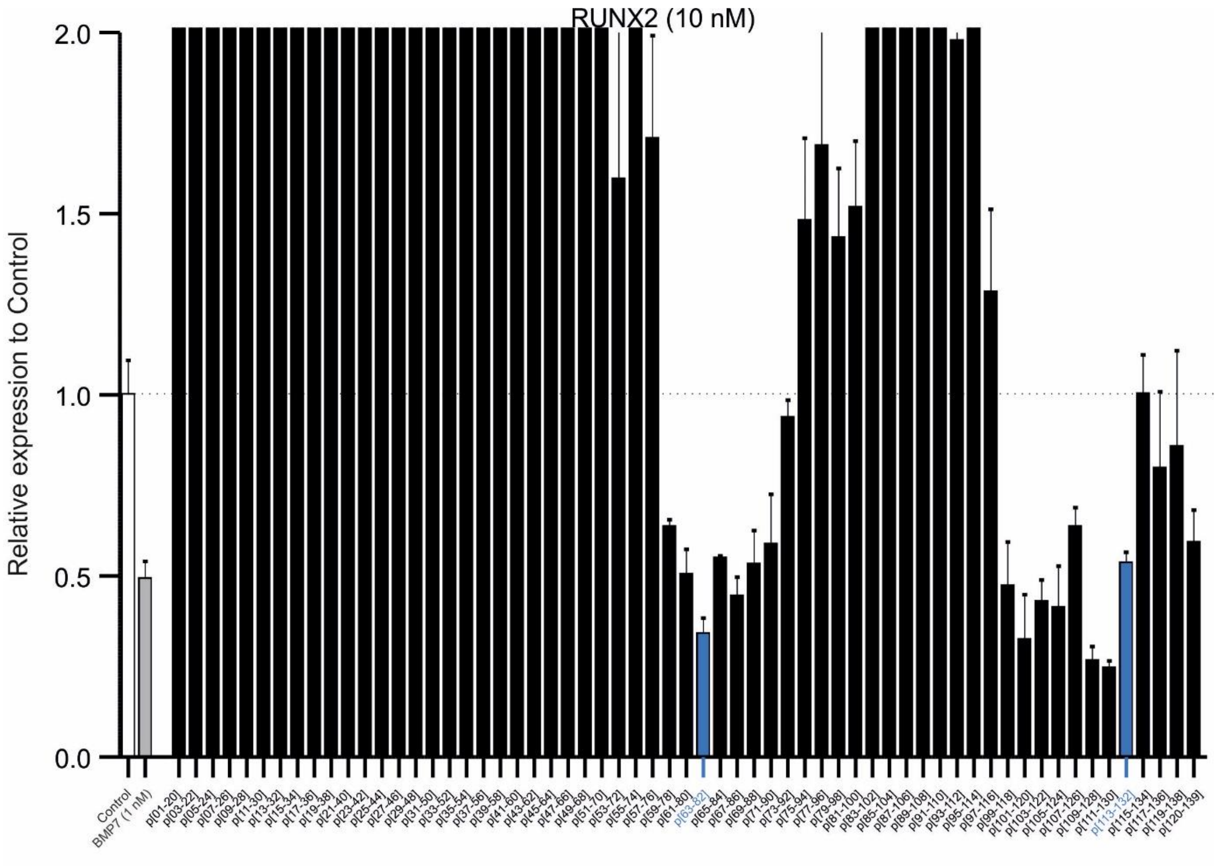
RUNX2 (10 nM)
Relative expression to Control
2.0
1.5
1.0
0.5
0.0
Control
BMP7 (1 nM)
p[01-20]
p[03-22]
p[05-24]
p[07-26]
p[09-28]
p[11-30]
p[13-32]
p[15-34]
p[17-36]
p[19-38]
p[21-40]
p[23-42]
p[25-44]
p[27-46]
p[29-48]
p[31-50]
p[33-52]
p[35-54]
p[37-56]
p[39-58]
p[41-60]
p[43-62]
p[45-64]
p[47-66]
p[49-68]
p[51-70]
p[53-72]
p[55-74]
p[57-76]
p[59-78]
p[61-80]
p[63-82]
p[65-84]
p[67-86]
p[69-88]
p[71-90]
p[73-92]
p[75-94]
p[77-96]
p[79-98]
p[81-100]
p[83-102]
p[85-104]
p[87-106]
p[89-108]
p[91-110]
p[93-112]
p[95-114]
p[97-116]
p[99-118]
p[101-120]
p[103-122]
p[105-124]
p[107-126]
p[109-128]
p[111-130]
p[113-132]
p[115-134]
p[117-136]
p[119-138]
p[120-139]

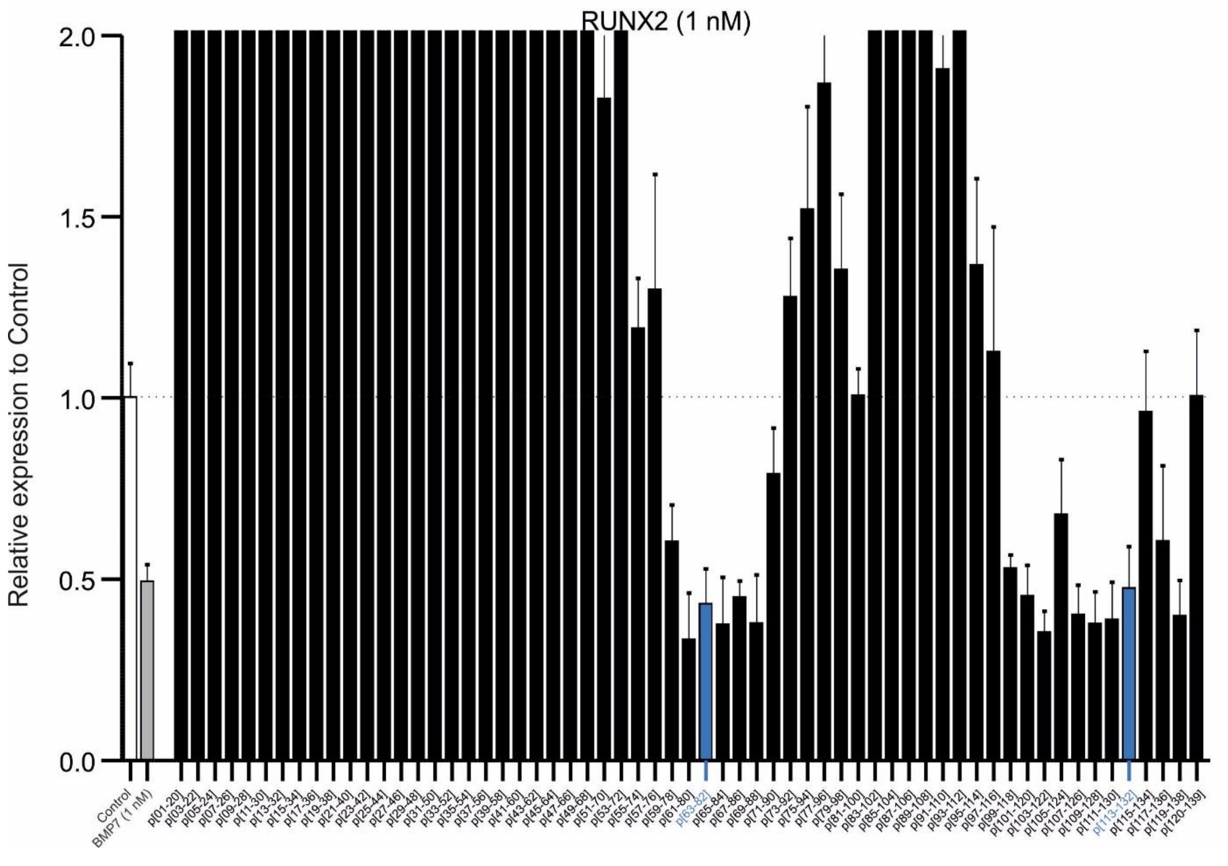
RUNX2 (1 nM)
Relative expression to Control
2.0
1.5
1.0
0.5
0.0
Control
BMP7 (1 nM)
p[01-20]
p[03-22]
p[05-24]
p[07-26]
p[09-28]
p[11-30]
p[13-32]
p[15-34]
p[17-36]
p[19-38]
p[21-40]
p[23-42]
p[25-44]
p[27-46]
p[29-48]
p[31-50]
p[33-52]
p[35-54]
p[37-56]
p[39-58]
p[41-60]
p[43-62]
p[45-64]
p[47-66]
p[49-68]
p[51-70]
p[53-72]
p[55-74]
p[57-76]
p[59-78]
p[61-80]
p[63-82]
p[65-84]
p[67-86]
p[69-88]
p[71-90]
p[73-92]
p[75-94]
p[77-96]
p[79-98]
p[81-100]
p[83-102]
p[85-104]
p[87-106]
p[89-108]
p[91-110]
p[93-112]
p[95-114]
p[97-116]
p[99-118]
p[101-120]
p[103-122]
p[105-124]
p[107-126]
p[109-128]
p[111-130]
p[113-132]
p[115-134]
p[117-136]
p[119-138]
p[120-139]

F

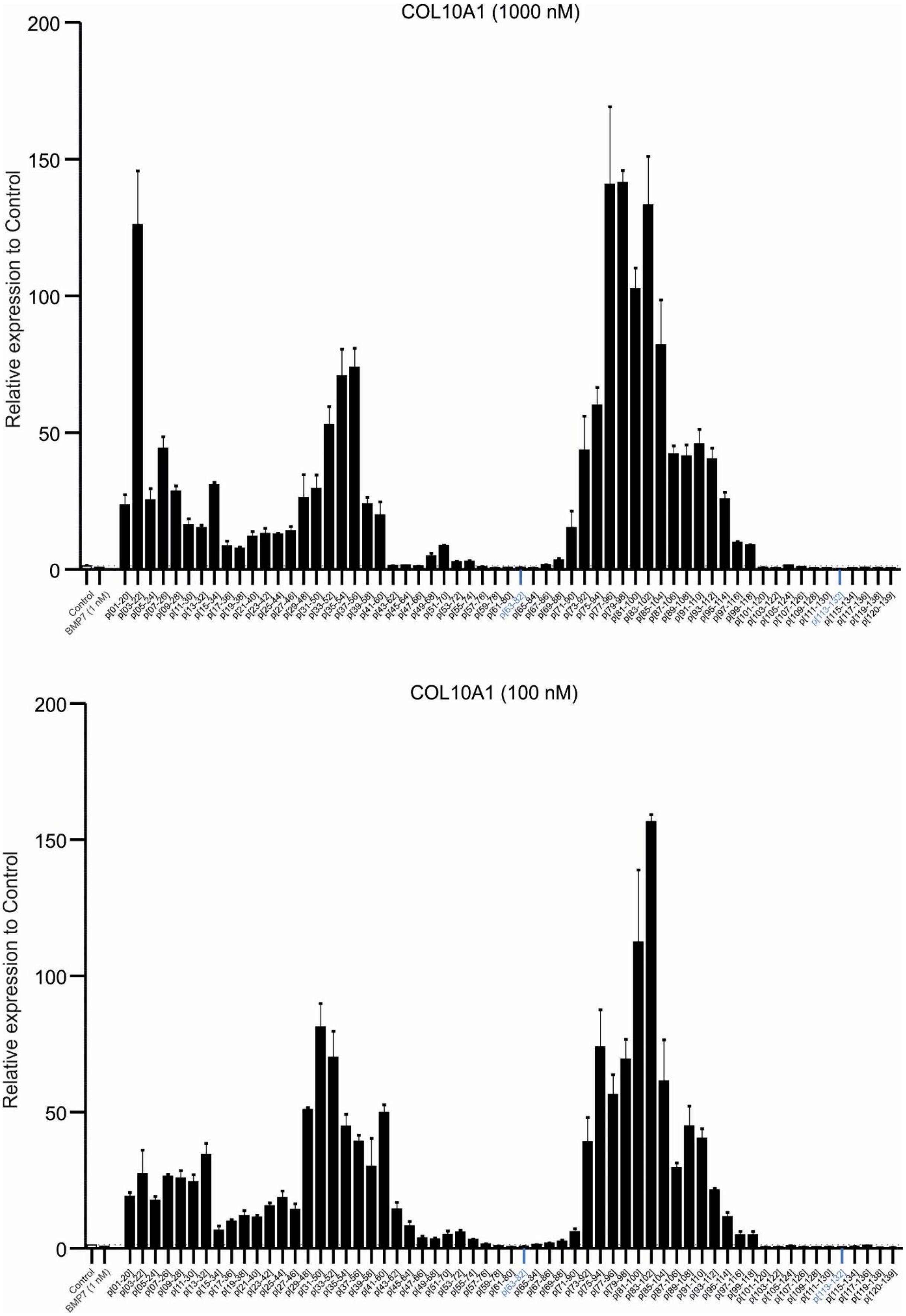
COL10A1 (1000 nM)
Relative expression to Control
200
150
100
50
0
Control
BMP7 (1 nM)
COL10A1 (100 nM)
Relative expression to Control
200
150
100
50
0
Control
BMP7 (1 nM)

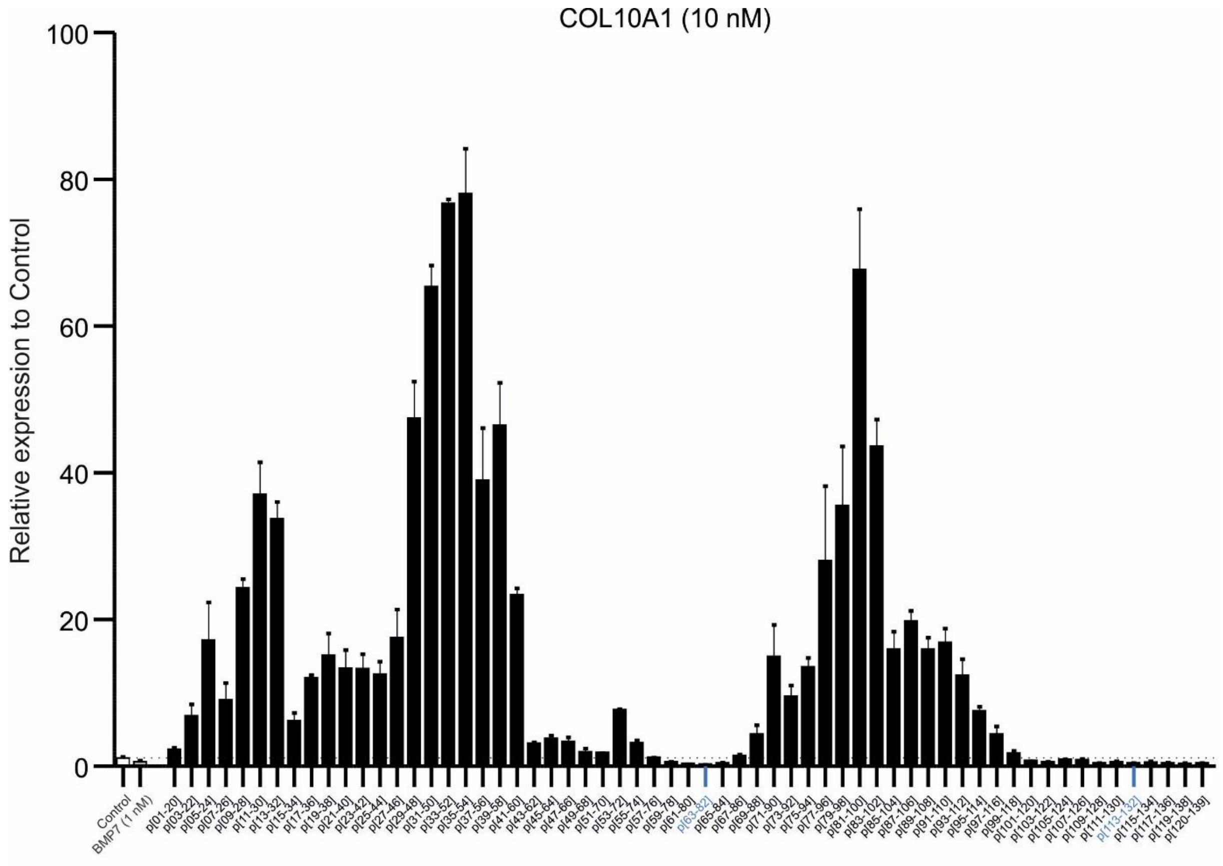
COL10A1 (10 nM)
Relative expression to Control
0
20
40
60
80
100
Control
BMP7 (1 nM)
p[01-20] p[03-22] p[05-24] p[07-26] p[09-28] p[11-30] p[13-32] p[15-34] p[17-36] p[19-38] p[21-40] p[23-42] p[25-44] p[27-46] p[29-48] p[31-50] p[33-52] p[35-54] p[37-56] p[39-58] p[41-60] p[43-62] p[45-64] p[47-66] p[49-68] p[51-70] p[53-72] p[55-74] p[57-76] p[59-78] p[61-80] p[63-82] p[65-84] p[67-86] p[69-88] p[71-90] p[73-92] p[75-94] p[77-96] p[79-98] p[81-100] p[83-102] p[85-104] p[87-106] p[89-108] p[91-110] p[93-112] p[95-114] p[97-116] p[99-118] p[101-120] p[103-122] p[105-124] p[107-126] p[109-128] p[111-130] p[113-132] p[115-134] p[117-136] p[119-138] p[120-139]

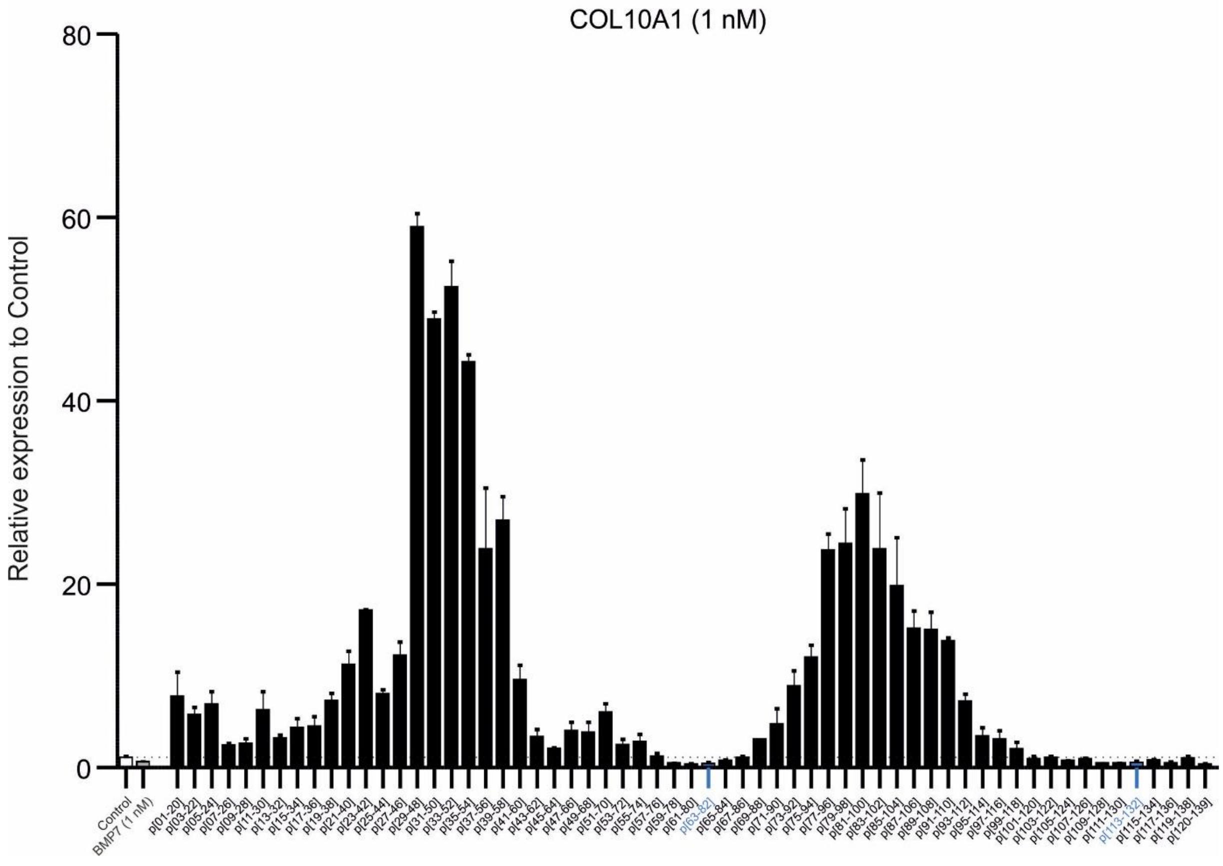
COL10A1 (1 nM)
Relative expression to Control
0
20
40
60
80
Control
BMP7 (1 nM)
p[01-20] p[03-22] p[05-24] p[07-26] p[09-28] p[11-30] p[13-32] p[15-34] p[17-36] p[19-38] p[21-40] p[23-42] p[25-44] p[27-46] p[29-48] p[31-50] p[33-52] p[35-54] p[37-56] p[39-58] p[41-60] p[43-62] p[45-64] p[47-66] p[49-68] p[51-70] p[53-72] p[55-74] p[57-76] p[59-78] p[61-80] p[63-82] p[65-84] p[67-86] p[69-88] p[71-90] p[73-92] p[75-94] p[77-96] p[79-98] p[81-100] p[83-102] p[85-104] p[87-106] p[89-108] p[91-110] p[93-112] p[95-114] p[97-116] p[99-118] p[101-120] p[103-122] p[105-124] p[107-126] p[109-128] p[111-130] p[113-132] p[115-134] p[117-136] p[119-138] p[120-139]

G

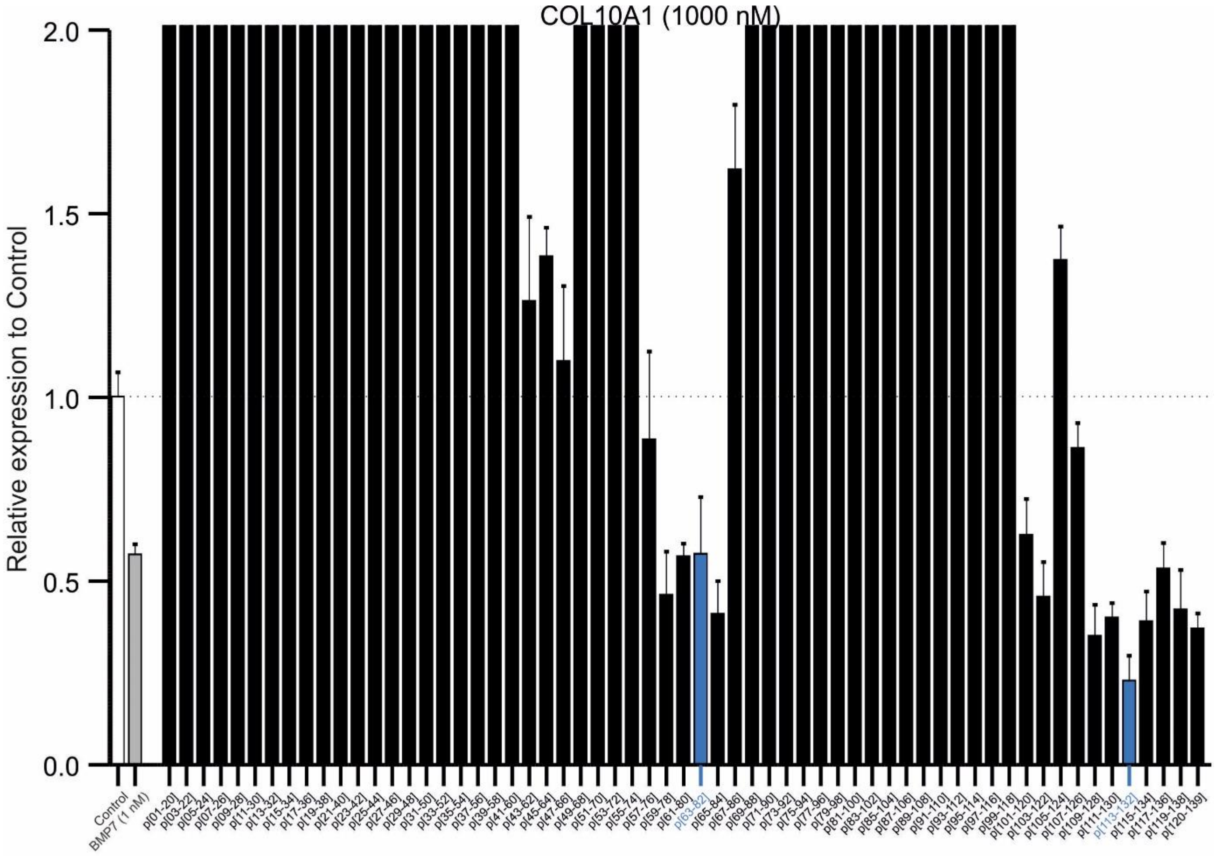
COL10A1 (1000 nM)
Relative expression to Control
2.0
1.5
1.0
0.5
0.0
Control
BMP7 (1 nM)

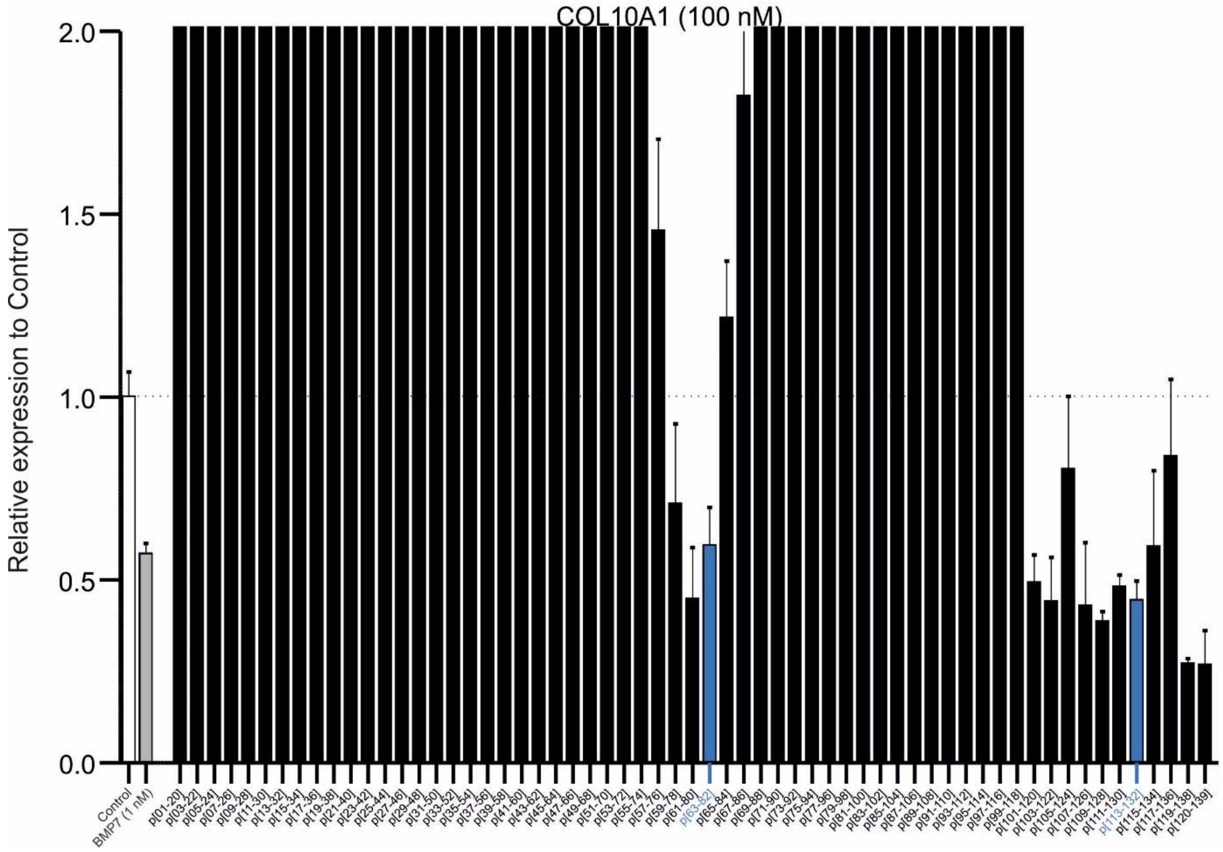
COL10A1 (100 nM)
Relative expression to Control
2.0
1.5
1.0
0.5
0.0
Control
BMP7 (1 nM)

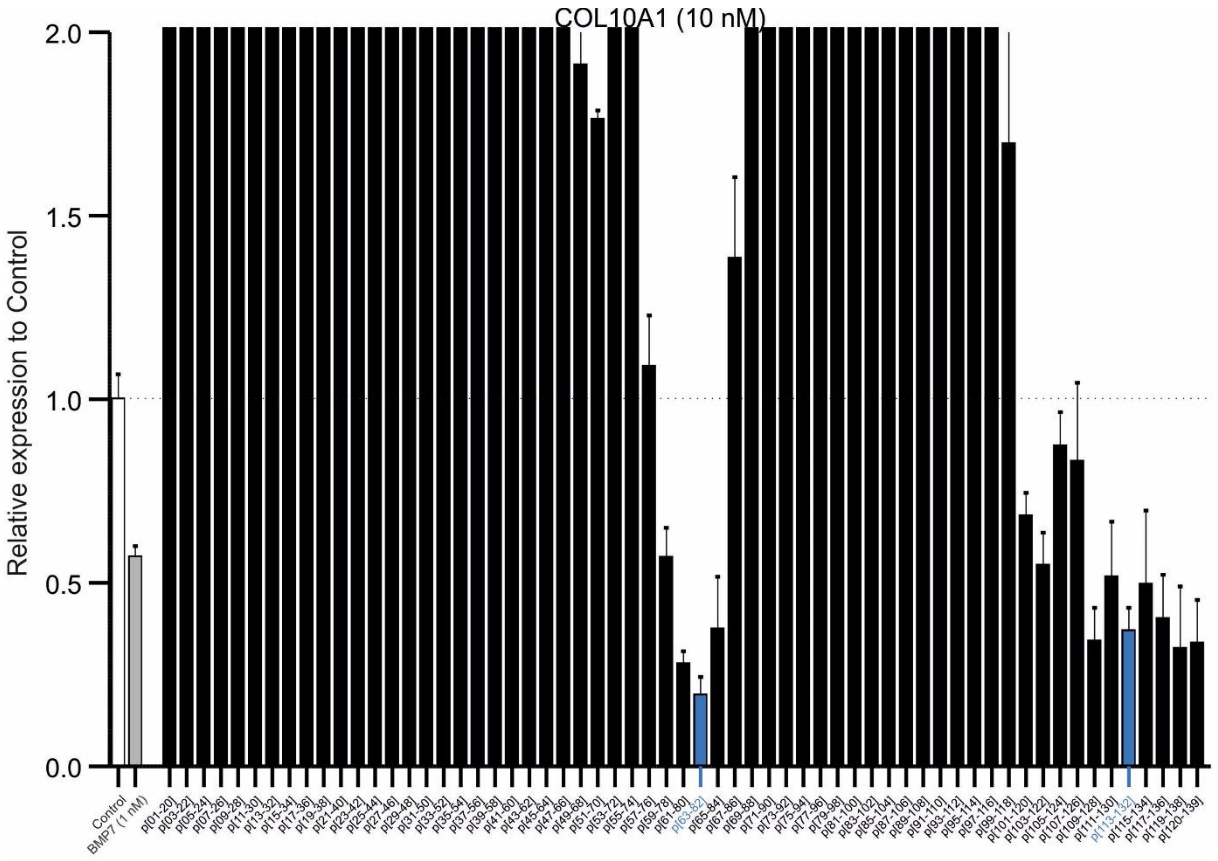
COL10A1 (10 nM)
Relative expression to Control
2.0
1.5
1.0
0.5
0.0
Control
BMP7 (1 nM)

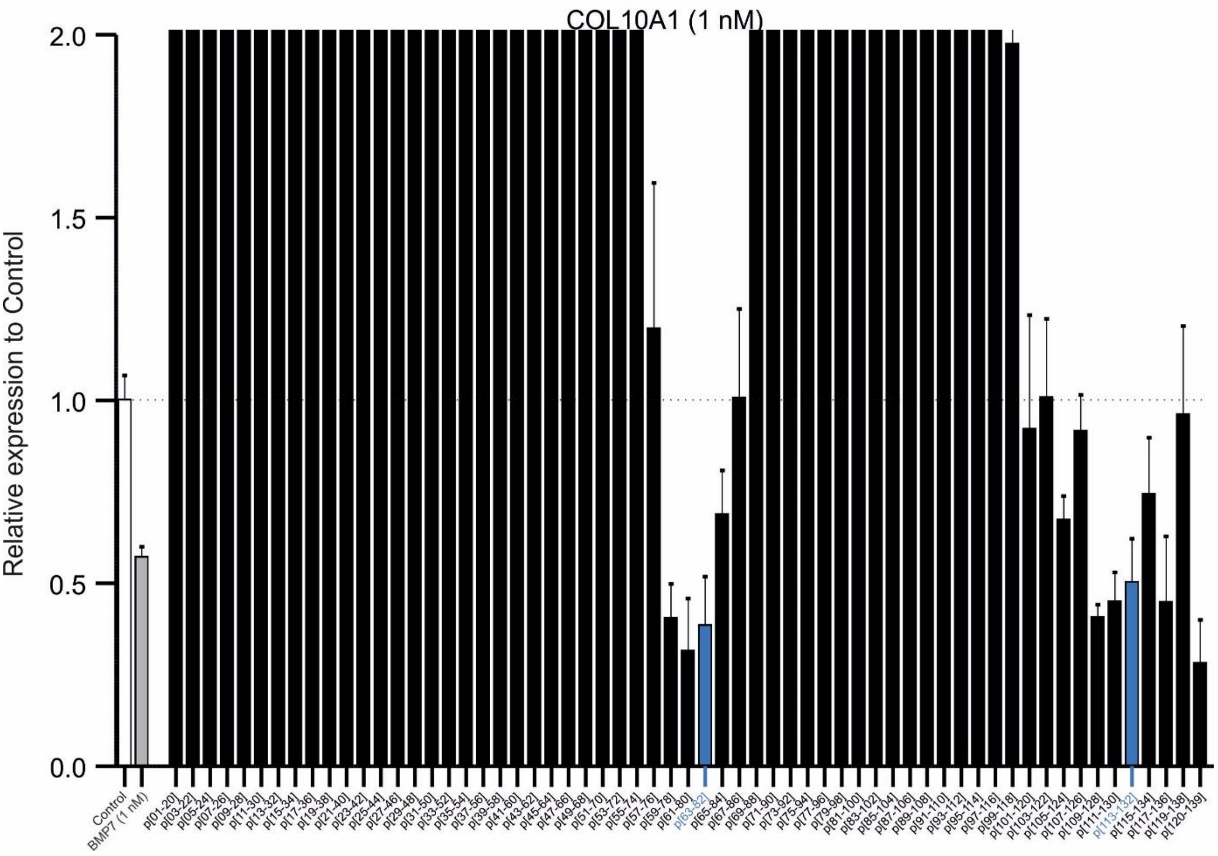
COL10A1 (1 nM)
Relative expression to Control
2.0
1.5
1.0
0.5
0.0
Control
BMP7 (1 nM)

H

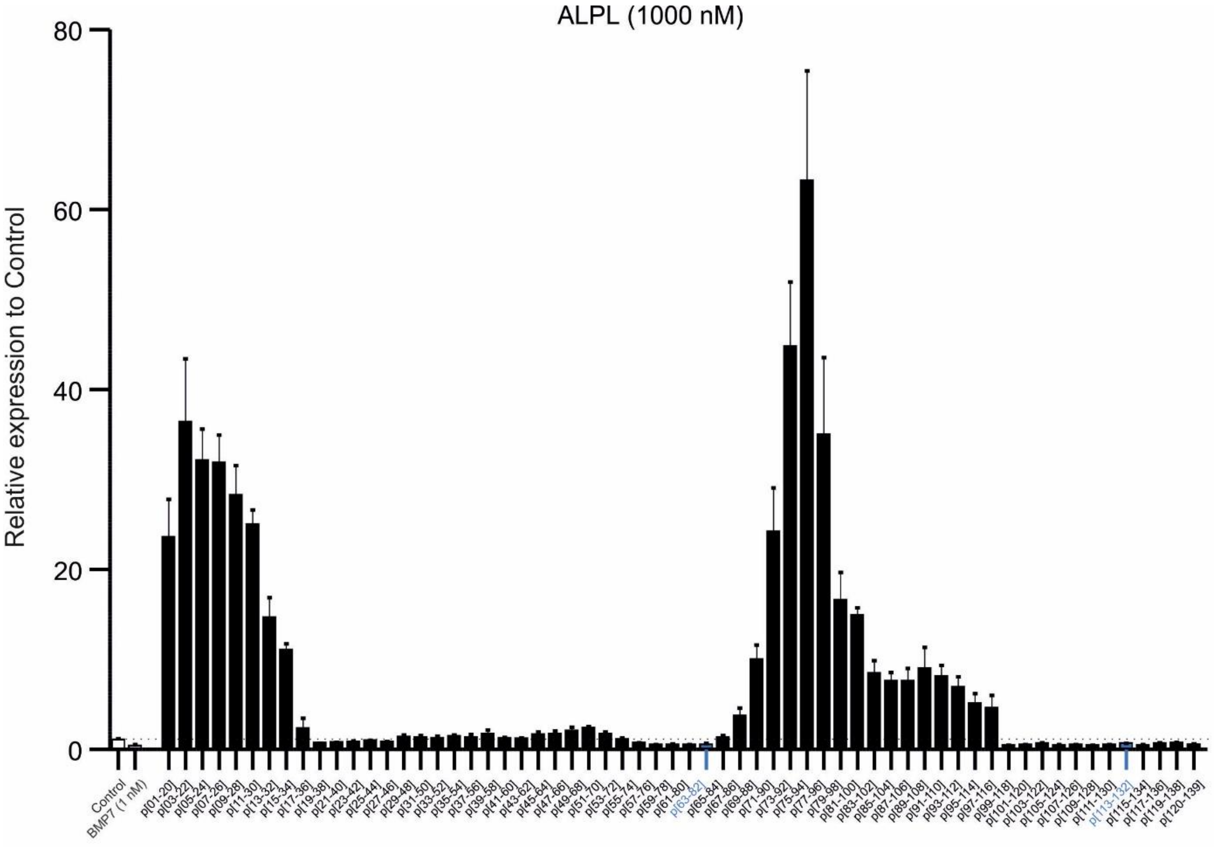
ALPL (1000 nM)
Relative expression to Control
80
60
40
20
0
Control
BMP7 (1 nM)

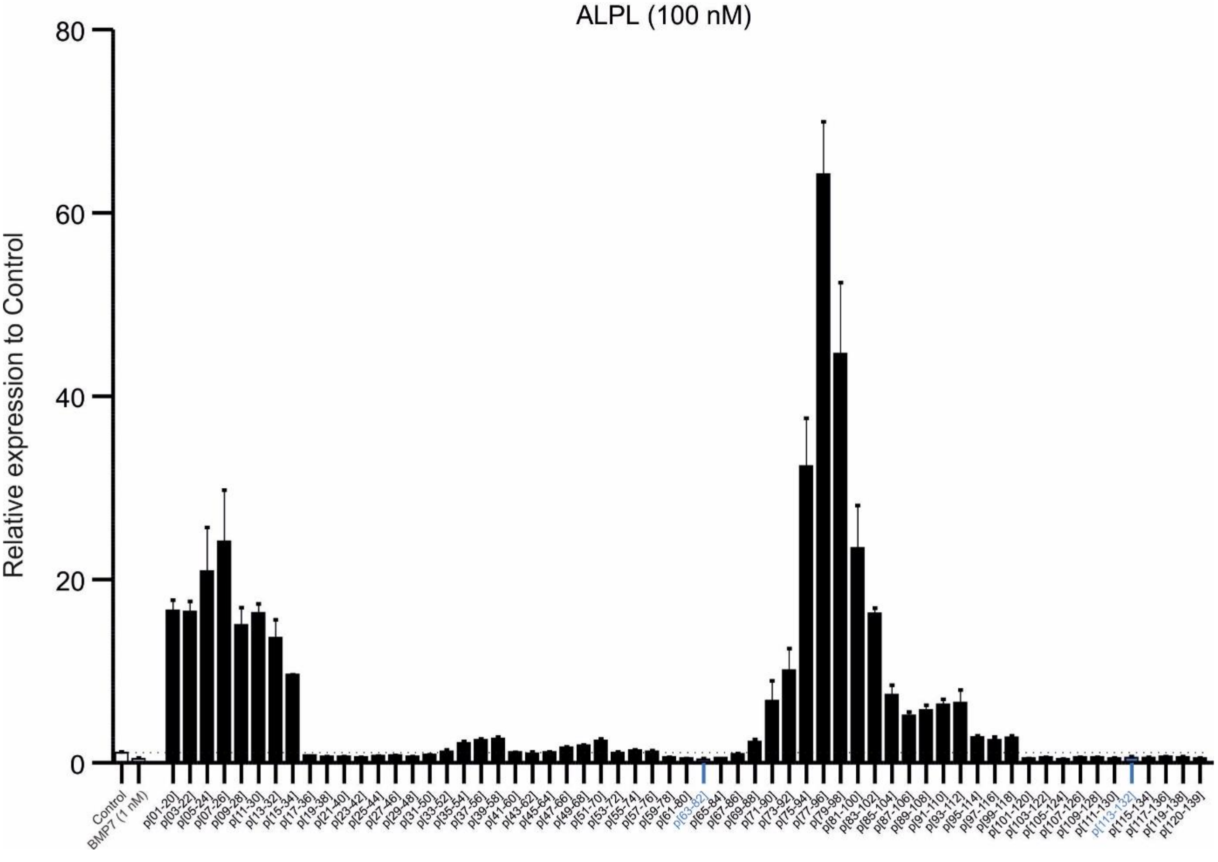
ALPL (100 nM)
Relative expression to Control
80
60
40
20
0
Control
BMP7 (1 nM)

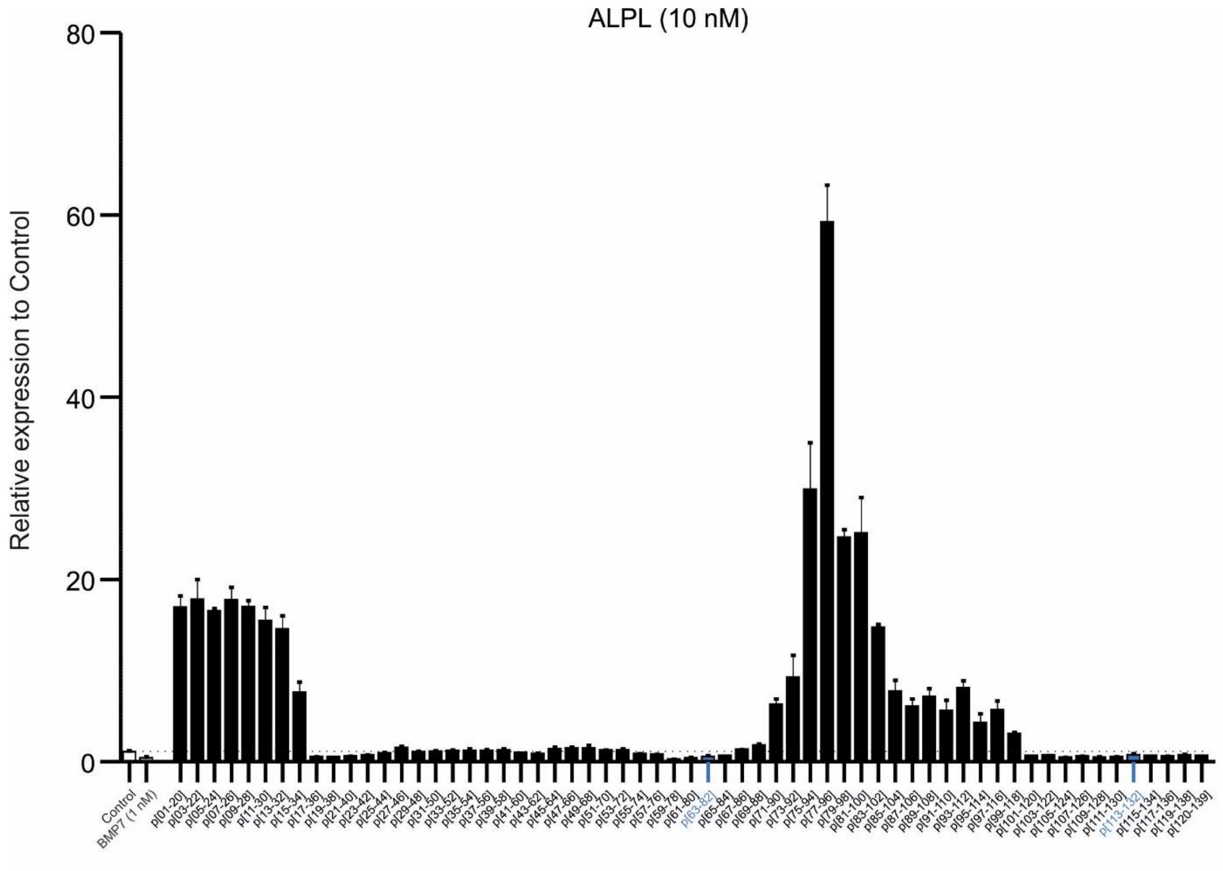
ALPL (10 nM)
Relative expression to Control
80
60
40
20
0
Control
BMP7 (1 nM)
p[01-20] p[03-22] p[05-24] p[07-26] p[09-28] p[11-30] p[13-32] p[15-34] p[17-36] p[19-38] p[21-40] p[23-42] p[25-44] p[27-46] p[29-48] p[31-50] p[33-52] p[35-54] p[37-56] p[39-58] p[41-60] p[43-62] p[45-64] p[47-66] p[49-68] p[51-70] p[53-72] p[55-74] p[57-76] p[59-78] p[61-80] p[63-82] p[65-84] p[67-86] p[69-88] p[71-90] p[73-92] p[75-94] p[77-96] p[79-98] p[81-100] p[83-102] p[85-104] p[87-106] p[89-108] p[91-110] p[93-112] p[95-114] p[97-116] p[99-118] p[101-120] p[103-122] p[105-124] p[107-126] p[109-128] p[111-130] p[113-132] p[115-134] p[117-136] p[119-138] p[120-139]

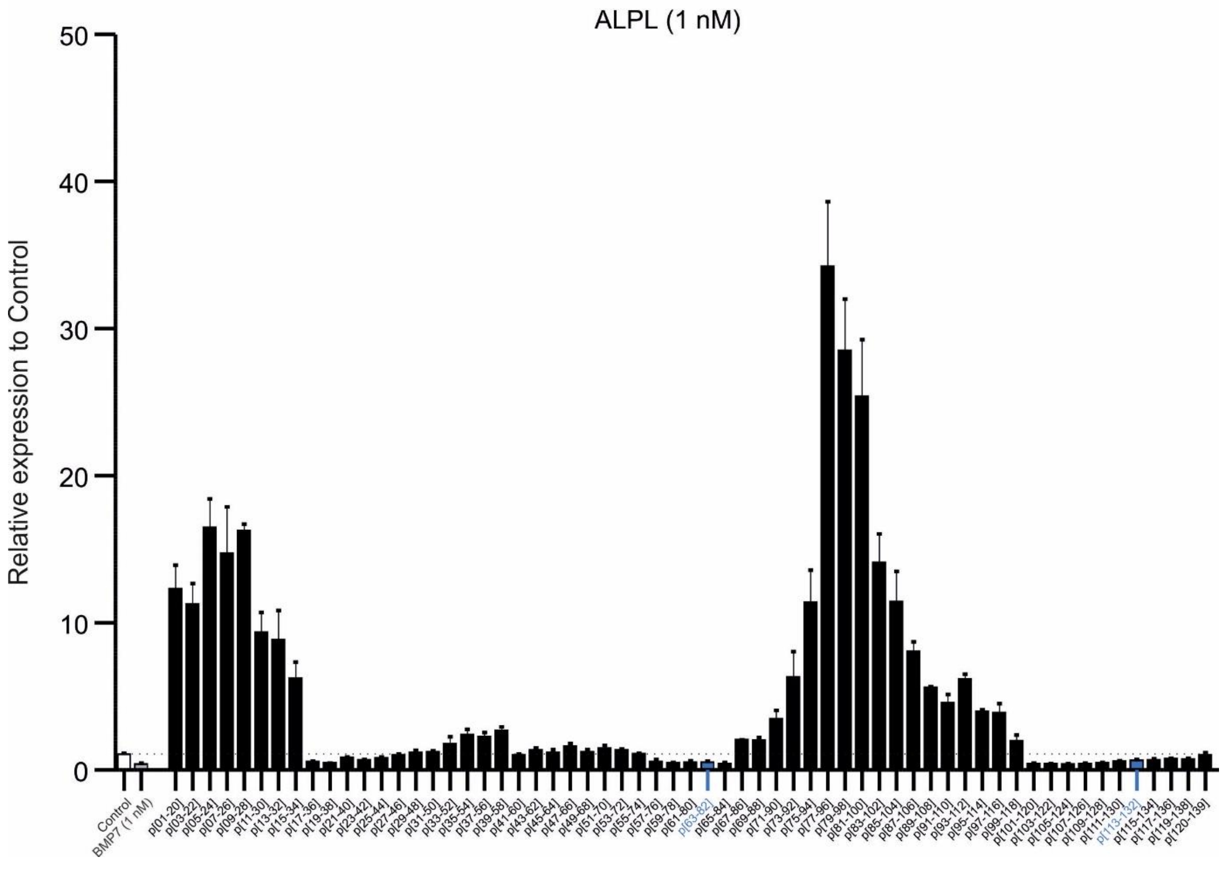
ALPL (1 nM)
Relative expression to Control
50
40
30
20
10
0
Control
BMP7 (1 nM)
p[01-20] p[03-22] p[05-24] p[07-26] p[09-28] p[11-30] p[13-32] p[15-34] p[17-36] p[19-38] p[21-40] p[23-42] p[25-44] p[27-46] p[29-48] p[31-50] p[33-52] p[35-54] p[37-56] p[39-58] p[41-60] p[43-62] p[45-64] p[47-66] p[49-68] p[51-70] p[53-72] p[55-74] p[57-76] p[59-78] p[61-80] p[63-82] p[65-84] p[67-86] p[69-88] p[71-90] p[73-92] p[75-94] p[77-96] p[79-98] p[81-100] p[83-102] p[85-104] p[87-106] p[89-108] p[91-110] p[93-112] p[95-114] p[97-116] p[99-118] p[101-120] p[103-122] p[105-124] p[107-126] p[109-128] p[111-130] p[113-132] p[115-134] p[117-136] p[119-138] p[120-139]

I

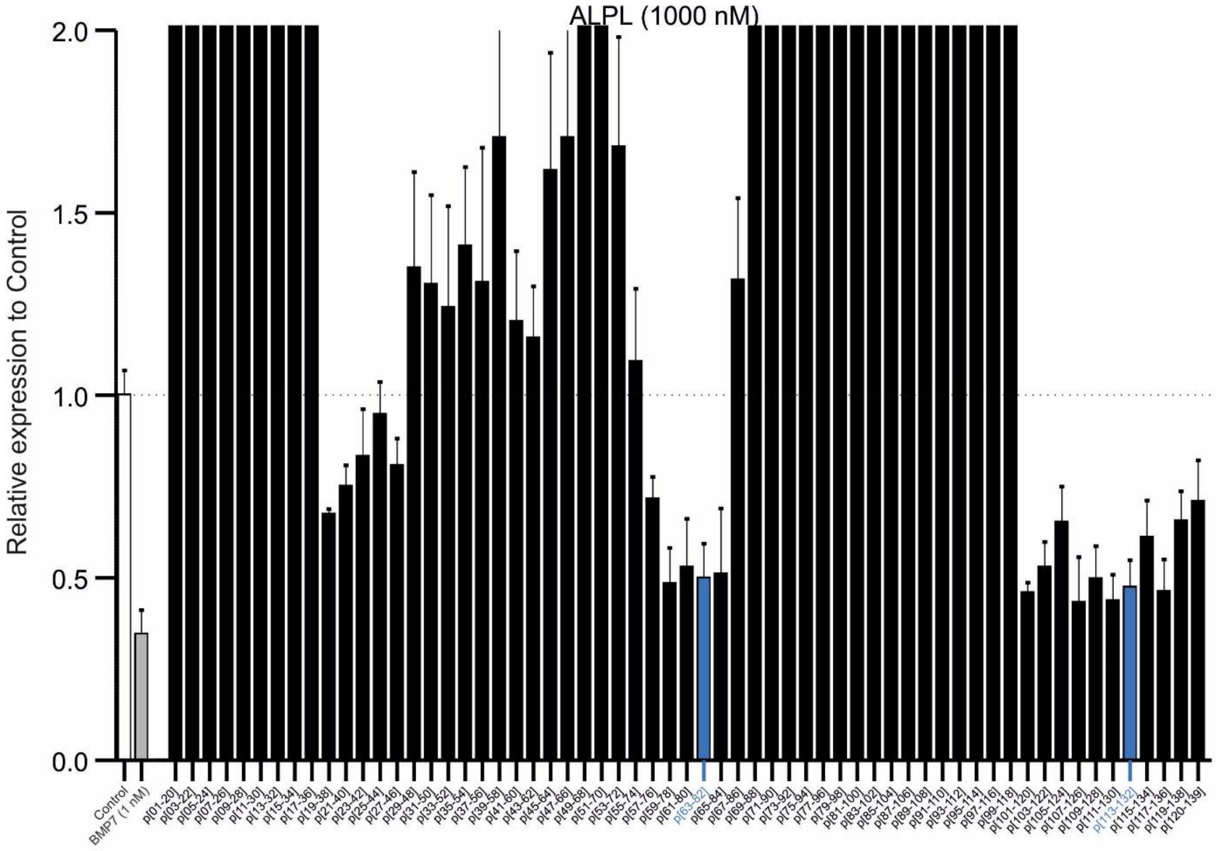
ALPL (1000 nM)
Relative expression to Control

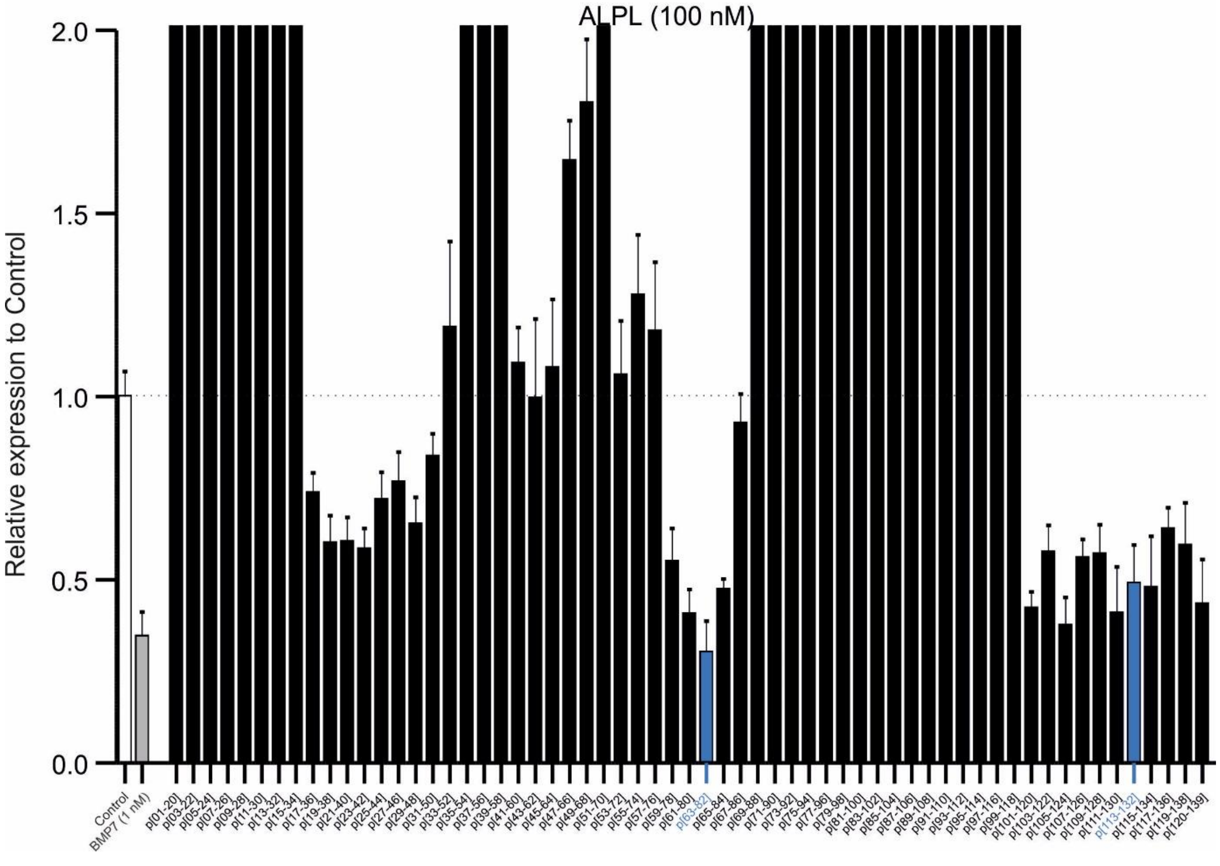
ALPL (100 nM)
Relative expression to Control

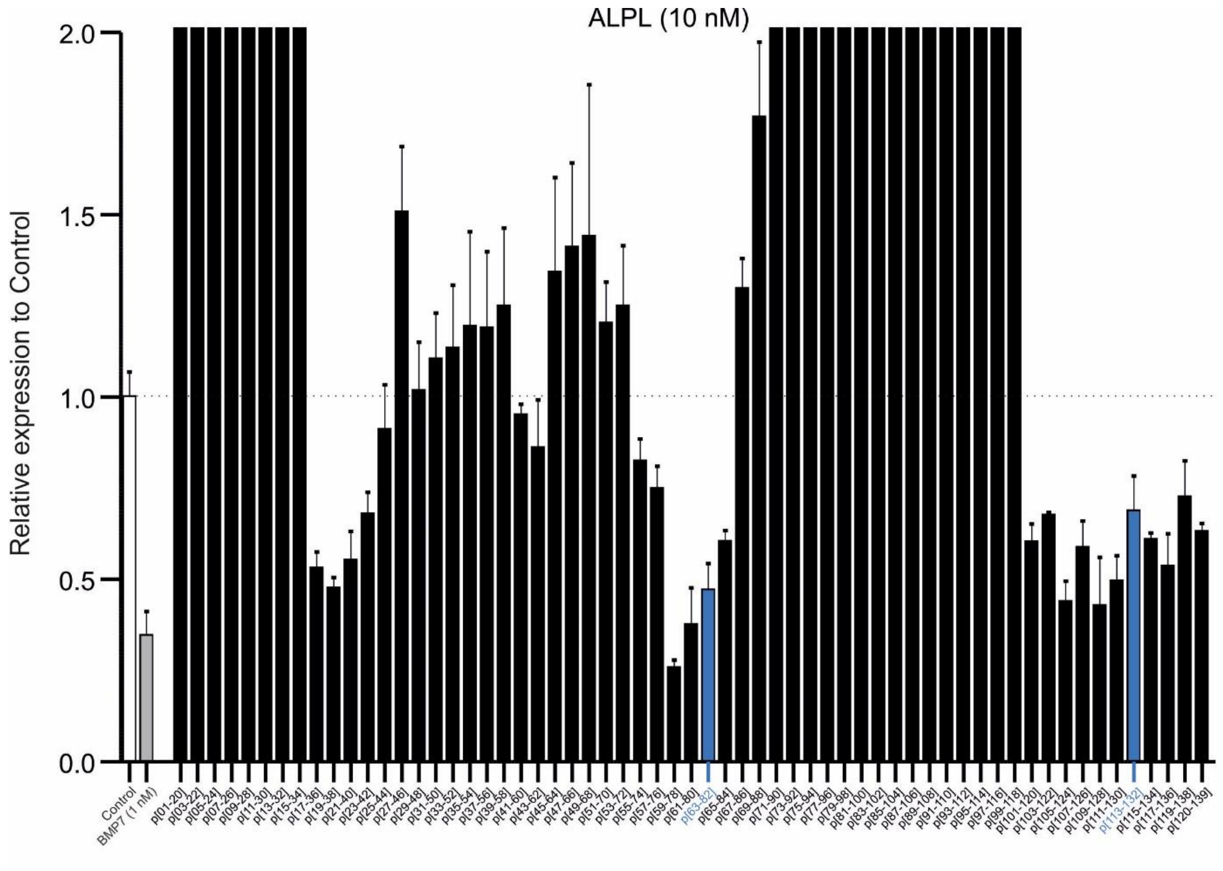
ALPL (10 nM)
Relative expression to Control

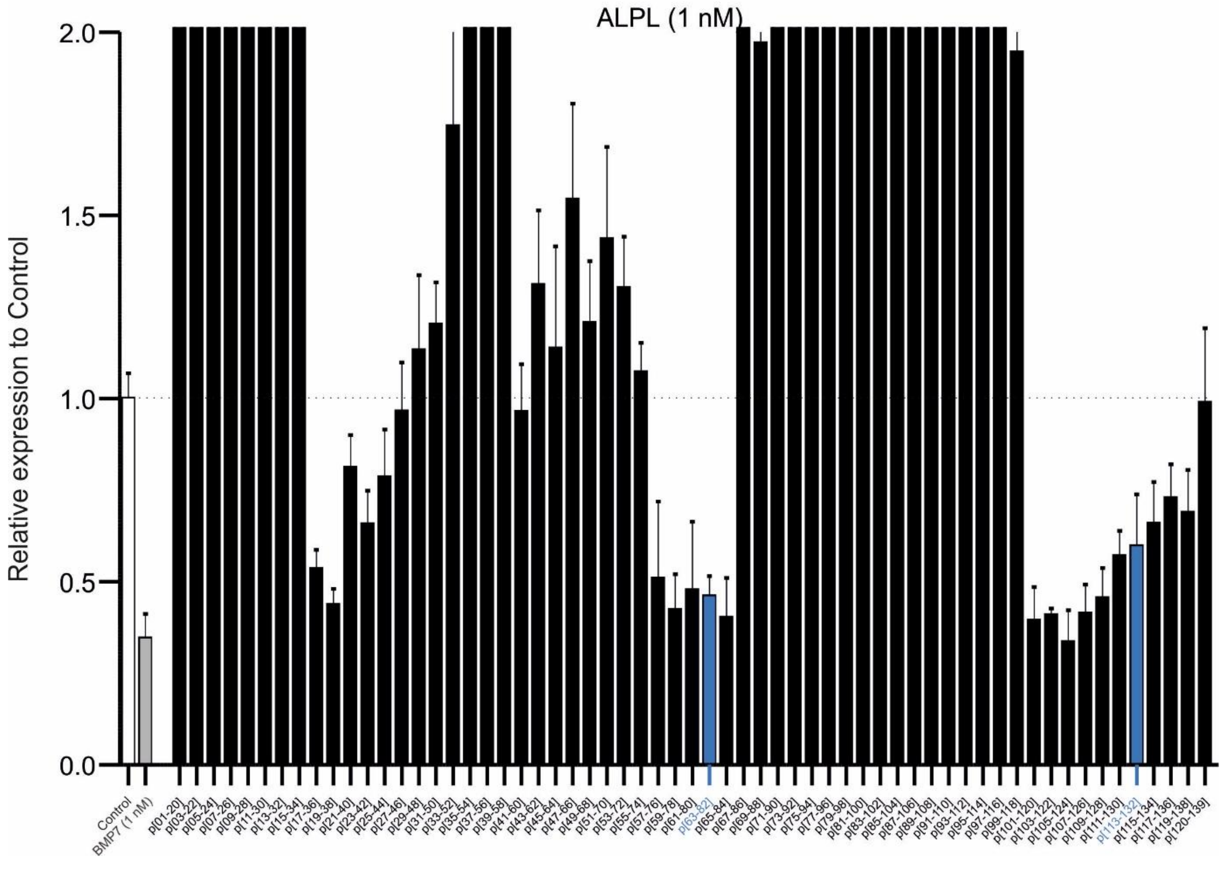
ALPL (1 nM)
Relative expression to Control

J

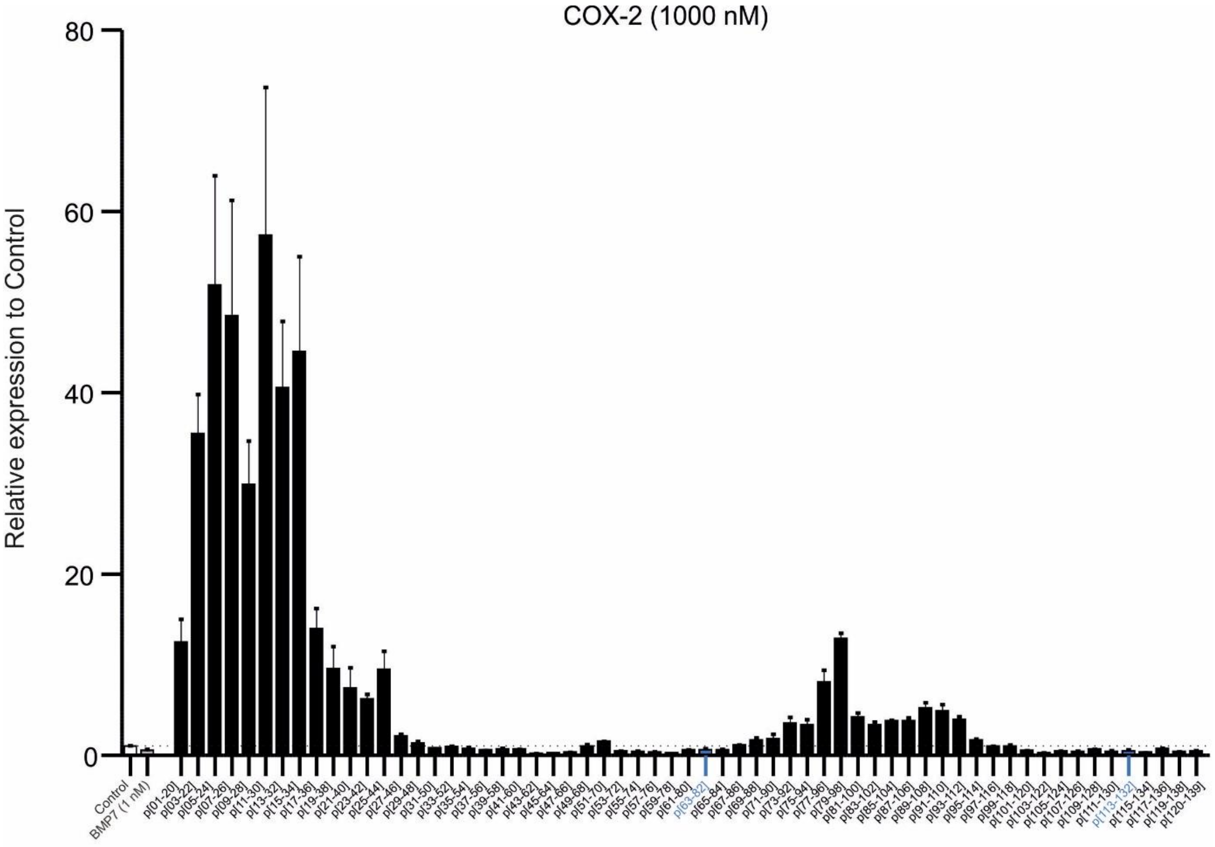
COX-2 (1000 nM)
Relative expression to Control
80
60
40
20
0

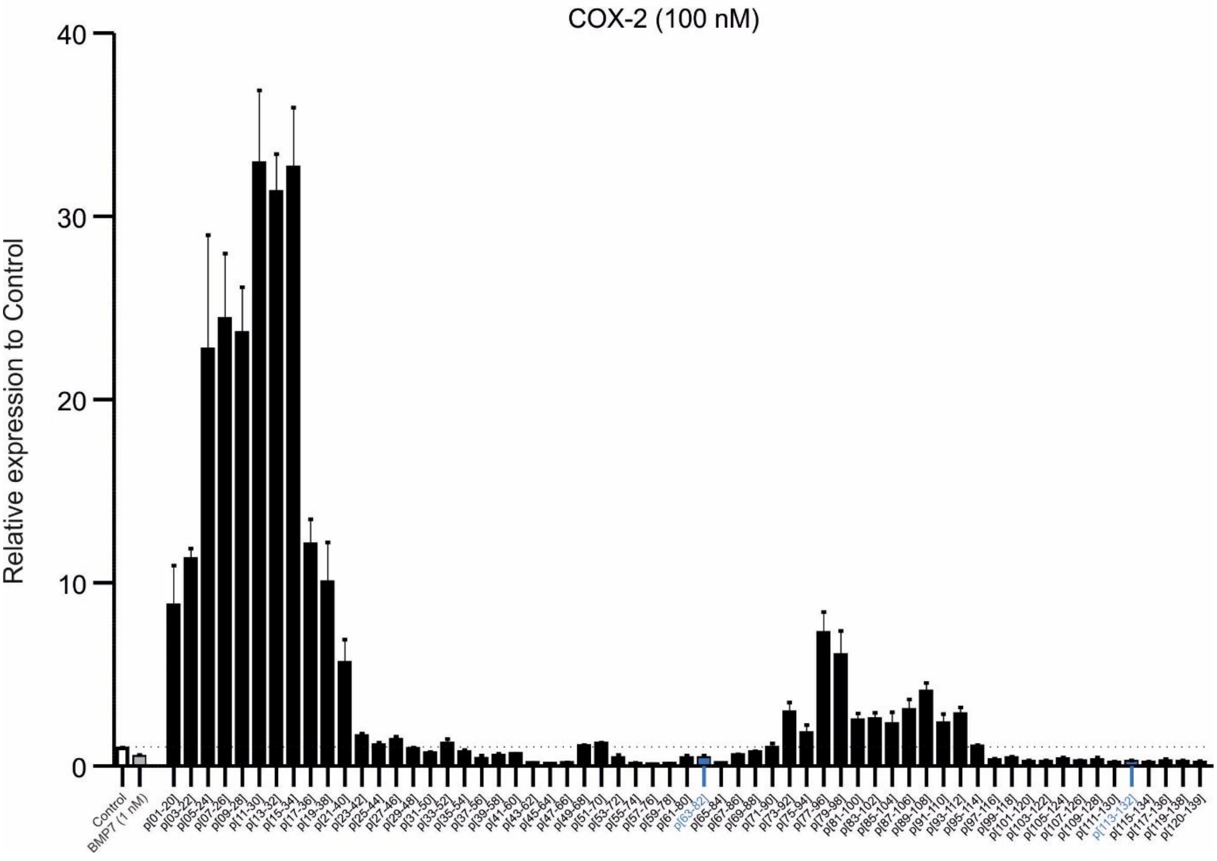
COX-2 (100 nM)
Relative expression to Control
40
30
20
10
0

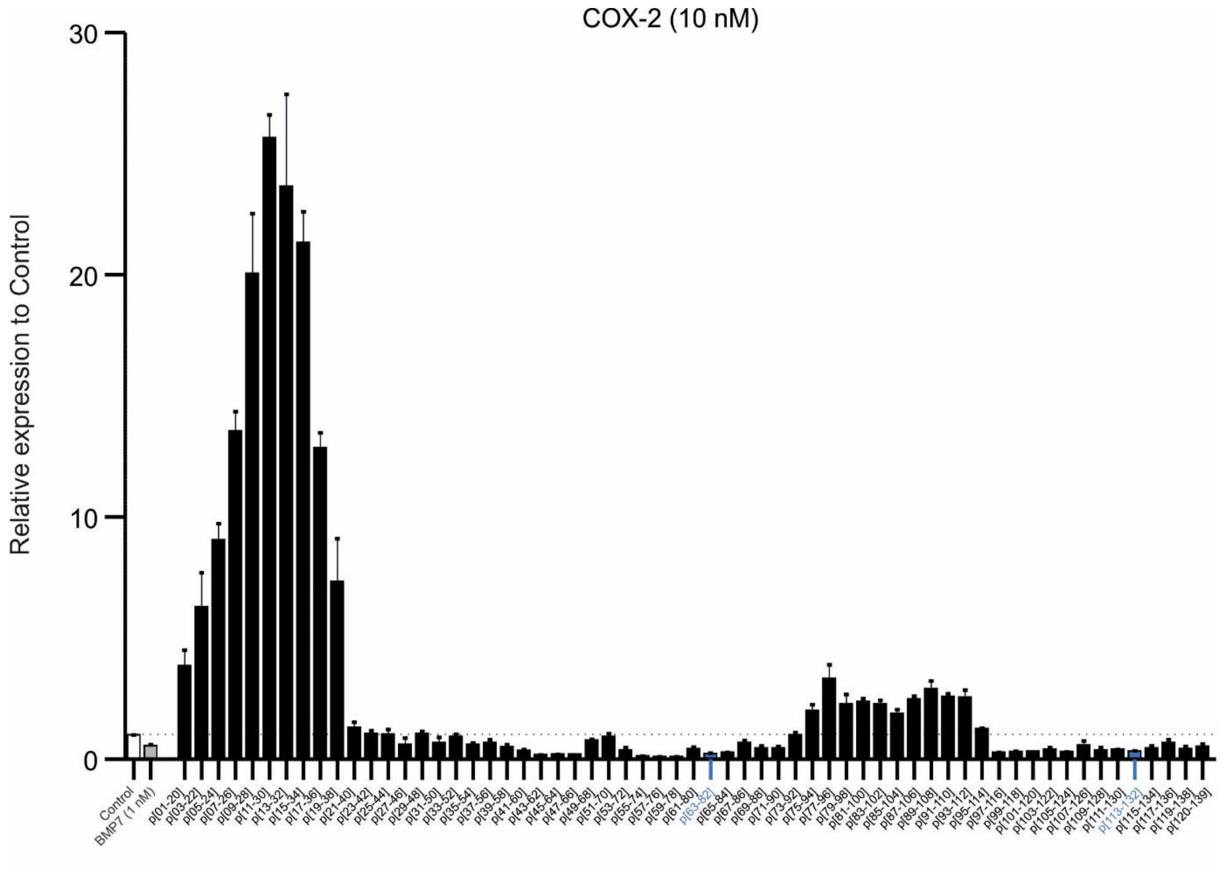
COX-2 (10 nM)
Relative expression to Control
30
20
10
0
Control
BMP7 (1 nM)

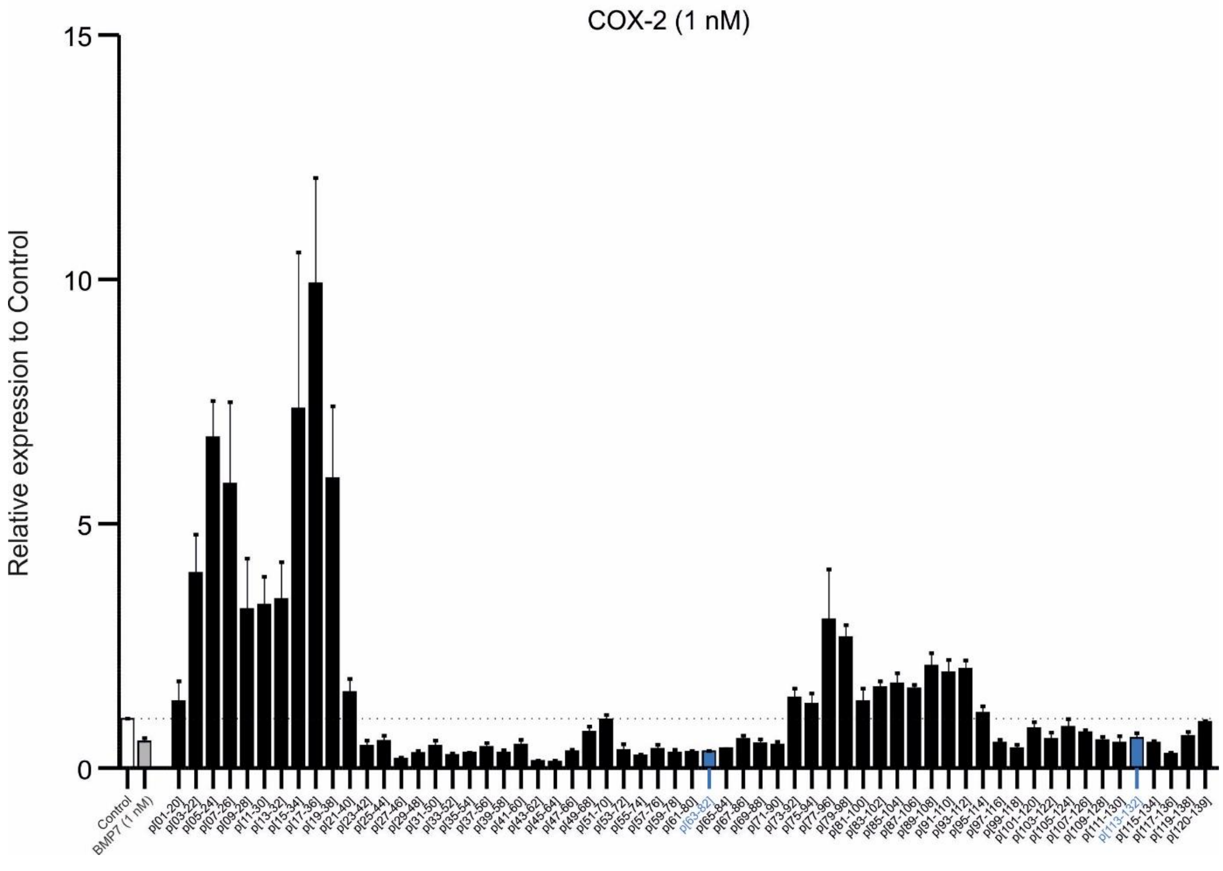
COX-2 (1 nM)
Relative expression to Control
15
10
5
0
Control
BMP7 (1 nM)

K

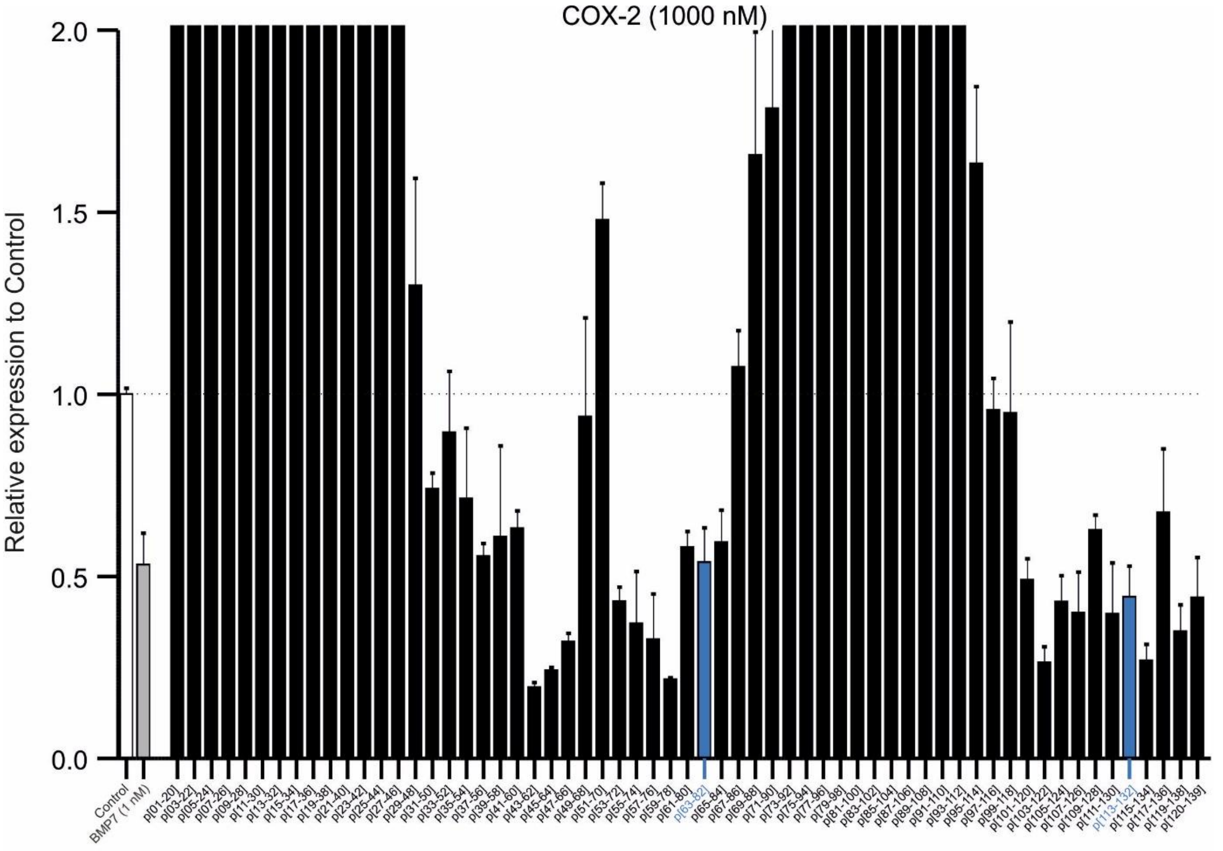
COX-2 (1000 nM)
Relative expression to Control
2.0
1.5
1.0
0.5
0.0
Control
BMP7 (1 nM)

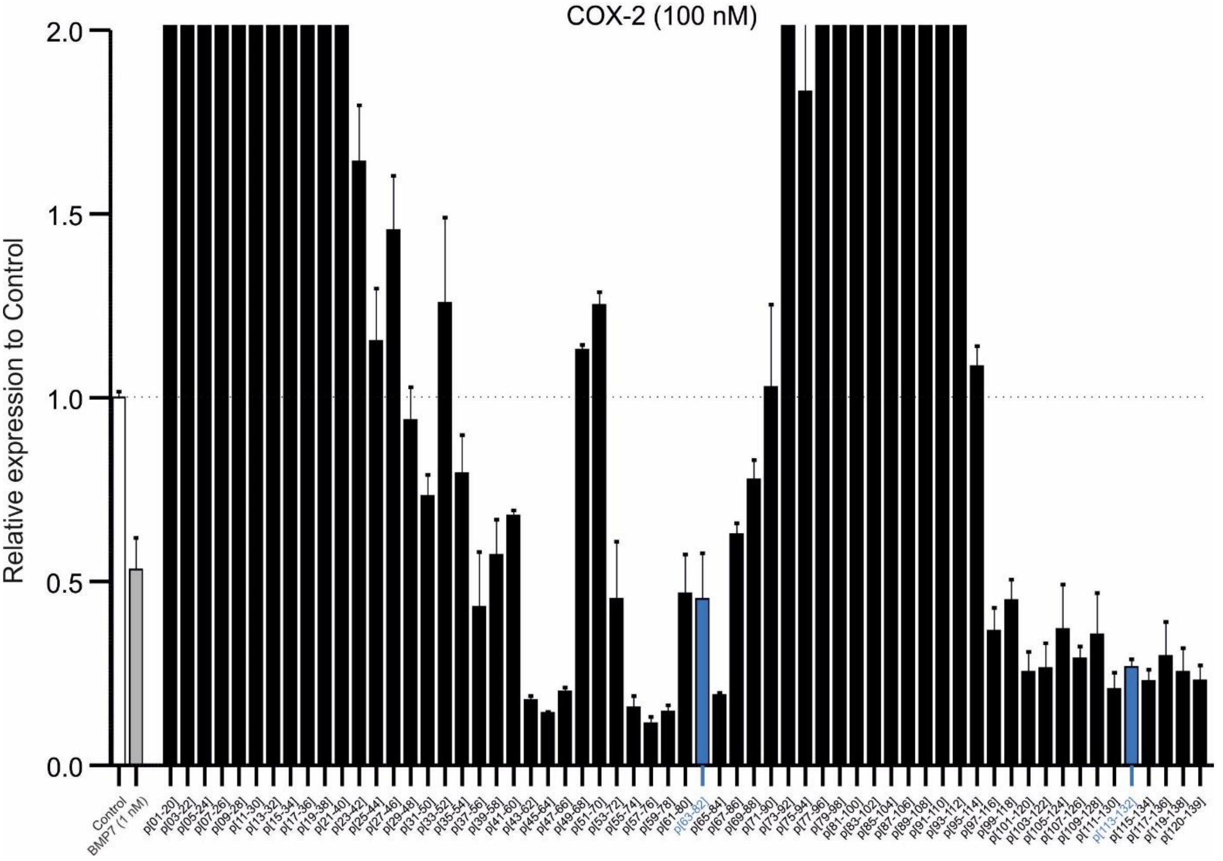
COX-2 (100 nM)
Relative expression to Control
2.0
1.5
1.0
0.5
0.0
Control
BMP7 (1 nM)

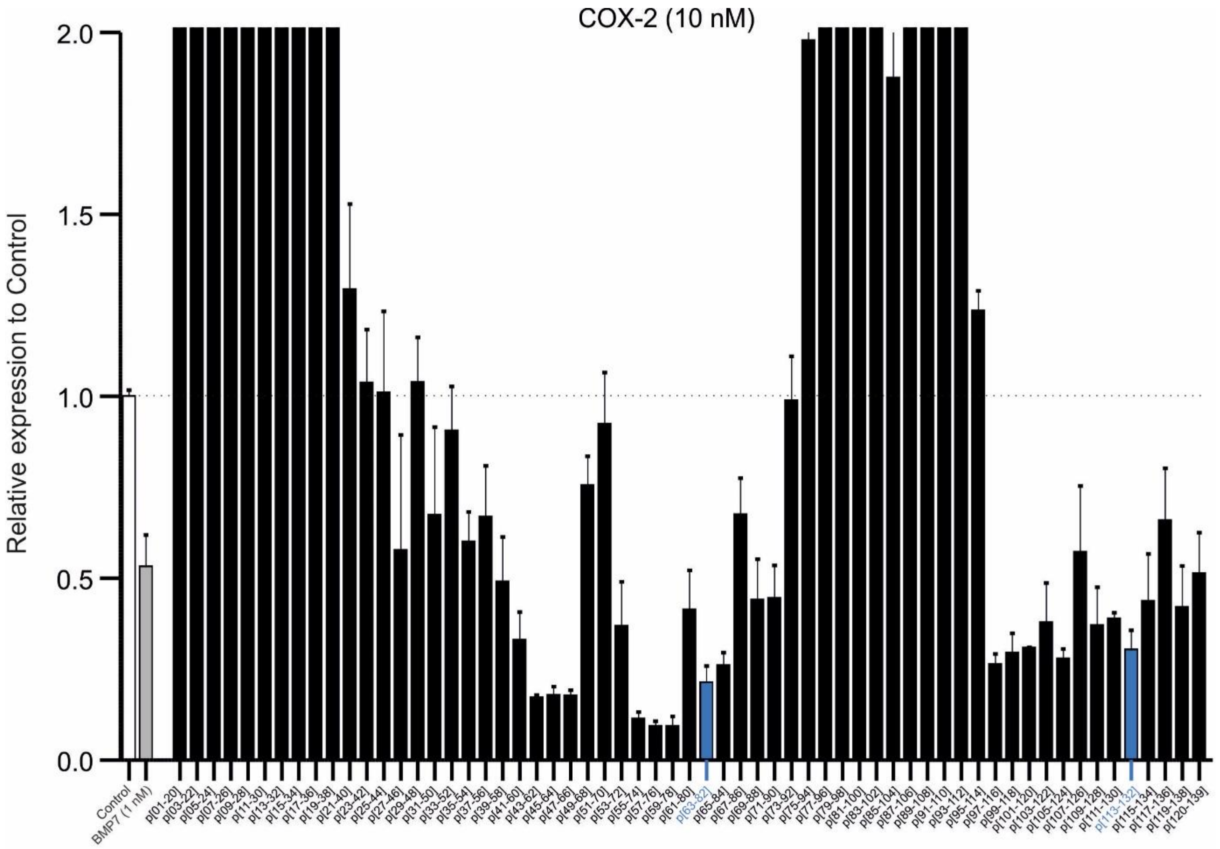
COX-2 (10 nM)
Relative expression to Control
0.0
0.5
1.0
1.5
2.0
Control
BMP7 (1 nM)
p[01-20] p[03-22] p[05-24] p[07-26] p[09-28] p[11-30] p[13-32] p[15-34] p[17-36] p[19-38] p[21-40] p[23-42] p[25-44] p[27-46] p[29-48] p[31-50] p[33-52] p[35-54] p[37-56] p[39-58] p[41-60] p[43-62] p[45-64] p[47-66] p[49-68] p[51-70] p[53-72] p[55-74] p[57-76] p[59-78] p[61-80] p[63-82] p[65-84] p[67-86] p[69-88] p[71-90] p[73-92] p[75-94] p[77-96] p[79-98] p[81-100] p[83-102] p[85-104] p[87-106] p[89-108] p[91-110] p[93-112] p[95-114] p[97-116] p[99-118] p[101-120] p[103-122] p[105-124] p[107-126] p[109-128] p[111-130] p[113-132] p[115-134] p[117-136] p[119-138] p[120-139]

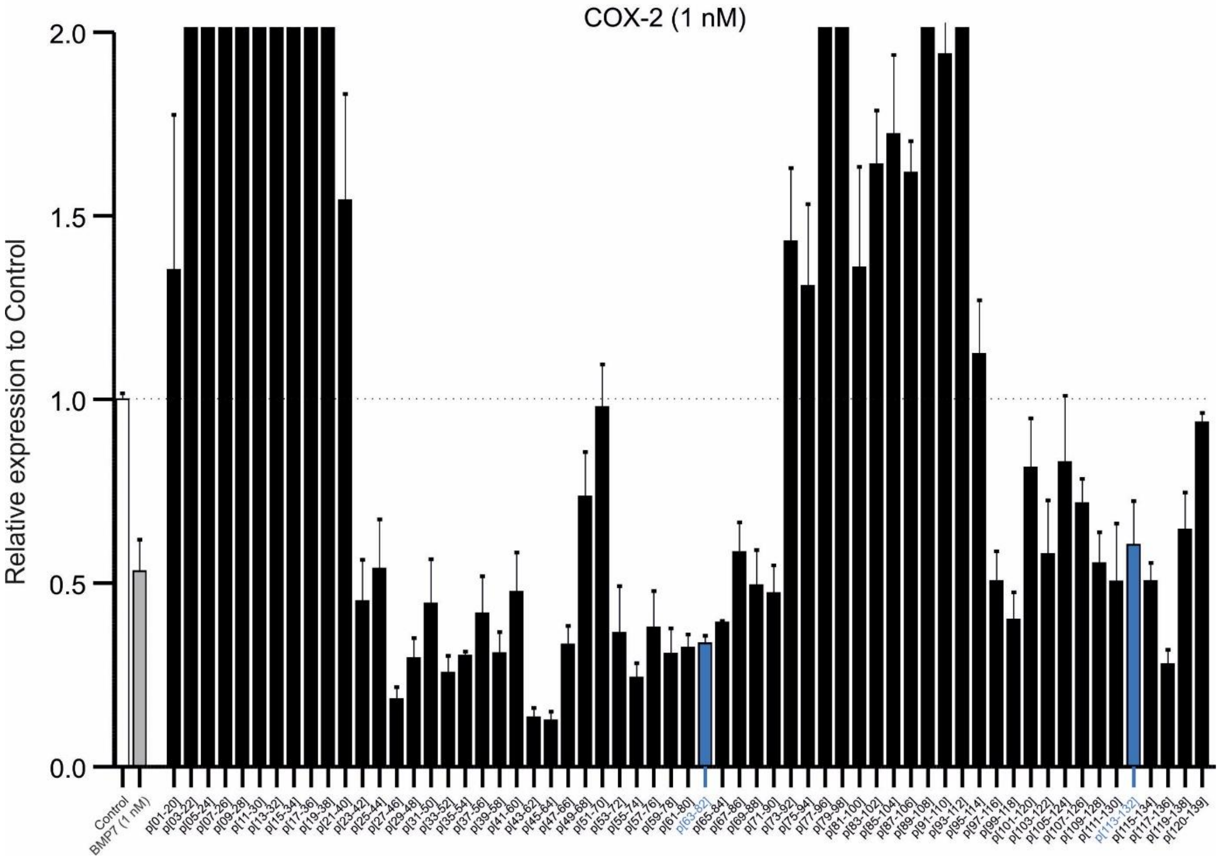
COX-2 (1 nM)
Relative expression to Control
0.0
0.5
1.0
1.5
2.0
Control
BMP7 (1 nM)
p[01-20] p[03-22] p[05-24] p[07-26] p[09-28] p[11-30] p[13-32] p[15-34] p[17-36] p[19-38] p[21-40] p[23-42] p[25-44] p[27-46] p[29-48] p[31-50] p[33-52] p[35-54] p[37-56] p[39-58] p[41-60] p[43-62] p[45-64] p[47-66] p[49-68] p[51-70] p[53-72] p[55-74] p[57-76] p[59-78] p[61-80] p[63-82] p[65-84] p[67-86] p[69-88] p[71-90] p[73-92] p[75-94] p[77-96] p[79-98] p[81-100] p[83-102] p[85-104] p[87-106] p[89-108] p[91-110] p[93-112] p[95-114] p[97-116] p[99-118] p[101-120] p[103-122] p[105-124] p[107-126] p[109-128] p[111-130] p[113-132] p[115-134] p[117-136] p[119-138] p[120-139]

L

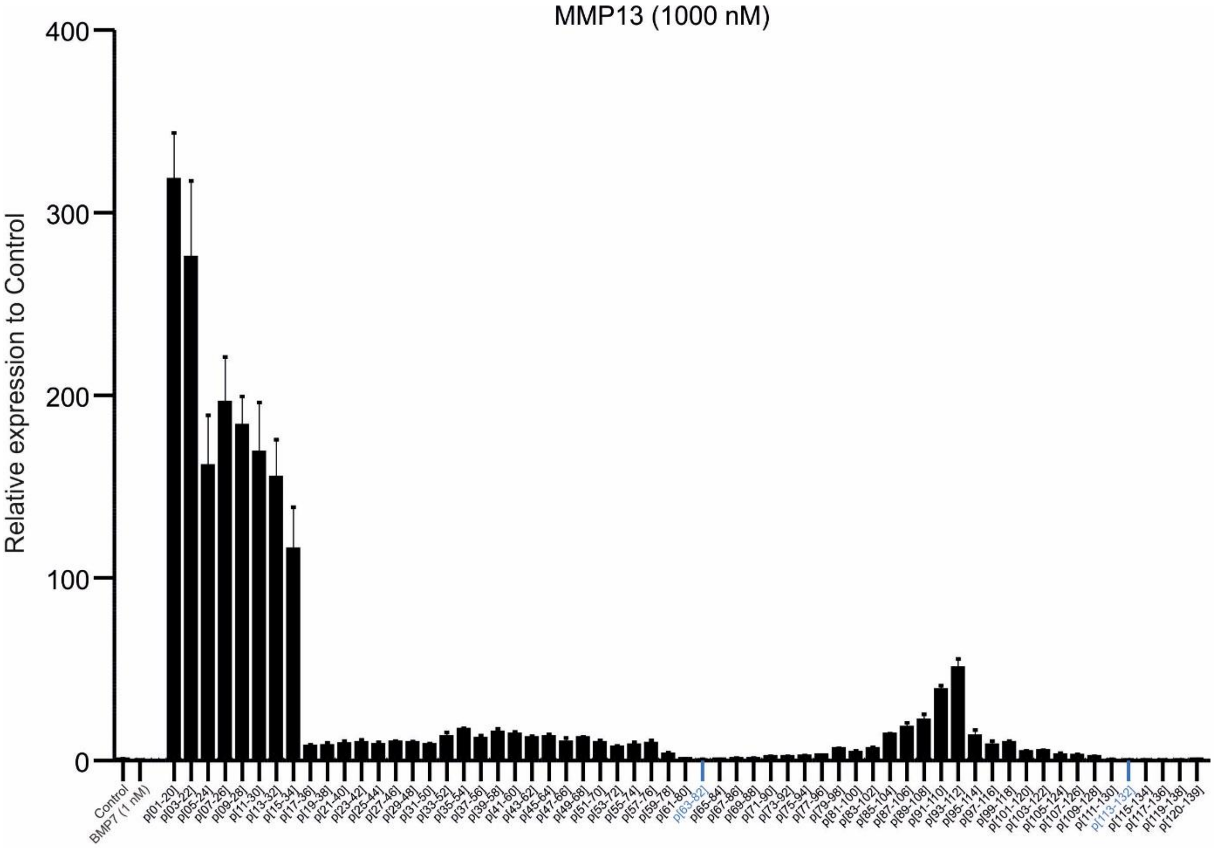
MMP13 (1000 nM)
Relative expression to Control
400
300
200
100
0
Control
BMP7 (1 nM)

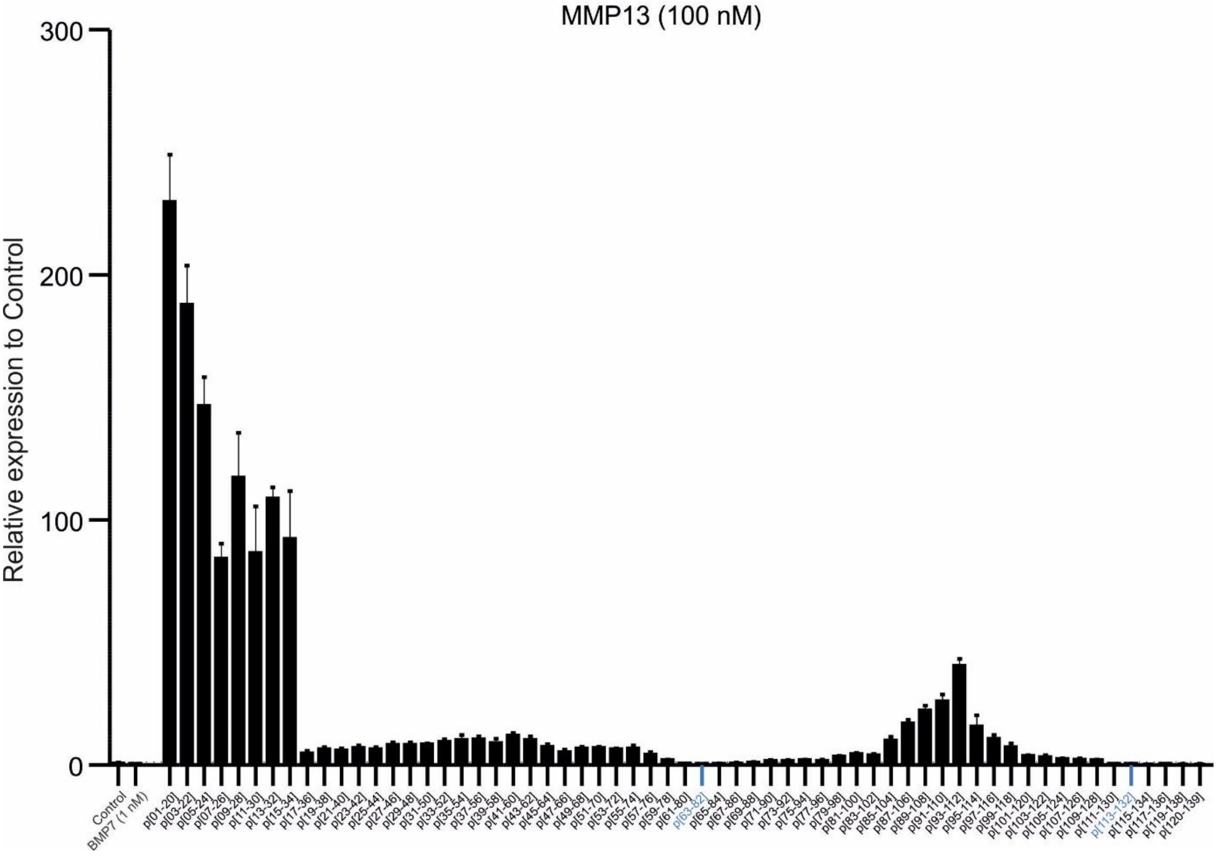
MMP13 (100 nM)
Relative expression to Control
300
200
100
0
Control
BMP7 (1 nM)

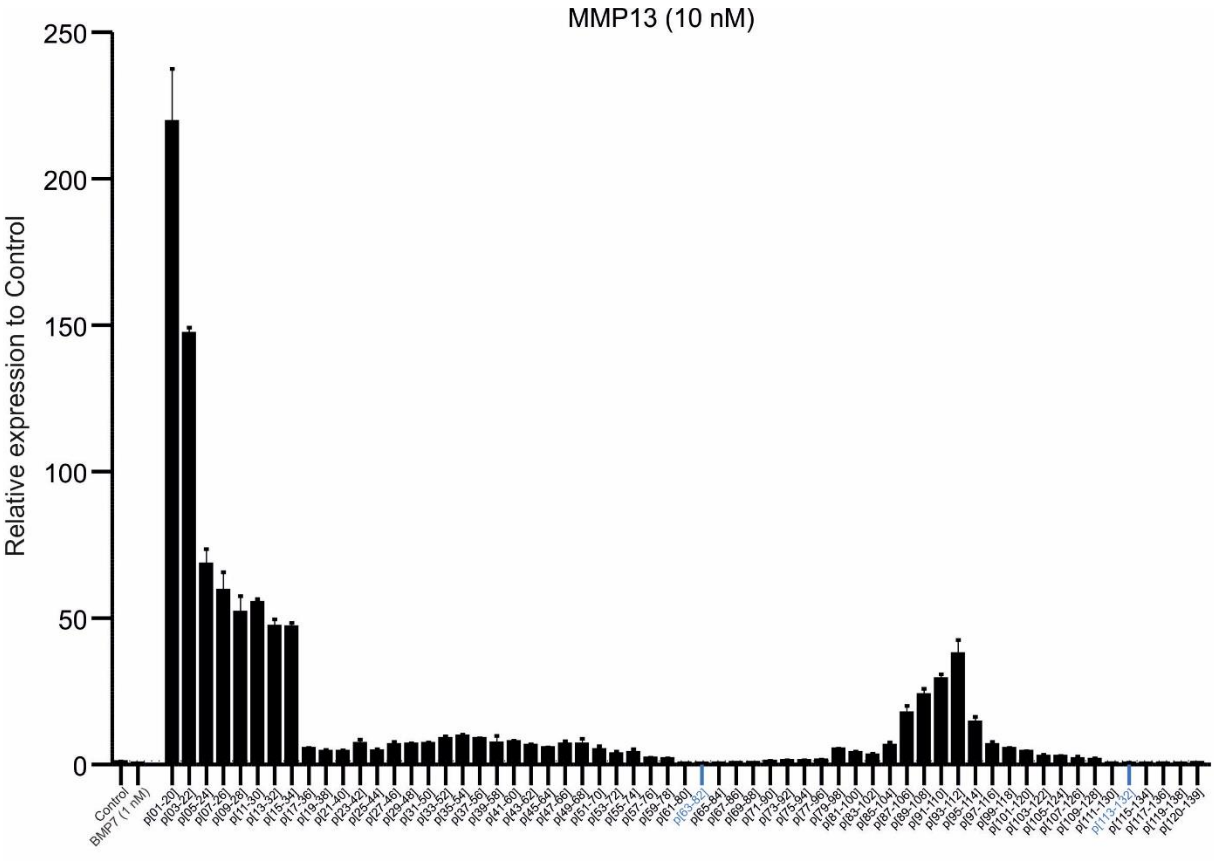
MMP13 (10 nM)
Relative expression to Control
250
200
150
100
50
0

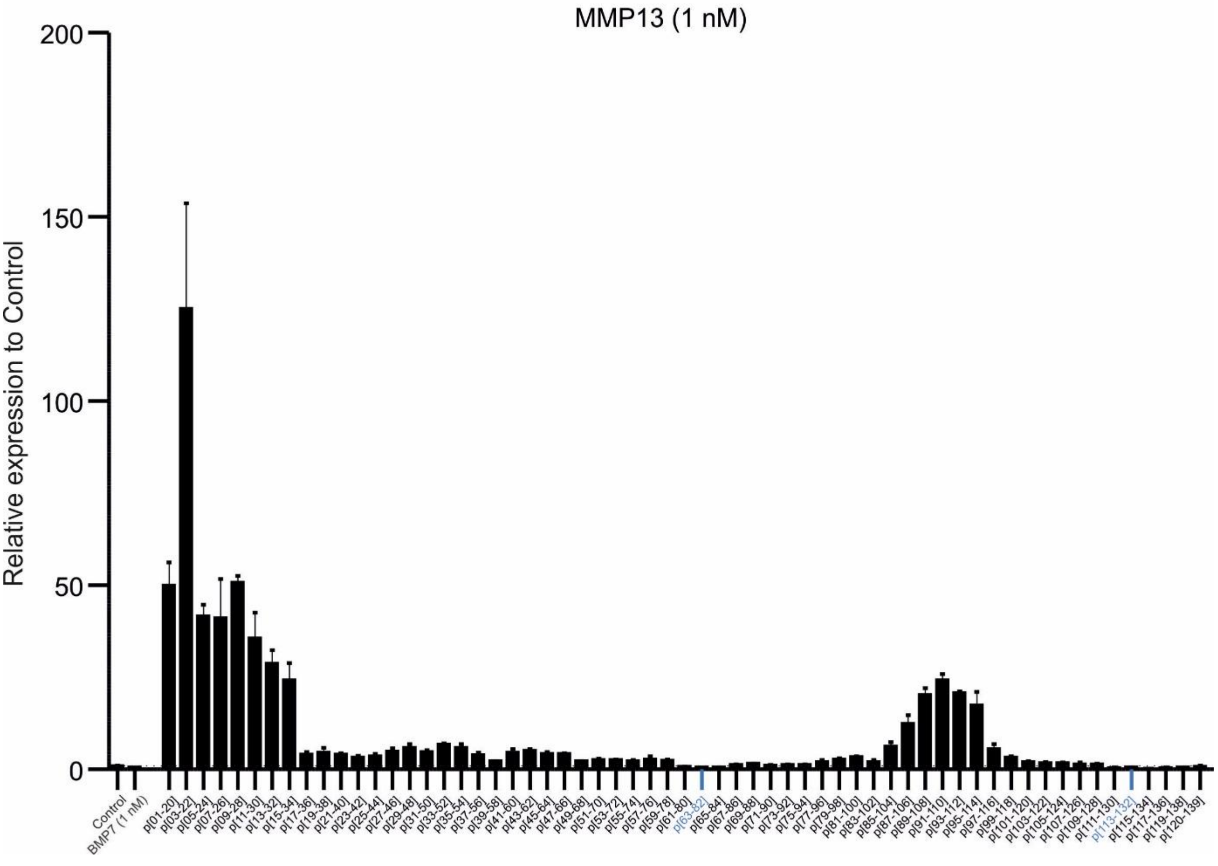
MMP13 (1 nM)
Relative expression to Control
200
150
100
50
0

M

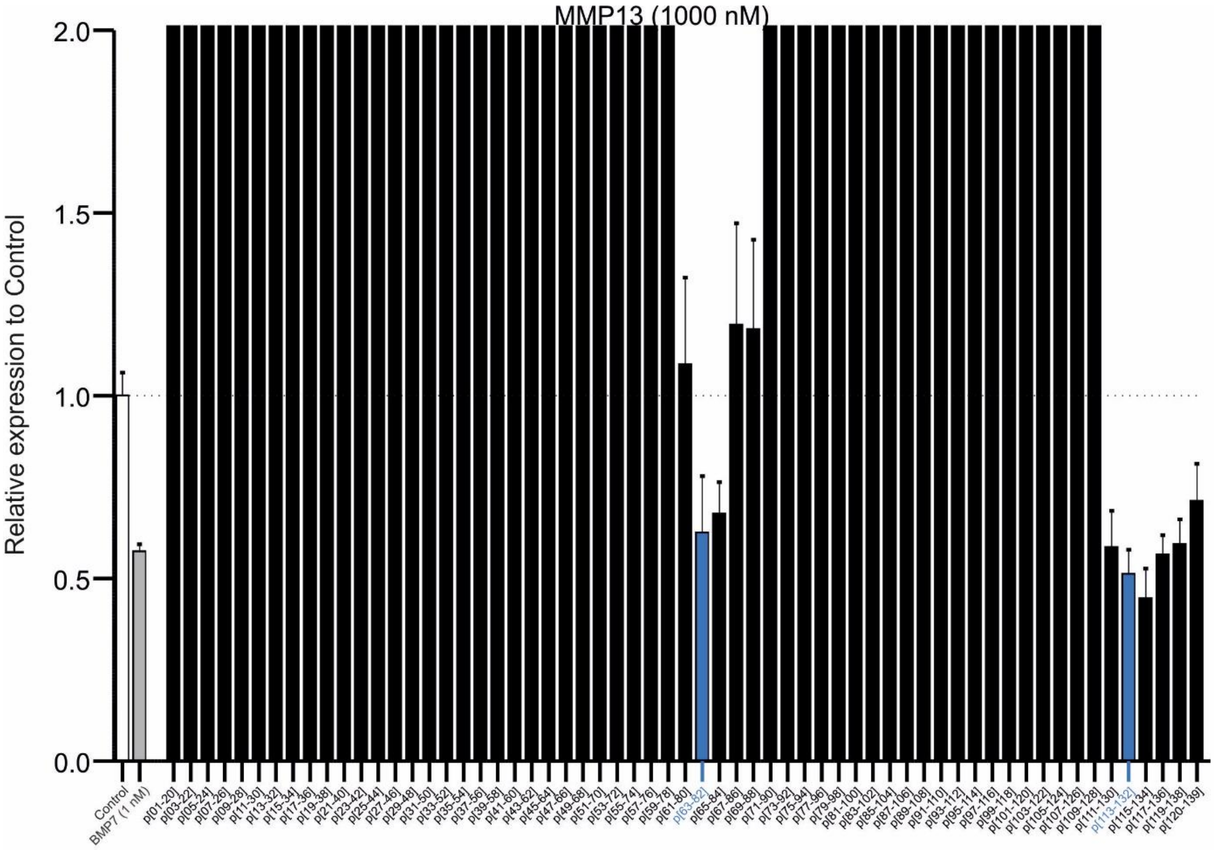
MMP13 (1000 nM)
Relative expression to Control
2.0
1.5
1.0
0.5
0.0
Control
BMP7 (1 nM)

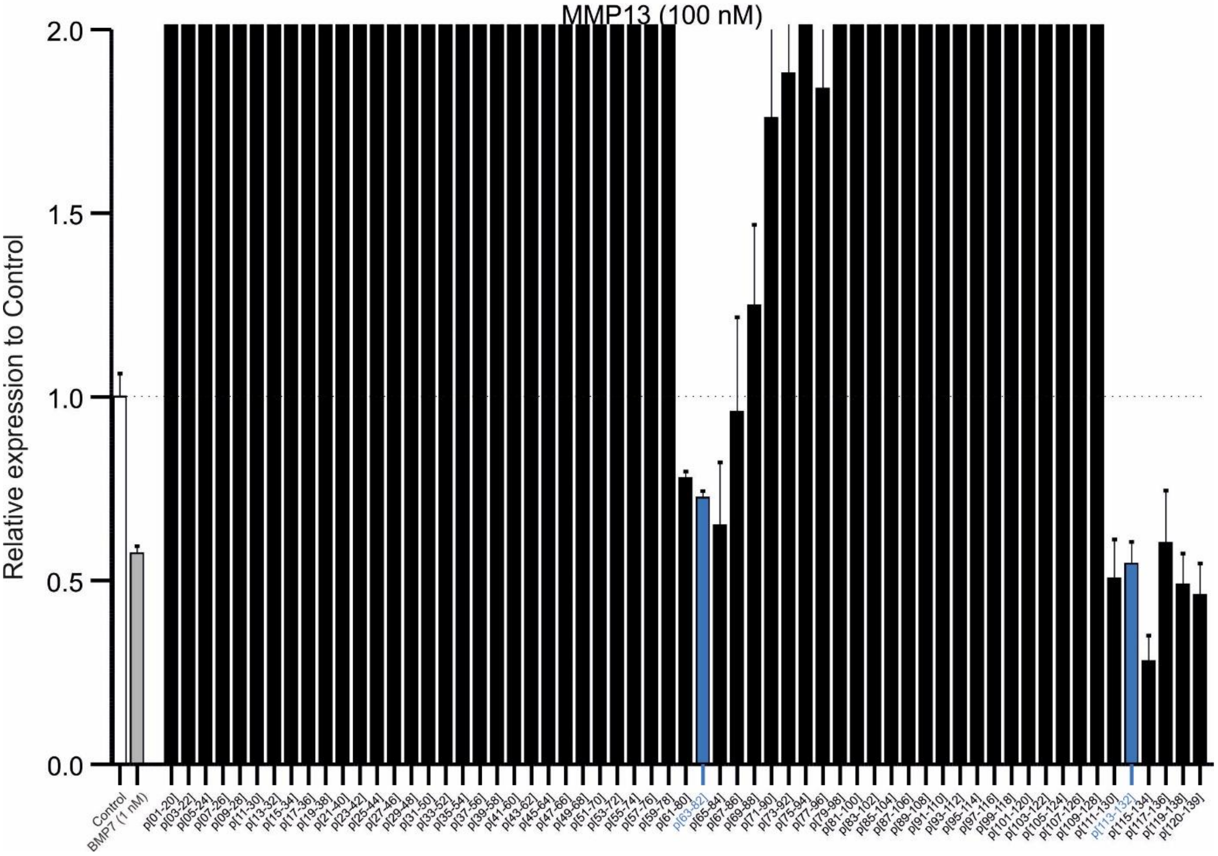
MMP13 (100 nM)
Relative expression to Control
2.0
1.5
1.0
0.5
0.0
Control
BMP7 (1 nM)

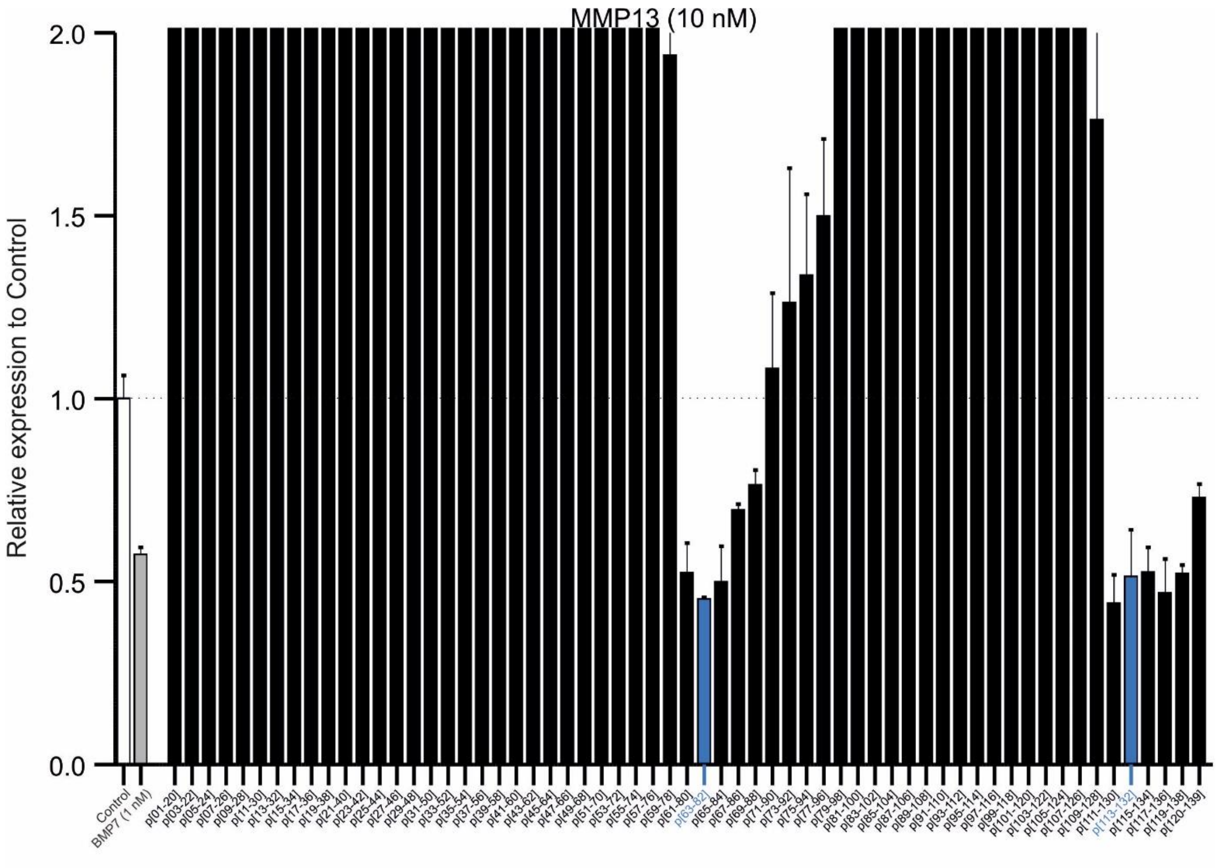
MMP13 (10 nM)
Relative expression to Control
2.0
1.5
1.0
0.5
0.0
Control
BMP7 (1 nM)
p[01-20] p[03-22] p[05-24] p[07-26] p[09-28] p[11-30] p[13-32] p[15-34] p[17-36] p[19-38] p[21-40] p[23-42] p[25-44] p[27-46] p[29-48] p[31-50] p[33-52] p[35-54] p[37-56] p[39-58] p[41-60] p[43-62] p[45-64] p[47-66] p[49-68] p[51-70] p[53-72] p[55-74] p[57-76] p[59-78] p[61-80] p[63-82] p[65-84] p[67-86] p[69-88] p[71-90] p[73-92] p[75-94] p[77-96] p[79-98] p[81-100] p[83-102] p[85-104] p[87-106] p[89-108] p[91-110] p[93-112] p[95-114] p[97-116] p[99-118] p[101-120] p[103-122] p[105-124] p[107-126] p[109-128] p[111-130] p[113-132] p[115-134] p[117-136] p[119-138] p[120-139]

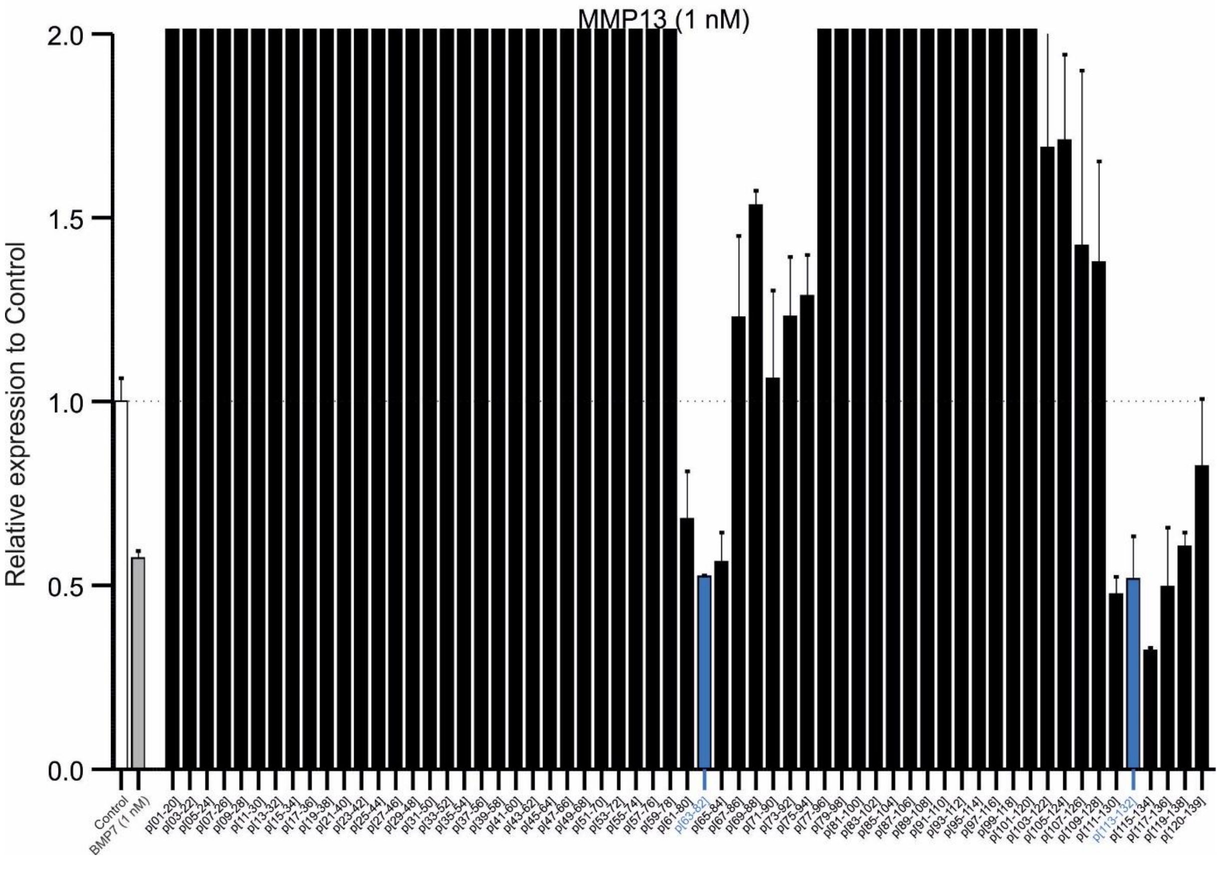
MMP13 (1 nM)
Relative expression to Control
2.0
1.5
1.0
0.5
0.0
Control
BMP7 (1 nM)
p[01-20] p[03-22] p[05-24] p[07-26] p[09-28] p[11-30] p[13-32] p[15-34] p[17-36] p[19-38] p[21-40] p[23-42] p[25-44] p[27-46] p[29-48] p[31-50] p[33-52] p[35-54] p[37-56] p[39-58] p[41-60] p[43-62] p[45-64] p[47-66] p[49-68] p[51-70] p[53-72] p[55-74] p[57-76] p[59-78] p[61-80] p[63-82] p[65-84] p[67-86] p[69-88] p[71-90] p[73-92] p[75-94] p[77-96] p[79-98] p[81-100] p[83-102] p[85-104] p[87-106] p[89-108] p[91-110] p[93-112] p[95-114] p[97-116] p[99-118] p[101-120] p[103-122] p[105-124] p[107-126] p[109-128] p[111-130] p[113-132] p[115-134] p[117-136] p[119-138] p[120-139]

N

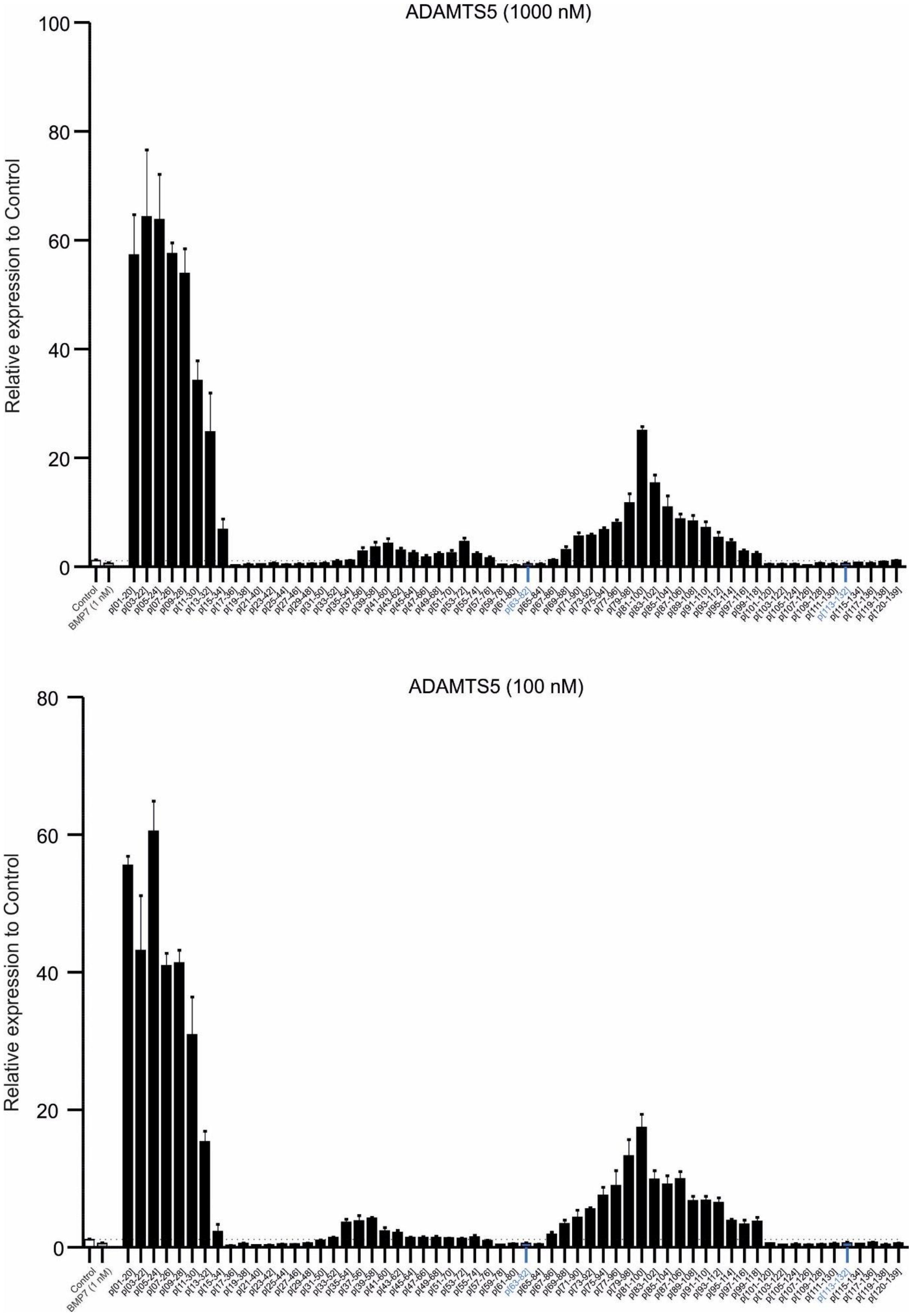
ADAMTS5 (1000 nM)
Relative expression to Control
100
80
60
40
20
0
Control
BMP7 (1 nM)
ADAMTS5 (100 nM)
Relative expression to Control
80
60
40
20
0
Control
BMP7 (1 nM)

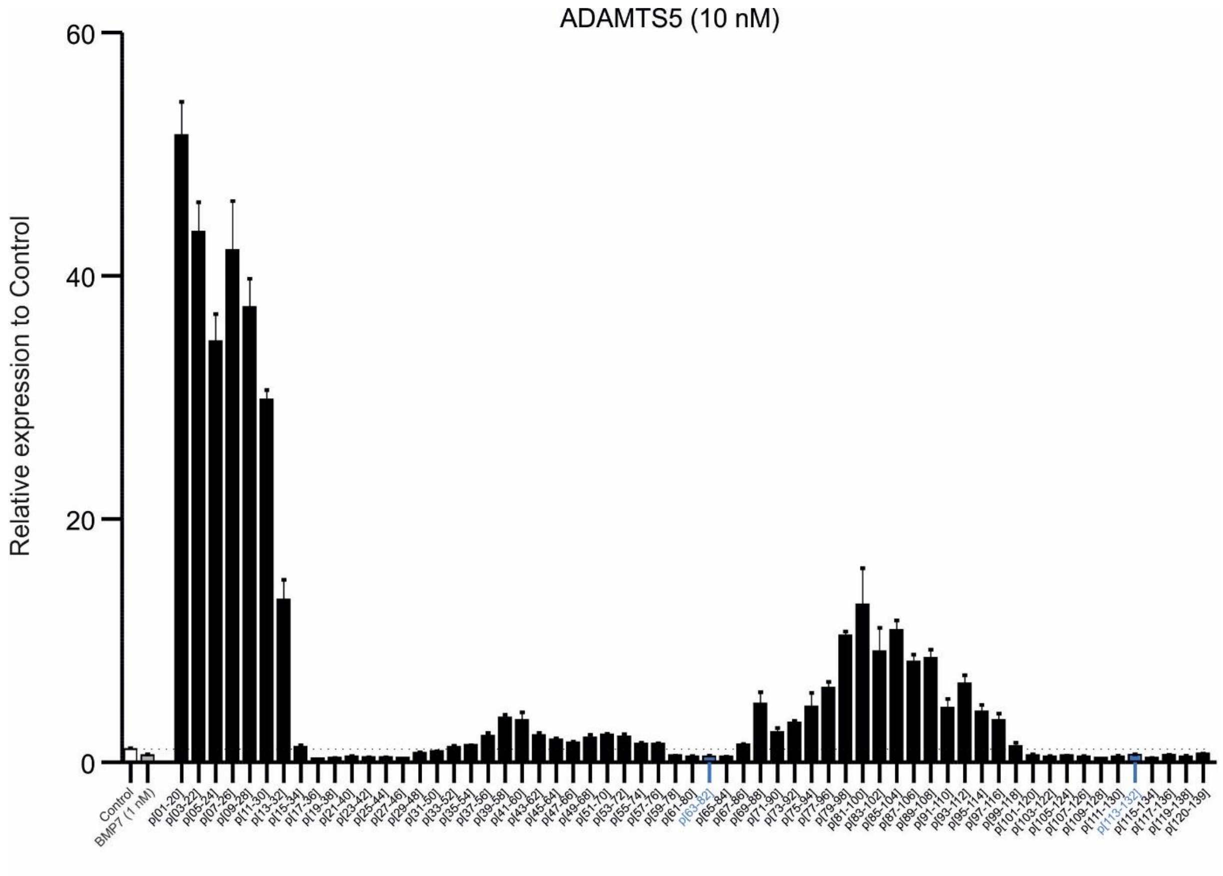
ADAMTS5 (10 nM)
Relative expression to Control
60
40
20
0
Control
BMP7 (1 nM)
p[01-20]
p[03-22]
p[05-24]
p[07-26]
p[09-28]
p[11-30]
p[13-32]
p[15-34]
p[17-36]
p[19-38]
p[21-40]
p[23-42]
p[25-44]
p[27-46]
p[29-48]
p[31-50]
p[33-52]
p[35-54]
p[37-56]
p[39-58]
p[41-60]
p[43-62]
p[45-64]
p[47-66]
p[49-68]
p[51-70]
p[53-72]
p[55-74]
p[57-76]
p[59-78]
p[61-80]
p[63-82]
p[65-84]
p[67-86]
p[69-88]
p[71-90]
p[73-92]
p[75-94]
p[77-96]
p[79-98]
p[81-100]
p[83-102]
p[85-104]
p[87-106]
p[89-108]
p[91-110]
p[93-112]
p[95-114]
p[97-116]
p[99-118]
p[101-120]
p[103-122]
p[105-124]
p[107-126]
p[109-128]
p[111-130]
p[113-132]
p[115-134]
p[117-136]
p[119-138]
p[120-139]

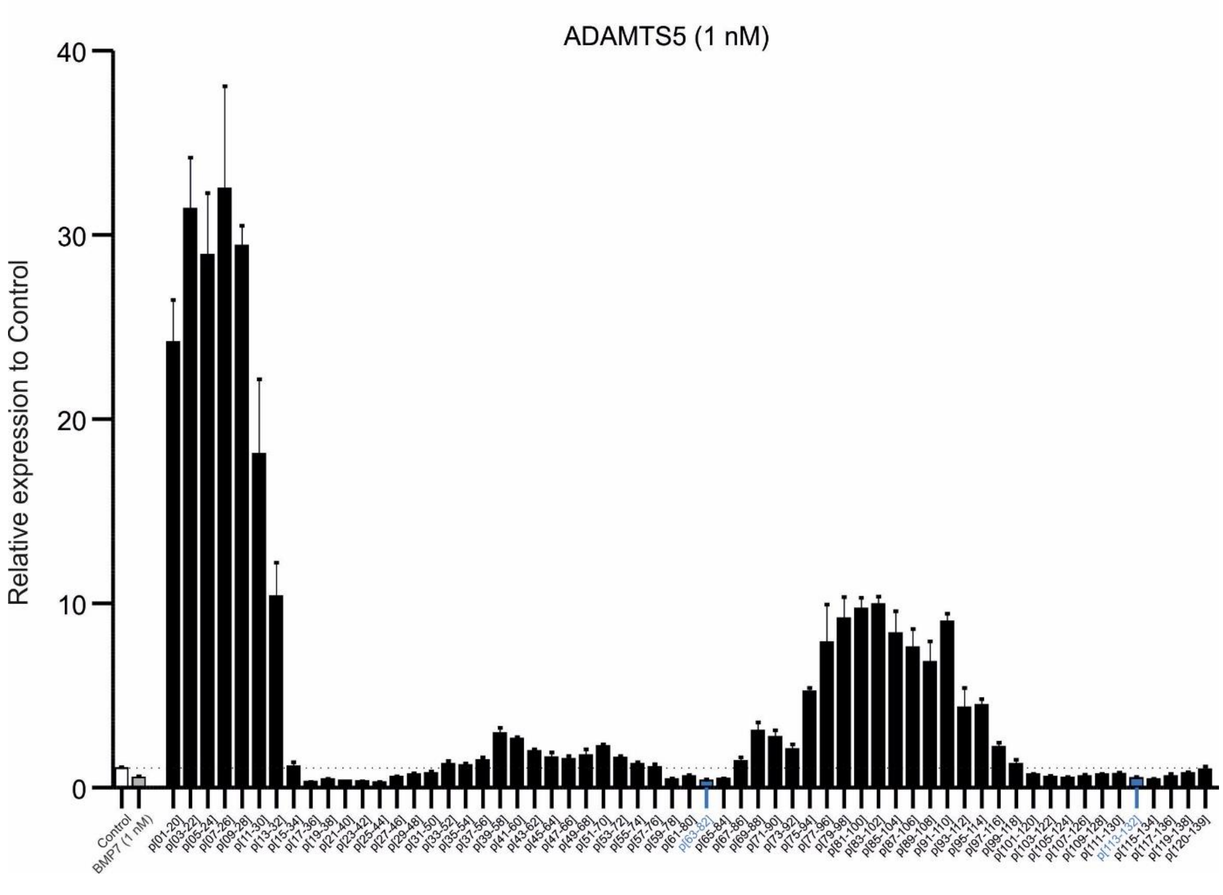
ADAMTS5 (1 nM)
Relative expression to Control
40
30
20
10
0
Control
BMP7 (1 nM)
p[01-20]
p[03-22]
p[05-24]
p[07-26]
p[09-28]
p[11-30]
p[13-32]
p[15-34]
p[17-36]
p[19-38]
p[21-40]
p[23-42]
p[25-44]
p[27-46]
p[29-48]
p[31-50]
p[33-52]
p[35-54]
p[37-56]
p[39-58]
p[41-60]
p[43-62]
p[45-64]
p[47-66]
p[49-68]
p[51-70]
p[53-72]
p[55-74]
p[57-76]
p[59-78]
p[61-80]
p[63-82]
p[65-84]
p[67-86]
p[69-88]
p[71-90]
p[73-92]
p[75-94]
p[77-96]
p[79-98]
p[81-100]
p[83-102]
p[85-104]
p[87-106]
p[89-108]
p[91-110]
p[93-112]
p[95-114]
p[97-116]
p[99-118]
p[101-120]
p[103-122]
p[105-124]
p[107-126]
p[109-128]
p[111-130]
p[113-132]
p[115-134]
p[117-136]
p[119-138]
p[120-139]

O

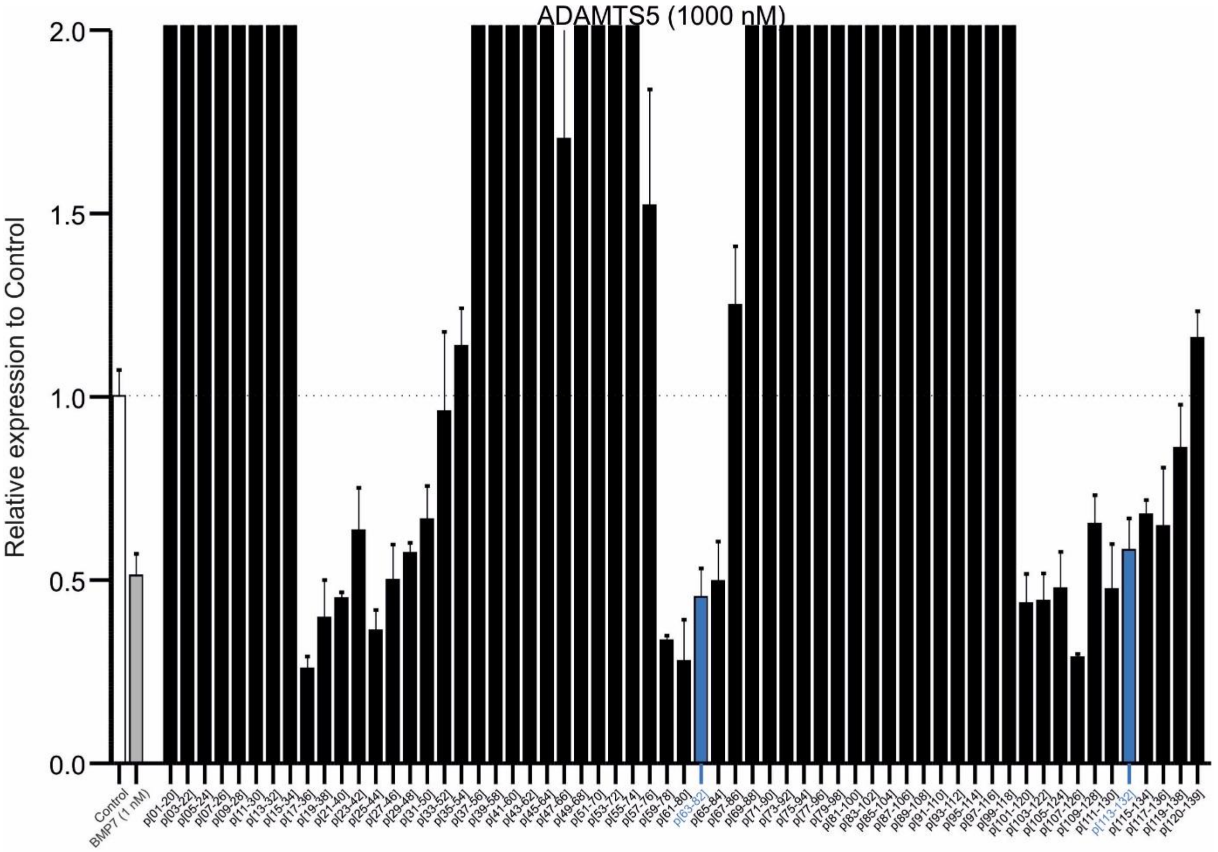
ADAMTS5 (1000 nM)
Relative expression to Control
2.0
1.5
1.0
0.5
0.0
Control
BMP7 (1 nM)

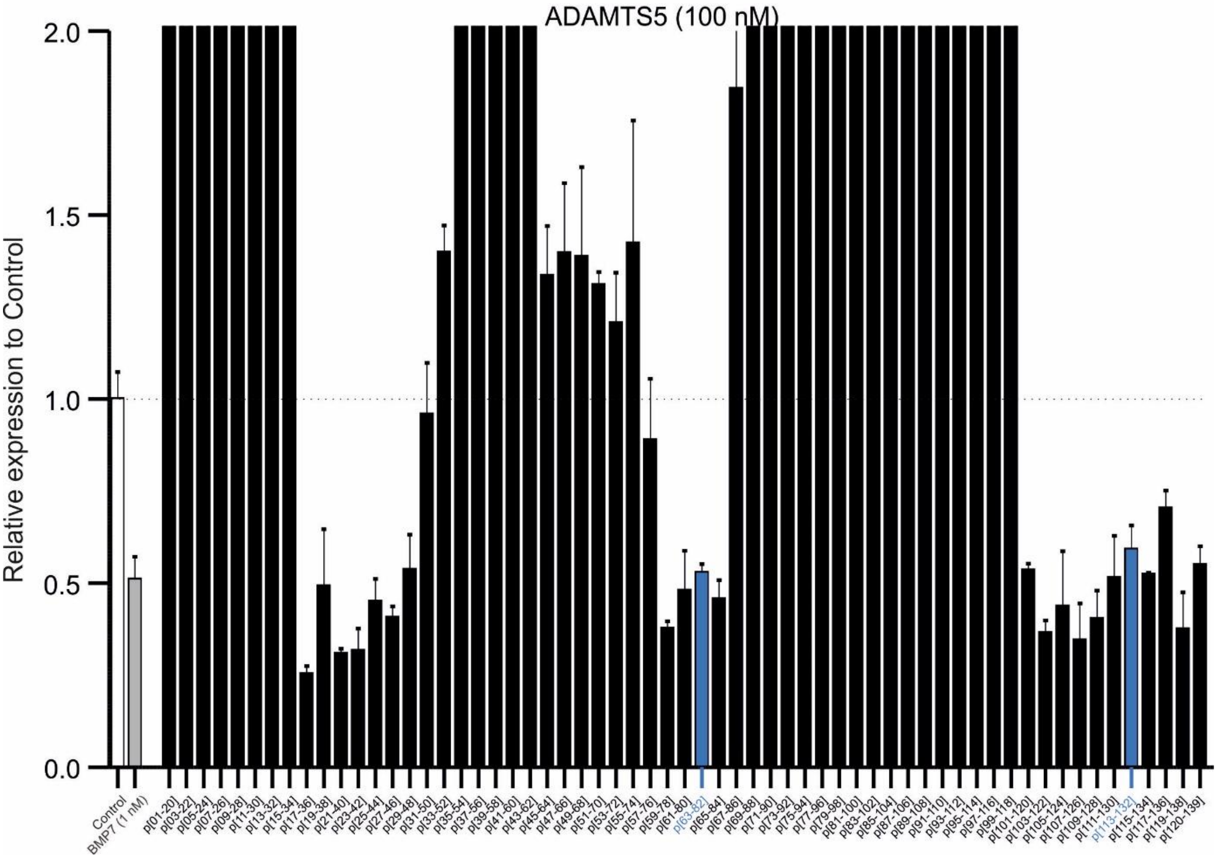
ADAMTS5 (100 nM)
Relative expression to Control
2.0
1.5
1.0
0.5
0.0
Control
BMP7 (1 nM)

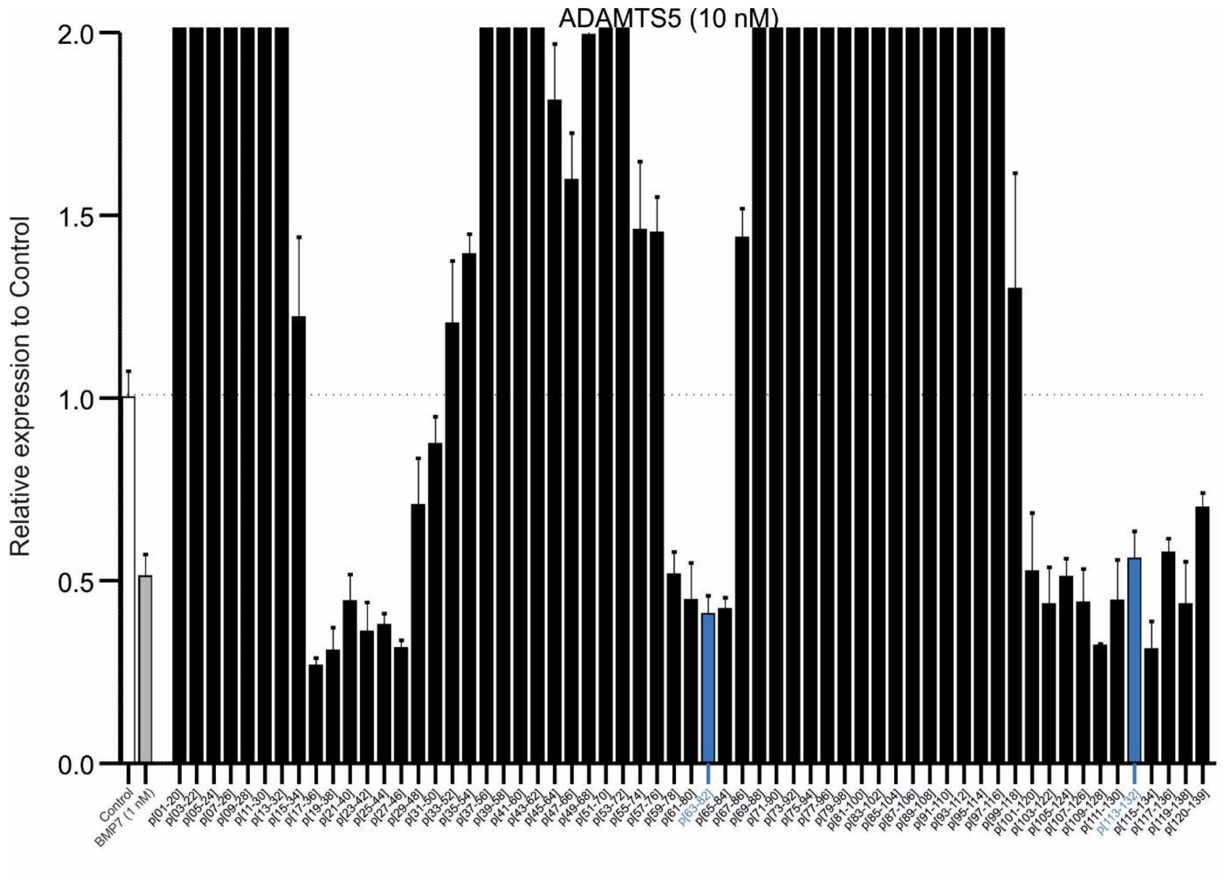
ADAMTS5 (10 nM)
Relative expression to Control
2.0
1.5
1.0
0.5
0.0
Control
BMP7 (1 nM)

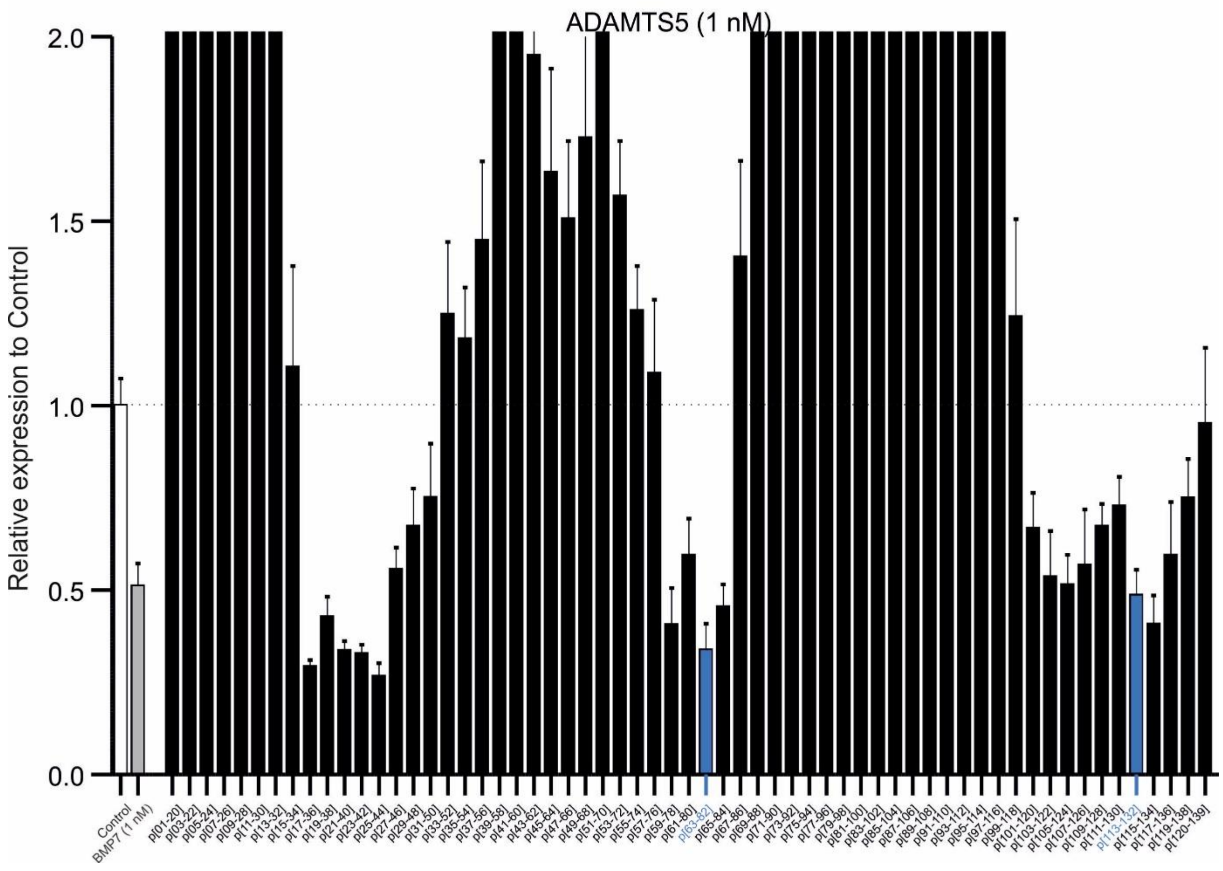
ADAMTS5 (1 nM)
Relative expression to Control
2.0
1.5
1.0
0.5
0.0
Control
BMP7 (1 nM)

P

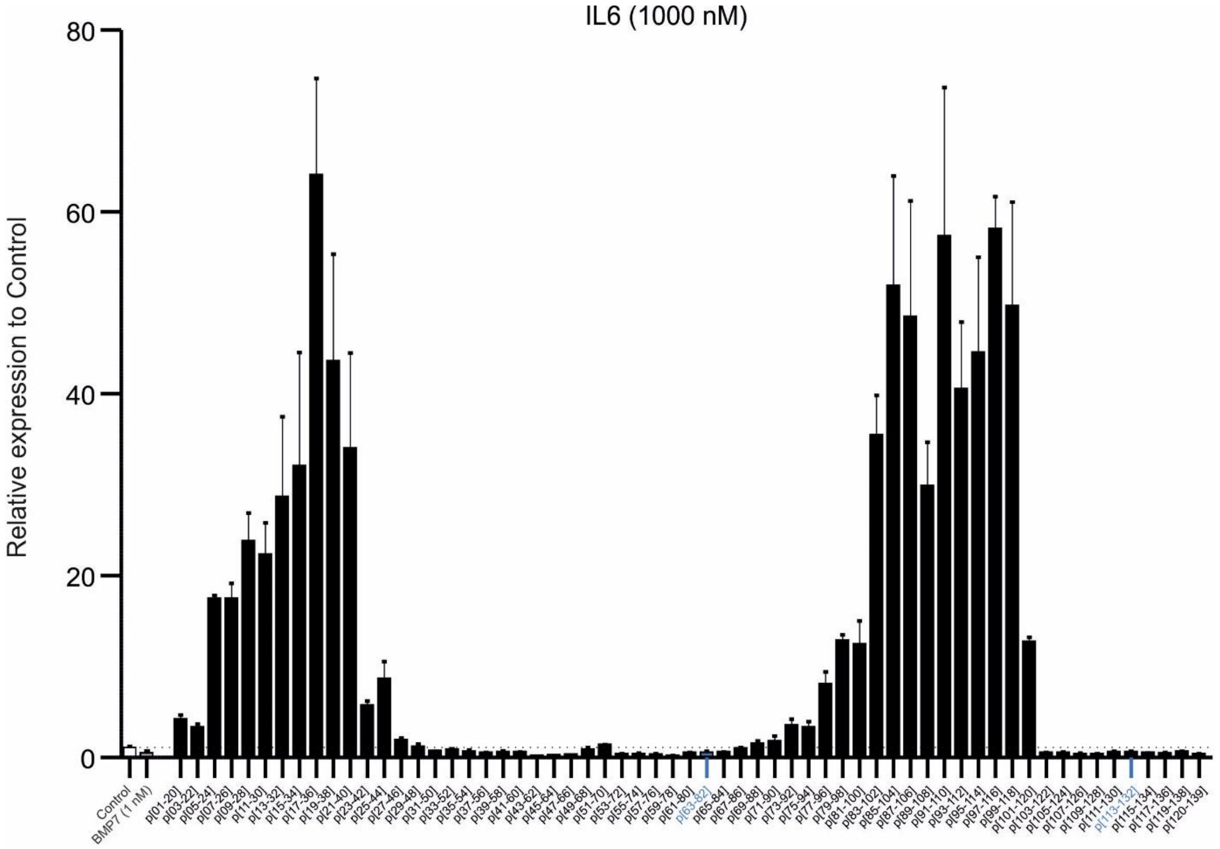
IL6 (1000 nM)
Relative expression to Control
80
60
40
20
0
Control
BMP7 (1 nM)

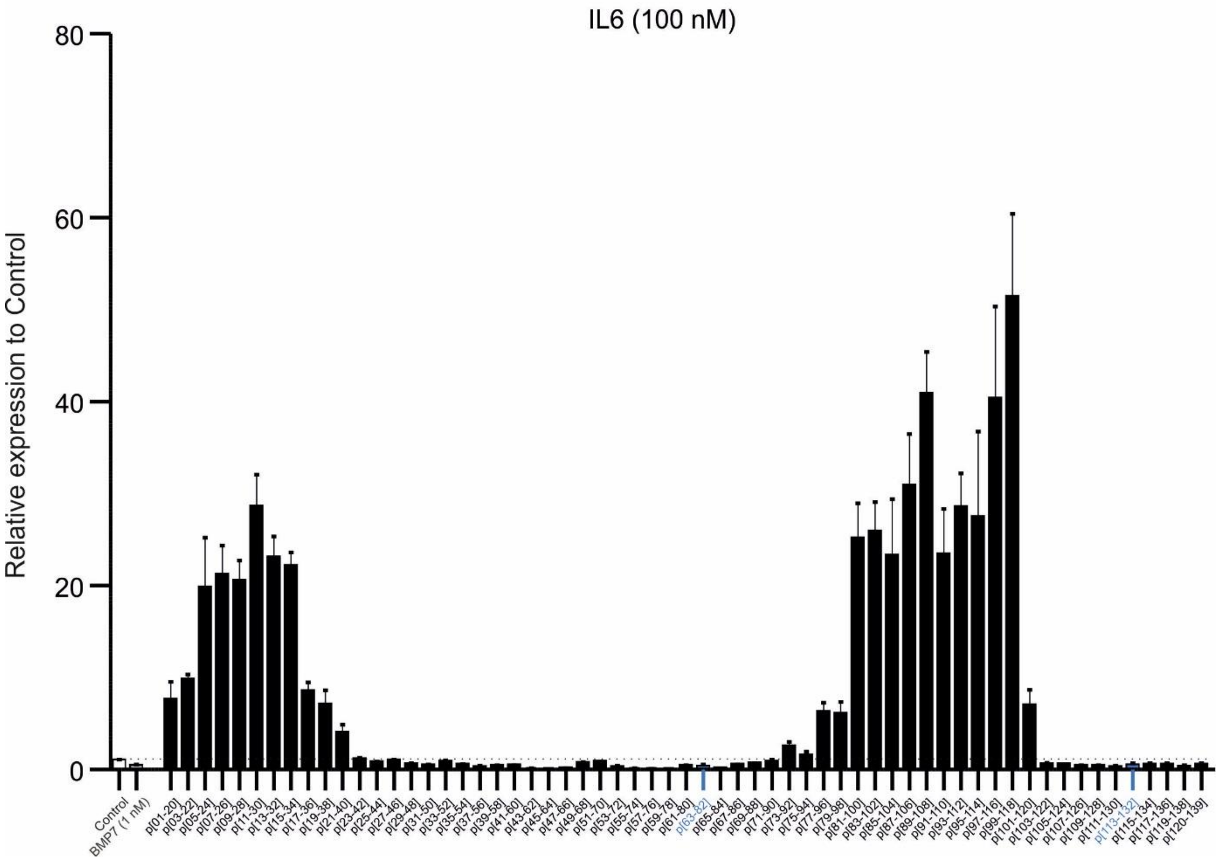
IL6 (100 nM)
Relative expression to Control
80
60
40
20
0
Control
BMP7 (1 nM)

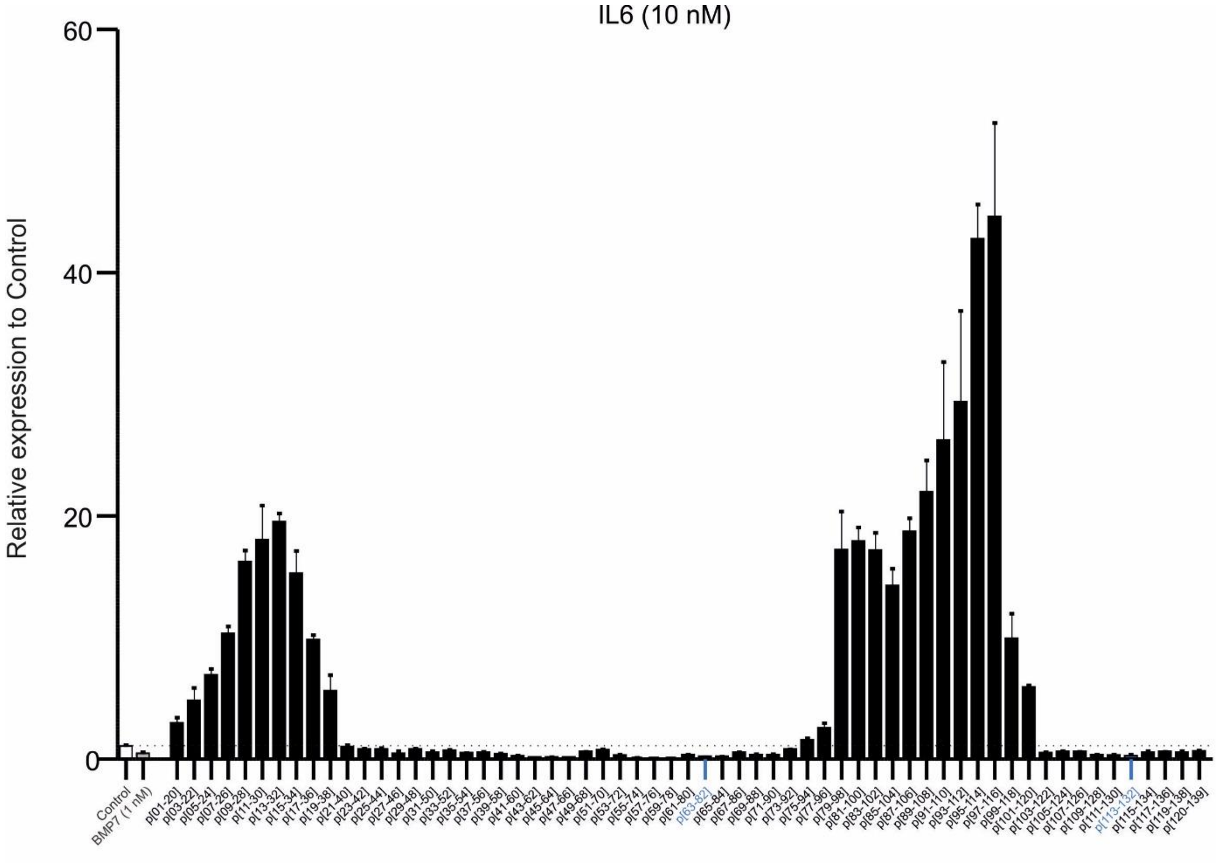
IL6 (10 nM)
Relative expression to Control
Control
BMP7 (1 nM)

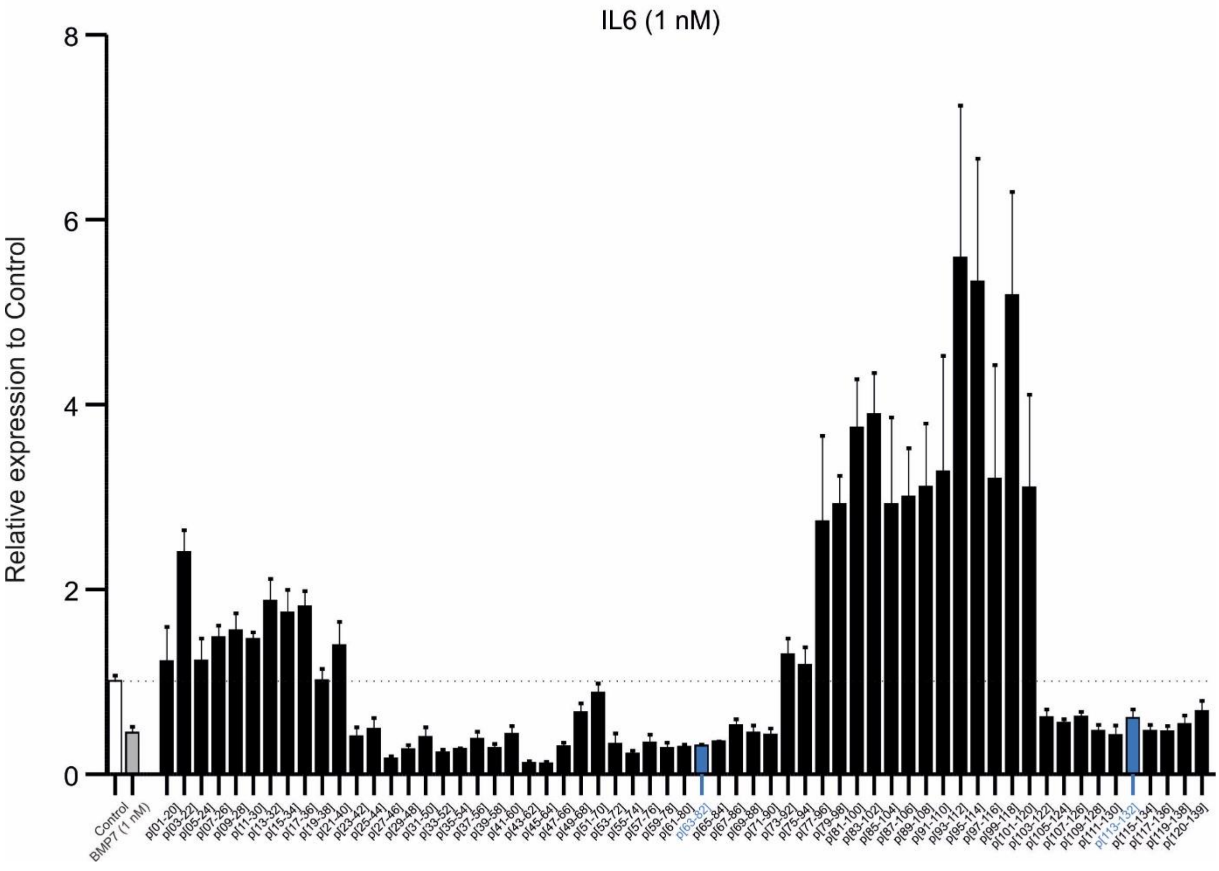
IL6 (1 nM)
Relative expression to Control
Control
BMP7 (1 nM)

Q

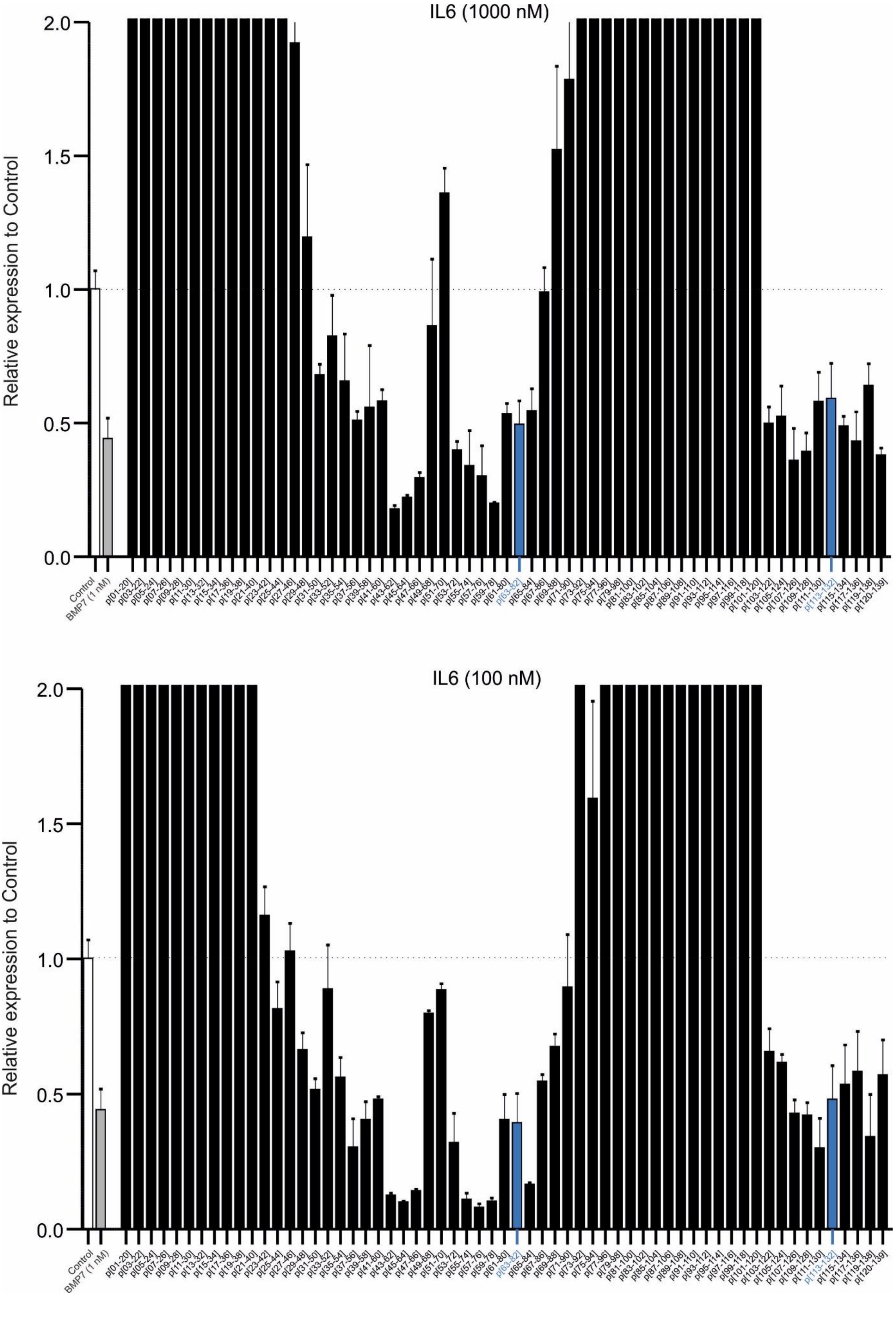
IL6 (1000 nM)
Relative expression to Control
2.0
1.5
1.0
0.5
0.0
Control
BMP7 (1 nM)
IL6 (100 nM)
Relative expression to Control
2.0
1.5
1.0
0.5
0.0
Control
BMP7 (1 nM)

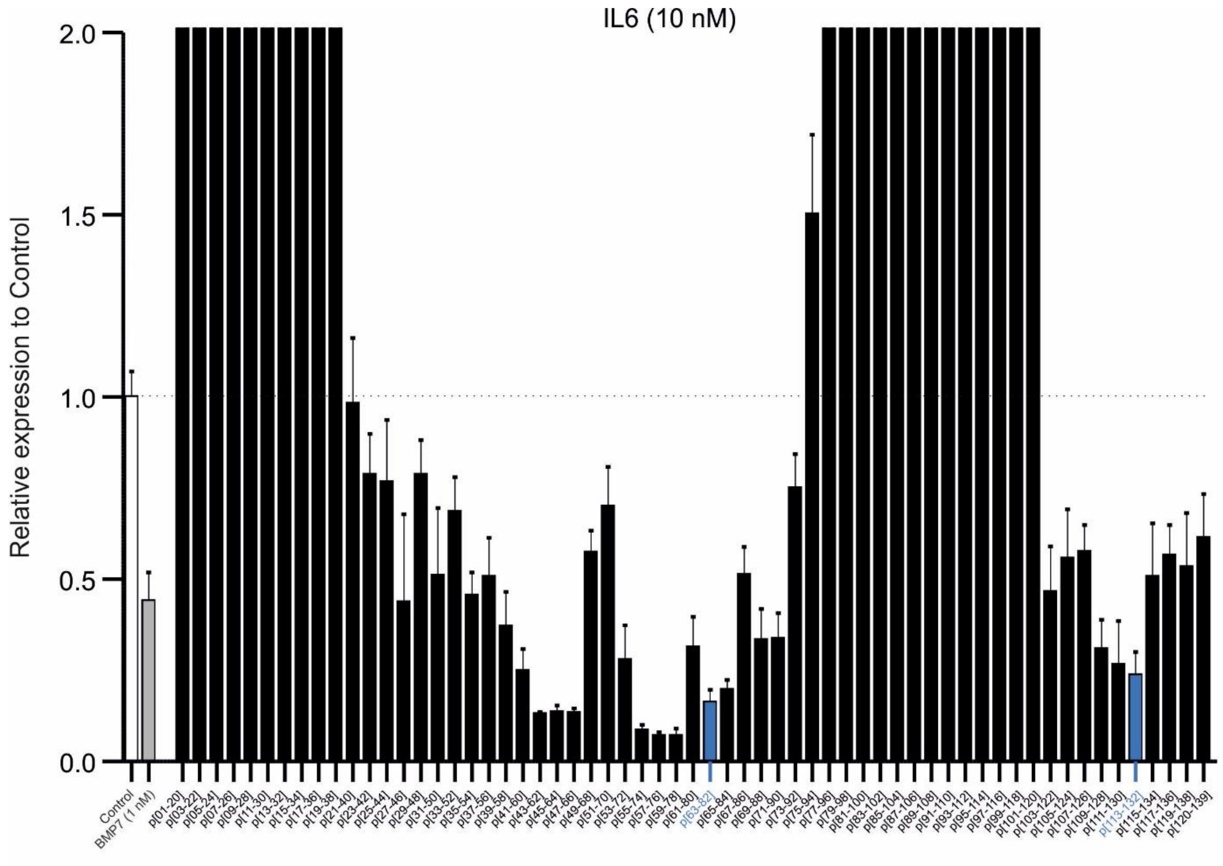
IL6 (10 nM)
Relative expression to Control
2.0
1.5
1.0
0.5
0.0
Control
BMP7 (1 nM)

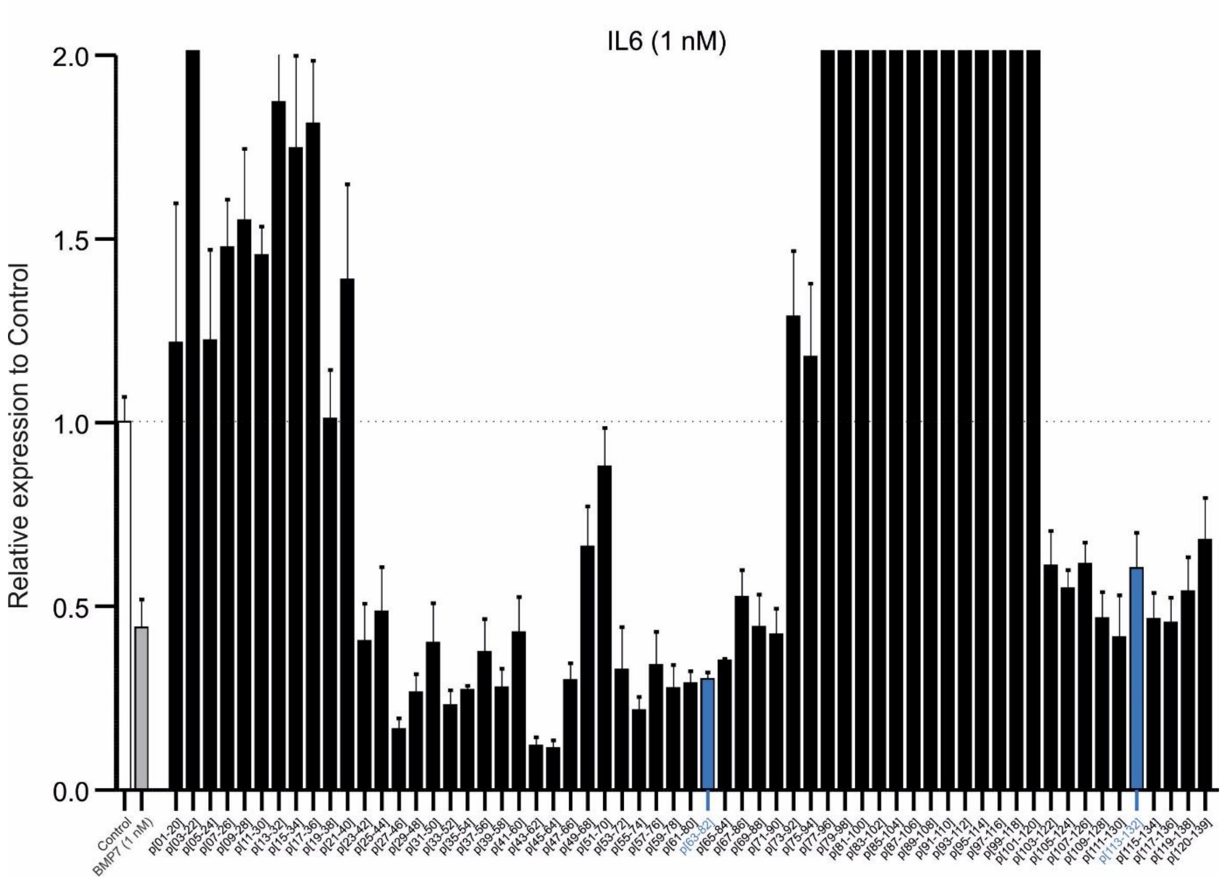
IL6 (1 nM)
Relative expression to Control
2.0
1.5
1.0
0.5
0.0
Control
BMP7 (1 nM)

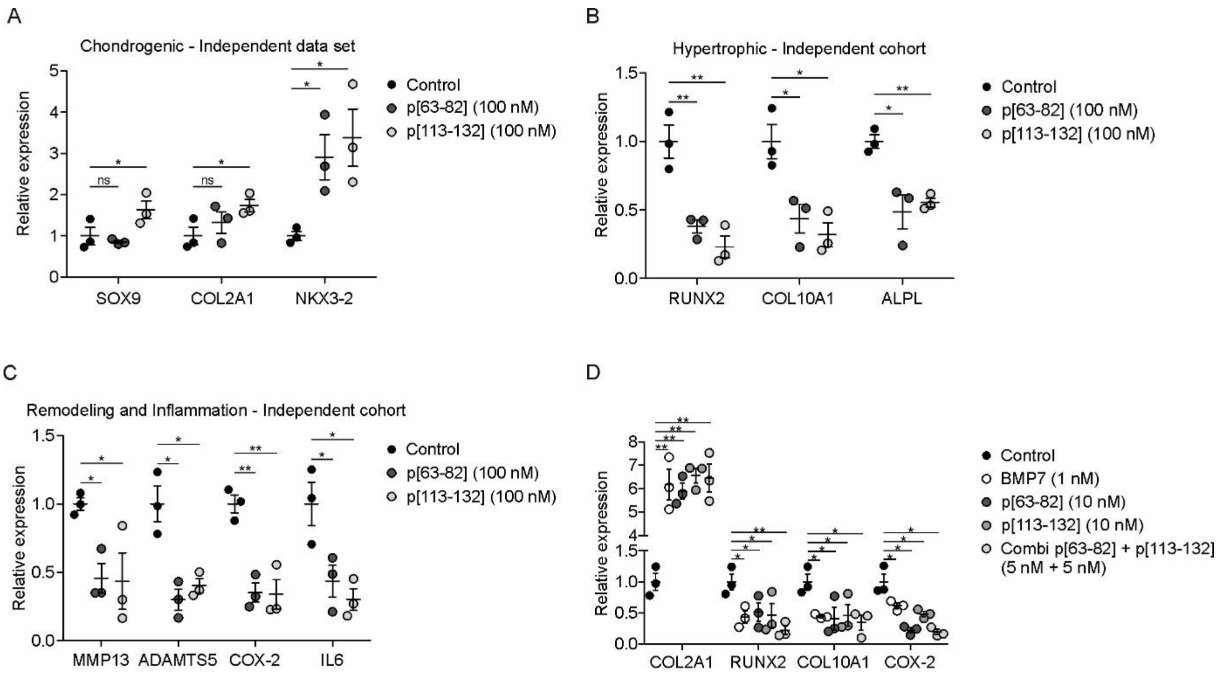

Supplementary Figure 3: Bioactivity of peptides p[63-82] and p[113-132] on independent OA-HAC cohort. A: Chondrogenic gene expression (SOX9, COL2A1, NKX3-2) in independent cohort of 3 individual OA-HAC donors. **B:** Hypertrophic gene expression (RUNX2, COL10A1, ALPL) expression in independent cohort of 3 individual OA-HAC donors. **C:** Remodelling (MMP13, ADAMTS5) and Inflammation (COX-2, IL6) gene expression in independent cohort of 3 individual OA-HAC donors. Conditions were: Control, p[63-82] and p[113-132]. Peptides were used at 100 nM each. **D:** Chondrogenic, hypertrophic and inflammatory gene expression (COL2A1, RUNX2, COL10A1, COX-2) in independent cohort of 3 individual OA-HAC donors. Conditions were: BMP7 (1 nM), Control, p[63-82] (10 nM), p[113-132] (10 nM), combination of p[63-82] + p[113-132] (5 + 5 nM). All OA-HAC cultures were exposed to peptides for 24 hours. Gene expression was normalized for 28S rRNA expression and set relative to control condition. Error bars represent mean ± SEM and statistical significance for peptide conditions versus control condition as determined by unpaired two-tailed student's t-test is represented as: * is p<0.05, ** is p<0.01, *** is p<0.001 and ns is=not significant.

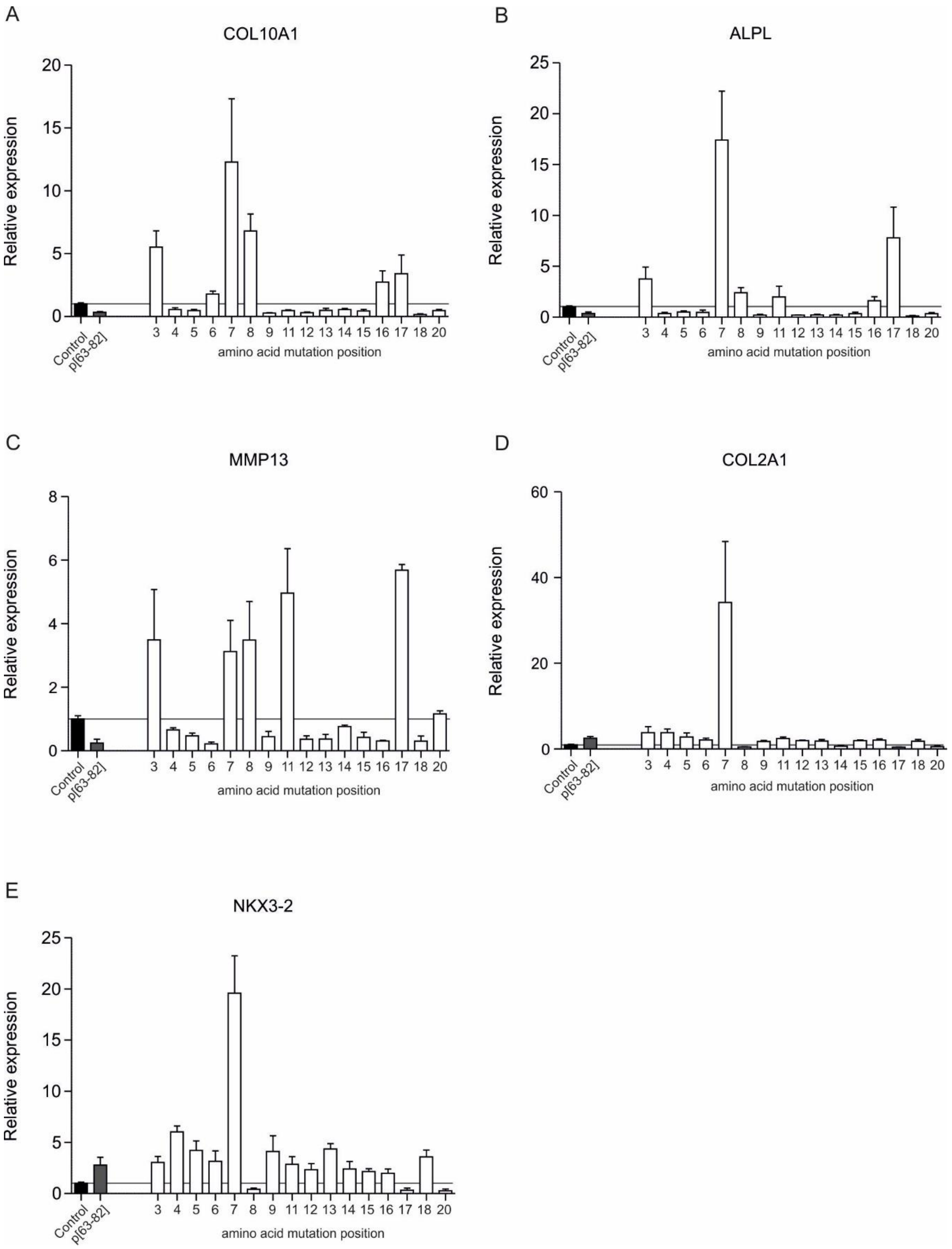

Supplementary Figure 4: Alanine scanning of peptide p[63-82]. A: COL10A1 mRNA expression in OA-HAC pool (n=18 individual donors). **B**: ALPL mRNA expression in OA-HAC pool (n=18). **C:** MMP13 mRNA expression in OA-HAC pool (n=18). **D:** COL2A1 mRNA expression in OA-HAC pool (n=18). **E:** NKX3-2 mRNA expression in OA-HAC pool

(n=18). Conditions were: Control, p[63-82] and peptides with amino acid substitution to alanine at indicated positions in p[63-82]. Peptides were used at 100 nM each. All OA-HAC cultures were exposed to peptides for 24 hours. Gene expression was normalized for 28S rRNA expression and set relative to control condition. Error bars represent mean ± SEM.

A

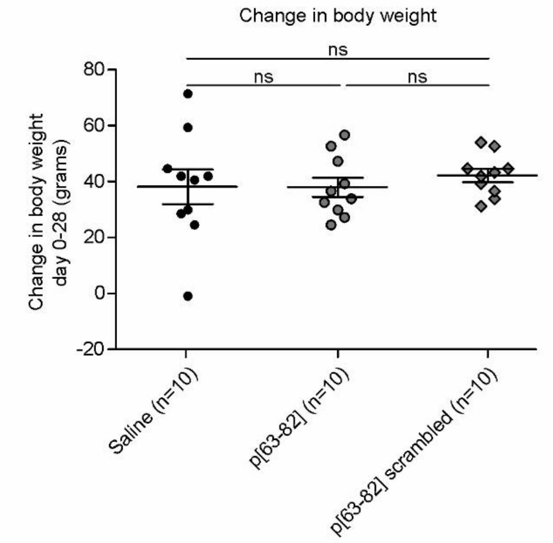

B

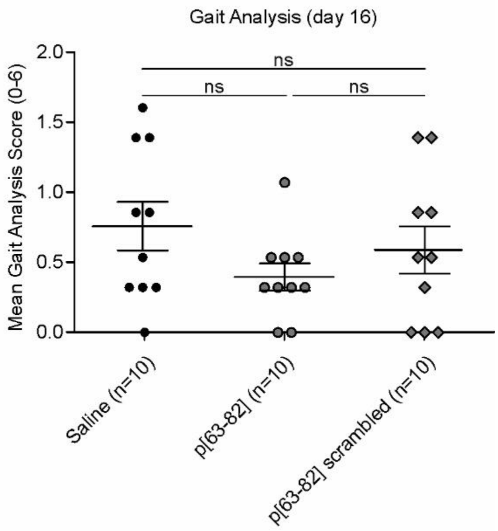

C

Parameter | Mean (SE)		Saline (n=10)	p[63-82] (n=10)	p[63-82] scrambled (n=10)	% Change p[63-82] vs Saline
**Medial Tibia Cartilage Degeneration Score**					
	Zone 1 (outside)	3,8 (0,2)	**2,6 (0,4)***	3,3 (0,4)	32%
	Zone 2 (middle)	1,2 (0,1)	1,0 (0,1)	1,2 (0,2)	17%
	Zone 3 (inside)	0,2 (0,1)	0,4 (0,1)	0,4 (0,1)	-100%
	Total	5,2 (0,2)	**4,0 (0,5)***	4,8 (0,6)	23%
**Medial Tibia Depth Ratio**					
	Zone 1 (outside)	0,93 (0,03)	**0,58 (0,11)****	0,74 (0,07)	38%
	Zone 2 (middle)	0,16 (0,05)	0,15 (0,03)	0,16 (0,05)	6%
	Zone 3 (inside)	0,01 (0,01)	0,02 (0,01)	0,02 (0,01)	-100%
	Total	0,37 (0,02)	**0,25 (0,03)***	0,30 (0,04)	32%
**Tibial Cartilage Degeneration Width (um)**					
	Substantial	595,0 (29,3)	370,0 (88,3)	495,0 (62,1)	38%
	Total	1500,0 (136,6)	1690,0 (116,9)	1660,0 (122,2)	-13%
**Total Medial Tibia Bone Score**					
	Damage	2,8 (0,2)	2,2 (0,3)	2,8 (0,3)	22%
	Sclerosis	2,8 (0,2)	2,5 (0,2)	2,6 (0,3)	11%
**Medial Femur Cartilage Degeneration Score**					
	Zone 1 (outside)	1,15 (0,3)	0,45 (0,1)	0,90 (0,2)	61%
	Zone 2 (middle)	0,40 (0,3)	0,05 (0,1)	0,10 (0,1)	88%
	Zone 3 (inside)	0,35 (0,3)	0,05 (0,1)	0,30 (0,3)	86%
	Total	1,90 (0,4)	**0,55 (0,1)****	1,30 (0,3)	71%
**Medial Tibia Osteophytes**					
	Measure (um)	489,0 (34,1)	500,0 (31,6)	494,0 (37,8)	-2%
	Score	3,8 (0,4)	3,8 (0,3)	3,7 (0,3)	0%
**Total Joint Score**					
	Without femur	9,0 (0,4)	7,9 (0,7)	8,5 (0,6)	12%
	With femur	11,1 (0,5)	**8,3 (0,9)***	9,9 (0,7)	25%
**Synovitis Score**					
		0,70 (0,1)	0,80 (0,2)	0,80 (0,1)	-14%

D

Parameter | Mean (SE)		Saline (n=10)	p[63-82] (n=10)	p[63-82] scrambled (n=10)	% Change p[63-82] vs Saline
Collagen Degeneration by Severity (% to total surface)					
	Total	46,2 (2,2)	48,5 (1,8)	43,3 (2,4)	-5%
	Severe	9,9 (2,5)	**3,0 (1,5)***	1,5 (1,5)	70%
	Marked	2,8 (1,3)	1,0 (0,7)	1,5 (1,1)	64%
	Moderate	1,3 (0,7)	0,5 (0,5)	3,0 (1,1)	62%
	Mild	6,1 (2,9)	9,2 (4,0)	11,4 (1,9)	-51%
	Minimal	26,5 (1,7)	35,0 (4,1)	25,9 (1,8)	-32%
Collagen Combined Degeneration (%)					
	Severe to Mild	20,0 (1,8)	13,7 (4,1)	17,4 (3,6)	32%
	Severe to Moderate	13,9 (2,9)	**4,5 (2,4)***	6,0 (2,7)	68%
	Mild to Minimal	32,4 (3,5)	**44,0 (3,0)***	37,3 (1,7)	-36%
	Severe to Marked	12,7 (2,7)	**4,0 (2,1)***	3,0 (2,1)	69%
	Moderate to Mild	33,7 (3,3)	**44,5 (2,6)***	40,3 (2,1)	-32%
Growth Plate Thickness (um)					
	Difference (Medial - Lateral)	28,0 (0,1)	40,0 (10,3)	32,0 (8,0)	-43%
Medial Collateral Ligament Thickness (um)					
		585,0 (20,1)	580,0 (12,3)	564,0 (27,0)	-1%

Supplementary Figure 5: BMP7-derived peptide p[63-82] in rat MMT model. The potency of BMP7 peptide p[63-82] to delay the progression of trauma-induced cartilage degeneration

was tested in the rat MMT model. One week post-MMT-surgery, rats were two times per week intra-articularly injected with saline, 100 ng peptide p[63-82] in saline or 100 ng scrambled peptide p[63-82] in saline (10 rats per group). All injection volumes were 50 µl. At four weeks post-MMT-surgery rats were sacrificed for histopathological scoring of the MMT knee joints. **A:** Change in body weight of the rats during the experiment (day 0-28). Conditions are indicated. **B:** Gait analysis scores at day 16 in the experiment **A/B**: Error bars represent mean ± SEM. **C:** Medial tibia cartilage degeneration score, Medial tibia depth ratio, Tibial cartilage degeneration width, Total medial tibia bone score, Medial femur cartilage degeneration score, Medial tibia osteophytes, Total joint score and Synovitis score are indicated in the table as mean (± SEM) for indicated conditions and percentage change of p[63-82] versus saline is presented in the last column. **D:** Collagen degeneration by severity, Collagen combined degeneration, Growth plate thickness and Medial collateral ligament thickness are indicated in the table as mean (± SEM) for indicated conditions and percentage change of p[63-82] versus saline is presented in the last column. Statistical differences were calculated between groups (Mann-Whitney U test) and * is $p<0.05$ ** is $p<0.01$, *** is $p<0.001$, and ns is not significant. Indicated scores are based on references: 1. Bendele, A.M., *Animal models of osteoarthritis.* J Musculoskelet Neuronal Interact, 2001. 1(4): p. 363-76. 2. Bendele, A.M., *Animal models of osteoarthritis in an era of molecular biology.* J Musculoskelet Neuronal Interact, 2002. 2(6): p. 501-3. 3. Gerwin, N., et al., *The OARSI histopathology initiative - recommendations for histological assessments of osteoarthritis in the rat.* Osteoarthritis Cartilage, 2010. 18 Suppl 3: p. S24-34.

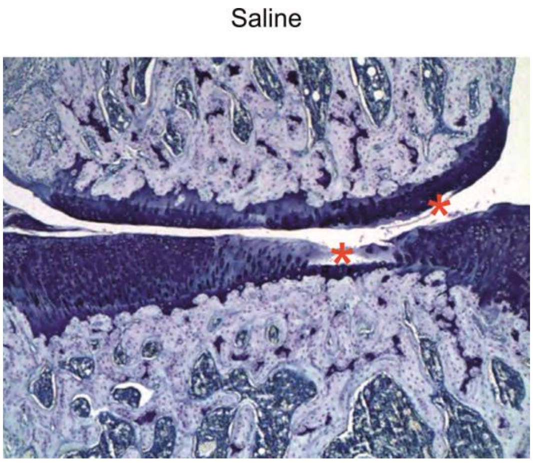

Supplementary Figure 6: Predominant cartilage lesion sites in the rat MMT experiment. Representative micrographs of toluidine blue-stained sections of medial aspects of the MMT knee joints. The predominant sites where lesions formed in this model in the tibial and femoral cartilage are indicated with an “*”.

SUPPLEMENTARY TABLES

Supplementary Table 1: Amino acid sequences of BMP7 peptide libraries

Peptide	Sequence	Peptide	Sequence
p[01-20]	H-STGSKQRSQNRSKTPKNQEA-OH	p[65-84]	H-YYSEGESAFPLNSYMNATNH-OH
p[03-22]	H-GSKQRSQNRSKTPKNQEALR-OH	p[67-86]	H-SEGESAFPLNSYMNATNHAI-OH
p[05-24]	H-KQRSQNRSKTPKNQEALRMA-OH	p[69-88]	H-GESAFPLNSYMNATNHAIVQ-OH
p[07-26]	H-RSQNRSKTPKNQEALRMANV-OH	p[71-90]	H-SAFPLNSYMNATNHAIVQTL-OH
p[09-28]	H-QNRSKTPKNQEALRMANVAE-OH	p[73-92]	H-FPLNSYMNATNHAIVQTLVH-OH
p[11-30]	H-RSKTPKNQEALRMANVAENS-OH	p[75-94]	H-LNSYMNATNHAIVQTLVHFI-OH
p[13-32]	H-KTPKNQEALRMANVAENSSS-OH	p[77-96]	H-SYMNATNHAIVQTLVHFINP-OH
p[15-34]	H-PKNQEALRMANVAENSSSDQ-OH	p[79-98]	H-MNATNHAIVQTLVHFINPET-OH
p[17-36]	H-NQEALRMANVAENSSSDQRQ-OH	p[81-100]	H-ATNHAIVQTLVHFINPETVP-OH
p[19-38]	H-EALRMANVAENSSSDQRQAS-OH	p[83-102]	H-NHAIVQTLVHFINPETVPKP-OH
p[21-40]	H-LRMANVAENSSSDQRQASKK-OH	p[85-104]	H-AIVQTLVHFINPETVPKPSS-OH
p[23-42]	H-MANVAENSSSDQRQASKKHE-OH	p[87-106]	H-VQTLVHFINPETVPKPSSAP-OH
p[25-44]	H-NVAENSSSDQRQASKKHELY-OH	p[89-108]	H-TLVHFINPETVPKPSSAPTQ-OH
p[27-46]	H-AENSSSDQRQASKKHELYVS-OH	p[91-110]	H-VHFINPETVPKPSSAPTQLN-OH
p[29-48]	H-NSSSDQRQASKKHELYVSFR-OH	p[93-112]	H-FINPETVPKPSSAPTQLNAI-OH
p[31-50]	H-SSDQRQASKKHELYVSFRDL-OH	p[95-114]	H-NPETVPKPSSAPTQLNAISV-OH
p[33-52]	H-DQRQASKKHELYVSFRDLGW-OH	p[97-116]	H-ETVPKPSSAPTQLNAISVLY-OH
p[35-54]	H-RQASKKHELYVSFRDLGWQD-OH	p[99-118]	H-VPKPSSAPTQLNAISVLYFD-OH
p[37-56]	H-ASKKHELYVSFRDLGWQDWI-OH	p[101-120]	H-KPSSAPTQLNAISVLYFDDS-OH
p[39-58]	H-KKHELYVSFRDLGWQDWIIA-OH	p[103-122]	H-SSAPTQLNAISVLYFDDSSN-OH
p[41-60]	H-HELYVSFRDLGWQDWIIAPE-OH	p[105-124]	H-APTQLNAISVLYFDDSSNVI-OH
p[43-62]	H-LYVSFRDLGWQDWIIAPEGY-OH	p[107-126]	H-TQLNAISVLYFDDSSNVILK-OH
p[45-64]	H-VSFRDLGWQDWIIAPEGYAA-OH	p[109-128]	H-LNAISVLYFDDSSNVILKKY-OH
p[47-66]	H-FRDLGWQDWIIAPEGYAAYY-OH	p[111-130]	H-AISVLYFDDSSNVILKKYRN-OH
p[49-68]	H-DLGWQDWIIAPEGYAAYYSE-OH	p[113-132]	H-SVLYFDDSSNVILKKYRNMV-OH
p[51-70]	H-GWQDWIIAPEGYAAYYSEGE-OH	p[115-134]	H-LYFDDSSNVILKKYRNMVVR-OH
p[53-72]	H-QDWIIAPEGYAAYYSEGESA-OH	p[117-136]	H-FDDSSNVILKKYRNMVVRAS-OH
p[55-74]	H-WIIAPEGYAAYYSEGESAFP-OH	p[119-138]	H-DSSNVILKKYRNMVVRASGS-OH
p[57-76]	H-IAPEGYAAYYSEGESAFPLN-OH	p[120-139]	H-SSNVILKKYRNMVVRASGSH-OH
p[59-78]	H-PEGYAAYYSEGESAFPLNSY-OH	p[63-82] scrambled	H-SEASPAMYYLNATANFYESG-OH
p[61-80]	H-GYAAYYSEGESAFPLNSYMN-OH	p[113-132] scrambled	H-VYISNVDDYSNKFLLKVRSM-OH
p[63-82]	H-AAYYSEGESAFPLNSYMNAT-OH		

Substitution position	Sequence	Substitution position	Sequence
Wildtype p[63-82]	H-AAYYSEGESAFPLNSYMNAT-OH	12	H-AAYYSEGESAFALNSYMNAT-OH
03	H-AAAYSEGESAFPLNSYMNAT-OH	13	H-AAYYSEGESAFPANSYMNAT-OH
04	H-AAYASEGESAFPLNSYMNAT-OH	14	H-AAYYSEGESAFPLASYMNAT-OH
05	H-AAYYAEGESAFPLNSYMNAT-OH	15	H-AAYYSEGESAFPLNAYMNAT-OH
06	H-AAYYSAGESAFPLNSYMNAT-OH	16	H-AAYYSEGESAFPLNSAMNAT-OH
07	H-AAYYSEAESAFPLNSYMNAT-OH	17	H-AAYYSEGESAFPLNSYANAT-OH
08	H-AAYYSEGASAFPLNSYMNAT-OH	18	H-AAYYSEGESAFPLNSYMAAT-OH
09	H-AAYYSEGEAAFPLNSYMNAT-OH	20	H-AAYYSEGESAFPLNSYMNAA-OH
11	H-AAYYSEGESAAPLNSYMNAT-OH		

Supplementary Table 2: siRNA sequences

mRNA	sense	antisense
NKX3-2	CCGAGACGCAGGUGAAAAUdTdT	AUUUUCACGUGCGUCUCGGdTdT

The oligonucleotide sequences are shown from 5’ to 3’. The 3’ termini were modified with two deoxythymidine nucleotides.

Supplementary Table 3: Oligonucleotide DNA sequences for RT-qPCR

(m)RNA	forward	reverse
28S rRNA	GCCATGGTAATCCTGCTCAGTAC	GCTCCTCAGCCAAGCACATAC
ADAMTS5	GTGGCTCACGAAATCGGACAT	GCGCTTATCTTCTGTGGAACCA
ALPL	AATGTCATCATGTTCCTGGGAGAT	TGGTGGAGCTGACCCTTGAG
NKX3-2	ACCTGGCAGCTTCGCTGAA	AGGTCGGCGGCCATCT
COL2A1	TGGGTGTTCTATTTATTTATTGTCTTCCT	GCGTTGGACTCACACCAGTTAGT
COL10A1	ATGATGAATACACCAAAGGCTACCT	ACGCACACCTGGTCATTTTCTG
COX-2	ACCAACATGATCTTTGCATTCTTT	GGTCCCCGCTTAAGATCTGTCT
IL6	TGTAGCCGCCCCACACA	GGATGTACCGAATTTGTTTGTCAA
MMP13	CTTCACGATGGCATTGCTGAC	CGCCATGCTCCTTAATTCCA
RUNX2	TGATGACACTGCCACCTCTGA	GCACCTGCCTGGCTCTTCT
SOX9	AGTACCCGCACCTGCACAAC	CGCTTCTCGCTCTCGTTCAG

DNA oligonucleotide sequences are shown from 5’ to 3’.

Supplementary Table 4: Medial Tibial and Femoral General Cartilage Degeneration Score

Score	Observation
0	No degeneration
0.5	Very minimal degeneration, within the zone less than 5% of the matrix has PG loss mainly with minor chondrocyte loss and little if any collagen matrix loss or damage
1	Minimal degeneration, within the zone 5-10% of the matrix appears non-viable as a result of significant chondrocyte loss (greater than 50% of normal cell density). PG loss is usually present in these areas of cell loss and collagen matrix loss may be present.
2	Mild degeneration, within the zone 11-25% of the matrix appears non-viable as a result of significant chondrocyte loss (greater than 50% of normal cell density). PG loss is usually present in these areas of cell loss and collagen matrix loss may be present.
3	Moderate degeneration, within the zone 26-50% of the matrix appears non-viable as a result of significant chondrocyte loss (greater than 50% of normal cell density). PG loss is usually present in these areas of cell loss and collagen matrix loss may be present.
4	Marked degeneration, within the zone 51-75% of the matrix appears non-viable as a result of significant chondrocyte loss (greater than 50% of normal cell density). PG loss is usually present in these areas of cell loss and collagen matrix loss may be present.
5	Severe degeneration, within the zone 76-100% of the matrix appears non-viable as a result of significant chondrocyte loss (greater than 50% of normal cell density). PG loss is usually present in these areas of cell loss and collagen matrix loss may be present.

General cartilage degeneration includes chondrocyte death/loss, proteoglycan (PG) loss, and collagen loss or fibrillation. Zones were scored individually according to the criteria in table and a sum of all three zones was calculated [1-3].

Supplementary Table 5: Medial Tibial and Femoral General Cartilage Degeneration Score

Medial Tibial Total Cartilage Degeneration Width	The width of the cartilage affected by any degeneration (cell loss, proteoglycan loss or collagen damage) was measured by ocular micrometer. This measurement extends from the origination of the osteophyte with adjacent cartilage degeneration (outside 1/3) across the surface to the point where tangential layer and underlying cartilage appear histologically normal [1-3].
Medial Tibial Substantial Cartilage Degeneration Width	Substantial Cartilage Degeneration was identified by chondrocyte and proteoglycan loss extending through greater than 50% of the cartilage thickness and was measured by ocular micrometer. In general, the collagen damage is mild (25% depth) or greater for this parameter but chondrocyte and proteoglycan loss extend to at least 50% or greater of the cartilage depth, indicating regions in which permanent structural changes have occurred [1-3].

Supplementary Table 6: Osteophyte Score and Measurement

Score	Observation	
0	None	Less than 200 μm
1	Small	200-299 μm
2	Medium	300-399 μm
3	Large	400-499 μm
4	Very large	500-599 μm
5	Very large	≥600 μm

Osteophyte thickness (tidemark to furthest point extending toward synovium) was measured with an ocular micrometer. Scores were assigned to the largest osteophyte in each section (typically found on the tibia) according to the criteria in the table [1-3].

Supplementary Table 7: Medial Tibial Bone Sclerosis Score

Score	Observation	
0	Normal	No observable difference in subchondral or epiphyseal trabecular bone thickness in medial vs. lateral.
1	Minimal	5-10% increase in subchondral or epiphyseal trabecular bone thickness in medial vs. lateral.
2	Mild	11-25% increase in subchondral or epiphyseal trabecular bone thickness in medial vs. lateral
3	Moderate	26-50% increase in subchondral or epiphyseal trabecular bone thickness in medial vs. lateral, obvious reduction in marrow spaces in outer 3/4 of medial tibia
4	Marked	51-75% increase in subchondral or epiphyseal trabecular bone thickness in medial vs. lateral, generally has very little marrow space in outer 3/4 of medial tibia, marrow spaces remain adjacent to cruciates.
5	Servere	76-100% increase in subchondral or epiphyseal trabecular bone thickness in medial vs. lateral, generally has very little marrow space remains in medial tibia

Medial tibial subchondral/epiphyseal bone thickening/sclerosis was scored based on the criteria in the table using a comparison to the lateral tibia and/or normal left medial tibias [1-3].

Supplementary Table 8: Total Joint Scores

The three-zone sums of the tibial and femoral cartilage degeneration scores, and the osteophyte score were summed to determine a total joint score. A sum of tibial cartilage degeneration and osteophyte scores without the femur was also calculated [1-3]

Supplementary Table 9: Synovitis Score

Score	Observation
0	Normal synovium
0.5	Very minimal synovitis (generally focal or scattered minimal diffuse).
1	Minimal synovitis (generally focal or scattered minimal diffuse).
2	Mild synovitis (multifocal to confluent areas of mild mononuclear cell infiltration).
3	Moderate synovitis (confluent areas of moderate mononuclear cell infiltration).
4	Marked synovitis (confluent areas of marked mononuclear cell infiltration).
5	Severe synovitis (confluent areas of severe mononuclear cell infiltration).

Synovial inflammation (mainly mononuclear cell infiltration concentrated on the medial side) was scored as indicated in Table 9 [1-3].

Supplementary Table 10: Medial Tibial Collagen Degeneration Severity

Any Damage	Fibrillation ranging from superficial to full thickness loss.
Severe Damage	Total or near total loss of collagen to tidemark, >90% thickness.
Marked Damage	Extends through 61-90% of the cartilage thickness.
Moderate Damage	Extends through 31-60% of the cartilage thickness.
Mild Damage	Extends through 11-30% of the cartilage thickness.
Minimal Damage	Very superficial, affecting upper 10% only.

Collagen damage across the medial tibial plateau (most severely affected section of the two halves) was quantified by measuring the total width of the collagen degeneration severities indicated in the table using an ocular micrometer. Measurements were expressed as a percentage of the total tibial surface width [1-3].

Supplementary Table 11: Growth Plate Thickness

Growth plate thickness was measured in all knees on medial and lateral sides (2 measurements per joint) at the approximate midpoint of the medial and lateral physis (assuming a non-tangential area of the section) using an ocular micrometer. The lateral thickness was also subtracted from the medial to determine the difference between the two [1-3].

Supplementary Table 12: Medial Collateral Ligament/Synovial Repair

Measurements were made of the thickness of the medial synovial/collateral ligament repair in a non-tangential area of the section using an ocular micrometer [1-3]

Supplementary Table 13: Gait analysis

Score	Observation
0	Normal, approximately equal ink staining to normal paw.
1	Slight limp/pain. Reduced inking area relative to the normal paw, but no full regions or structures are missing.
2	Mild limp/pain. Print extends to the end or near to the end of the “curlicue” structure. If normal paw has very little heel staining (rat walks mainly on toes/ball of foot), then slightly less staining.
3	Moderate limp/pain. Toes and full ball of foot, extending to the top of the “curlicue” structure are present. If normal paw has very little heel staining (rat walks mainly on toes/ball of foot), then toes with small portion of ball of foot.
4	Marked limp/pain. Toes and partial ball of foot, no heel or posterior foot. If normal paw has very little heel staining (rat walks mainly on toes/ball of foot), then toes only.
5	Severe limp/pain. Toes only, no ball of foot, no heel. If normal paw has very little heel staining (rat walks mainly on toes/ball of foot), then partial toes or non-specific marks.
6	Hopping. Carrying leg, no footprint is evident.

Gait analysis was performed on day 16 to confirm expected animal mobility post-surgery. Gait was evaluated by applying ink to the ventral surface of the foot and documenting weight bearing during movement (footprints) across paper. Rear feet of rats were placed in ink, then rats were placed on paper and allowed to walk the full length. This process was repeated as necessary to generate 4 clear, evenly inked footprint pairs representing the overall pattern of gait. Gait was scored visually as described in the table (descriptions refer to diseased leg) [1-3].

References

1. Bendele, A.M., *Animal models of osteoarthritis.* J Musculoskelet Neuronal Interact, 2001. **1**(4): p. 363-76.
2. Bendele, A.M., *Animal models of osteoarthritis in an era of molecular biology.* J Musculoskelet Neuronal Interact, 2002. **2**(6): p. 501-3.
3. Gerwin, N., et al., *The OARSI histopathology initiative - recommendations for histological assessments of osteoarthritis in the rat.* Osteoarthritis Cartilage, 2010. **18 Suppl 3**: p. S24-34.
